# All-metal aromatic cationic palladium triangles can mimic aromatic donor ligands with Lewis acidic cations†
†Electronic supplementary information (ESI) available: Reaction procedures, characterization of complexes, copies of all spectra and cif files, modelling details and *XYZ* coordinates. CCDC 1410440–1410442. For ESI and crystallographic data in CIF or other electronic format see DOI: 10.1039/c7sc03475j



**DOI:** 10.1039/c7sc03475j

**Published:** 2017-08-29

**Authors:** Yanlan Wang, Anna Monfredini, Pierre-Alexandre Deyris, Florent Blanchard, Etienne Derat, Giovanni Maestri, Max Malacria

**Affiliations:** a ICSN CNRS (UPR2301) , 1 Av. de la Terrasse, Bat. 27 , 91198 Gif s/Yvette , France; b UPMC Sorbonne Université , IPCM (UMR CNRS 8232) , 4 place Jussieu, C. 229 , 75005 Paris , France; c Dipartimento SCVSA , Università degli Studi di Parma , 17/A Parco Area delle Scienze , 43124 Parma , Italy . Email: giovanni.maestri@unipr.it

## Abstract

We present that cationic rings can act as donor ligands thanks to suitably delocalized metal–metal bonds. This could grant parent complexes with the peculiar properties of aromatic rings that are crafted with main group elements. We assembled Pd nuclei into equilateral mono-cationic triangles with unhindered faces. Like their main group element counterparts and despite their positive charge, these noble-metal rings form stable bonding interactions with other cations, such as positively charged silver atoms, to deliver the corresponding tetranuclear dicationic complexes. Through a mix of modeling and experimental techniques we propose that this bonding mode is an original coordination-like one rather than a 4-centre–2-electron bond, which have already been observed in three dimensional aromatics. The present results thus pave the way for the use of suitable metal rings as ligands.

## Abstract 2

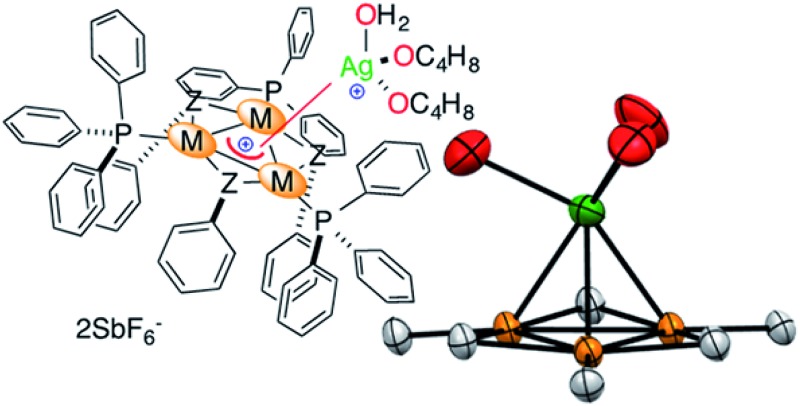
Replacing aromatic donor ligands with cationic Pd_3_^+^ complexes.

## Introduction

**Fig. 1:**
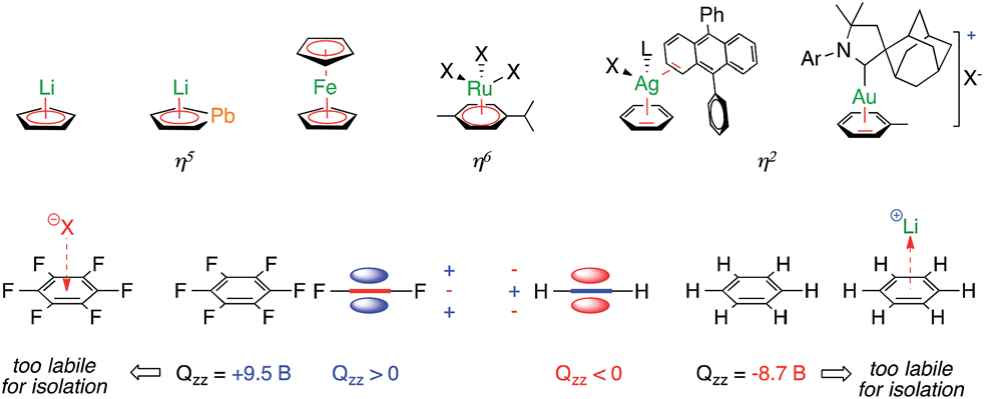
Selected stable complexes featuring covalent metal–aromatic bonding (top) and non-covalent ion–arene interactions (bottom).

Aromaticity is a fascinating chemical concept. It provides a unifying picture to account for and predict the properties of millions of structures that share the presence of delocalized bonding orbitals.^1^ The success of this concept can be perfectly witnessed by its growth since the historical proposition of the structure of benzene 150 years ago, even though this success eventually blurred the concept itself.^2^ Delocalized molecular orbitals of regular aromatics can interact with ions. Anionic and neutral aryl rings are ubiquitous ligands in organometallic chemistry (Fig. 1, top). Neutral arenes can often rely on their quadrupolar moment only to interact with Lewis acids.^3^ Among the π-acidic group XI elements, a few η^2^ silver-benzene structures have been reported^4^ and a gold-toluene analogue entered the scene more recently.^5^ Besides coordination chemistry, non-covalent cation–π interactions are nonetheless well documented in neutral main group aromatics and play a crucial role in chemistry and biology.^6^ Cations form these bonding interactions with arenes that have a negative quadrupolar moment perpendicular to their plane (*Q*_*zz*_, Fig. 1, bottom), while anions form these interactions with those that have a positive one.^7^

For decades, chemists have only played with a few nuclei to construct aromatics, mostly H, C, N and O. However, suitably delocalized molecular orbitals can be found in structures featuring transition metal atoms too.^8^ Although all-metal aromatics have been intensively studied *in silico*,^9^ their applications actually fell far shorter than the predicted expectations.^10^ We recently reported the assembly of [Pd_3_]^+^ analogues of the cyclopropenyl cation.^11^ A second-generation one-pot synthesis allowed us to access a larger library of structures including [Pt_3_]^+^ and mixed [Pd_2_Pt]^+^ or [PdPt_2_]^+^ derivatives.^11*b*^ This ensured a stable supply chain of these molecules and enabled us to gain insight into their catalytic behavior towards alkynes,^11c,d^ which is one of the rare applications of small palladium clusters in catalysis and arguably one of the best semi-reduction methods based on this metal known to date too, confirming the original nature of the metal–metal bonding in these [Pd_3_]^+^ complexes.

**Fig. 2:**
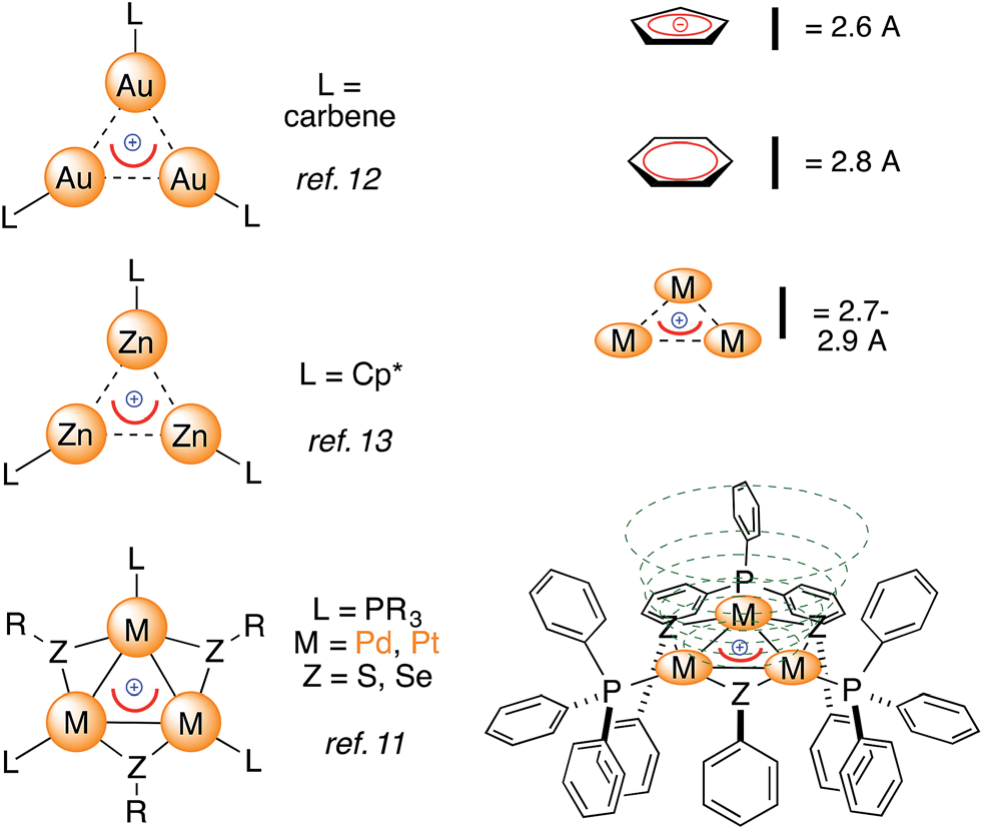
Stable trinuclear all-metal aromatic [M_3_]^+^ complexes (left) and a comparison to regular electron-rich aromatics (right); the values in Å refer to the height of each polygon.

The height of the equilateral metallic core of stable all-metal aromatic [M_3_]^+^ complexes^11–13^ closely matches that of the benzene hexagon or the cyclopentadienyl pentagon (Fig. 2), whose π clouds are routinely used to coordinate Lewis acids. The organic arms of the [Pd_3_]^+^ and [Pt_3_]^+^ complexes leave the core of these prototypical metal surfaces unhindered.^11^ We reasoned that their delocalized metal–metal bonds might therefore interact with other ions, either through non-covalent bonding or *via* coordination.

**Fig. 3:**
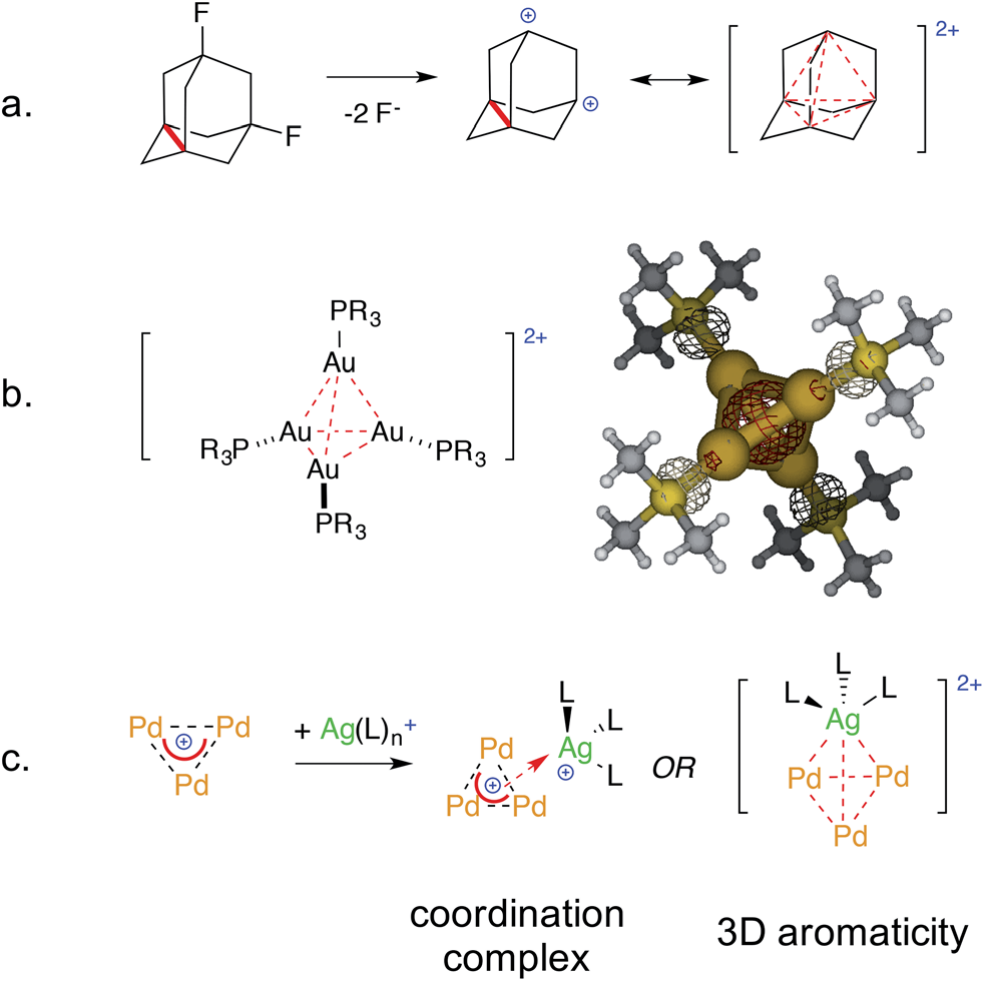
The 4-center–2-electron bond of the adamantyl dication (a, ref. 15) and its noble-metal counterpart highlighting its delocalized 4c–2e metal–metal bond (b, spherical red basin inside the metal cage, ref. 16) *versus* the present work (c).

Examples of bonding interactions between cationic nuclei are limited to very few rigidly tethered structures, such as the adamantyl dication^14^ (Fig. 3, top) and certain disulfonium species.^15^ The former represents a prototypical example of three-dimensional aromaticity.^9,10^ Its σ-bonding HOMO represents a 4-center–2-electron bond that grants its core with perfect tetrahedral symmetry. This rare and fascinating bonding mode has a metal-based analogue. Pyykkö and Runeberg^16*a*^ described the bonding of [(LAu)_4_]^2+^, a minor product formed in 5% yield from mononuclear Au(i) precursors,^16*b*^ as essentially identical to that of the adamantyl dication. Its 4-center–2-electron bond tethers together four equivalent Au nuclei that thus share the formal 1/2 oxidation state. This bonding mode is in agreement with its structure; the gold nuclei describe the vertices of a perfect trigonal pyramid. Their equivalence is confirmed by the arrangement of phosphines too, which are not coplanar with any Au_3_ face, deviating by around 30°.

Computational studies modeling anionic and neutral all-metal aromatics have predicted their ability to behave as ligands.^17^ Murahashi reported the assembly of stacking complexes sandwiching all-metal aromatic Pd_4_ units with two regular carbon-based units.^18^ To the best of our knowledge, the *intermolecular* addition of evenly charged fragments to form stable molecules, either through 4-center–2-electron bonds or *via* coordination, as described hereafter, has no precedent. We elicited experimentally a similar bonding event.

We report herein that trinuclear cationic frameworks featuring delocalized metal–metal bonds can interact with unsaturated metal cations thanks to two d-type electrons that were originally delocalized among the [Pd_3_]^+^ triangle (Fig. 3). Cationic all-metal aromatics can therefore mimic neutral or anionic donor ligands that are assembled by combining main group elements.

## Results and discussion

The project begun *in silico* with an unexpected observation. Preliminary modeling on [M_3_]^+^ provided largely negative quadrupolar moment values. This would be expected from electron rich, neutral or anionic species instead.^6,7^ Depending on the model, the calculated *Q*_*zz*_ values of the [M_3_]^+^ cations outmatched that of benzene, the benchmark among regular aromatics, by four to seven times. We observed this trend among different levels of theory (HF and DFT), functionals (exchange–correlation and orthodox hybrid ones) and basis sets (both double and triple-*ζ* ones for metal atoms). This minimized the odds of modeling artifacts. The largely negative values of [M_3_]^+^ aromatics suggested that they should interact with other cations.

We thus modeled a naked Li^+^ 6.0 Å above the planar core of the [M_3_]^+^ *cationic* complexes (details are in the ESI†).

In striking contrast to reasonable expectation, in which electrostatic repulsion should have simply pushed the two cations away without constraints,^19^ the optimizations converged eventually leaving Li^+^ 2.11–2.13 Å above the center of the [M_3_]^+^ triangles. The M–M distances remained basically untouched (at around 2.9 Å for all of the [M_3_]^+^ complexes) and the three M–Li ones were nearly identical, at around 2.4 Å. Each resulting metal-edged tetrahedron is thus an irregular pyramid, with an almost equilateral homonuclear base and a significantly shorter height.

**Fig. 4:**
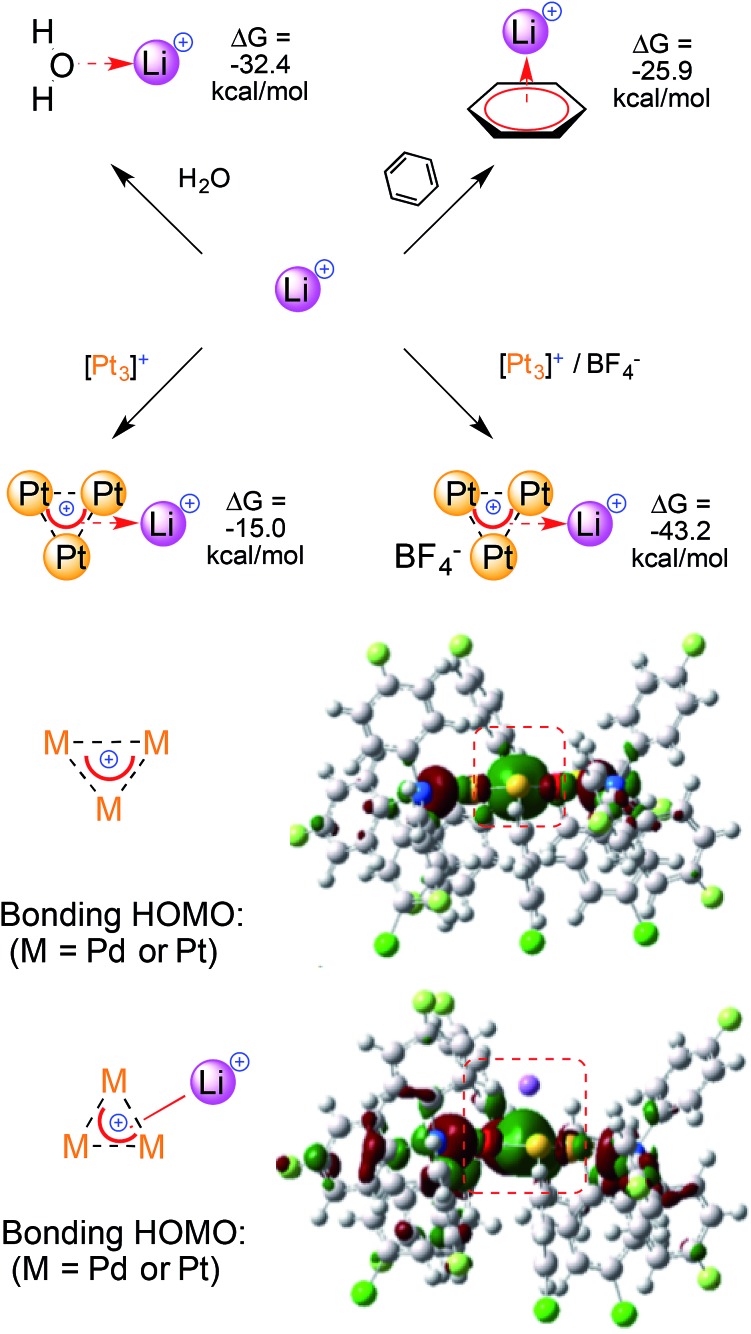
A comparison of the Δ*G* values for Li^+^ complexation by regular ligands and [M_3_]^+^ (top) and the Frontier orbitals involved (bottom); the values were obtained at the M06/Def2-SVP level.

The calculated binding of Li^+^ by the [Pt_3_]^+^ cation has a Δ*G* of –15.0 kcal mol^–1^. The Δ*G* value sinks to –43.2 kcal mol^–1^ when adding a non-coordinating BF_4_^–^ ion to model a monocationic adduct (Fig. 4, top, the anion remains outside of the first coordination sphere of the metals). The large increase in the calculated Δ*G* values reflects a lower destabilizing electrostatic contribution and highlights the magnitude of the calculated (metal)aromatic–cation bonding interaction. Comparable results were obtained when modeling the homo- and heteronuclear [M_3_]^+^ complexes, although the Δ*G* values became slightly less negative when replacing the Pt atoms with Pd ones. Indeed, the values when modeling [Pt_2_Pd]^+^, [PtPd_2_]^+^ and [Pd_3_]^+^ were –41.1, –39.3 and –37.9 kcal mol^–1^, respectively (for a possible rationalization *vide infra*). For comparison, calculated Δ*G* values for Li^+^ binding by neutral ligands were –32.4 and –25.9 kcal mol^–1^ using water and benzene, respectively. It should be noted, however, that concentration effects could not be taken into account by this computational approach, as they have been thoroughly studied in ion–π interactions.^6,7^ The observation of stable adducts is indeed not possible in the presence of large molar excesses of competitive ligands, such as water. In other words, concentration effects can trump the trend of the calculated Δ*G* values and dictate complexation equilibria, precluding experimental access to complexes that feature this bonding mode.

The delocalized bonding HOMOs of [M_3_]^+^ have sigmoidal symmetry that overlap with the LUMO of Li^+^, which is an empty 2s atomic orbital. The calculated HOMO of [M_3_Li]^++^ remains similar and its central lobe slightly expands towards the alkaline atom (Fig. 4, bottom). Similar bonding modes have limited experimental analogies. Li^+^ complexation was observed by an *anionic*, mixed organo-lead aromatic.^20^

We then tried to translate this promising modeling event to the real world, trying to react [Pd_3_]^+^ complexes with simple lithium salts under mild conditions.

**Fig. 5:**
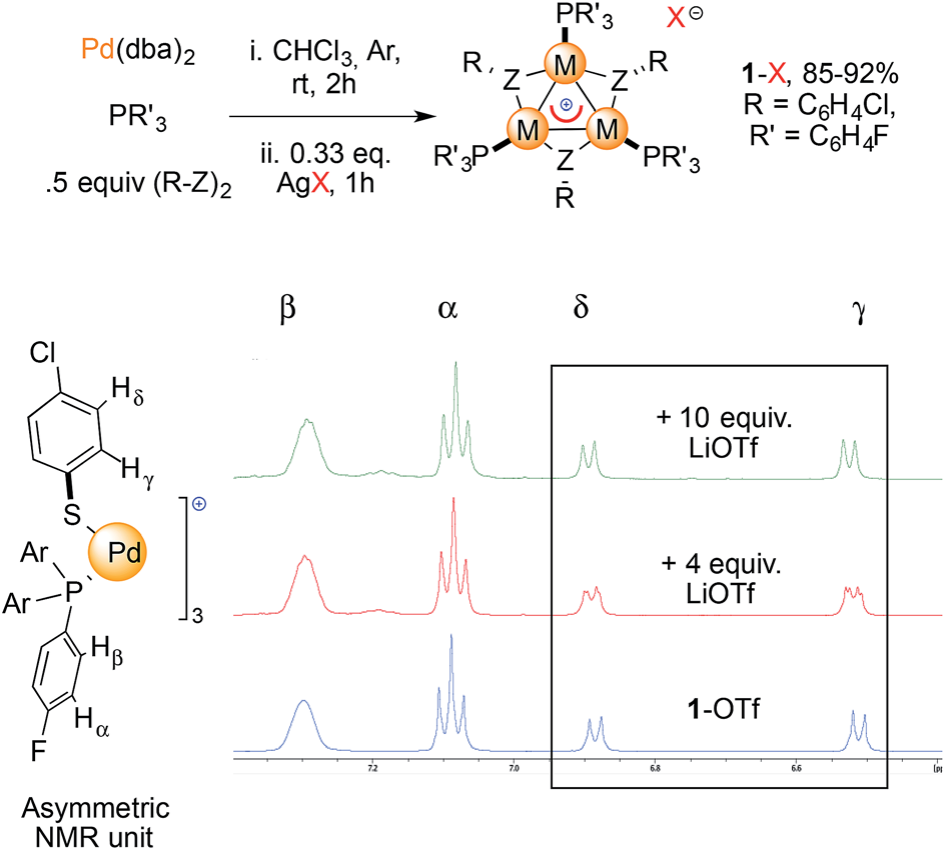
Synthesis of complexes **1**-X used in this study and ^1^H NMR spectra in acetone-*d*_6_ recorded upon treatment of **1**-OTf with LiOTf (red and green line).

We prepared a small library of [Pd_3_]^+^ complexes with various counterions (**1**-X, X = CF_3_SO_3_^–^, BF_4_^–^, SbF_6_^–^ Fig. 5, top). Complexes were formed by mixing Pd(dba)_2_ (dba = dibenzylideneacetone) with 1 equiv. of tris(*p*-fluorophenyl)phosphine and 0.5 equiv. of *p*-chlorophenyldisulfide for one hour and then adding 0.33 equiv. of the desired AgX salt according to our previously developed method.^11*b*^ This combination of phosphine and disulfide proved to be the most favorable to easily grow crystals of **1**-X, which streamlined their purification to obtain reasonable amounts for our further use. We then mixed each of these **1**-X complexes with the corresponding lithium salt in dry THF at room temperature (Fig. 5, bottom). To our delight, the resonances of the thiolate protons in the [Pd_3_]^+^ complex **1**-OTf (OTf = CF_3_SO_3_^–^) appeared to be split upon treatment with 4 equiv. of lithium triflate (red line, two apparent dd peaks centered at 6.88 and 6.52 ppm). The reaction of **1**-OTf with 10 equiv. of lithium triflate showed a single pattern of resonances instead (green line). The thiolate protons provided signals that evenly corresponded to those that appeared in the previous case. These resonances showed a slight but definite shifting (0.01–0.02 ppm) compared to those of the parent complex **1**-OTf (blue line). In contrast, the two groups of protons on the phosphine group P(4-ClC_6_H_4_)_3_ did not shift at all during these experiments. Treatment of tetrafluoroborate [Pd_3_]^+^ complex **1**-BF_4_ with 4 and 10 equiv. of LiBF_4_ provided an identical trend. The shifts of the peaks in the ^1^H NMR spectra were between 0.02 and 0.04 ppm. We observed small, discrete shifts when analyzing the resonances of ^19^F and ^31^P nuclei in all cases. These results are surely too close to call but nonetheless proved to be fully reproducible by two operators.

Taken together, these results could be consistent with the formation of new molecular species in solution. Unfortunately, several attempts to detect any complex using high-resolution mass spectroscopy (HRMS) proved fruitless, invariably showing just cation **1**. We reasoned that electrospray ionization (ESI) might have been too severe to preserve the labile [Pd_3_]–Li interactions, so we tried a variety of methods to obtain crystals that would be suitable for X-ray diffraction. Try as we might, our efforts were unsuccessful. This could correlate with regular aromatics, for which ion–π interactions are weak non-covalent events that preclude the isolation of stable molecular adducts.^6,7,20^

We judged that these data were encouraging for the feasibility of our project, but not sufficiently clear-cut. We then turned our attention towards other metals. We reasoned that an increase in atomic number would reduce the Coulomb density and might limit electrostatic repulsion. Upon an extensive screening sweeping through the periodic table, we tested a variety of Group XI M(I) species. Their cations can have metal-centered LUMOs with sigmoidal symmetry that might boost overlap with the HOMO of cation **1**.

The commercial reagent (CuOTf)_2_·PhCH_3_ gave us a proof of principle for our hypothesis. We mixed **1**-OTf with 4 equiv. of this salt in dry THF at room temperature for one hour, then filtered the mixture through celite and removed volatiles. The resulting red solid was retrieved with a high mass return of 73%.

It was significantly less soluble in apolar chlorinated solvents than **1**-OTf and proved to be more prone to decomposition, with black particles forming within a few days from aerated homogeneous solutions. However, once freshly prepared, it displayed a slight downfield shift of its thiolate protons in its ^1^H NMR spectrum (0.02 ppm), as observed with lithium salts.

Furthermore, we detected the diagnostic isotopic pattern of a [Pd_3_Cu] unit through ESI-HRMS analyses in this case. Once again, the use of 10 equiv. of the copper complex provided a larger downfield shift in the proton spectrum (0.04 ppm compared to **1**-OTf). The solubility and stability appeared unchanged. The HRMS analysis showed the presence of a [Pd_3_Cu] unit, like in the previous case. These MS analyses are consistent with the formation of tetranuclear complexes.

**Fig. 6:**
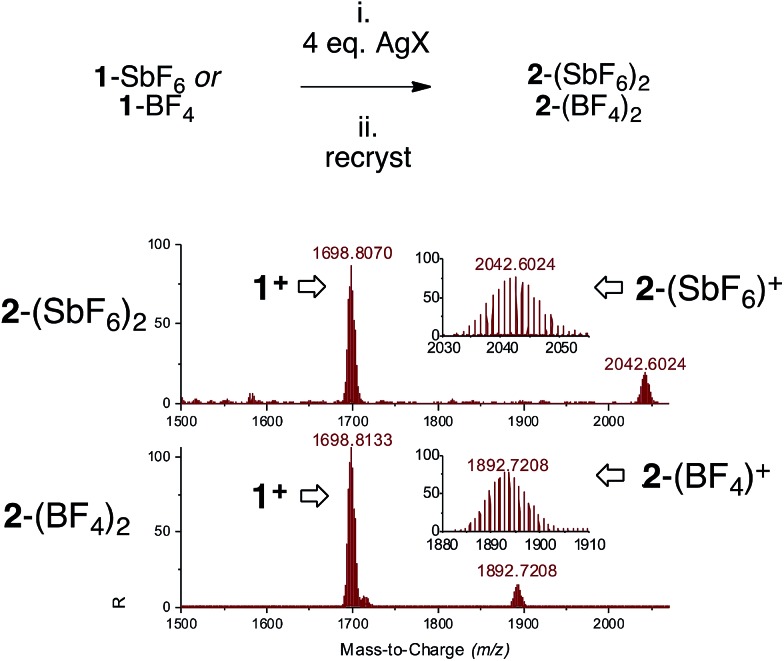
Synthesis of complexes **2** (top, on a 0.2 mmol scale, 0.01 M in degassed THF) and HRMS analyses on crystals obtained *via* vapor diffusion (bottom).

We switched to silver and mixed three [Pd_3_]^+^ complexes **1**-X with the corresponding silver salts for 1 hour in THF, then filtered traces of grey precipitates and removed volatiles. The use of AgSbF_6_, AgBF_4_ and AgCO_2_CF_3_ enabled mass recoveries of 74%, 76% and 71%, respectively. As observed with copper, the solubility of these powders in dichloromethane and chloroform proved to be lower than that of the parent **1**-X complexes. This could correlate with increased polarity due to the presence of a dicationic core. Multinuclear NMR analyses followed the same trend as presented above. In general, ^1^H and ^13^C NMR resonances showed modest shifts compared to their parent [Pd_3_]^+^ analogues. The ^31^P NMR peaks shifted downfield by 0.35–2.55 ppm. HRMS analyses showed the appearance of the isotopic fingerprint of the [Pd_3_Ag] cores. For instance, treatment of **1**-SbF_6_ with AgSbF_6_ provided a diagnostic pattern of cation [**2**-(SbF_6_)]^+^ centered at *m*/*z* = 2042, together with a highest total ion current coming from cation **1** (*m*/*z* = 1698). Analyses on **2**-BF_4_ and **2**-CO_2_CF_3_ followed suit. These results confirm the relative lability of [Pd_3_]–M interactions under MS conditions that possibly caused initial lackluster results (Fig. 6).

We then tested gold and started screening with the relatively labile (Me_2_S)AuCl complex with the aim of replacing its neutral sulfide ligand with a [Pd_3_]^+^ unit. However, the reaction showed the formation of PAr_3_AuCl and partial decomposition of **1**. We then prepared the analogue of **1**-SbF_6_ with ancillary PPh_3_ ligands and PPh_3_AuSbF_6_ to counter any scrambling of phosphorous ligands. Gratifyingly, no decomposition occurred in this case and we observed the presence of the [Pd_3_Au] unit (peak centered at *m*/*z* = 2230) by HRMS analysis. Multinuclear NMR analyses correlate with those of the other group XI metals, showing small but discrete shifts compared to the starting materials (^31^P NMR Δ = 0.8 ppm).

As mentioned above, the isolation of molecular adducts featuring cation–π interactions is usually precluded by their weak nature and by the presence of large quantities of polar (co)solvents, which are necessary to ensure ion solubility but can compete with arenes for coordination.^6,7,20^ Upon treatment of **1** with group XI metal salts, we observed a decrease in the solubility of the resulting materials in chlorinated solvents. The use of more polar solvents, such as THF, acetone and acetonitrile, solved this issue but eventually provided spectroscopic data that differed only marginally from those of **1**, as observed with lithium salts (*vide supra*). We reasoned that these differences, although strengthened by modeling data and HRMS analyses, were not sufficient to prove beyond question the formation of novel molecular entities. Clear-cut experimental evidence would have required the isolation of well-defined [Pd_3_M]^++^ complexes, which might have been possible using vapor diffusion. The technique enables a slow and gradual decrease of the concentration of the polar solvent that ensures solubility, which could in turn boost the odds of trapping the desired [Pd_3_M]^++^ complexes by gradually reversing the coordination equilibria involving the polar solvent itself.

Single crystals of [Pd_3_Ag]^++^ complex **2**-BF_4_ were obtained *via* vapor diffusion from CHCl_3_/*n*-hexane. The red powder (10 mg) was dissolved in 2 mL of chloroform in a 5 mL vial, which was then sealed in a 100 mL jar containing 15 mL of *n*-hexane. Red crystals suitable for analysis formed after five days (5 mg).

**Fig. 7:**
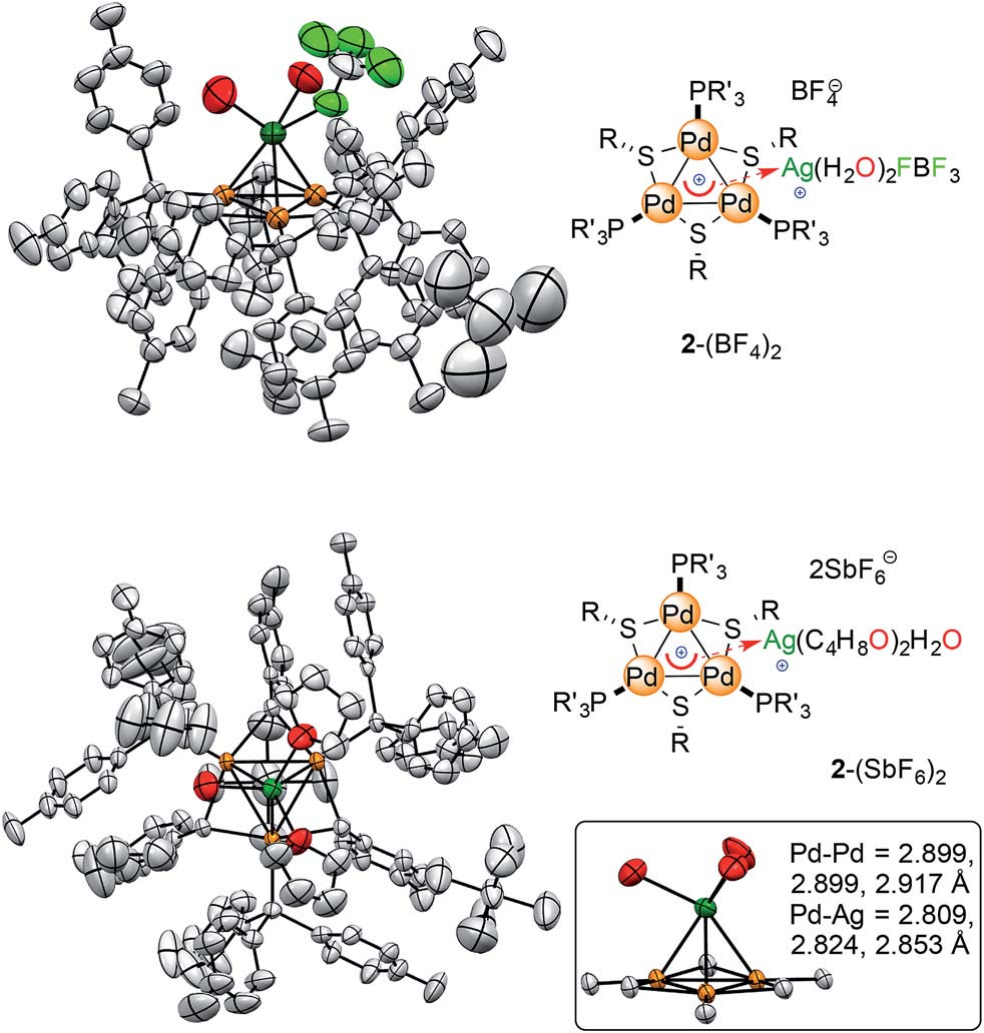
The crystal structures of the [Pd_3_Ag] complexes, with colors highlighting silver atoms (dark green) and their first coordination sphere (oxygen, fluorine and palladium in red, pale green and orange, respectively). Hydrogen atoms are omitted for clarity; ellipsoids are drawn at 50% probability, see the ESI† for extensive crystallographic details.

X-Ray diffraction analysis confirmed the presence of a [Pd_3_Ag]^++^ unit in **2**-BF_4_ and showed that the silver atom lies 2.4 Å above the center of the planar [Pd_3_]^+^ triangle (Fig. 7, top). Its first coordination sphere is filled by the oxygen atoms of two water molecules and a fluorine atom of a tetrafluoroborate anion. Silver(i) complexes are most commonly 18-electron species with a tetrahedral geometry, which is ensured by four ligands flanking the formal d-10 metal center (Fig. 1).^4^ This is essentially the same coordination pattern observed in **2**-BF_4_. The [Pd_3_]^+^ triangle remains almost equilateral upon coordination with the Pd–Pd distances being 2.909(1), 2.882(1) and 2.874(1) Å, respectively. These distances closely match that in **1** (2.8731(8) Å).^11^ The three Pd–Ag distances are 2.819(1), 2.804(1) and 2.793(1) Å, respectively. These structural features would be considered a hallmark of η^3^ coordination by a 2-electron donor ligand in complexes with regular π-aromatics. The Ag–O distances are 2.35(1) and 2.32(1) Å, respectively, and the Ag–F one is elongated (2.58(1) Å). The Ag–F distances in Ag–F–BF_3_ fragments are usually 0.2 to 0.4 Å shorter. The phosphorous and sulfur atoms of the [Pd_3_]^+^ unit remain almost coplanar with the metal triangle with the dihedrals remaining below 5°. We then repeated the approach presented above to crystallize complex **2**-SbF_6_. Crystals suitable for X-ray diffraction (4 mg) grew *via* vapor diffusion using THF and *n*-hexane (1 and 10 mL, respectively). The silver atom has no anionic species in its first coordination sphere in **2**-SbF_6_. It includes the [Pd_3_]^+^ unit, two oxygens from THF molecules (2.383(7) and 2.378(8) Å, respectively) and one from a water molecule (Ag–O distance of 2.370(7) Å). The latter fragment is engaged in hydrogen bonding with two more THF units in the crystal cell. In **2**-SbF_6_, the silver cation of a [AgL_3_]^+^ fragment completes its tetrahedral coordination with the cationic [Pd_3_]^+^ triangle. Pd–Pd (2.917(1), 2.8990(8) and 2.8988(9) Å) and Pd–Ag (2.853(1), 2.824(1) and 2.809(1) Å) distances followed the trend of those in **2**-BF_4_. The structures of complexes **2** show minimal changes in angles, distances and dihedrals compared to the corresponding complex **1**. For instance, the differences in the Pd–P and Pd–S distances remains below 0.03 Å.

Complexes **2** are the first reported [Pd_3_Ag] species.

All of the metal–metal distances in **2**-BF_4_ and **2**-SbF_6_ are significantly shorter than the sum of the van der Waals radii of palladium and silver (3.26 and 3.30 Å, respectively). This is consistent with the presence of chemical bonding between these nuclei. Both **1**-BF_4_ and **1**-SbF_6_ display a perfectly equilateral triangular kernel.^11*b*^ The [Pd_3_]^+^ cores of **2**-BF_4_ and **2**-SbF_6_ no longer present actual bond length equalization, with metal–metal distances varying by (just) 0.02 Å. The metal triangle shows the same trend as highly symmetric regular arenes, which present lower geometric symmetry upon coordination.

The literature does not present general criteria to predict the prowess of triangular metal clusters towards bonding.^21^ Reactions of [M_3_]^*n*^ complexes can deliver capped species when one of the reagents is *neutral* or *anionic*. The capping unit is often a nucleophile and the electrons of this formal ligand contribute to the bonding. The lone pairs of suitable M′ fragments could act in a similar way to organics. These reactions can thus yield tetra- and polynuclear clusters and their metal–metal bonds involve *multiple molecular orbitals*. Focusing on Group X atoms, examples of platinum triangles that allow access to [Pt_3_M′]^*n*^ complexes by far outnumber examples with palladium.^22^ This divergent behavior can be due to the lower electron density of the second-row elements compared to that of the corresponding third-row nuclei and can be confirmed by the Pd–Pd distances, which are longer than the Pd–Pt ones in isostructural nanowires.^23^ This combines with reduced relativistic effects, which increase the metallophilicity of Pt by expanding its d-type orbitals^24^ and highlights the challenges behind the present findings. Furthermore, these rationales evenly correlate with the trend of the Δ*G* values calculated for Li^+^ complexation by our [M_3_]^+^ series, which decrease linearly when replacing each Pt atom with Pd ones (*vide supra*).

Regarding the structures of **2**-BF_4_ and **2**-SbF_6_, it is worth noting that the heteroatoms that complete the first coordination sphere of palladium do not tilt in both cases and remain almost coplanar with the trinuclear metal kernel (Fig. 7 inset). This is a striking structural difference compared to the phosphinic fragments of the [Au_4_] complex that features a covalent 4c–2e bond. This difference could be consistent with a coordination-like bonding mode, in which the [Pd_3_]^+^ unit mimics a π–donor ligand, rather than a covalent one that is delocalized among the four metal nuclei. We thus thought to take advantage of the relative lability of the coordination compounds by testing the effect of water. This might indeed revert the complexation equilibria, stabilizing the aquo complexes of the bare metal cations.

This eventually proved to be the key to get a clear-cut experimental result and enabled us to discriminate between the two limit forms describing the metal–metal bonding of [Pd_3_Ag]^++^ presented in Fig. 3, namely the 3D aromatic 4c–2e bond *versus* the coordination one.

**Fig. 8:**
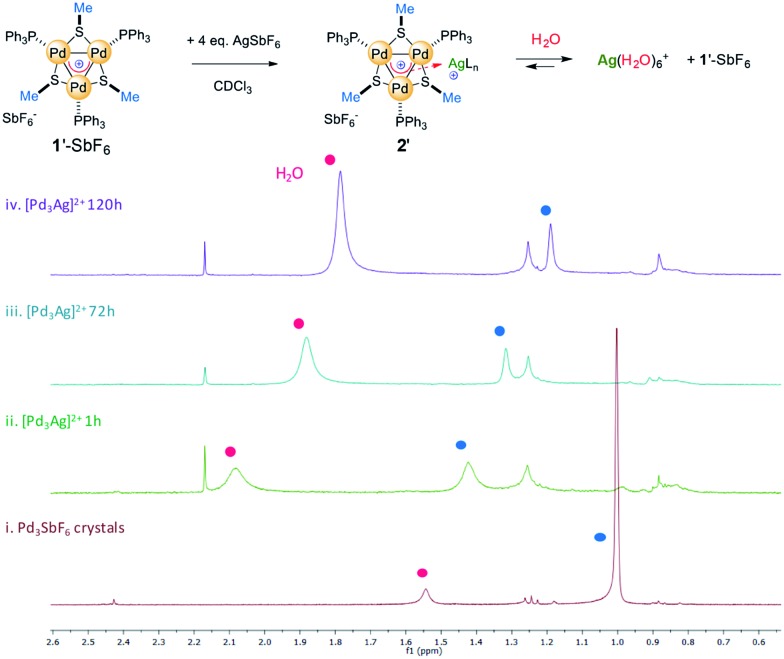
The key ^1^H NMR region for the reaction of complex **1′**-SbF_6_ with AgSbF_6_, enabling the slow diffusion of water (red circles) and highlighting the complexation equilibrium; the assignment of the methyl resonances was supported by HSQC correlation.

We prepared complex **1′**-SbF_6_, which has methyl groups on the bridging thiolates that resonate as a sharp singlet at 1.0 ppm in the ^1^H NMR spectrum (red line, Fig. 8). We dissolved **1′**-SbF_6_ in CDCl_3_, added 4 equiv. of previously dried AgSbF_6_ and put the mixture in an unsealed NMR tube. This enables water vapor to slowly diffuse through its rubber septum over time. Recording ^1^H and ^31^P spectra 1 hour upon addition (green line) provided meaningful chemical shifts of the methyl protons (Δ = 0.45 ppm) and a slightly increased resonance already due to residual water (broad band at 2.1 ppm). Upon 24 hours at RT, the shift in the methyl resonances was reduced and the water peak continued to increase. This could be consistent with a competing complexation equilibrium controlled by concentration effects. The trend is confirmed upon 72 hours with the methyl shift being reduced to 0.3 ppm (cyan line). Upon 5 days (purple line), the water resonance continues to increase, moving back to its usual shift in CDCl_3_ (1.5 ppm) and the methyl signal further reverts back to close to the value of pure complex **1′**. This trend in the shifts is similarly observed, although to a reduced extent, both with the aromatic resonances of the PPh_3_ fragments and with ^31^P ones (spectra in the ESI†).

Taken together, these experimental observations are consistent with a coordination-like bonding mode, while they are at odds with the covalent 4c–2e bond of 3D aromatics. We are so far unable to grow crystals that are suitable for recording the X-ray diffraction pattern of **2′**-SbF_6_, and we thus tried to carry out further modeling studies to strengthen these experimental findings.

**Fig. 9:**
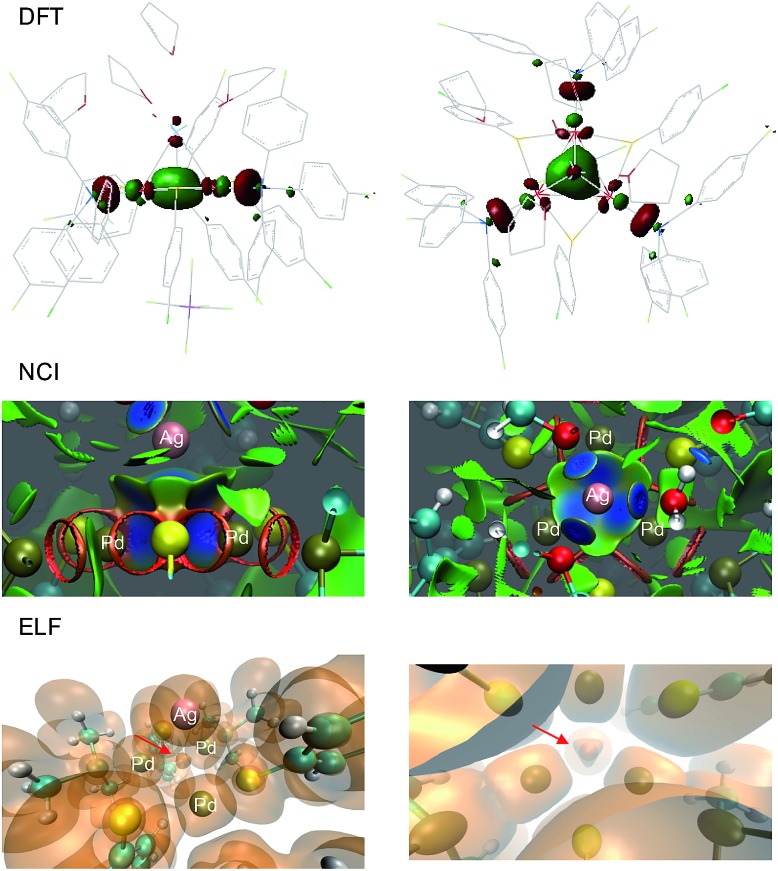
Calculated HOMO of **2**-SbF_6_, upon optimization of X-ray geometry at the M11/Def2-SVP level (top), NCI (center) and ELF topological analysis (bottom); the side view is on the left and the top view is on the right.

The solid state structure of **2**-SbF_6_ was optimized without any constraints using various DFT functionals and basis sets. At the M11/Def2-SVP level, the Pd–Pd distances were 2.921, 2.905 and 2.901 Å, respectively, and were thus within a difference of 0.01 Å to the X-ray data.^25^ The Pd–Pd distances were slightly longer when using other combinations of functionals (B3LYP, BP86, PBE0 and M06) and basis sets (lacvp(d)) but nonetheless yielded comparable results regarding orbitals, population analyses and charges. This trend is also observed using Def2-TZVP on a truncated system with PMe_3_, SMe and H_2_O fragments. The latter simplified complex was freely optimized at the MP2 and double hybrid B2PLYP level with the Def2-TZVP triple-*ζ* set for metals only. In all cases, no meaningful differences appeared on the delocalized orbitals. The calculated natural and Mulliken charges always showed limited differences to **1**. Those of the palladium nuclei slightly increased (+2–8%), as expected upon coordination of a cation. In **1**, the metal d_*x*^2^–*y*^2^_ AOs of the three palladium atoms combine to form the bonding HOMO and two almost degenerate empty MOs, the LUMO and LUMO+1 (Δ of 0.08 kcal mol^–1^). As expected for aromatics,^1^ the HOMO–LUMO gap is considerably large (36.5 kcal mol^–1^). The HOMO has sigmoidal symmetry and makes **1** a d-orbital all-metal aromatic.^11^ It is fully delocalized among the triangle in the reagent and is slightly expanded towards the silver atom in the tetranuclear complex (Fig. 9, top). Upon coordination, the energetic level of the HOMO increases by +2.1 kcal mol^–1^. The two degenerate LUMOs of **1** moved inversely, resulting in lower energies by –1.7 and –3.8 kcal mol^–1^. Taken together, these features parallel those displayed by regular arenes upon metal coordination. NCI (Non-Covalent Interaction) analysis was then performed on the X-ray structure of **2**.^26^ This method allows the visualization of weak interactions with color coding, ranging from blue, which represents the strongest, to green, highlighting the dispersion forces, and eventually red for repulsive ones (Fig. 8, center). The blue basin localized among the four metals indicates the bonding interaction between them. The volume described by this surface is closer to the Pd_3_ face than to the center of the pyramid. The complementary topological analysis ELF (Electron Localization Function) confirms that two electrons only are present inside the metal cage. Once again, this basin is closer to the Pd_3_ face than to the center of the tetrahedron (lower part of Fig. 9). These results further strengthen the DFT findings.

**Fig. 10:**
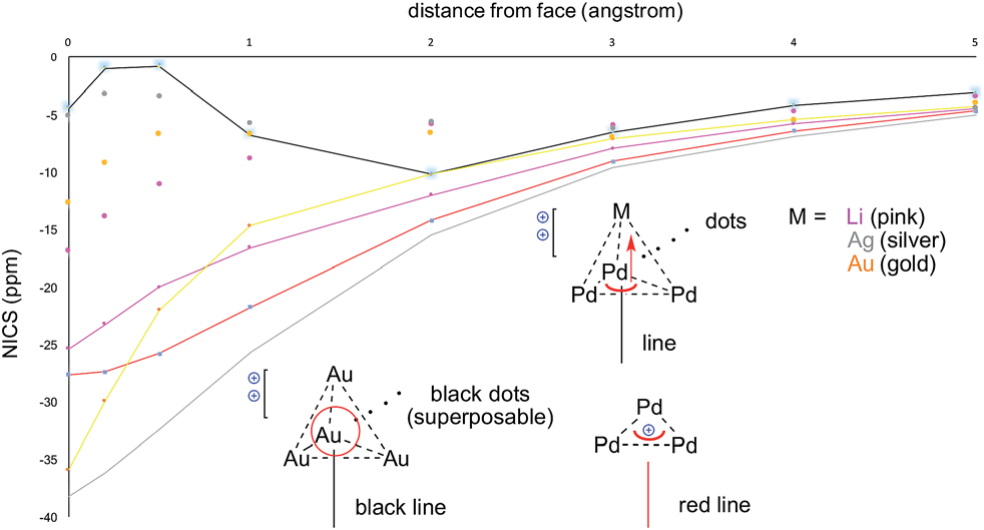
NICS calculations for [Pd_3_]^+^, [Au_4_]^++^, [Pd_3_Li]^++^, [Pd_3_Ag]^++^ and [Pd_3_Au]^++^, comparing the differences from their M3 (lines) *vs.* M2M′ (dots) faces.

The calculated HOMO of **2**-SbF_6_ tethers together a [Pd_3_]^+^ fragment and a [AgL_3_]^+^ one. The four metal nuclei of **2**-SbF_6_ describe the vertices of an almost equilateral ideal pyramid, as observed with the adamantyl dication and its gold-based analogue. This is due to the similar atomic radii of Pd and Ag. Indeed, all of the other trigonal pyramids that were modeled show either a shorter height (with Li^+^ and Cu^+^, which are smaller nuclei) or a longer one (with LAu^+^). To further confirm this hypothesis, we performed NICS analysis to compare [Pd_3_M]^++^ (M = Li, Ag and Au) and [Au_4_]^++^ (Fig. 10). This modeling tool^27^ is routinely used to assess aromaticity, which induces negative values of the calculated magnetic shifts of points in the space perpendicular to delocalized electrons. The 3D aromatic [Au_4_]^++^ complex expectedly provided negative values up to 5 Å away from the center of its cage. Modeling a series of points perpendicular to each of its Au_3_ faces provided identical values and hence superposable curves (Fig. 10, black line and superposable black dots). This correlates with both its high symmetry and the sigmoidal shape of its 4c–2e metal–metal bond. In contrast, the calculated values for [Pd_3_Li]^++^ are significantly different for the Pd_3_ face (pink line) compared to each Pd_2_Li one (pink dots). This behavior is the same for both [Pd_3_Ag]^++^ and [Pd_3_Au]^++^ complexes (silver and gold, respectively). This is consistent with delocalized bonding that remains localized on the Pd_3_ face, which occurs in the coordination complexes of regular arenes. The trend of the calculated NICS values is similarly different. It presents two flex points for [Au_4_]^++^ and none for [Pd_3_]^+^ and [Pd_3_M]^++^, which further suggests that delocalized electrons represent complementary bonding modes among these complexes.

In all of the modeled [Pd_3_M]^++^ complexes we did not find any other bonding orbital among the four metal nuclei besides the bonding HOMO. Other MOs, including the LUMO and LUMO+1, were basically unaffected compared to the parent [Pd_3_]^+^. This correlates with the minimal structural reorganization of **1** in **2**. Both the NBOs (natural bond orbitals) and AdNDP (adaptive natural density partitioning)^28^ confirmed this result, showing that each palladium atom retains four doubly occupied d-type lone pairs while silver expectedly has five (always above 1.93 occupation each). These results are at odds with cve (cve = cluster valence electron) counting, for which each metal is zero-valent.^21,22^ This framework was not designed, however, to rationalize bonding in all-metal aromatics.

Taken together, all of these features parallel those of traditional cation–aromatic bonding interactions, in which valence p_*z*_ electrons are engaged in delocalized MOs with π symmetry that can interact with Lewis acids. Taken together, these experimental and computational features let us draw these molecules using the formalism of η^*n*^ complexes.

## Conclusions

We report the synthesis of discrete tetranuclear dicationic complexes from triangular tripalladium clusters and simple metal salts under mild conditions. These [Pd_3_]^+^ complexes displayed Lewis acid properties.^11*c*^ The present study proves that cationic all-metal aromatics could be Lewis bases at the same time, with their core acting as an amphiphilic metallic surface.^29^ They represent, therefore, the bottleable complement to recently reported anionic boron clusters that present Lewis acidity.^30^ Thanks to suitably delocalized metal–metal bonds, readily available [Pd_3_]^+^ complexes can mimic regular aromatics, playing the role of a donor ligand and coordinate Lewis acidic species. This ligand-like behavior has already proven to be relatively general towards various metal nuclei. We anticipate that the introduction of all-metal aromatic cationic metal rings as a new class of donor ligand is promising for vast innovation in coordination chemistry and catalysis.

## Conflicts of interest

There are no conflicts to declare.
