# Supplementary material for: All-metal aromatic cationic palladium triangles can mimic aromatic donor ligands with Lewis acidic cations
Source: Chem Sci. 2017 Aug 29;8(11):7394–402. doi: 10.1039/c7sc03475j (PMC5672843; doi:10.1039/c7sc03475j)
Supplement: Supplementary file 1 [file SC-008-C7SC03475J-s001.pdf]

Electronic Supplementary Material (ESI) for Chemical Science.
This journal is © The Royal Society of Chemistry 2017

Electronic Supporting Information
for

All-Metal Aromatic Cationic Palladium Triangles Can Mimic Aromatic Donor Ligands towards Lewis Acidic Cations

Yanlan Wang, Anna Monfredini, Pierre-Alexandre Deyris, Florent Blanchard, Giovanni Maestri, and Max Malacria

Contents

	Page
1. General remarks	3
2. Experimental procedures	6
3. Spectroscopic data	9
4. Copies of spectra	
additional MS and NMR experiments	16
copies of HRMS, NMR, IR and UV-vis. spectra of complexes **1** and **2**	20
5. Additional modeling data	67
6. Copy of .cif files for clusters **1**-OTf, **2**-SbF_6 and **2**-BF_4	80
7. XYZ coordinates	103
8. References	181

1. General Remarks

Disulfide, phosphines and $Pd(dba)_2$ were purchased from commercial sources and used as received. Solvents were degassed by bubbling argon for at least 30 minutes prior to use. Reactions and filtrations were carried out under argon using standard Schlenk technique. ^1H NMR, ^{13}C NMR, and ^{31}P NMR spectra were recorded in acetone-d_6 at 300 K on a Bruker 500 AVANCE spectrometer fitted with a BBFO probe head at 500, 125, and 202 MHz respectively, using the solvent acetone-d_6 as internal standard (2.05 ppm for ^1H NMR and 29.84 ppm for ^{13}C NMR, respectively). For ^{31}P, H_3PO_4 was used as external standards. ^{19}F NMR spectra were recorded in acetone-d_6 at 300 K on a Bruker 300 AVANCE spectrometer fitted with a BBFO probe head at 282 MHz. Reported assignments are based on COSY, decoupling, HMBC, HSQC, NOESY and ROESY correlation experiments. The terms m, s, d, t, and q represent multiplet, singlet, doublet, triplet and quadruplet respectively, and the term br means a broad signal. Exact masses were recorded on an Agilent Q-TOF 6540 spectrometer (electrospray source). IR spectra were recorded with a Bruker Tensor 27 ATR diamant PIKE spectrometer and UV-visible spectra were recorded on a Shimadzu UV-2101 spectrophotometer.

Single crystals of **1**-OTf (CCDC 1410442), **2**-SbF_6 (CCDC 1410441) and **2**-BF_4 (CCDC 1410440) were obtained by recrystallization from THF / hexane mixture for (**1**) and (**2**-SbF_6) and $CHCl_3$ / hexane for (**2**-BF_4). A Rigaku XtaLabPro diffractometer equipped with a microfocus source (MicroMax003_Mo) and multilayer confocal mirrors (Mo Kα radiation, λ = 0.71075 Å) was used for collecting room temperature X-ray crystallographic data of (**1**). X-ray crystallographic data for compound (**2**-SbF_6) and (**2**-BF_4) were collected at 200K on a Rigaku Rapid II (IP area detector system) diffractmeter equipped with a rotating anode mm007 HF generator and Osmic mirrors (Cu Kα radiation, λ = 1.54187 Å) using ω-scans. Data were indexed, integrated and scaled using *d*TREK* for (**1**) and FS_Process for (**2**-SbF_6) and (**2**-BF_4) from the *CrystalClear*[1] software suite. They were also corrected for polarization, Lorentz and absorption effects, *REQAB* for (**1**) and *ABSCOR* for (**2**-SbF_6) and (**2**-BF_4).

The structure was solved with the ShelXT[2a] structure solution program using Direct Methods and refined with the ShelXL[2b] refinement package using Least Squares minimisation. All non-hydrogen atoms were refined with anisotropic displacement parameters and H atoms have been added geometrically and treated as riding on their parent atoms. Due to a disorder on triflate anion $CF_3SO_3^-$ in compound (**1**), its position was refined over two

orientations using FVAR variable (occupancy factor: 0.657(4)/0.343(4)). "Idealized Molecular Geometry Library"[3] was used for modelling this triflate anion, inserted with FRAG command in ShelXL. Rigid body restrains were applied along the entire connectivity set of complexes (**2**-SbF_6) and (**2**-BF_4) leading to more reasonable anisotropic displacement parameters, using standard deviation values: sigma for 1-2 distances of 0.004 and sigma for 1-3 distances of 0.004. Some large electron peaks due to solvent $CHCl_3$ molecules were found during refinement of complex (**2**-BF_4). As we failed to model them properly, the rest of the molecule was refined without the effect of the solvent molecule(s) by the PLATON SQUEEZE technique. More details including comprehensive table of crystallographic information and summary of X-ray diffraction analysis are presented in the dedicated part of this document.

Calculations were performed with Gaussian 09 at DFT level.[4] The geometries of all complexes reported herein were optimized without any symmetry constraints at the generalized gradient approximation using the Minnesota family of hybrid functionals described by Zhao and Truhlar.[5] Optimizations were carried out using Def2-svp[6] basis sets, which provided the best correlation with solid state structures for aromatic Pd_3 clusters obtained from isothioureas. Other families of functionals (B3LYP, PBE0 and BP86) provided comparable results regarding orbitals and their population but lower correlation with solid state geometries, namely an overestimation of M-M distances (around +0.05-0.1 Angstoms). LACVP(d),[7] and RSC97[8] basis sets were also tested; they provided the same results describing delocalized molecular orbitals among metal centers but a slightly lower correlation with solid state structures found by X-ray. Single point calculations were perfomerd at the MP2 level[9] and using double hybrid B2PLYP functional[10] to exclude that different Hartree-Fock contribution could provide meaningful differences in calculated molecular orbitals. Harmonic frequencies were calculated at the M06/Def2-svp level to characterize optimized structures as stationary points by the absence of imaginary frequencies in their Hessian matrixes. Quadrupolar moments were calculated at the M06/Def2-svp level. The same method was used to model $[M_3]^+$-Li^+ adducts and lithium complexation by a water or a benzene molecule. The best correlation with X-Ray structure of complexes **2** was obtained at the M11/Def2-svp level. Other families of functionals (B3LYP, PBE0 and BP86) provided comparable results regarding orbitals and their population but lower correlation with solid state geometries, namely an overestimation of M-M and M-M' distances (around +0.03-0.06 Angstoms). Free optimization of tetranuclear complexes using Def2-TZVP basis set for metal atoms (Li, Pd, Ag and/or Au) provided minimal differences in structures (within 0.004

Angstroms) and no differences either in bonding metal-metal bonds. Similarly, the population of lone pairs (for Pd, Ag and Au) did not change significantly compared to double-ζ functionals. This is consistent with previous results from our group of all-metal aromatic M_3^+ complexes (see references 11 of the main article) and is likely due to the spatial contraction of d-type atomic orbitals of late transition metals compared to early ones, which often prevents the population of multiple metal-metal bonds.[11] Gaussian09 was used to obtain both canonical molecular orbitals and natural ones (NBO). The latter were used for AdNDP analysis through its dedicated software.[12]

2. Experimental procedures

Synthesis of Triangular Tripalladium Clusters 1-X (X = OTf, BF_4, SbF_6, CF3CO_2)

The synthesis of complex **1-X** has been carried out according to the following procedure. $Pd(dba)_2$ (0.2 mmol, 115 mg, 1 equiv.) was added to a 50 mL Schlenk. The vessel underwent at least three vacuum/Ar cycles. 20 mL of freshly degassed $CHCl_3$ were then syringed under Ar. The phosphine (0.2 mmol, 1 equiv.) and the bis(4-chlorophenyl) disulfide (0.1 mmol, 0.5 equiv.) were immediately added to the mixture under Ar. The resulting solution was kept under stirring at r.t. for 2 hours, the silver salt (0.067 mmol, 0.33 equiv.) was then added under Ar and the solution was put in the dark. Stirring was maintained for 1 hour and the mixture was then filtered through a short pad of celite under Ar to remove traces of black metals. The solvent was removed under vacuum to leave a deep red solid that was purified by $CHCl_3$/hexane washings (1/30 v/v, 3 x 30 mL). Evaporation of solvents under vacuum afforded NMR pure clusters as orange/red solids. Complexes were further purified by recrystallization by vapor diffusion using THF/hexane. Through this method, crystals of **1-**OTf suitable for X-Ray diffractions were obtained too. Complexes **1**-X were characterized by ^{1}H, ^{13}C, ^{31}P and ^{19}F NMR, UV-vis., IR and ESI^+-Tof HRMS.

Reaction of 1-SbF_6 with $AgSbF_6$

$AgSbF_6$ (0.0444 mmol, 4 equiv.) was added to a solution of compound **1-SbF_6** (0.0111 mmol, 1 equiv.) in 5 mL of $CHCl_3$ under Ar. The deep red solution was put in the dark. Stirring was maintained for 1 hour and the mixture was then filtered through a short pad of Celite under Ar. The solvent was removed under vacuum to leave a deep red solid. Then the compound was purified by recrystallization. Crystals of **2-SbF_6** suitable for X-Ray diffractions were obtained by vapor diffusion using THF/hexane. Crystals of **2-SbF_6** were analyzed by ^{1}H, ^{13}C, ^{31}P and ^{19}F NMR, UV-vis., IR and ESI^+-Tof HRMS.

Reaction of 1-BF_4 with $AgBF_4$

$AgBF_4$ (0.0444 mmol, 4 equiv.) was added to a solution of compound **1-BF_4** (0.0111 mmol, 1 equiv.) in 5 mL of $CHCl_3$ under Ar. The deep red solution was put in the dark. Stirring was maintained for 1 hour and the mixture was then filtered through a short pad of Celite under Ar. The solvent was removed under vacuum to leave a deep red solid. Then the compound was purified by recrystallization. Crystals of **2-BF_4** suitable for X-Ray diffractions were

obtained by vapor diffusion using $CHCl_3$/hexane. Crystals of **2-BF_4** were analyzed by ^{1}H, ^{13}C, ^{31}P and ^{19}F NMR, UV-vis., IR and ESI^{+}-Tof HRMS.

Reaction of 1-CF_3CO_2 with $AgCF_3CO_2$

CF_3CO_2Ag (0.0444 mmol, 4 equiv.) was added to a solution of compound **1-CF_3CO_2** (0.0111 mmol, 1 equiv.) in 5 mL of $CHCl_3$ under Ar. The deep red solution was put in the dark. Stirring was maintained for 1 hour and the mixture was then filtered through a short pad of Celite under Ar. The solvent was removed under vacuum to leave a deep red solid. Then the compound was purified by recrystallization by vapor diffusion using THF/hexane. Crystals were then analyzed by by ^{1}H, ^{13}C, ^{31}P and ^{19}F NMR, UV-vis., IR and ESI^{+}-Tof HRMS.

Reaction of 1-SbF_6-PPh_3 with PPh_3AuCl

PPh_3AuCl (0.0667 mmol, 1 equiv.) and $AgSbF_6$ (0.0667 mmol, 1 equiv.) were added to freshly degassed $CHCl_3$ (10 mL) and the mixture stirred for 1 hour in the dark. After filtration through Celite to remove AgCl, the solvent was evaporated to dryness under vacuum, the residue was dissolved in THF (10 mL) and the resulting solution was canulated to a solution of **1-SbF_6-PPh_3** (0.0667 mmol, 1 equiv.) in 10 mL of THF under Ar. The resulting solution was put in the dark. Stirring was maintained for 1 hour and the mixture was then filtered through a short pad of Celite under Ar. Evaporation of solvents under vacuum afforded an orange solid. The compound was purified by recrystallization by vapor diffusion using THF/hexane, although the quality of crystals did not allow to perform RX. Crystals were then analyzed by ^{1}H, ^{13}C, ^{31}P and ^{19}F NMR, UV-vis., IR and ESI^{+}-Tof HRMS.

Reaction of 1-OTf with $(CF_3SO_3Cu)_2$•$PhCH_3$

$(CF_3SO_3Cu)_2$•$PhCH_3$ (0.0222 mmol, 4 equiv.) was added to a solution of compound **1-OTf** (0.0111 mmol, 1 equiv.) in 5 mL of $CHCl_3$ under Ar. The deep red solution was put in the dark. Stirring was maintained for 1 hour and the mixture was then filtered through a short pad of Celite under Ar. The solvent was removed under vacuum to leave a deep red solid. The compound was purified by recrystallization by vapor diffusion using THF/hexane, although the quality of crystals did not allow to perform RX. Crystals were then analyzed by ^{1}H, ^{13}C, ^{31}P and ^{19}F NMR, UV-vis., IR and ESI^{+}-Tof HRMS.

Reaction of 1-OTf with CF_3SO_3Li

Complex **1-OTf** (0.0667 mmol, 1 equiv.) was solubilized in distilled THF (20 mL) and CF_3SO_3Li (0.6667 mmol, 10 equiv.) was then added under Ar. The solution was put in the dark. Stirring was maintained for 1 hour and the mixture was then filtered through a short pad of Celite under Ar. Evaporation of solvents under vacuum afforded a deep orange solid. The sample (**1** + excess CF_3SO_3Li) was analyzed by ^{1}H, ^{13}C, ^{31}P and ^{19}F NMR, UV-vis., IR and ESI^{+}-Tof HRMS.

Reaction of 1-BF4 with $LiBF_4$

Complex **1-BF$_4$** (0.0667 mmol, 1 equiv.) was solubilized in distilled THF (20 mL) and $LiBF_4$ (0.6667 mmol, 10 equiv.) was then added under Ar. The solution was put in the dark. Stirring was maintained for 1 hour and the mixture was then filtered through a short pad of Celite under Ar. The solvent was removed under vacuum to leave a deep red solid. The sample (**1** + excess $LiBF_4$) was analyzed by ^{1}H, ^{13}C, ^{31}P and ^{19}F NMR, UV-vis., IR, and ESI^{+}-Tof HRMS.

3. Spectroscopic data of $[Pd_3]^+$ complexes 1-X

Cluster **1**-OTf

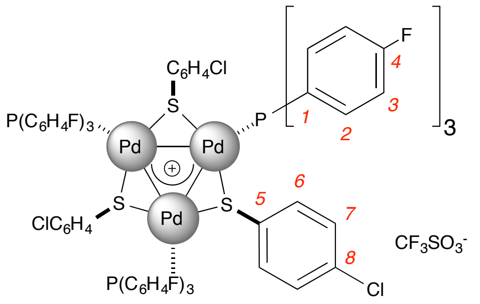

Yield = 91%; HRMS calculated for $C_{72}H_{48}Cl_3F_9P_3Pd_3S_3^+$ 1698.8170, found 1698.8167. ^1H NMR (500 MHz, CD_3COCD_3): δ 7.29 (br s, 18H, H2), 7.09 (t, J = 8.8 Hz, 18H, H3), 6.88 (d, J = 8.5 Hz, 6H, H7), 6.51 (d, J = 8.5 Hz, 6H, H6); 2.84 (s, H_2O). ^{13}C NMR (125 MHz, CD_3COCD_3): δ 165.2 (d, J = 252.0 Hz, C4), 137.1 (s, C2), 136.9 (br s, C6), 135.7 (C8), 134.8 (C5), 129.6 (C7), 128.0 (br s, C1), 116.8 (d, J = 22.0 Hz, C3). ^{31}P NMR (202 MHz, CD_3COCD_3): δ 13.64 (s, $P(C_6H_4F)_3$). ^{19}F NMR (282 MHz, CD_3COCD_3): δ -109.45 (s, $P(C_6H_4F)_3$), -78.78 (s, $CF_3SO_3^-$). IR (cm^{-1}): ν 3071, 2926, 2853, 1589, 1497, 1470, 1394, 1271, 1238, 1162, 1093, 1030, 1011, 827. UV-vis.: c = 8.5 × 10^{-6} mol/L in $CHCl_3$, λ_{max} = 243 nm, ε_{max} = 9.5 × 10^4 M^{-1} cm^{-1}.

Cluster **1**-SbF_6

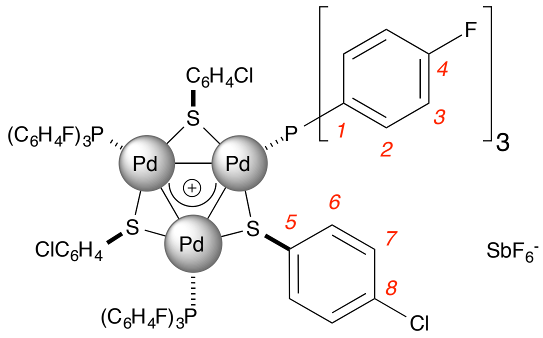

Yield = 90%; HRMS calculated for $C_{72}H_{48}P_3Pd_3S_3Cl_3F_9^+$ 1698.8170, found 1698.8122. ^1H NMR (300 MHz, CD_3COCD_3): δ 7.30 (br s, 18H, H2), 7.09 (t, J = 8.6 Hz, 18H, H3), 6.87 (d, J = 8.4 Hz, 6H, H7), 6.48 (d, J = 8.1 Hz, 6H, H6); 2.80 (s, H_2O). ^{13}C NMR (125 MHz, CD_3COCD_3): δ 165.2 (d, J = 251.9 Hz, C4), 137.0 (br s, C2), 136.9 (s, C6), 135.7 (C8), 134.8 (C5), 129.5 (C7), 128.0 (br s, C1), 116.9 (d, J = 21.9 Hz, C3). ^{31}P NMR (202 MHz,

$CD_3COCD_{3,}$): δ 12.66 (s, $P(C_6H_4F)_3$). ^{19}F NMR (282 MHz, CD_3COCD_3): δ-109.38 (s, $P(C_6H_4F)_3$). IR (cm^{-1}): ν 3069, 2955, 2924, 2855, 1587, 1493, 1470, 1392, 1236, 1158, 1092, 1011, 940, 824, 814, 744. UV-vis: c = 1.59 × 10^{-5} mol/L in $CHCl_3$, λ_{max} = 244 nm, ε_{max} = 0.44 × 10^5 M^{-1} cm^{-1}.

Cluster **1**-BF_4

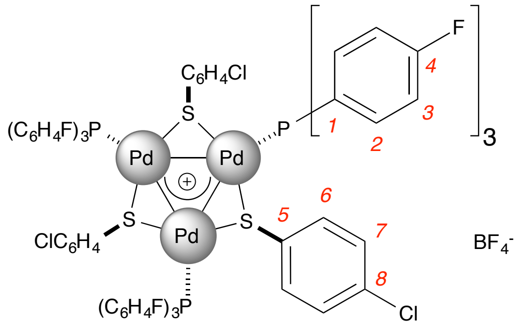

Yield = 85%; HRMS calculated for $C_{72}H_{48}Cl_3F_9P_3Pd_3S_3^+$ 1698.8170, found 1698.8167. ^1H NMR (500 MHz, CD_3COCD_3): δ 7.29 (br s, 18H, H2), 7.09 (t, *J* = 8.7 Hz, 18H, H3), 6.88 (d, *J* = 8.5 Hz, 6H, H7), 6.50 (d, *J* = 8.5 Hz, 6H, H6); 2.83 (s, H_2O). ^{13}C NMR (75 MHz, CD_3COCD_3): δ 165.2 (d, *J* = 251.9 Hz, C4), 137.1 (br s, C2), 136.9 (s, C6), 135.6 (C8), 134.8 (C5), 129.6 (C7), 128.0 (br s, C1), 116.9 (d, *J* = 22.2 Hz, C3). ^{31}P NMR (202 MHz, CD_3COCD_3): δ 13.60 (s, $P(C_6H_4F)_3$). ^{19}F NMR (282 MHz, CD_3COCD_3): δ -109.39 (s, $P(C_6H_4F)_3$), -151.66 (s, BF_4^-). IR (cm^{-1}): ν 3068, 2925, 2853, 1711, 1588, 1494, 1472, 1394, 1234, 1161, 1089, 1050, 1010, 825. UV-vis.: c = 1 × 10^{-5} mol/L in $CHCl_3$, λ_{max} = 244 nm, ε_{max} = 9.3 × 10^4 M^{-1} cm^{-1}.

Cluster **1**-CF_3CO_2

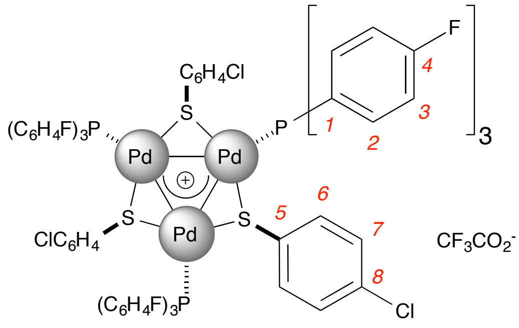

Yield = 86%; HRMS calculated for $C_{72}H_{48}Cl_3F_9P_3Pd_3S_3^+$ 1698.8170, found 1698.8132. ^1H NMR (500 MHz, CD_3COCD_3): δ 7.32 (br s, 18H, H2), 7.07 (br s, 18H, H3), 6.88 (d, J = 7.1 Hz, 6H, H7), 6.49 (br s, 6H, H6); 2.80 (s, H_2O), 0.88, 1.30 (hexane). ^{13}C NMR (125 MHz, CD_3COCD_3): δ 165.2 (d, J = 251.9 Hz, C4), 137.0 (br s, C2), 136.9 (s, C6), 135.7 (C8), 134.8 (C5), 129.5 (C7), 128.2 (br s, C1), 116.8 (d, J = 22.1 Hz, C3). ^{31}P NMR (202 MHz, CD_3COCD_3,): δ 13.97 (s, $P(C_6H_4F)_3$). ^{19}F NMR (282 MHz, CD_3COCD_3): δ -109.50 (s, $P(C_6H_4F)_3$), -73.91 (s, $CF_3CO_2^-$). IR (cm^{-1}): ν 3067, 2954, 2922, 2853, 1686, 1667, 1586, 1495, 1470, 1394, 1232, 1154, 1088, 1010, 811, 751, 705. UV-vis.: c = 5 × 10^{-6} mol/L in $CHCl_3$, λ_{max} = 242 nm, ε_{max} = 7.2 × 10^4 M^{-1} cm^{-1}.

Cluster **1**-SbF_6 with PPh_3 as ligand

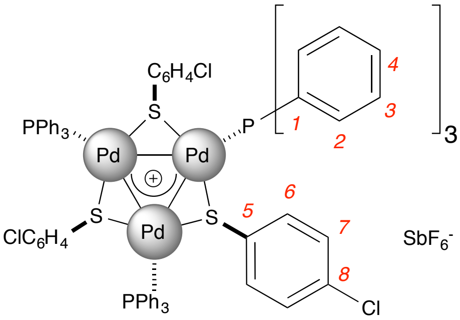

Yield = 92%; HRMS calculated for $C_{72}H_{57}Cl_3P_3Pd_3S_3^+$ 1536.8901, found 1536.8946. ^1H NMR (500 MHz, CD_3COCD_3): δ 7.43 (br s, 9H, H4), 7.29 (br s, 18H, H3), 7.24 (br s, 18H, H2), 6.76 (d, J = 7.1 Hz, 6H, H7), 6.35 (d, J = 7.1 Hz, 6H, H6); 2.80 (s, H_2O). ^{13}C NMR (75 MHz, CD_3COCD_3): δ 136.9 (s, C6), 136.0 (C8), 134.5 (br s, C3), 134.2 (C5), 131.8 (br s, C4), 129.5 (br, C1+C2), 129.2 (C7). ^{31}P NMR (202 MHz, CD_3COCD_3): δ 16.15 (s, PPh_3). IR (cm^{-1}): ν 3056, 2922, 2857, 1700, 1588, 1572, 1479, 1470, 1435, 1388, 1216, 1185, 1094, 1010, 823, 743, 691. UV-vis.: c =1 × 10^{-5} mol/L in $CHCl_3$, λ_{max} = 246 nm, ε_{max} = 6.2 × 10^4 M^{-1} cm^{-1}.

Complex **2**-SbF_6

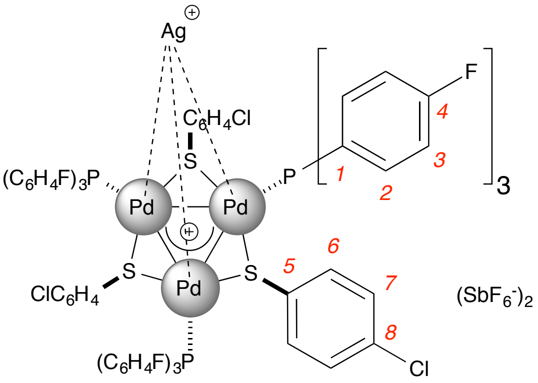

Mass recover = 76%; recrystallization yield = 48%; HRMS calculated for $C_{72}H_{48}Cl_3F_9P_3Pd_3S_3AgSbF_6^+$ 2042.6163, found 2042.6026. ^1H NMR (500 MHz, CD_3COCD_3): δ 7.30 (br s, 18H, H2), 7.09 (t, J = 8.7 Hz, 18H, H3), 6.88 (d, J = 8.4 Hz, 6H, H7), 6.50 (d, J = 8.4 Hz, 6H, H6); 1.78, 3.63 (THF). ^{13}C NMR (75 MHz, CD_3COCD_3): δ 165.3 (d, J = 252.3 Hz, C4), 137.0 (br s, C2), 136.8 (s, C6), 135.2 (C8), 134.7 (C5), 129.8 (C7), 127.2-127.8 (br, C1), 116.9 (d, J = 21.4 Hz, C3). ^{31}P NMR (202 MHz, CD_3COCD_3,): δ 13.92 (s, $P(C_6H_4F)_3$). ^{19}F NMR (282 MHz, CD_3COCD_3): δ -108.47 (s, $P(C_6H_4F)_3$), -109.65 (s, SbF_6^-). IR (cm^{-1}): ν 3068, 2922, 2853, 1724, 1700, 1586, 1493, 1470, 1436, 1394, 1302, 1232, 1161, 1091, 1011, 823, 747, 707, 691. UV-vis.: c = 5 × 10^{-6} mol/L in $CHCl_3$, λ_{max} = 242 nm, ε_{max} = 7.8 × 10^4 M^{-1} cm^{-1}.

Complex **2**-BF_4

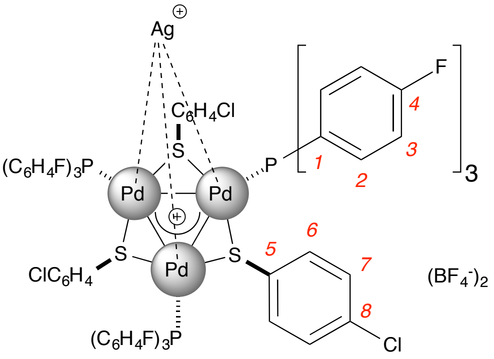

Mass recover = 73%; recrystallization yield = 51%; HRMS calculated for $C_{72}H_{48}Cl_3F_9P_3Pd_3S_3AgBF_4^+$ 1891.7261, found 1891.7207. ^1H NMR (500 MHz, CD_3COCD_3): δ 7.28 (br s, 18H, H2), 7.10 (t, J = 8.0 Hz, 18H, H3), 6.94 (d, J = 7.4 Hz, 6H, H7), 6.61 (d, J = 7.3 Hz, 6H, H6); 2.82 (s, H_2O). ^{13}C NMR (125 MHz, CD_3COCD_3): δ 165.3 (d, J = 252.1 Hz, C4), 137.2 (br s, C2), 137.0 (s, C6), 135.2 (C8), 134.4 (C5), 129.9 (C7), 127.5 (br s, C1),

116.9 (d, J = 20.7 Hz, C3). ^{31}P NMR (202 MHz, CD_3COCD_3,): δ 15.35 (s, $P(C_6H_4F)_3$). ^{19}F NMR (282 MHz, CD_3COCD_3): δ -109.15 (s, $P(C_6H_4F)_3$), -151.93 (s, BF_4^-). IR (cm^{-1}): ν 2955, 2926, 2857, 1657, 1587, 1498, 1468, 1392, 1232, 1160, 1092, 1011, 826. UV-vis.: c = 5 × 10^{-6} mol/L in $CHCl_3$, λ_{max} = 245 nm, ε_{max} = 9.4 × 10^4 M^{-1} cm^{-1}.

Complex **2**-CF_3CO_2

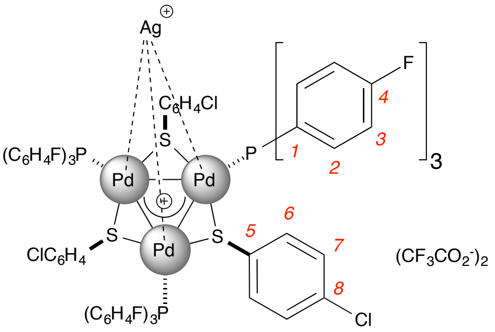

Mass recover = 71%; recrystallization yield = 50%; HRMS calculated for $C_{72}H_{48}Cl_3F_9P_3Pd_3S_3CF_3CO_2Ag^+$ 1918.7072, found 1918.7033. ^{1}H NMR (500 MHz, CD_3COCD_3): δ 7.37 (br s, 18H, H2), 6.99 (br, 18H, H3), 6.87 (d, J = 7.4 Hz, 6H, H7), 6.35 (br, 6H, H6); 2.82 (s, H_2O). ^{13}C NMR (75 MHz, CD_3COCD_3): δ 165.2 (d, J = 252.0 Hz, C4), 136.9-137.2 (br, C2), 137.0 (C6), 135.6 (C8), 134.9 (C5), 129.7 (C7), 127.8-128.5 (br, C1), 116.8 (ddd, J = 22.8, 7.2, 4.0 Hz, C3). ^{31}P NMR (202 MHz, CD_3COCD_3): δ 14.32 (s, $P(C_6H_4F)_3$). ^{19}F NMR (282 MHz, CD_3COCD_3): δ -109.50 (s, $P(C_6H_4F)_3$), -73.69 (s, $CF_3CO_2^-$). IR (cm^{-1}): ν 3069, 2926, 2854, 1899, 1664, 1586, 1495, 1470, 1394, 1237, 1195, 1160, 1089, 1010, 814, 798, 721. UV-vis.: c = 5 × 10^{-6} mol/L in $CHCl_3$, λ_{max} = 242 nm, ε_{max} = 6.2 × 10^4 M^{-1} cm^{-1}.

Cluster **2**-SbF_6-Au

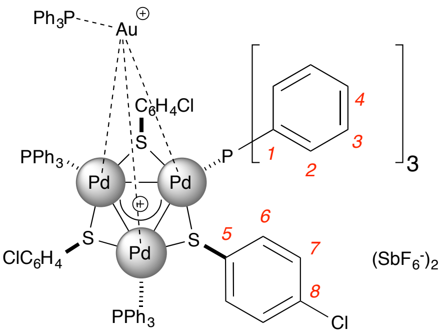

Mass recover = 72%; recrystallization yield = 56%; HRMS calculated for $C_{90}H_{72}Cl_3F_6P_4Pd_3S_3SbAu^+$ 2230.8547, found 2230.8426. ^1H NMR (500 MHz, CD_3COCD_3): δ 7.66-7.73 (br, 15H, Ph/$AuPPh_3^+$), 7.43 (br s, 9H, H4), 7.29 (br s, 18H, H3), 7.22 (br s, 18H, H2), 6.76 (d, *J* = 8.4 Hz, 6H, H7), 6.36 (d, *J* = 8.4 Hz, 6H, H6); 2.79 (s, H_2O). ^{13}C NMR (75 MHz, CD_3COCD_3): *δ* 136.9 (s, C6), 136.0 (C8), 135.2 (Ph/$AuPPh_3^+$), 134.4 (br s, C3), 134.2 (C5), 133.4 (br s, Ph/$AuPPh_3^+$), 131.8 (br s, C4), 130.7 (Ph/$AuPPh_3^+$), 129.4 (br, C1+C2), 129.2 (C7). ^{31}P NMR (202 MHz, CD_3COCD_3,): *δ* 15.81 (s, PPh_3), 44.33 (s, $AuPPh_3^+$). IR (cm^{-1}): ν 3058, 2923, 2853, 1572, 1479, 1470, 1435, 1388, 1308, 1216, 1185, 1095, 1070, 1010, 820, 744, 691. UV-vis.: c = 1 × 10^{-5} mol/L in $CHCl_3$, λ_{max} = 244 nm, ε_{max} = 5.2 × 10^4 M^{-1} cm^{-1}.

Cluster **2**-OTf-Cu

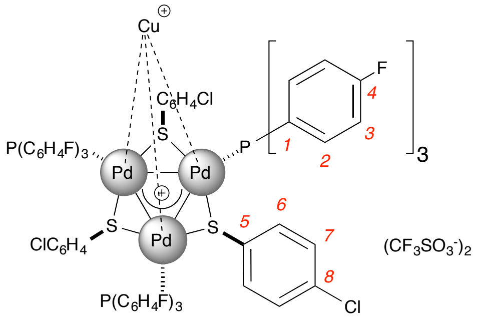

Mass recover = 75%; recrystallization yield = 46%; HRMS calculated for $C_{72}H_{48}Cl_3F_9P_3Pd_3S_3CF_3SO_3Cu^+$ 1910.6981, found 1910.6886. ^1H NMR (500 MHz, CD_3COCD_3): *δ* 7.30 (br s, 18H, H2), 7.09 (t, *J* = 8.7 Hz, 18H, H3), 6.89 (d, *J* = 8.4 Hz, 6H, H7), 6.51 (d, *J* = 8.4 Hz, 6H, H6); 2.91 (s, H_2O). ^{13}C NMR (125 MHz, CD_3COCD_3): *δ* 165.2 (d, *J* = 251.9 Hz, C4), 137.1 (br s, C2), 137.0 (dq, *J* = 9.3, 5.2 Hz, C6), 135.6 (C8), 134.7 (C5), 129.6 (C7), 128.1 (br s, C1), 116.8 (dq, *J* = 22.8, 4.0 Hz, C3). ^{31}P NMR (202 MHz, CD_3COCD_3): *δ* 13.67 (s, $P(C_6H_4F)_3$). ^{19}F NMR (282 MHz, CD_3COCD_3): *δ* -109.47 (s, $P(C_6H_4F)_3$), -78.38 (s, $CF_3SO_3^-$). IR (cm^{-1}): ν 3100, 3070, 2925, 2845, 1699, 1589, 1497, 1470, 1395, 1265, 1237, 1161, 1093, 1030, 1011, 827. UV-vis.: c = 7.5 × 10^{-6} mol/L in $CHCl_3$, λ_{max} = 244 nm, ε_{max} = 8.7 × 10^4 M^{-1} cm^{-1}.

Sample of **1**-OTf + LiOTf

HRMS calculated for $C_{72}H_{48}Cl_3F_9P_3Pd_3S_3CF_3SO_3Li^+$ 1792.8366, found (without CF_3SO_3Li) 1698.8167 (corresponding to cation 1). ^1H NMR (500 MHz, CD_3COCD_3): *δ* 7.29 (br s, 18H,

H2), 7.08 (t, *J* = 8.6 Hz, 18H, H3), 6.89 (d, *J* = 8.1 Hz, 6H, H7), 6.53 (d, *J* = 8.1 Hz, 6H, H6); 3.11 (s, H_2O), 1.79, 3.63 (m, THF). ^{13}C NMR (125 MHz, CD_3COCD_3): δ 165.1 (d, *J* = 251.9 Hz, C4), 137.2 (br s, C2), 136.9 (dt, *J* = 9.9, 4.9 Hz, C6), 135.5 (C8), 134.7 (C5), 129.6 (C7), 128.1 (br s, C1), 121.8 (q, *J* = 320.0 Hz, C/$CF_3SO_3^-$), 116.8 (dq, *J* = 22.1, 3.8 Hz, C3); 26.15, 68.10 (THF). ^{31}P NMR (202 MHz, CD_3COCD_3,): δ 13.72 (s, $P(C_6H_4F)_3$). ^{19}F NMR (282 MHz, CD_3COCD_3): δ -109.50 (s, $P(C_6H_4F)_3$), -79.09 (s, ($CF_3SO_3^-$). IR (cm^{-1}): ν 3523, 3489, 2955, 2926, 2857, 1647, 1590, 1497, 1290, 1268, 1238, 1194, 1165, 1094, 1060, 1010, 827, 678. UV-vis.: c = 6.5 × 10^{-6} mol/L in $CHCl_3$, λ_{max} = 245 nm, ε_{max} = 8.7 × 10^4 M^{-1} cm^{-1}.

Sample of **1-**BF_4 + $LiBF_4$

HRMS calculated for $C_{72}H_{48}Cl_3F_9P_3Pd_3S_3LiBF_4^+$ 1792.8366, found (without $LiBF_4$) 1698.8187 (corresponding to cation 1). ^{1}H NMR (500 MHz, CD_3COCD_3): δ 7.29 (br s, 18H, H2), 7.09 (t, *J* = 8.5 Hz, 18H, H3), 6.90 (d, *J* = 8.1 Hz, 6H, H7), 6.54 (d, *J* = 8.0 Hz, 6H, H6); 3.09 (s, H_2O), 0.86, 1.29 (hexane). ^{13}C NMR (75 MHz, CD_3COCD_3): δ 165.2 (d, *J* = 251.6 Hz, C4), 137.2 (br s, C2), 136.9 (m, C6), 135.5 (C8), 134.7 (C5), 129.6 (C7), 128.1 (br s, C1), 116.8 (ddd, *J* = 21.7, 7.3, 4.2 Hz, C3). ^{31}P NMR (202 MHz, CD_3COCD_3,): δ 13.73 (s, $P(C_6H_4F)_3$). ^{19}F NMR (282 MHz, CD_3COCD_3): δ -109.56 (s, $P(C_6H_4F)_3$), -153.29 (s, BF_4^-). IR (cm^{-1}): ν 3610, 3450, 3013, 2972, 2941, 1737,1639, 1589, 1494, 1438, 1365, 1228, 1216, 1204, 1162, 1093, 1030, 1009, 824. UV-vis.: c = 1 × 10^{-5} mol/L in $CHCl_3$, λ_{max} = 243 nm, ε_{max} = 5.5 × 10^4 M^{-1} cm^{-1}.

4. Copies of spectra

Additional experiments confirming dynamic complexation equilibria in solution

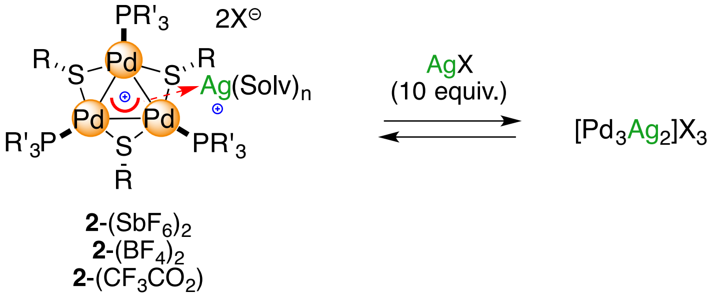

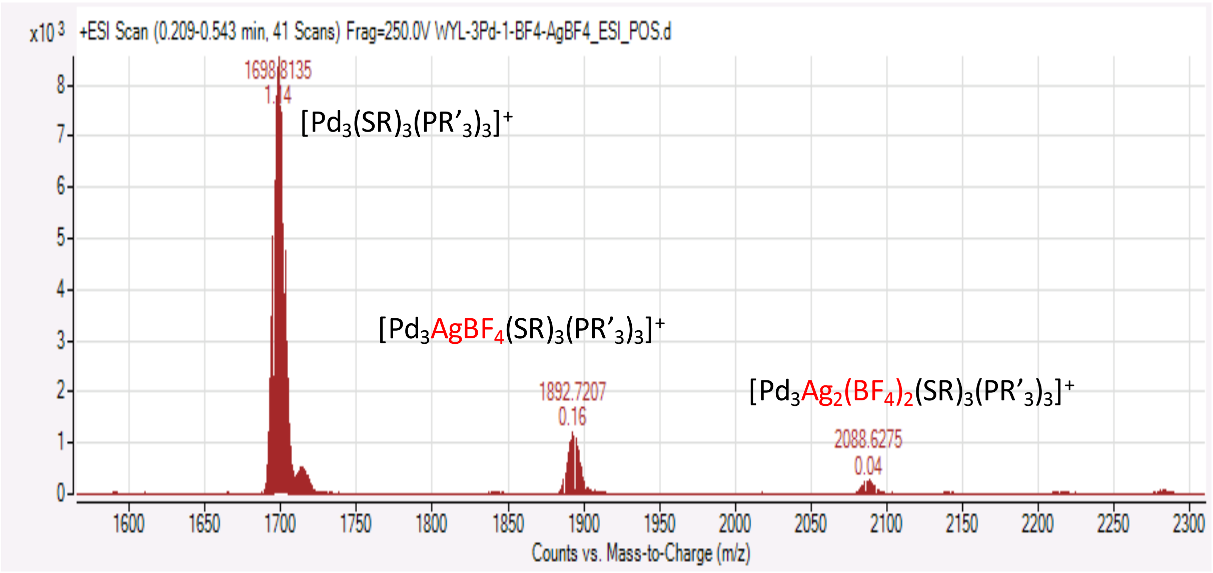

Figure S1. HRMS spectra of the reaction of Pd_3Ag^{++} with excess $AgBF_4$, showing the appeareance of the pentanuclear species.

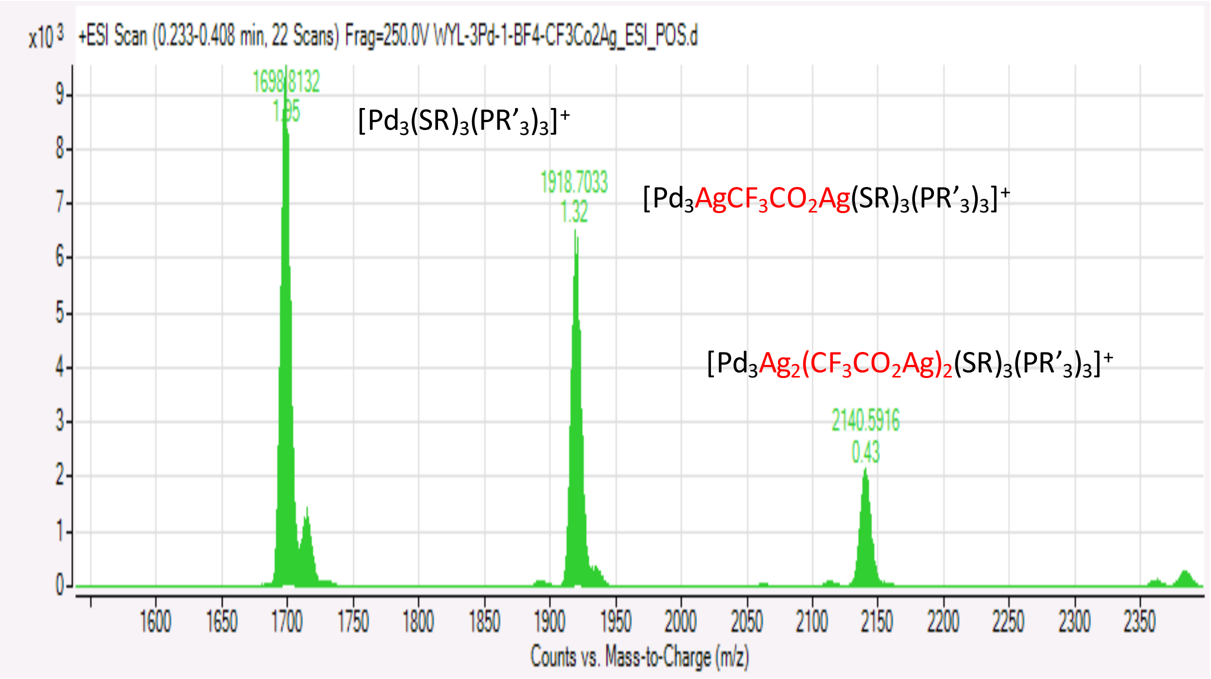

Figure S2. HRMS spectra of the reaction of Pd_3Ag^{++} with excess Ag trifluoroacetate, showing the appeareance of pentanuclear species.

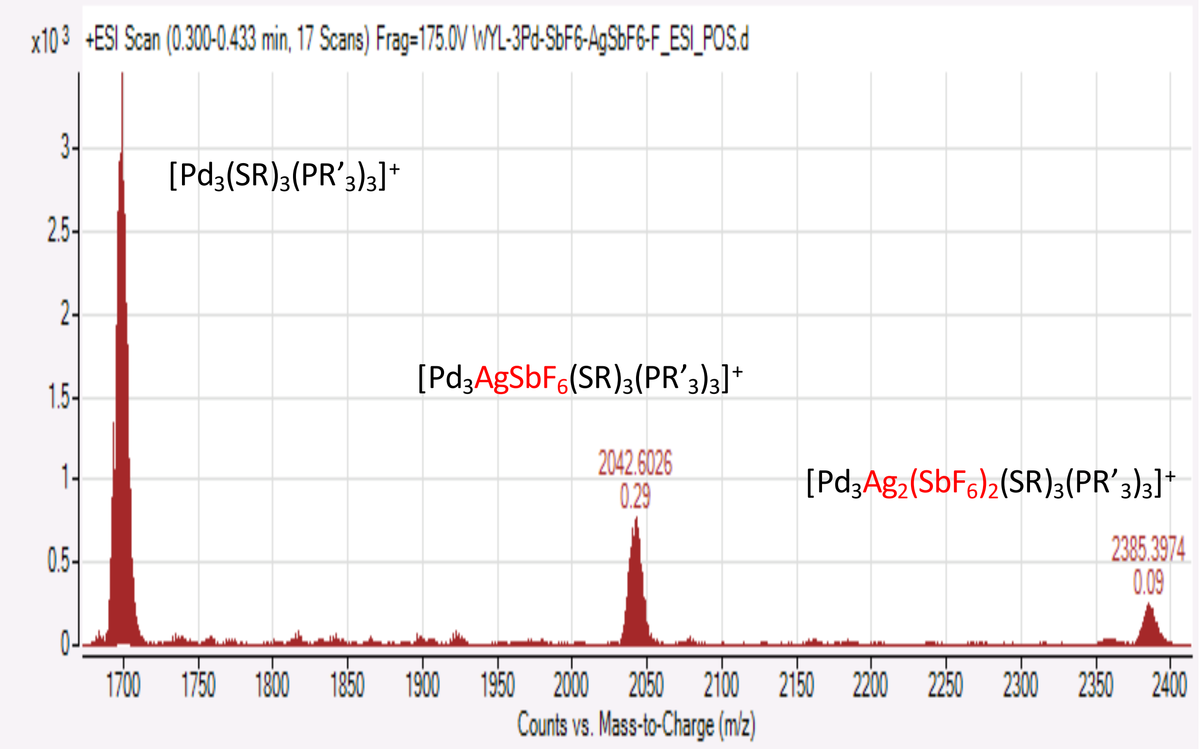

Figure S3. HRMS spectra of the reaction of Pd_3Ag^{++} with excess Ag hexafluoroantimonate, showing the appeareance of pentanuclear species.

Table S1. Comparative ^{31}P NMR data[a]

Complexes	^{31}P NMR (δ_{ppm})[a]
$[Pd_3(SR)_3(PR'_3)_3]^+BF_4$	13.60
$[Pd_3AgBF_4(SR)_3(PR'_3)_3]^+BF_4$	14.65
$[Pd_3Ag_2(BF_4)_2(SR)_3(PR'_3)_3]^+BF_4$	17.03
$[Pd_3Ag_2(CF_3CO_2)_2(SR)_3(PR'_3)_3]^+BF_4$	15.41
$[Pd_3AgSbF_6(SR)_3(PR'_3)_3]^+SbF_6$	14.78
$[Pd_3Ag_2(SbF_6)_2(SR)_3(PR'_3)_3]^+SbF_6$	16.91
$[Pd_3(SR)_3(PPh_3)_3]^+SbF_6$	15.81
$[Pd_3AuPPh_3SbF_6(SR)_3(PPh_3)_3]^+SbF_6$	16.60

[a] ^{31}P NMR were measured in d^6-acetone. Formula in square brackets refer to the cation at highest *m/z* observed by HRMS; -SR = $-SC_6H_4Cl$, $PR'_3 = P(C_6H_4F)_3$.

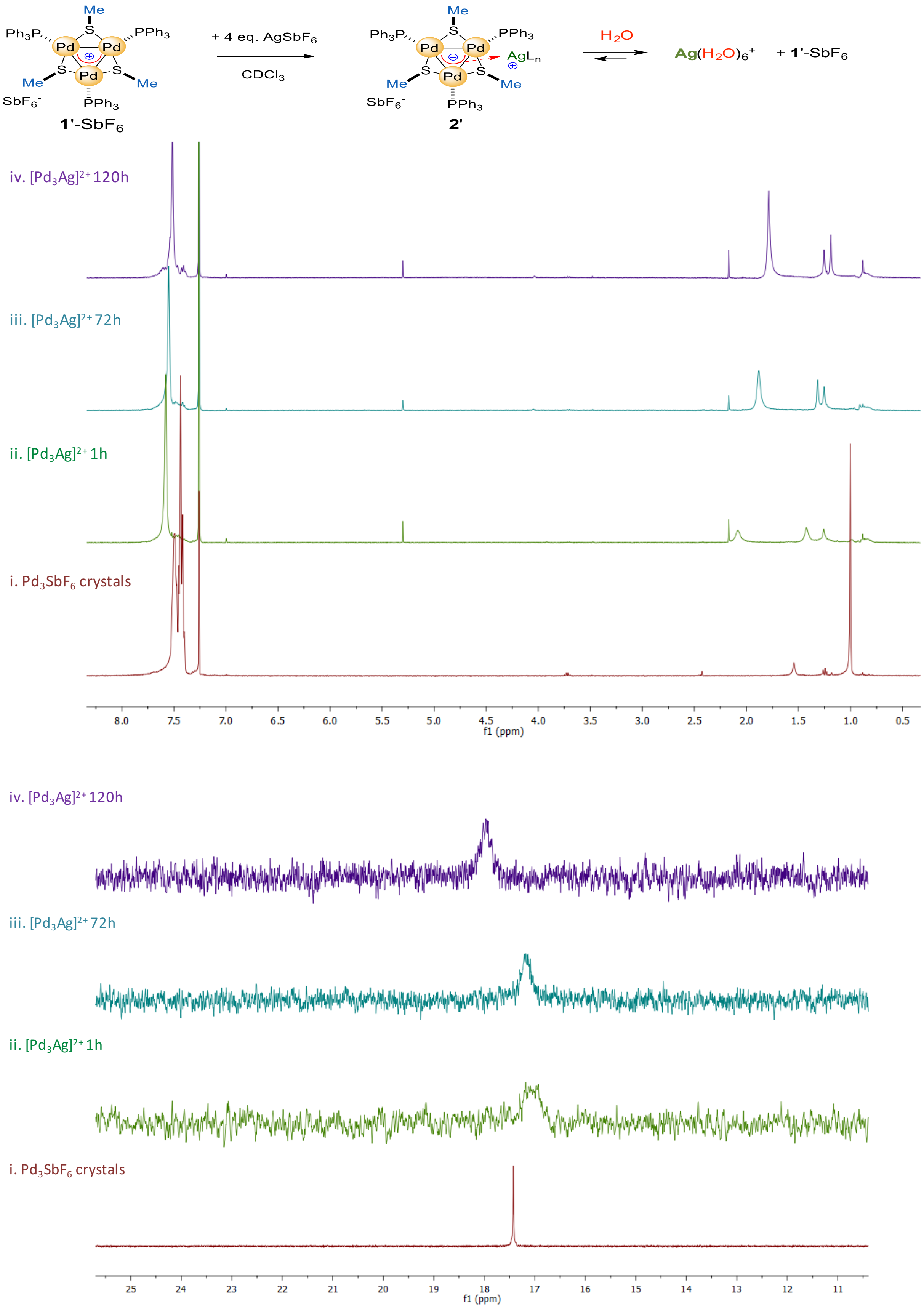

Figure S4. NMR spectra of the dynamic equilibrium shifted by the concentration of water, full 1H and 31P respectively.

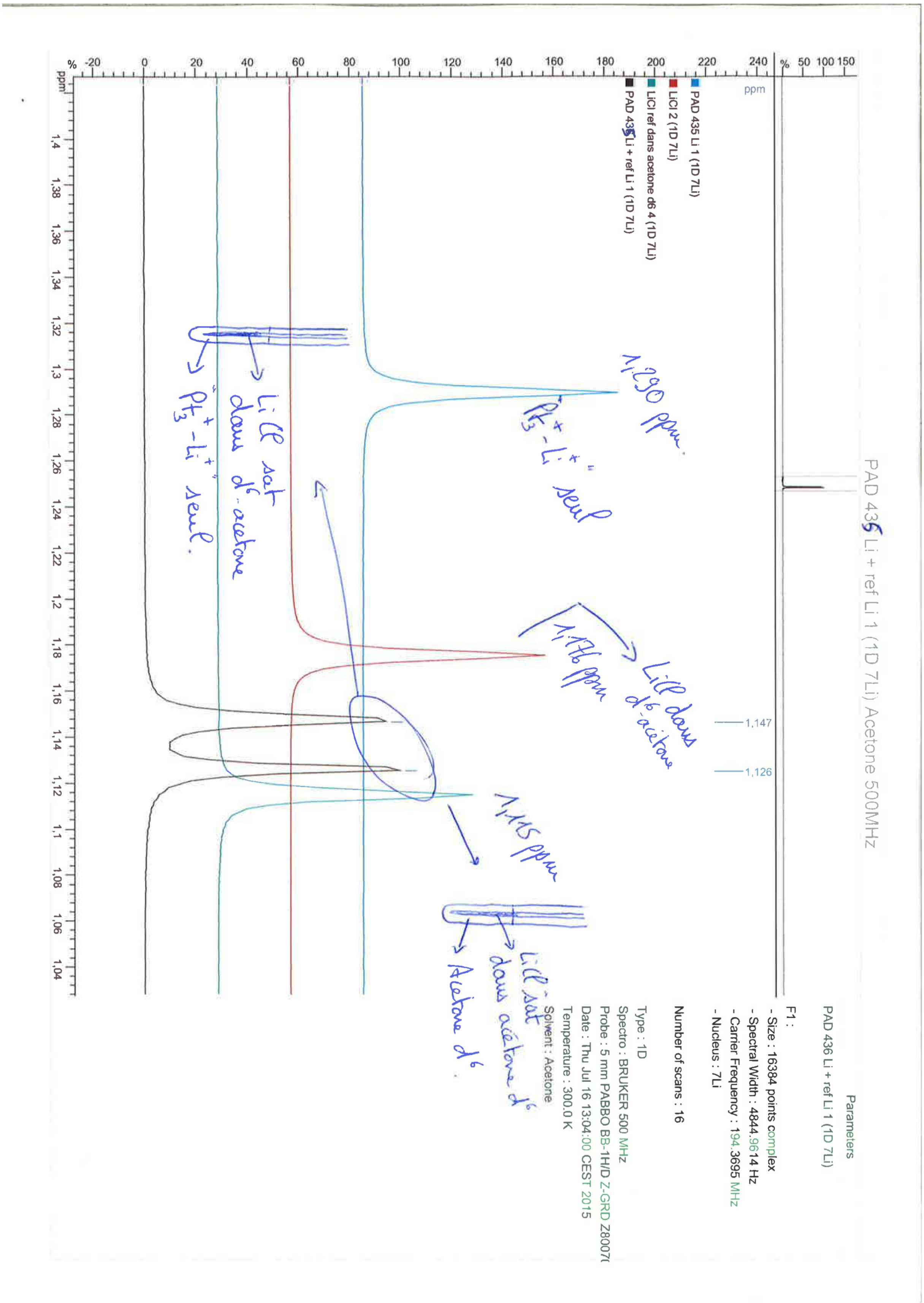

Figure S5. ^{7}Li NMR spectra of mixture of Pt_3^+ complex with LiCl compared to a LiCl reference in a coaxial tube.

4.1. Spectra of 1-OTf

HRMS

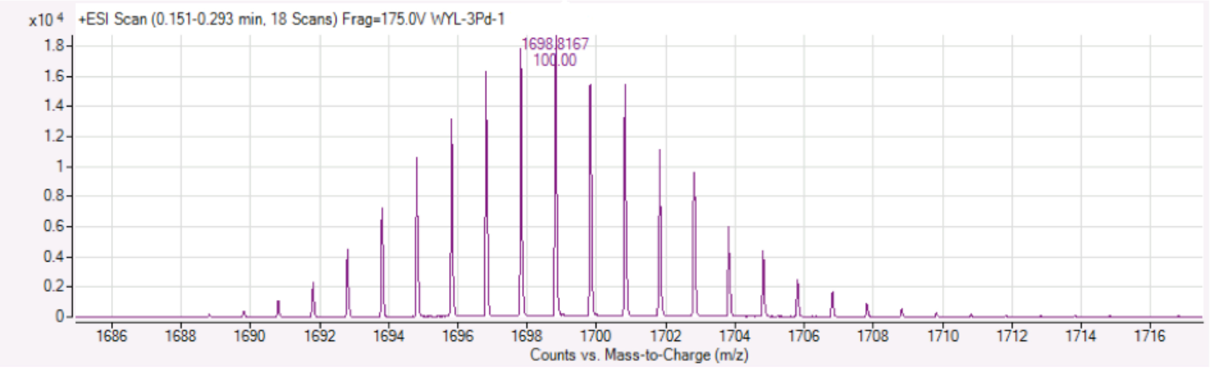

HRMS calculated for cation **1**, $C_{72}H_{48}Cl_3F_9P_3Pd_3S_3^+$ 1698.8170, found 1698.8167.

^{1}H NMR

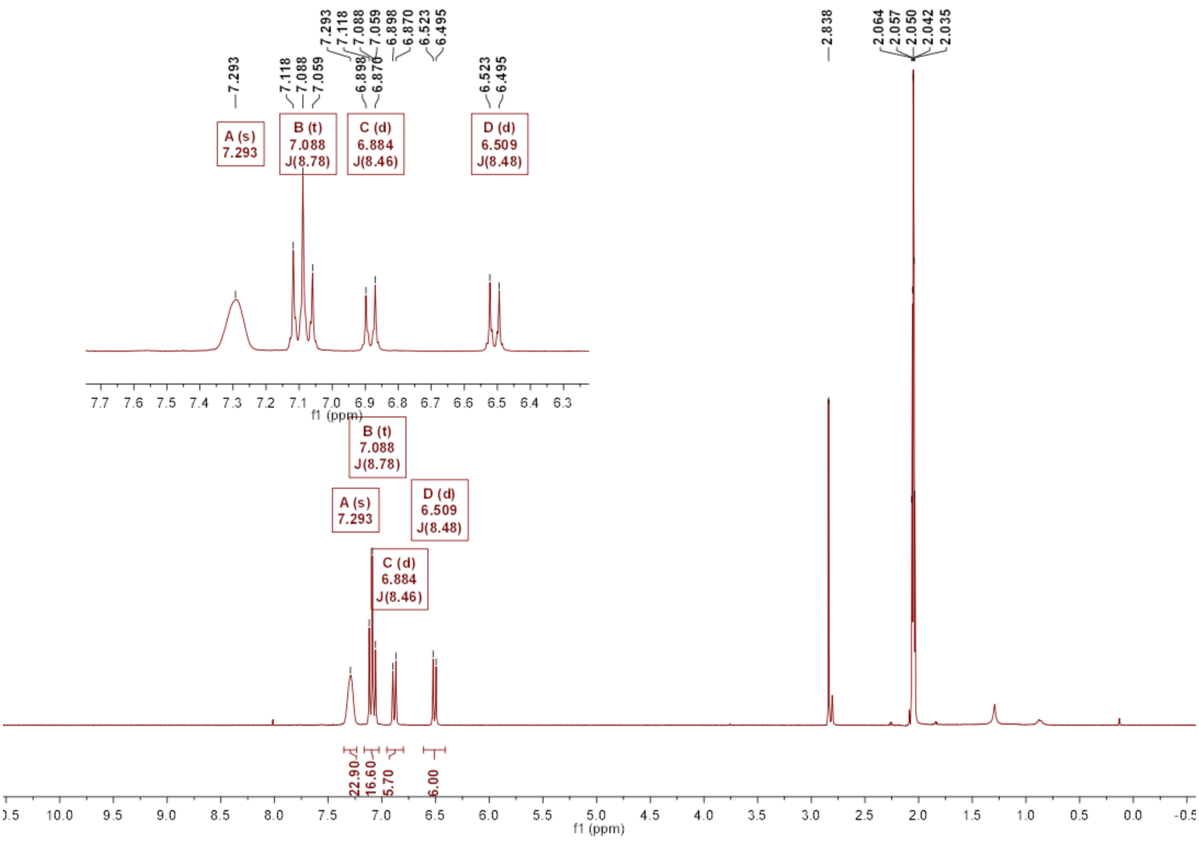

^{13}C NMR

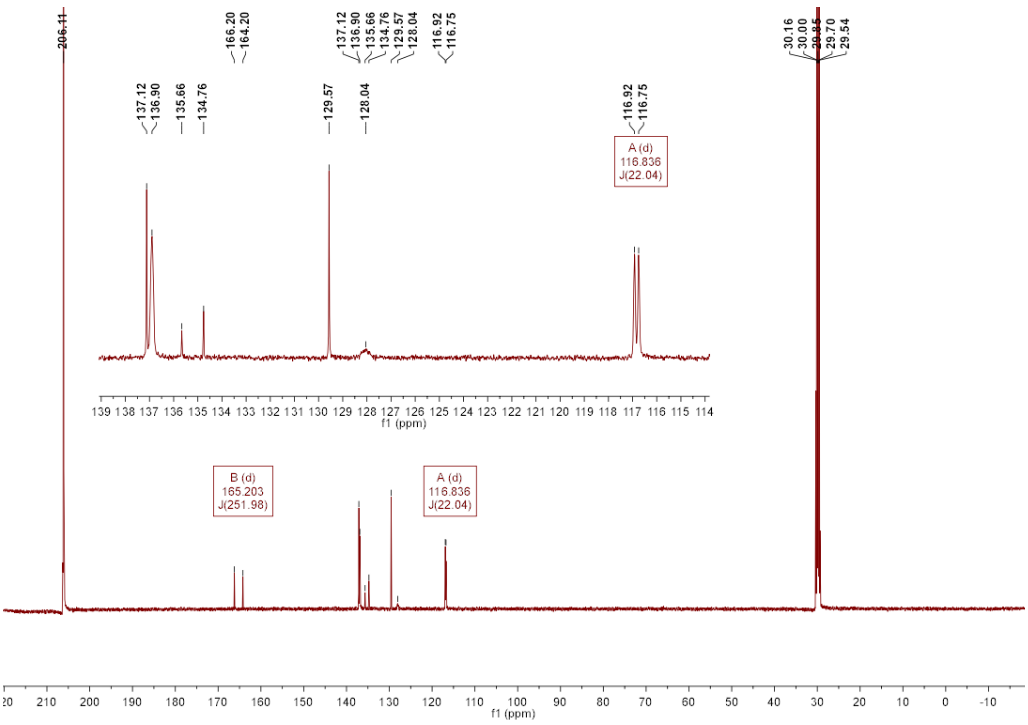

^{31}P NMR

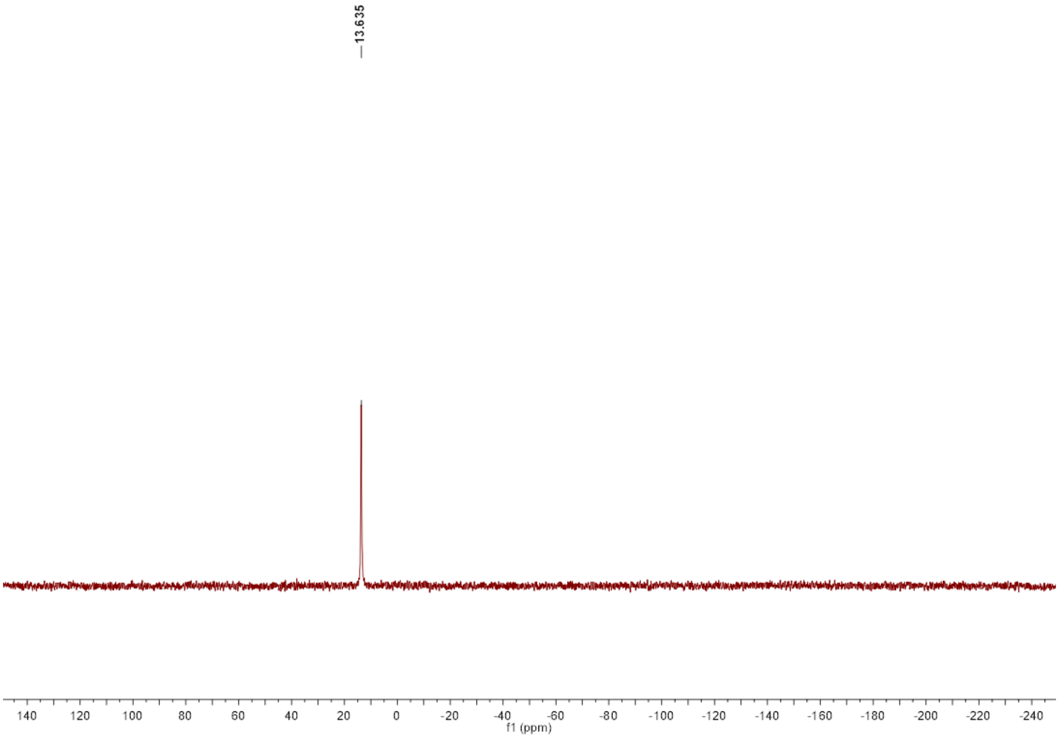

^{19}F NMR

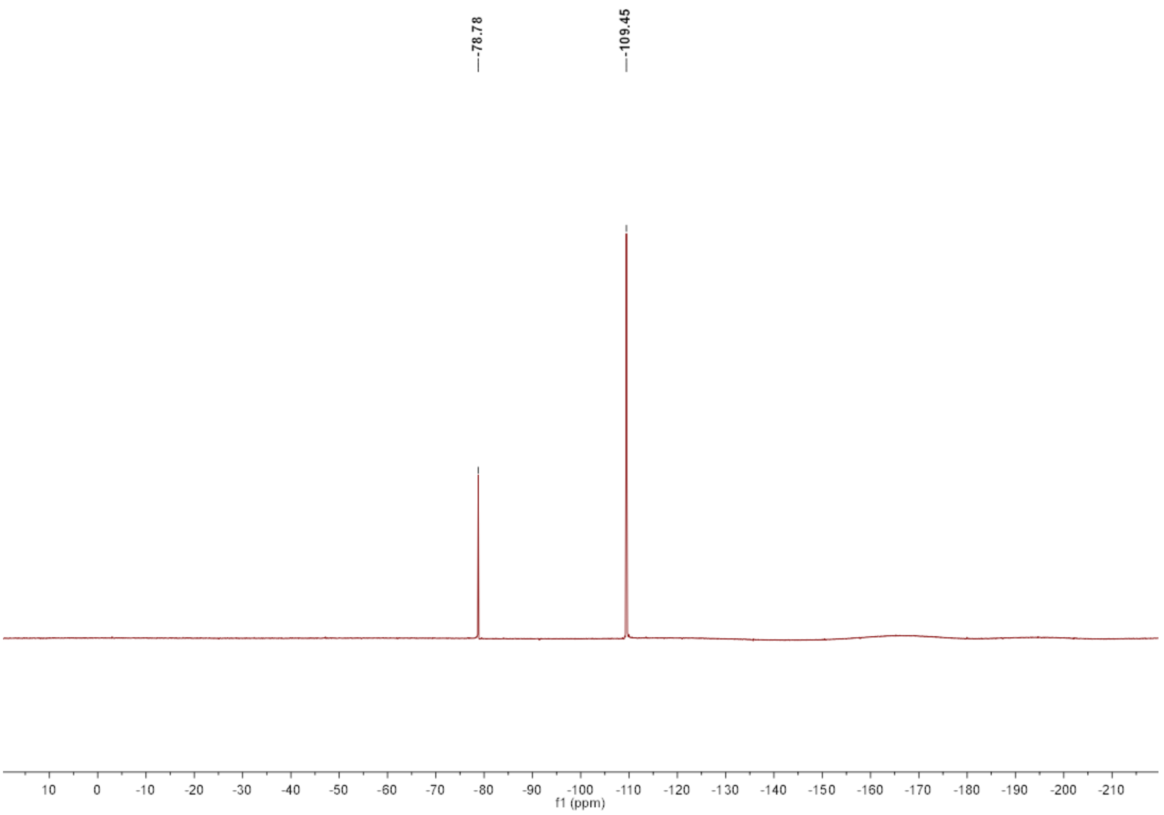

-78.78
-109.45
10
0
-10
-20
-30
-40
-50
-60
-70
-80
-90
-100
-110
-120
-130
-140
-150
-160
-170
-180
-190
-200
-210
f1 (ppm)

FT-IR

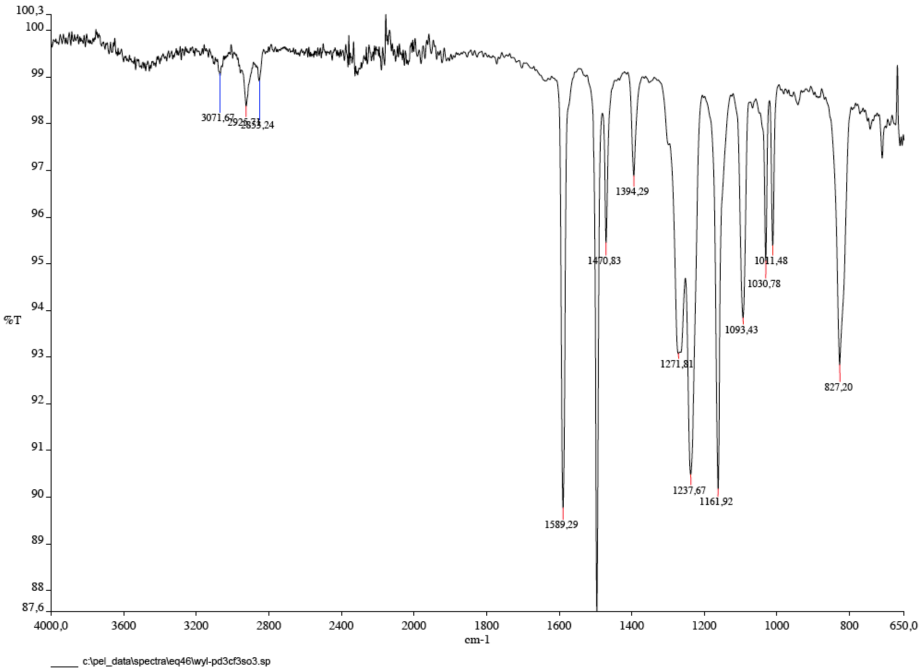

%T
cm-1
3071,67
2926,77
2853,24
1589,29
1470,83
1394,29
1271,81
1237,67
1161,92
1093,43
1030,78
1011,48
827,20
c:\pel_data\spectra\eq46\wyl-pd3cf3so3.sp

UV-vis.

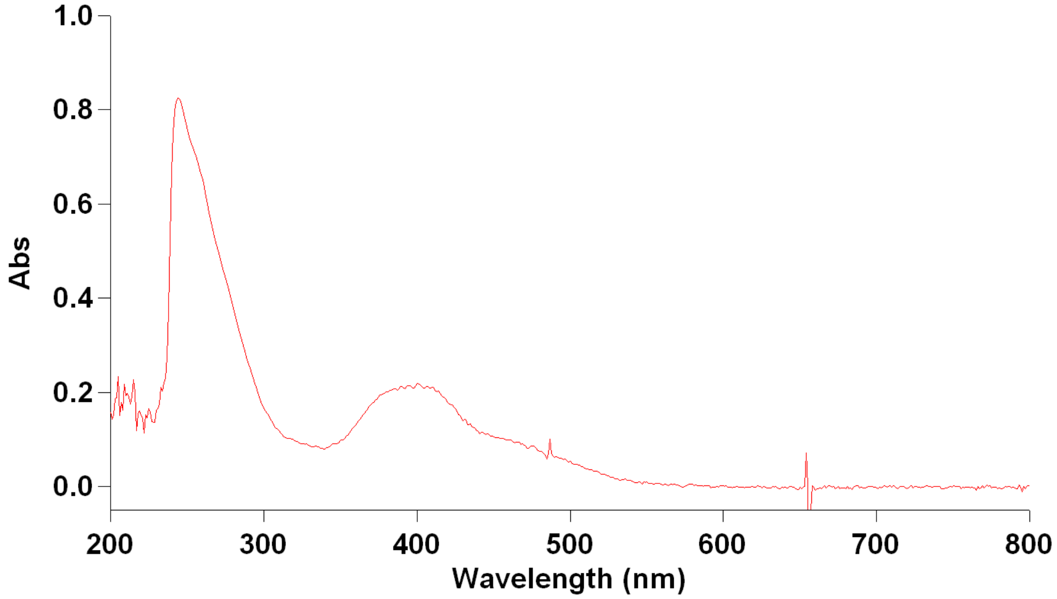

UV-vis. for **1**-OTf: c = 8.5 × 10^{-6} mol/L in $CHCl_3$, λ_{max} = 243 nm, ε_{max} = 9.5 × 10^4 M^{-1} cm^{-1}.

4.2. Spectra of 1-SbF_6

HRMS

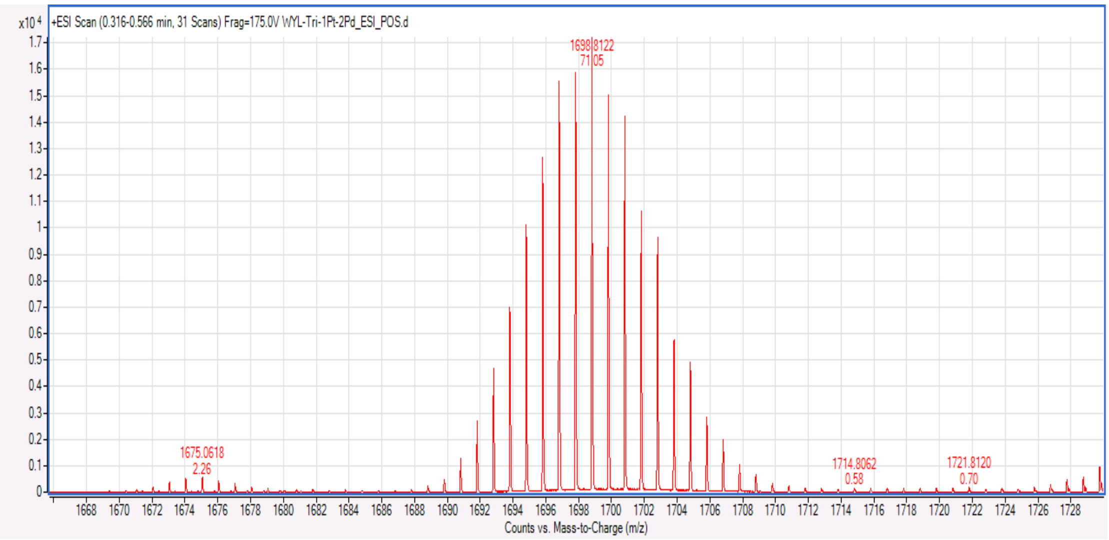

HRMS calculated for cation **1**, $C_{72}H_{48}Cl_3F_9P_3Pd_3S_3^+$ 1698.8170, found 1698.8122.

^{1}H NMR

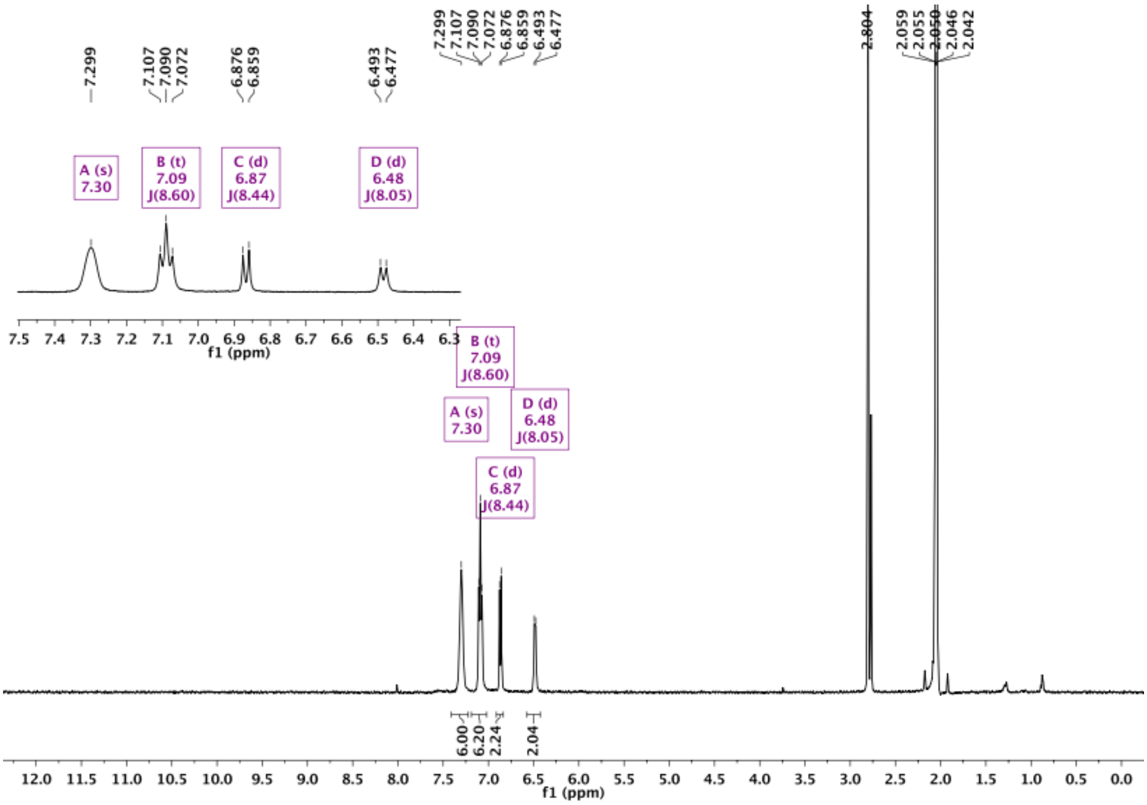

^{13}C NMR

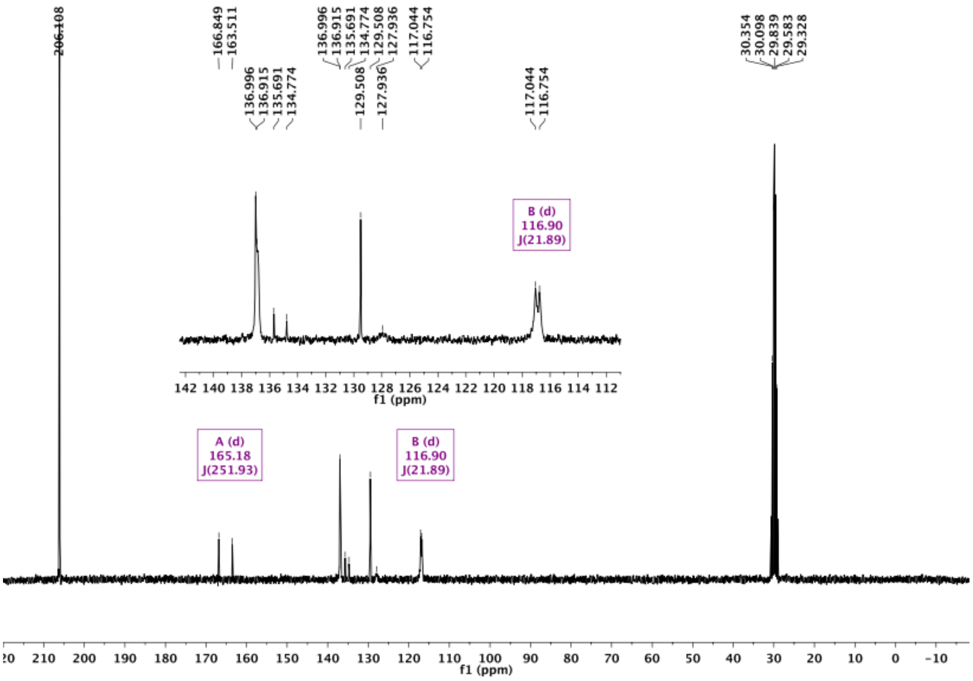

^{31}P NMR

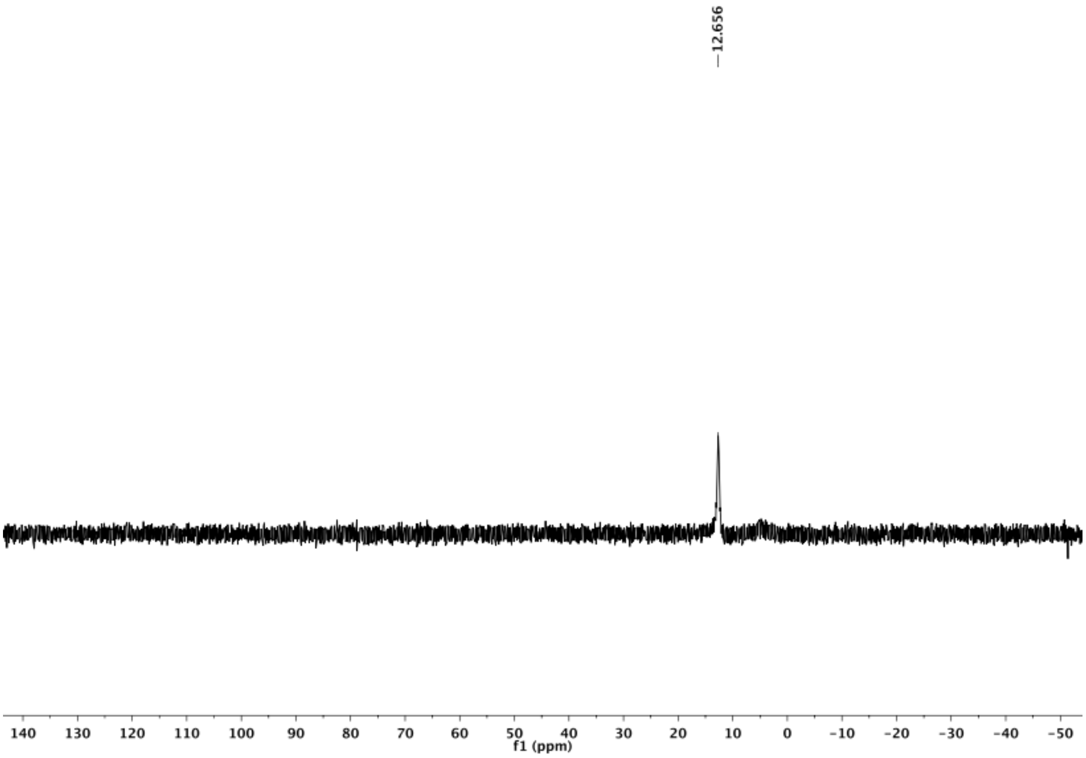

^{19}F NMR

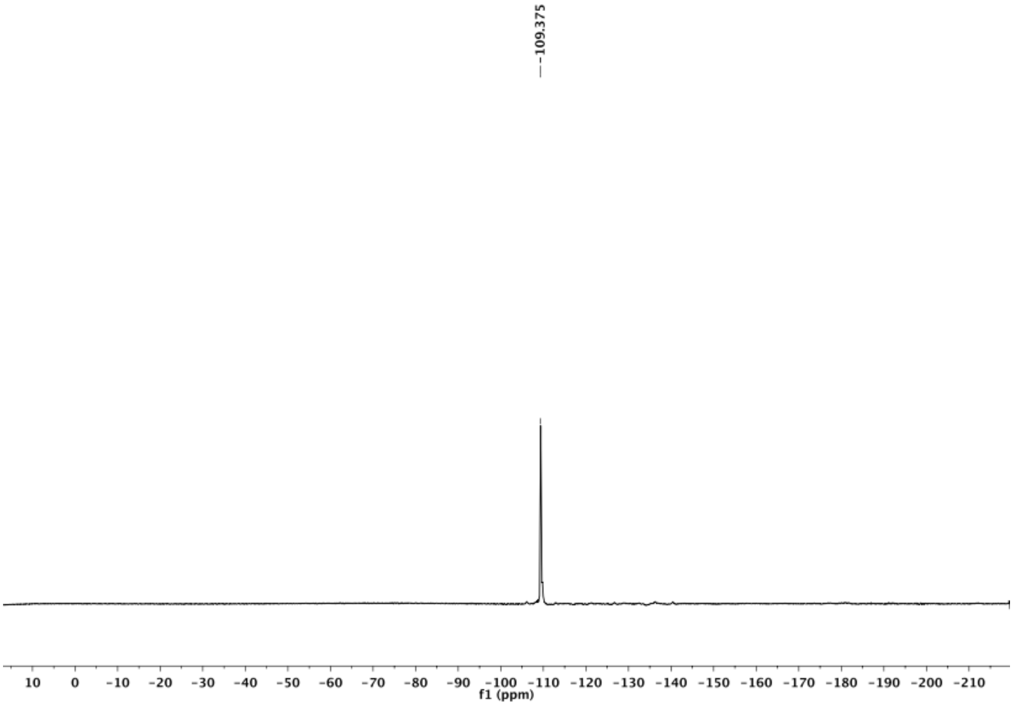

FT-IR (cm^{-1})

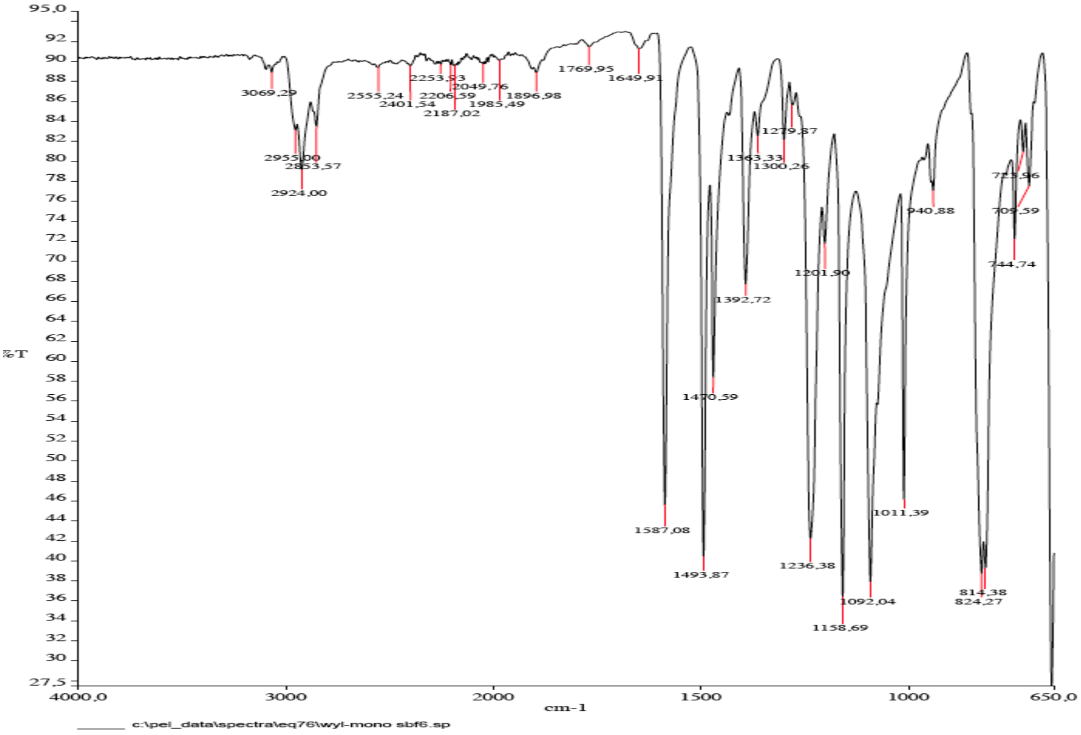

UV-vis.

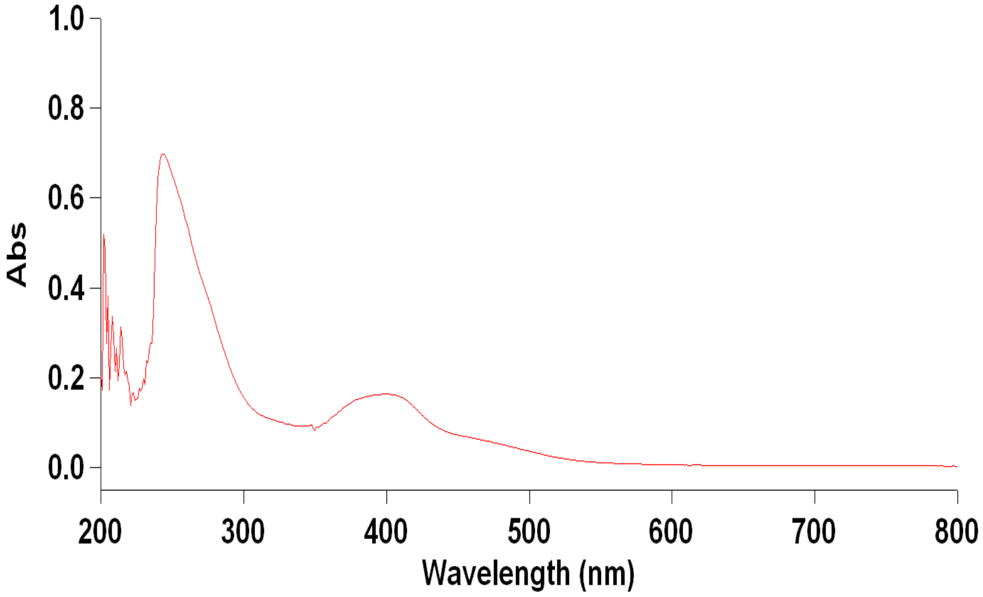

UV-vis. of **1**-SbF_6: c = 1.59 × 10^{-5} mol/L in $CHCl_3$, λ_{max} = 244 nm, ε_{max} = 0.44 × 10^5 M^{-1} cm^{-1}.

4.3. Spectra of 1-BF4

HRMS

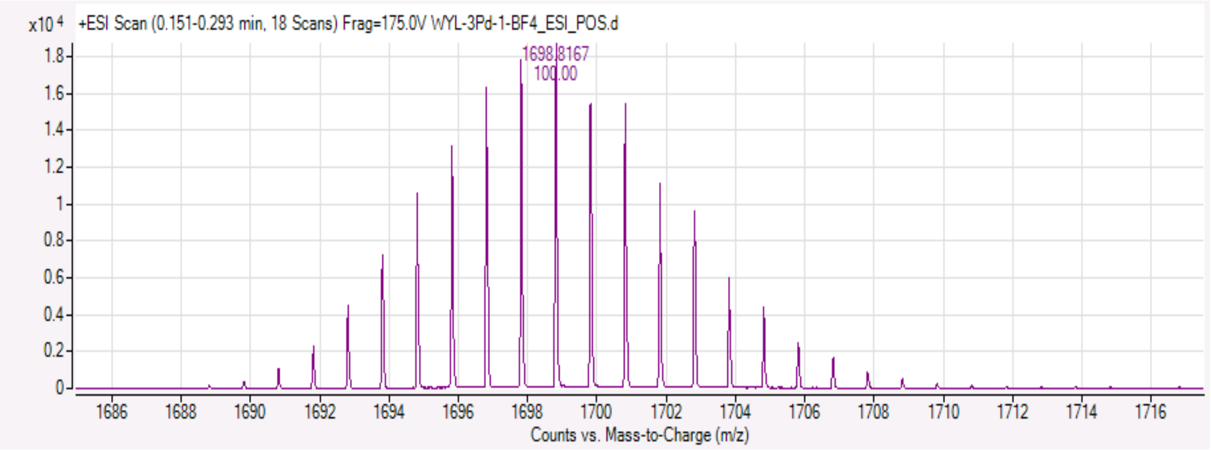

HRMS calculated for $C_{72}H_{48}Cl_3F_9P_3Pd_3S_3^+$ 1698.8170, found 1698.8167.

^{1}H NMR

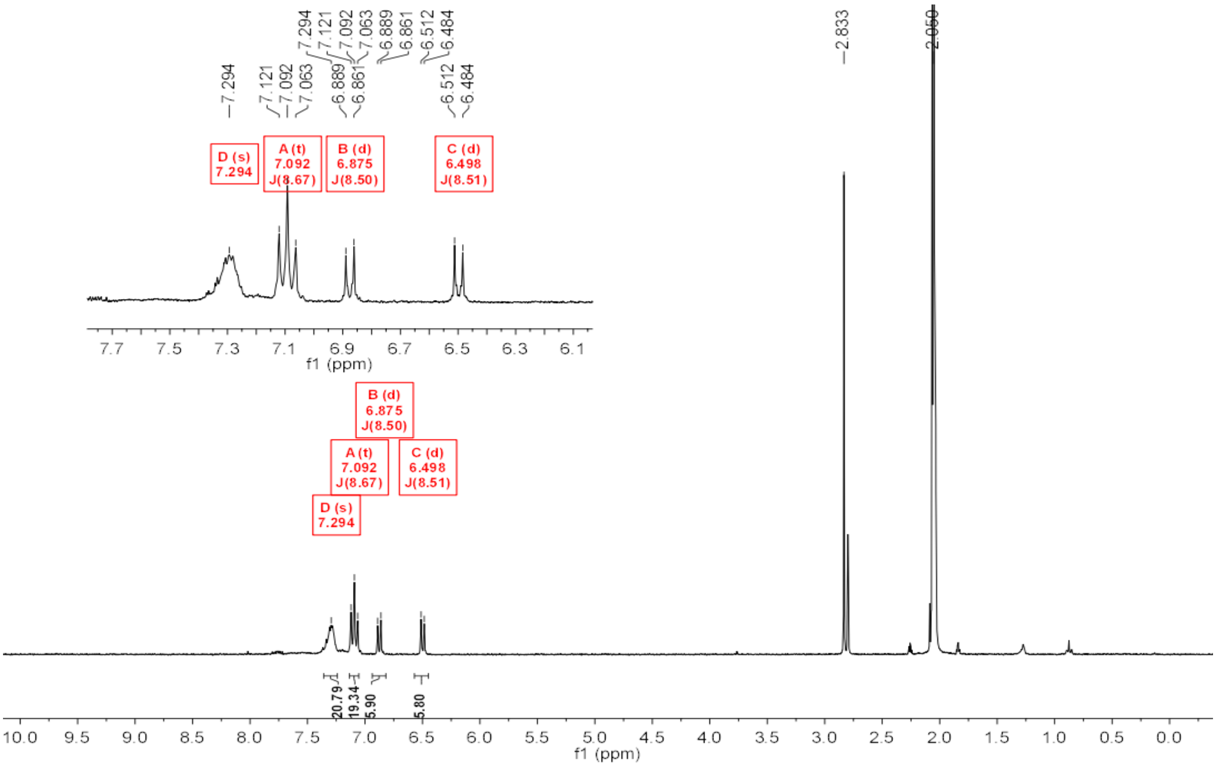

^{13}C NMR

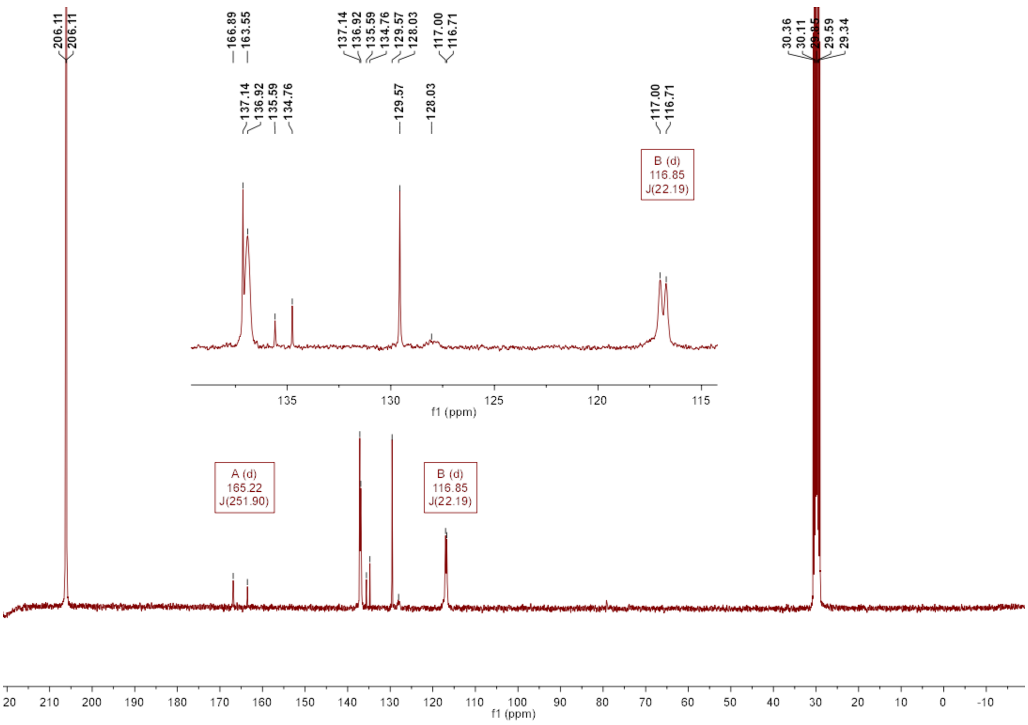

^{31}P NMR

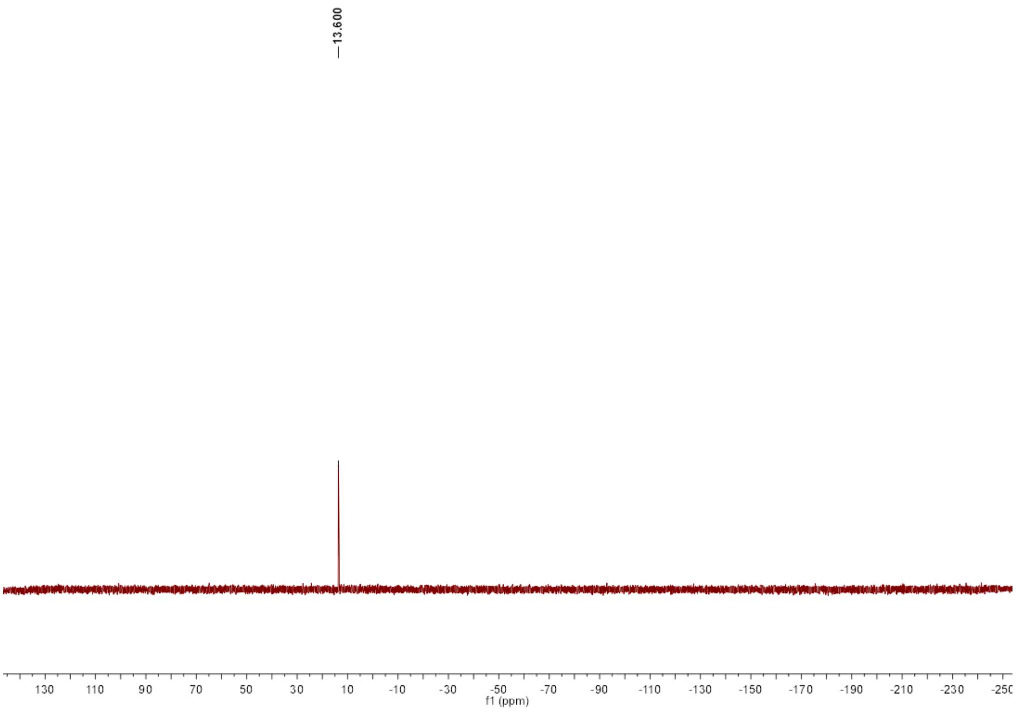

^{19}F NMR

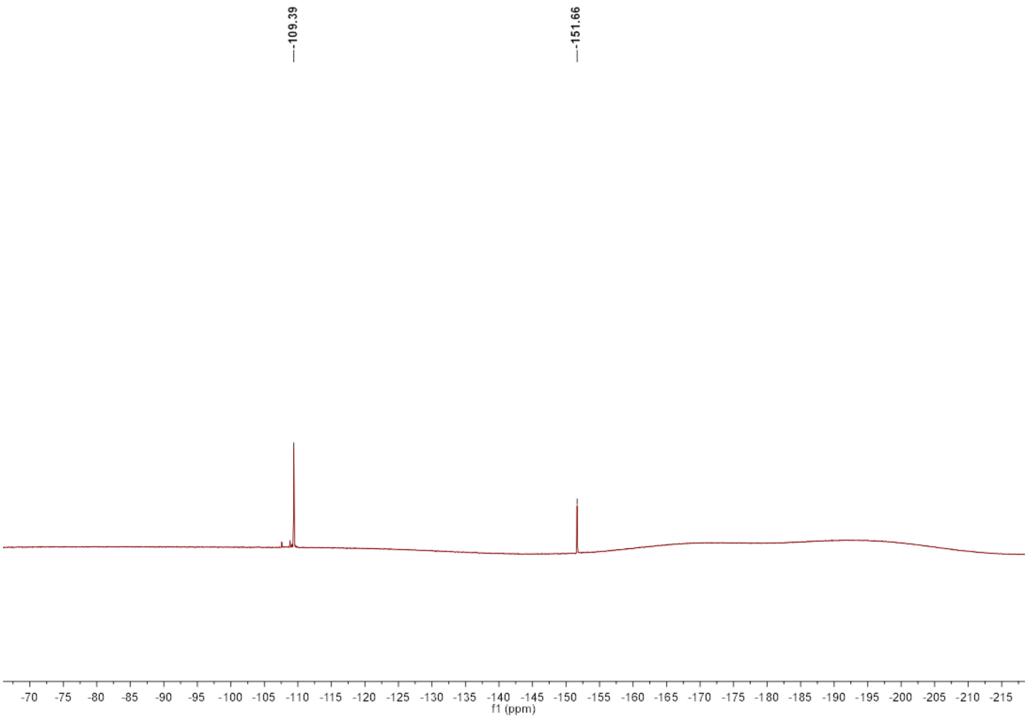

FT-IR

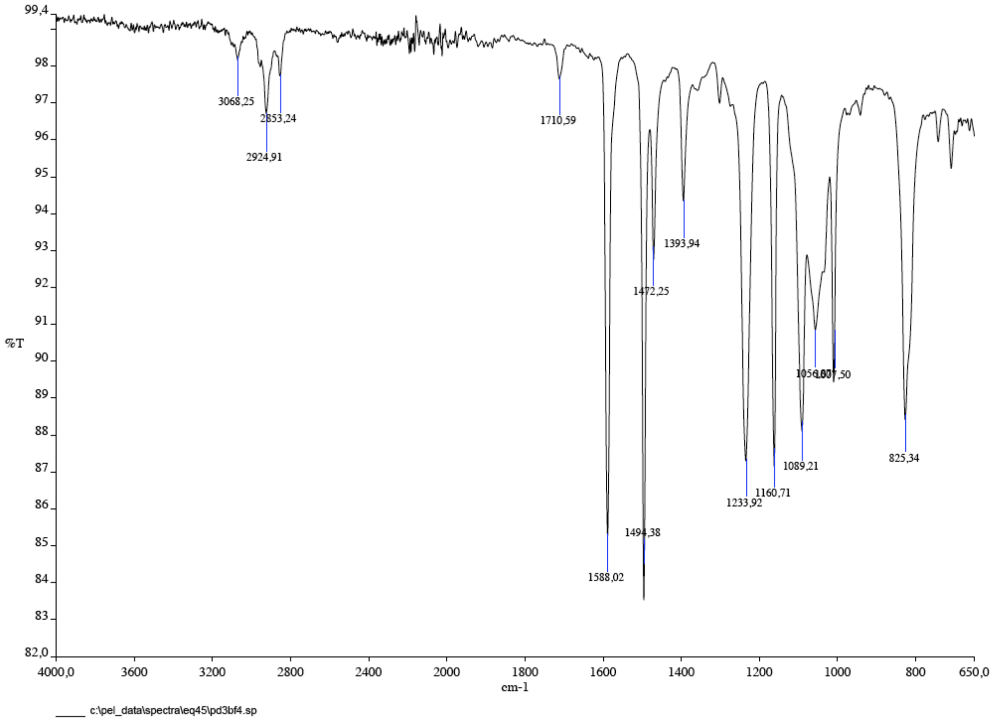

UV-vis.

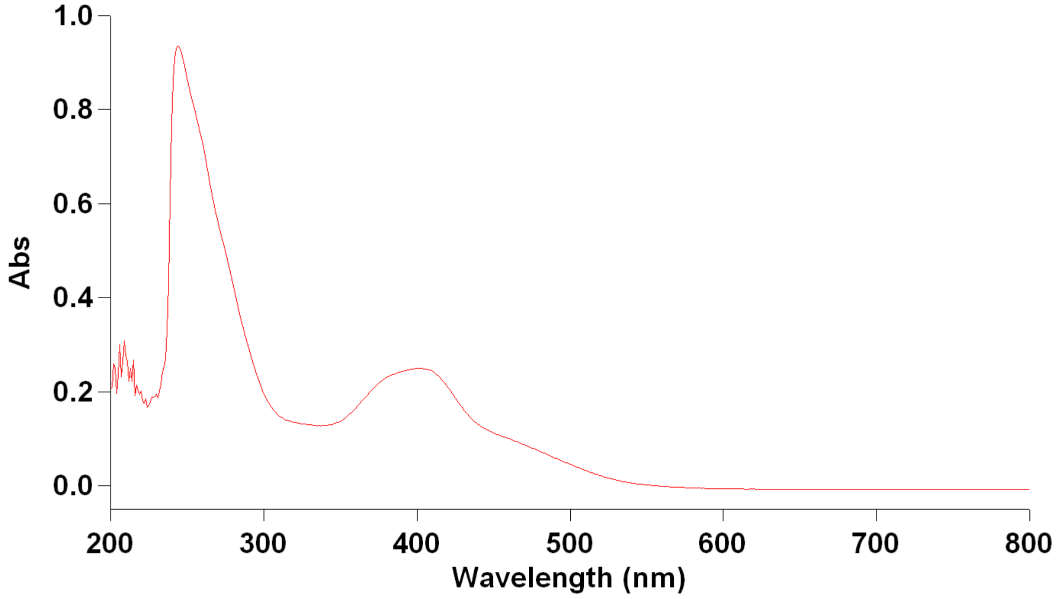

UV-vis. for **1**-BF_4: c = 1 × 10^{-5} mol/L in $CHCl_3$, λ_{max} = 244 nm, ε_{max} = 9.3 × 10^4 M^{-1} cm^{-1}.

4.4. Spectra of 1-CF_3CO_2

HRMS

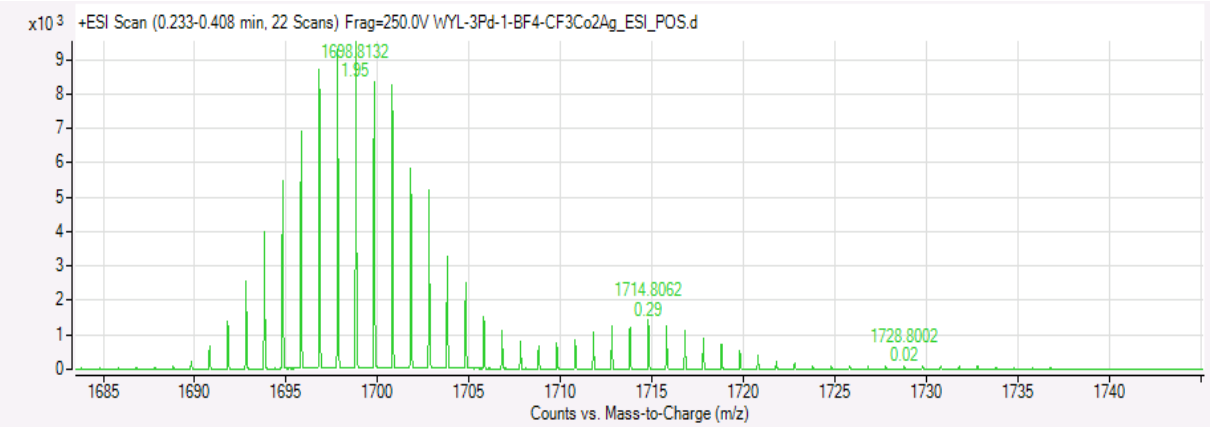

HRMS calculated for $C_{72}H_{48}Cl_3F_9P_3Pd_3S_3^+$ 1698.8170, found 1698.8132.

^{1}H NMR

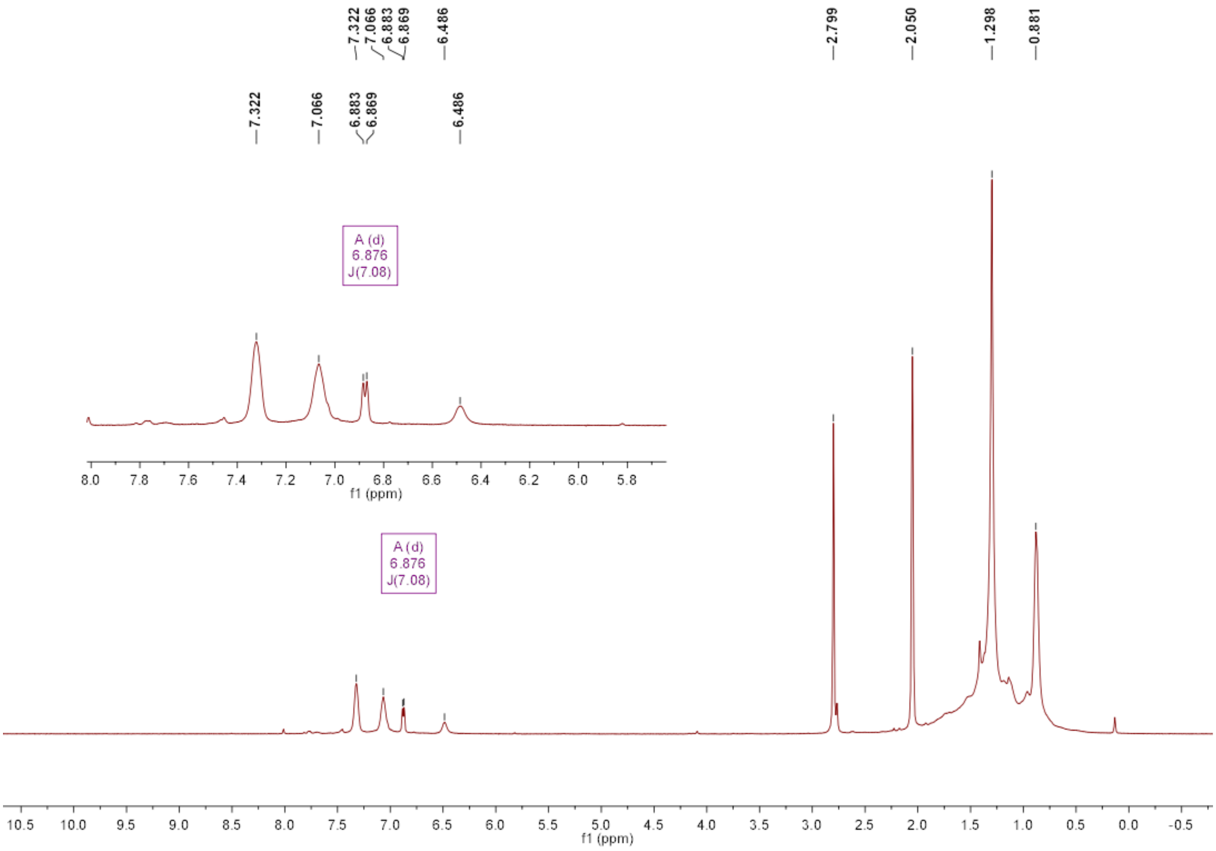

^{13}C NMR

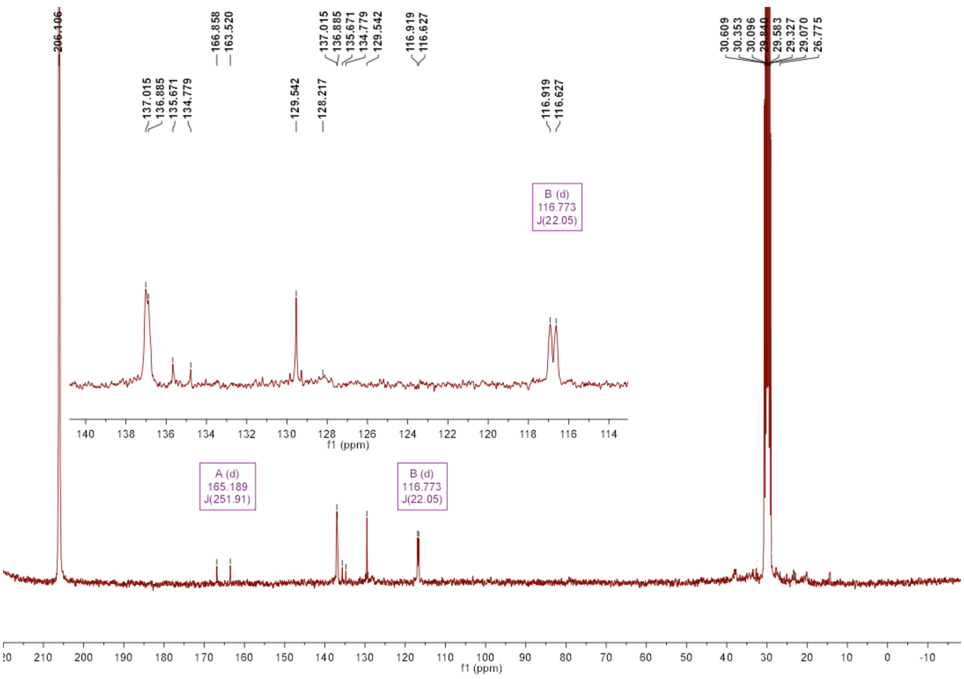

^{31}P NMR

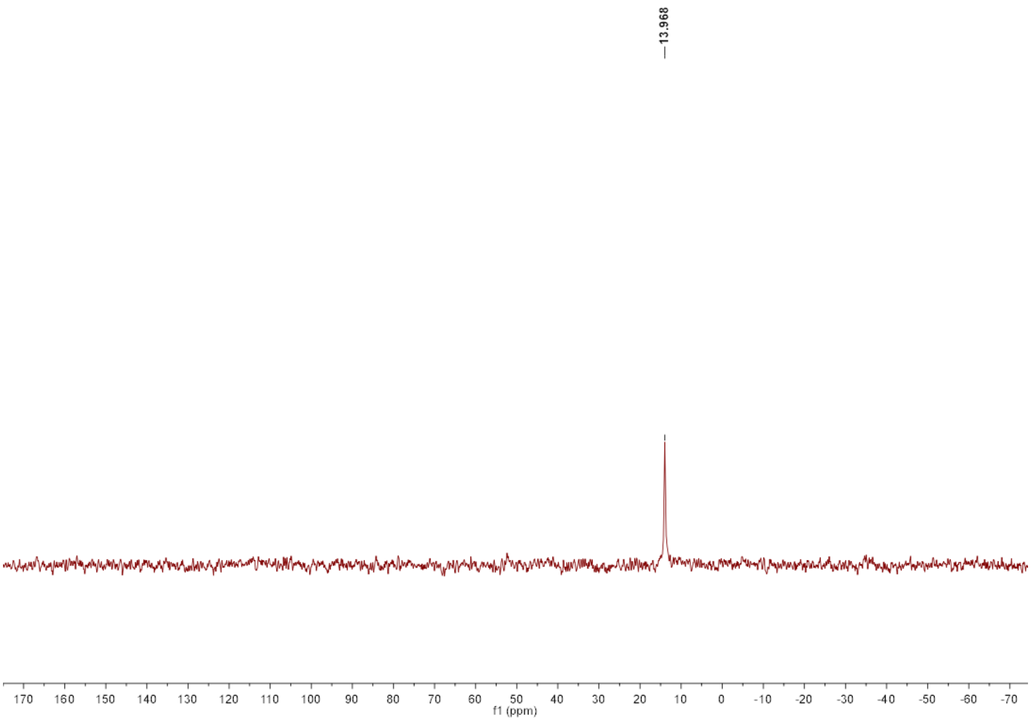

^{19}F NMR

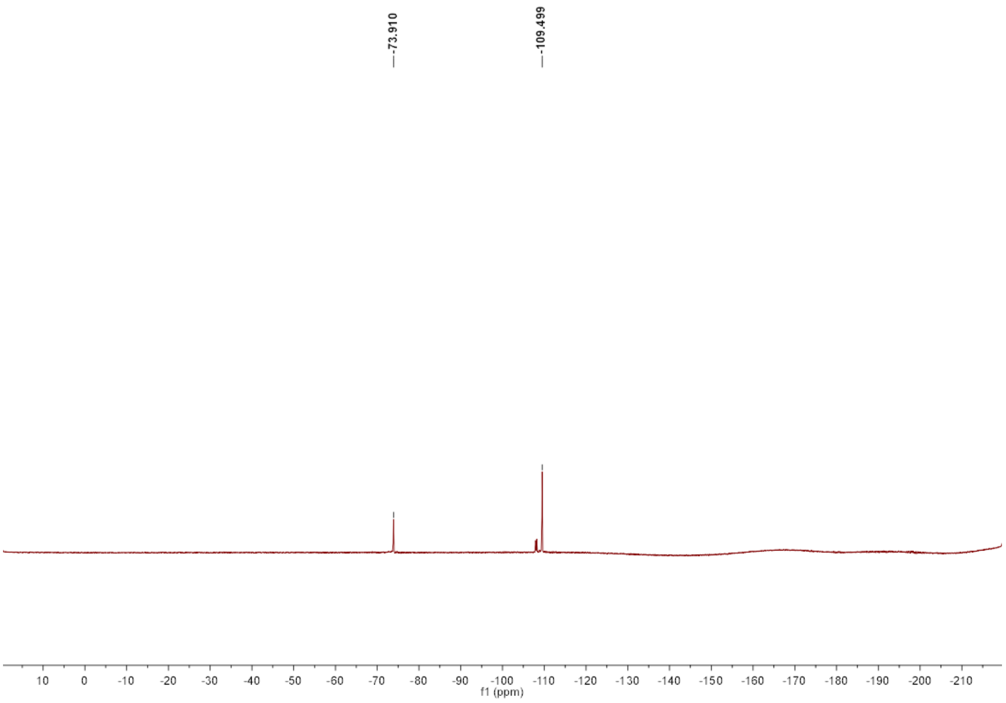

-73.910
-109.499
10 0 -10 -20 -30 -40 -50 -60 -70 -80 -90 -100 -110 -120 -130 -140 -150 -160 -170 -180 -190 -200 -210
f1 (ppm)

FT-IR

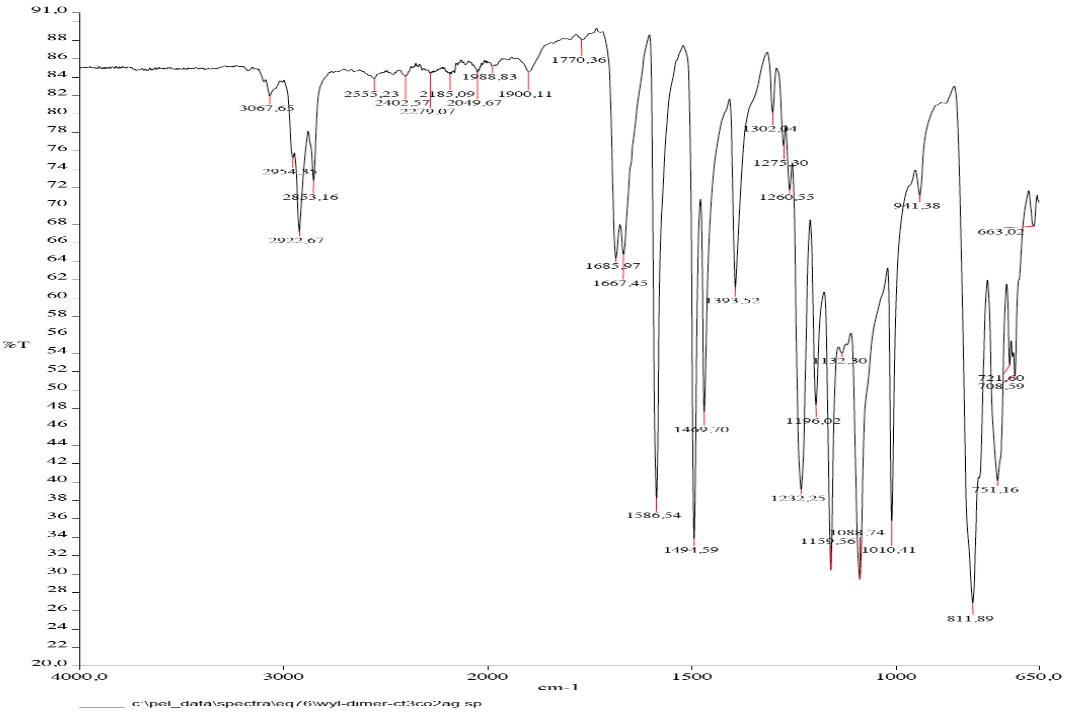

%T
cm-1
4000,0 3000 2000 1500 1000 650,0
3067,65
2954,35
2853,16
2922,67
2555,23
2402,57
2279,07
2185,09
2049,67
1988,83
1900,11
1770,36
1685,97
1667,45
1586,54
1494,59
1469,70
1393,52
1302,04
1275,30
1260,55
1232,25
1196,02
1132,30
1159,56
1088,74
1010,41
941,38
811,89
751,16
721,60
708,59
663,02
c:\pel_data\spectra\eq76\wyl-dimer-cf3co2ag.sp

UV-vis.

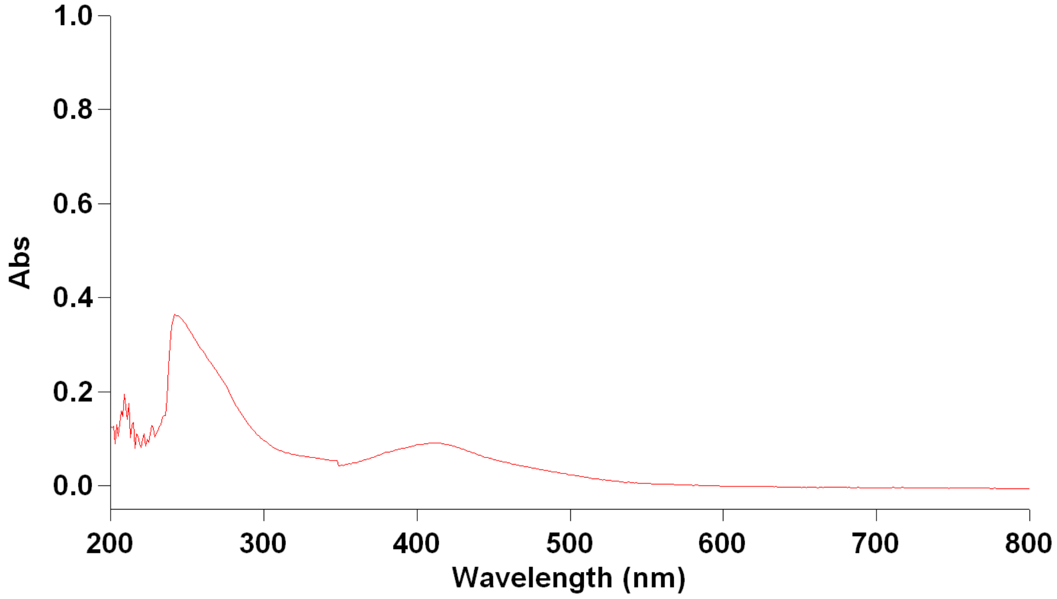

UV-vis for **1**-CF_3CO_2: c = 5 × 10^{-6} mol/L in $CHCl_3$, λ_{max} = 242 nm, ε_{max} = 7.2 × 10^4 M^{-1} cm^{-1}.

4.5. Spectra of 1- SbF_6 with PPh_3 ligand

HRMS

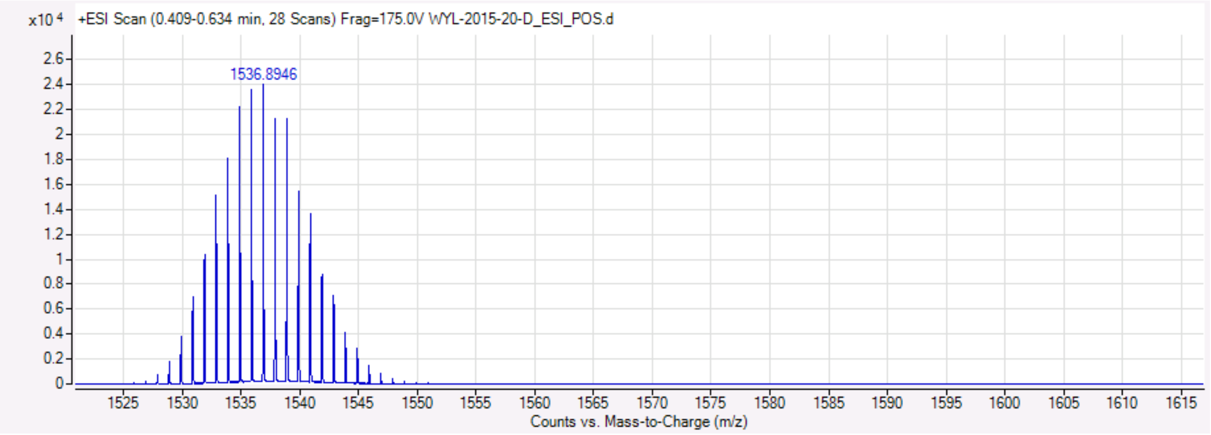

HRMS calculated for $C_{72}H_{57}Cl_3P_3Pd_3S_3^+$ 1536.8901, found 1536.8946.

^{1}H NMR

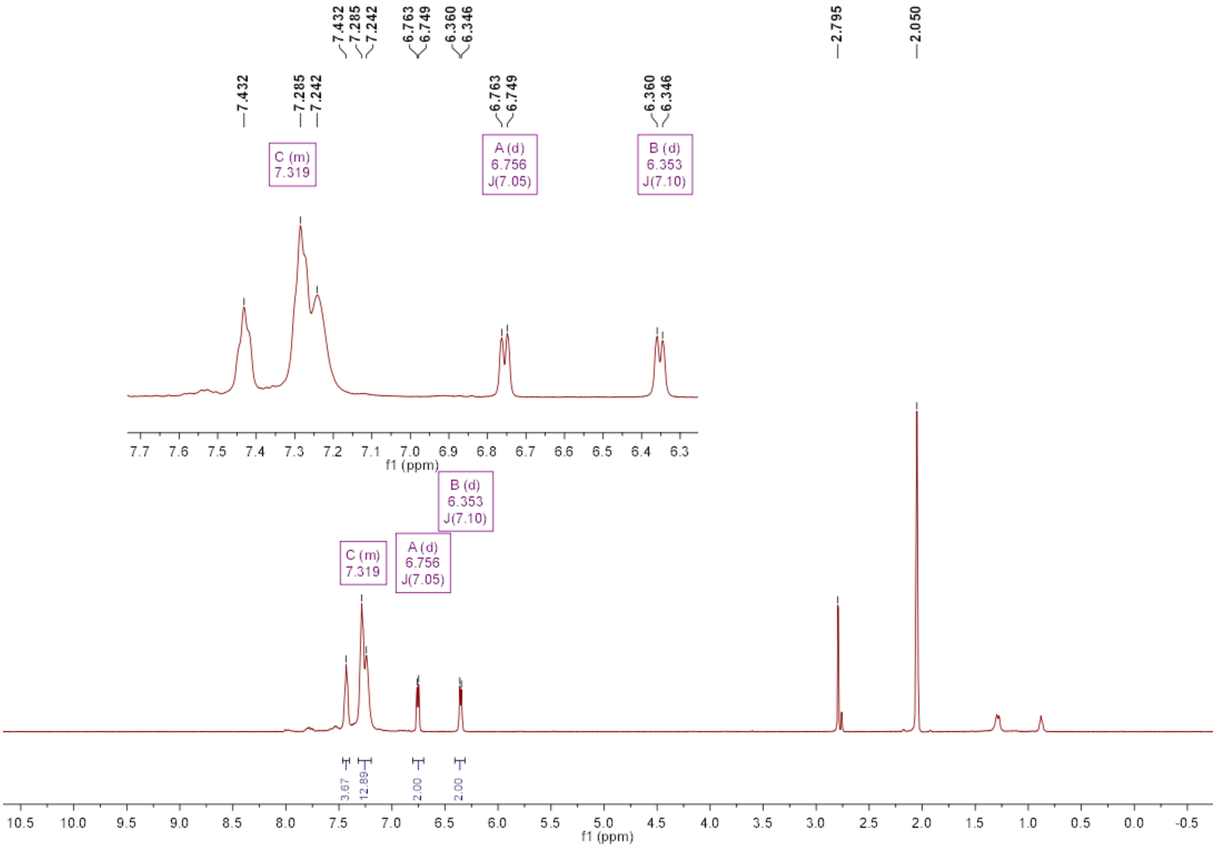

^{31}P NMR

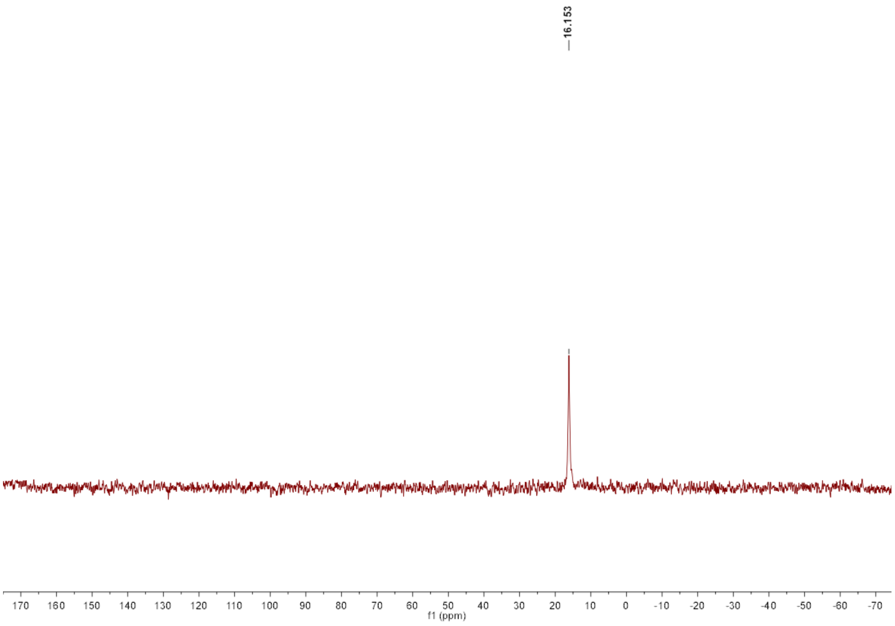

^{13}C NMR

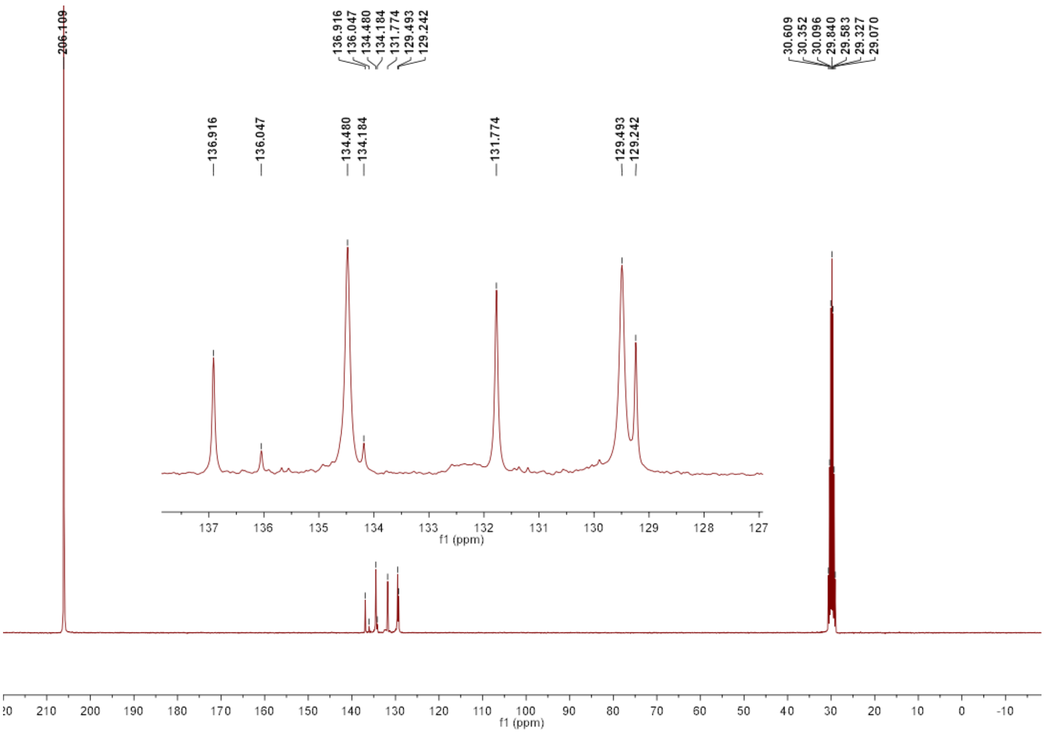

FT-IR

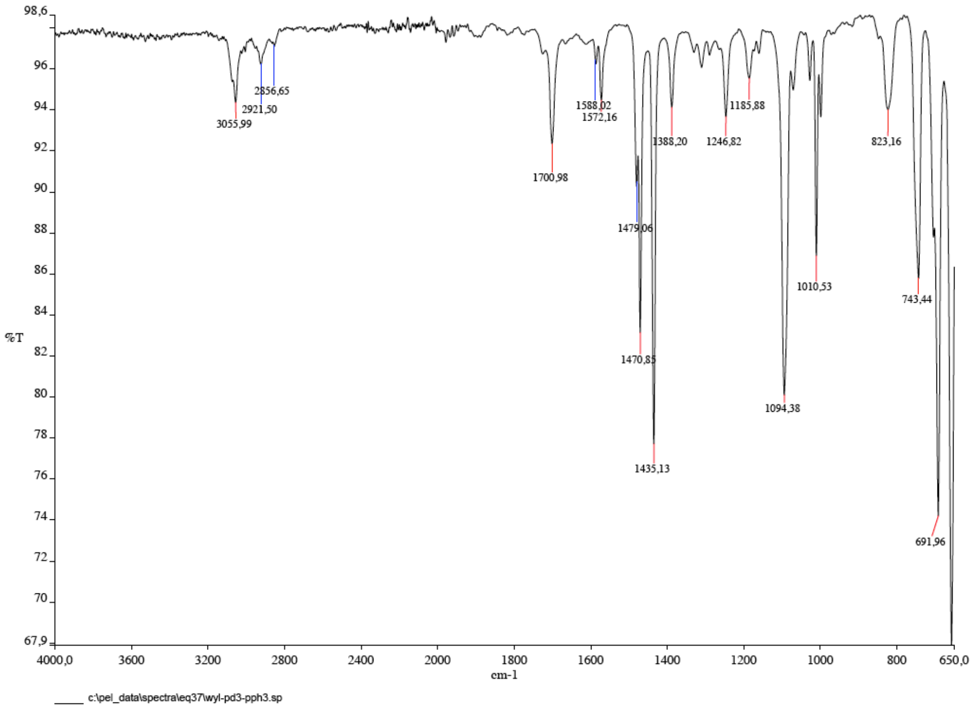

UV-vis.

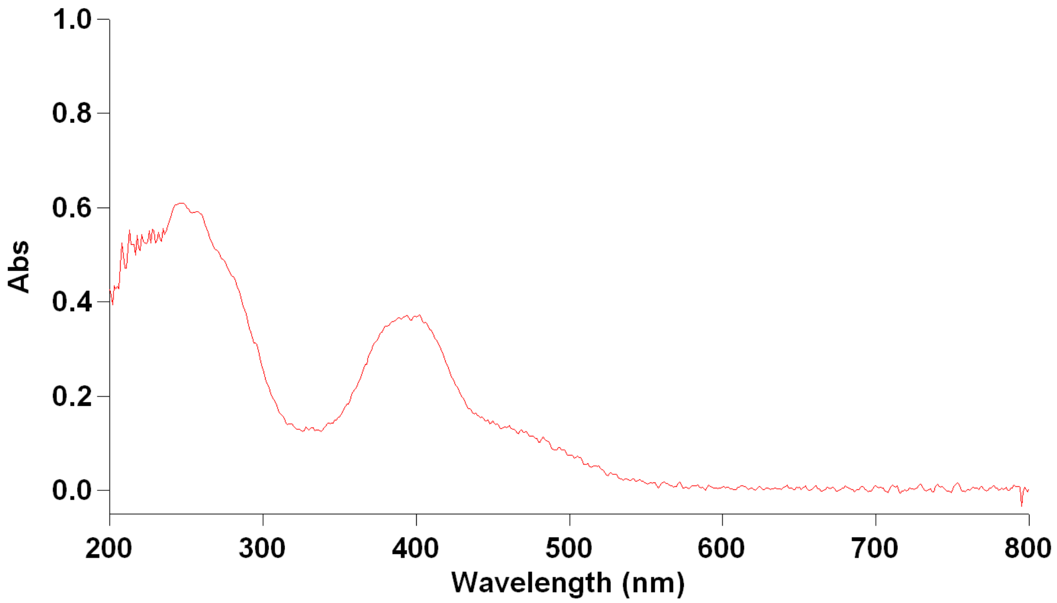

UV-vis. for **1**-SbF_6 with PPh_3 as ligand: $c = 1 \times 10^{-5}$ mol/L in $CHCl_3$, $\lambda_{max} = 246$ nm, $\varepsilon_{max} = 6.2 \times 10^4$ M^{-1} cm^{-1}.

4.6. Spectra of **2**-SbF_6

HRMS

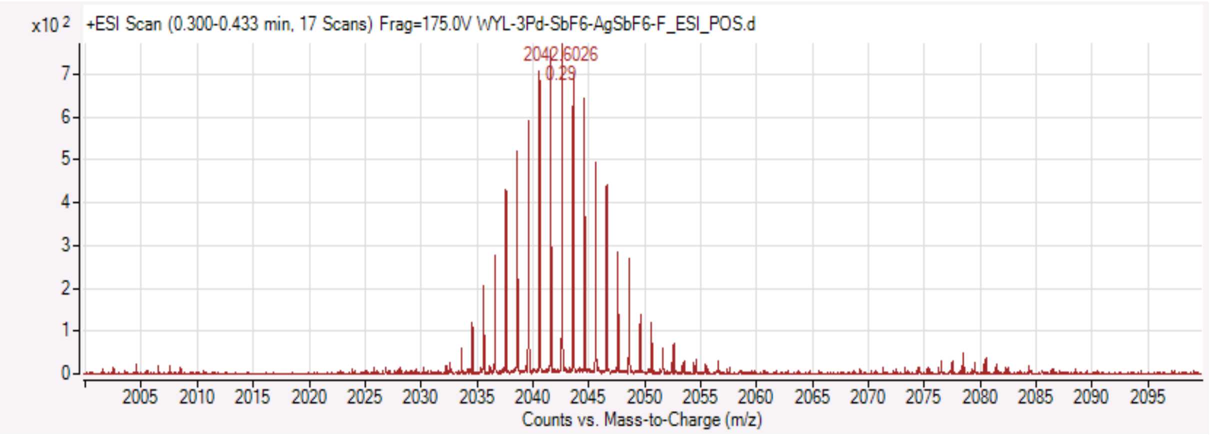

HRMS calculated for $C_{72}H_{48}Cl_3F_9P_3Pd_3S_3AgSbF_6^+$ 2042.6163, found 2042.6026.

^{1}H NMR

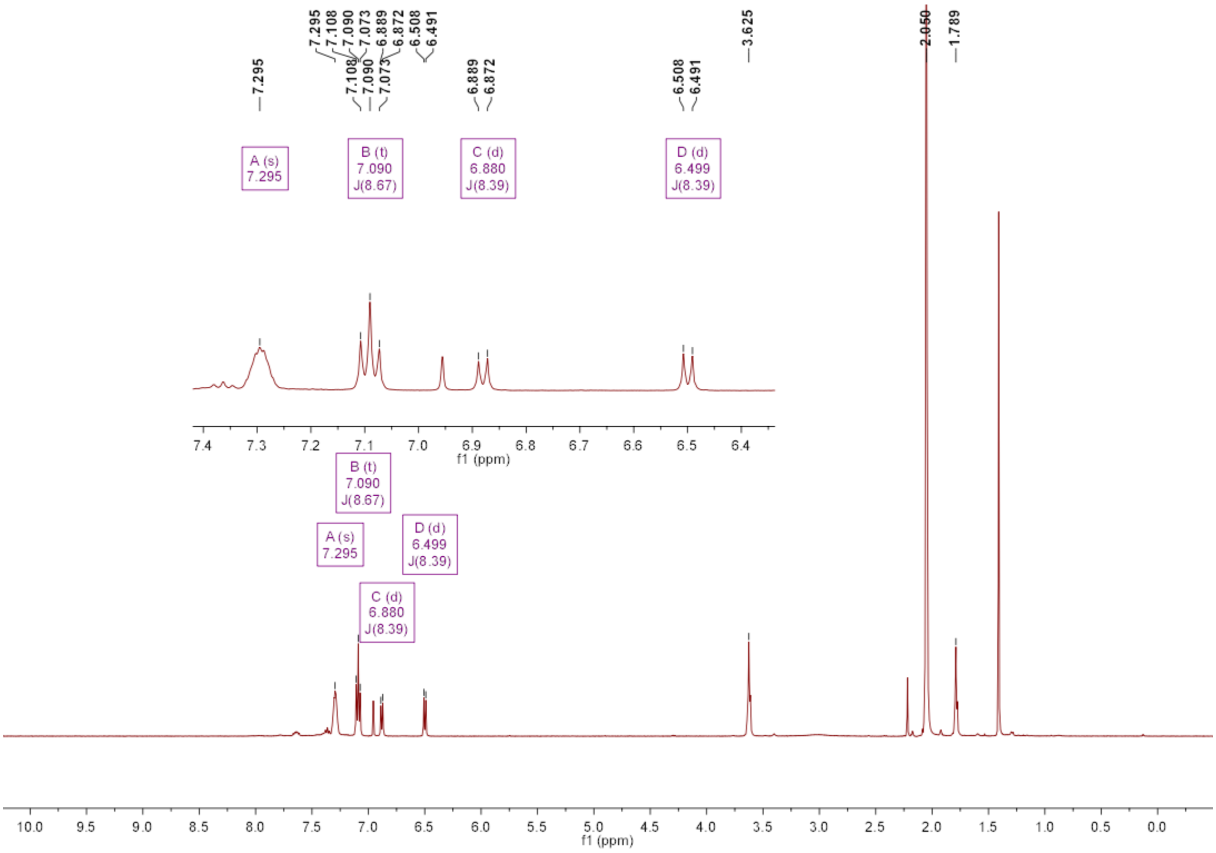

^{1}H NMR (500 MHz, CD_3COCD_3): δ 7.30 (br s, 18H, H2), 7.09 (t, J = 8.7 Hz, 18H, H3), 6.88 (d, J = 8.4 Hz, 6H, H7), 6.50 (d, J = 8.4 Hz, 6H, H6); 1.78, 3.63 (THF).

^{13}C NMR

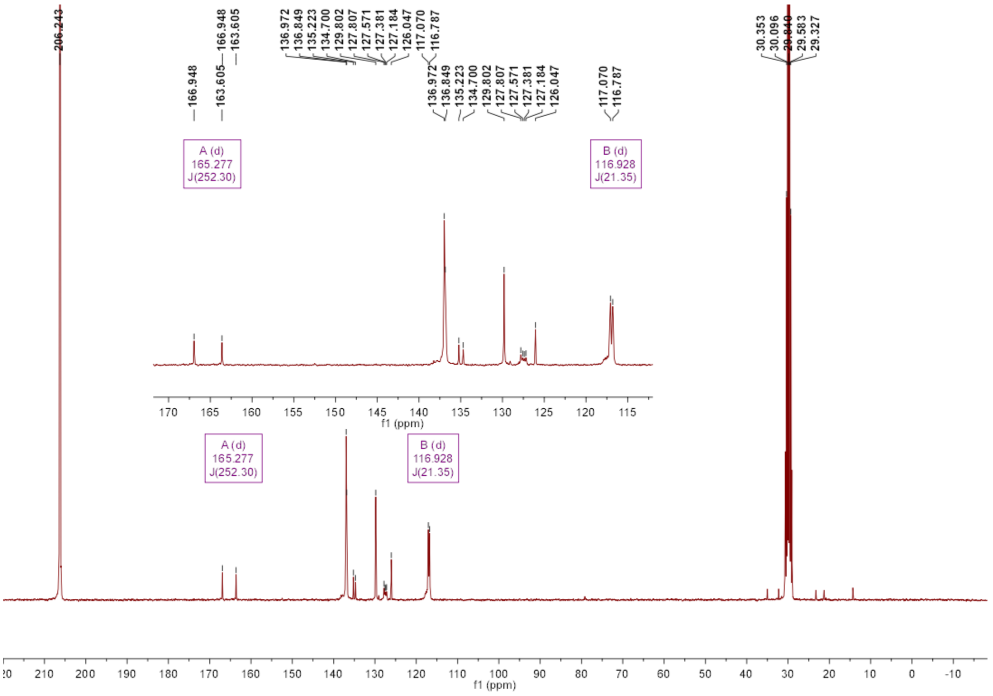

^{13}C NMR (75 MHz, CD_3COCD_3): δ 165.3 (d, J = 252.3 Hz, C4), 137.0 (br s, C2), 136.8 (s, C6), 135.2 (C8), 134.7 (C5), 129.8 (C7), 127.2-127.8 (br, C1), 116.9 (d, J = 21.4 Hz, C3).

^{31}P NMR

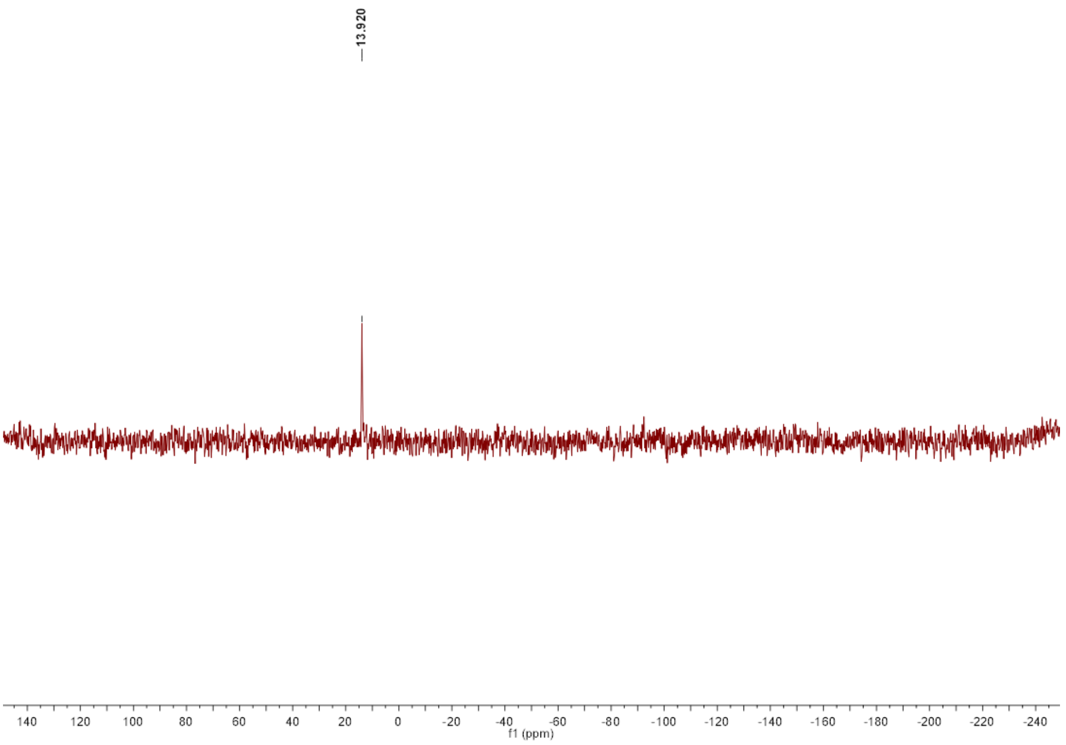

^{31}P NMR (202 MHz, CD_3COCD_3,): δ 13.92 (s, $P(C_6H_4F)_3$).

^{19}F NMR

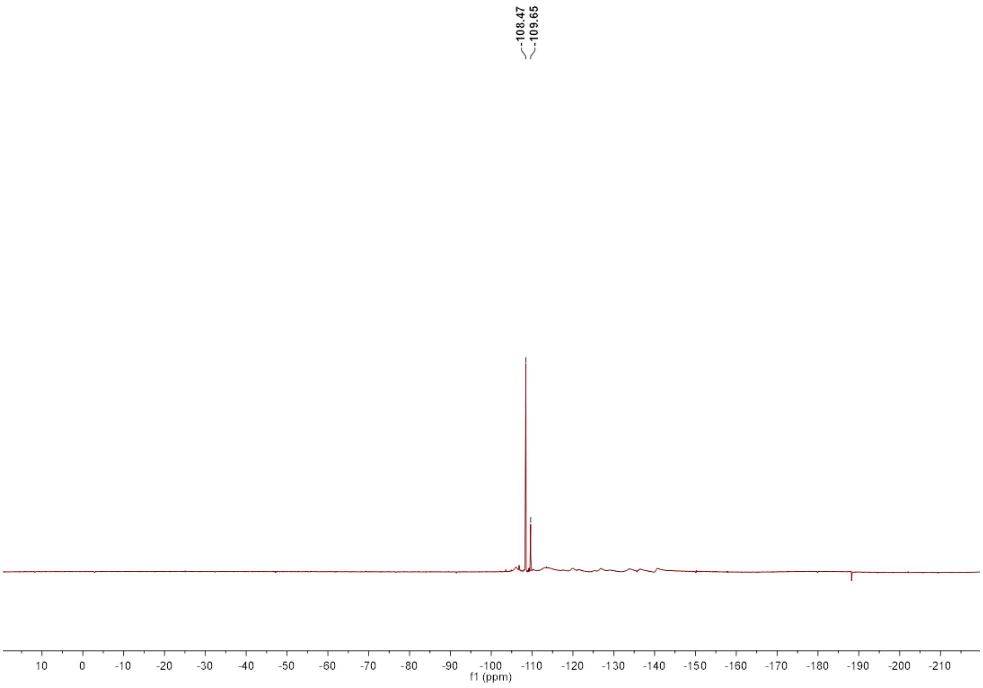

^{19}F NMR (282 MHz, CD_3COCD_3): δ -108.47 (s, $P(C_6H_4F)_3$), -109.65 (s, SbF_6^-),.

IR

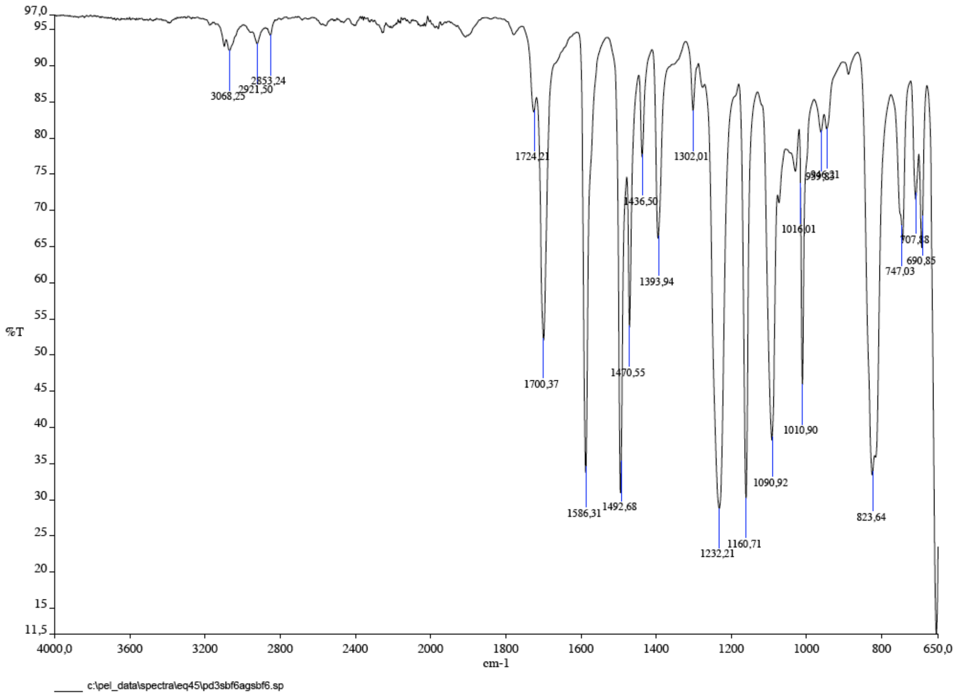

IR (cm^{-1}): ν 3068, 2922, 2853, 1724, 1700, 1586, 1493, 1470, 1436, 1394, 1302, 1232, 1161, 1091, 1011, 823, 747, 707, 691.

UV-vis.

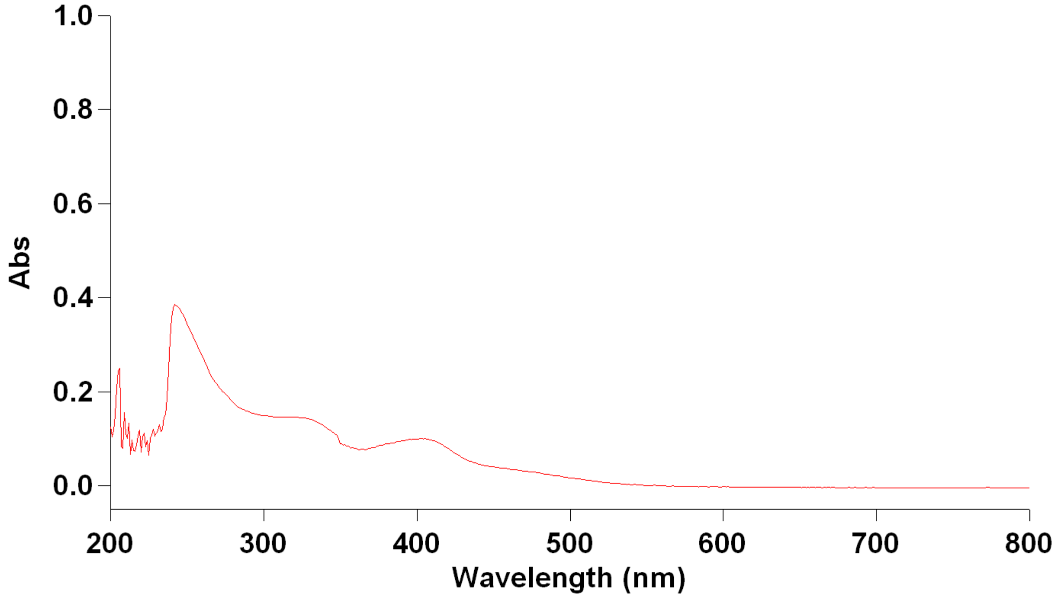

UV-vis.: c = 5 × 10^{-6} mol/L in $CHCl_3$, λ_{max} = 242 nm, ε_{max} = 7.8 × 10^4 M^{-1} cm^{-1}.

4.7. Spectra of **2**-BF_4

HRMS

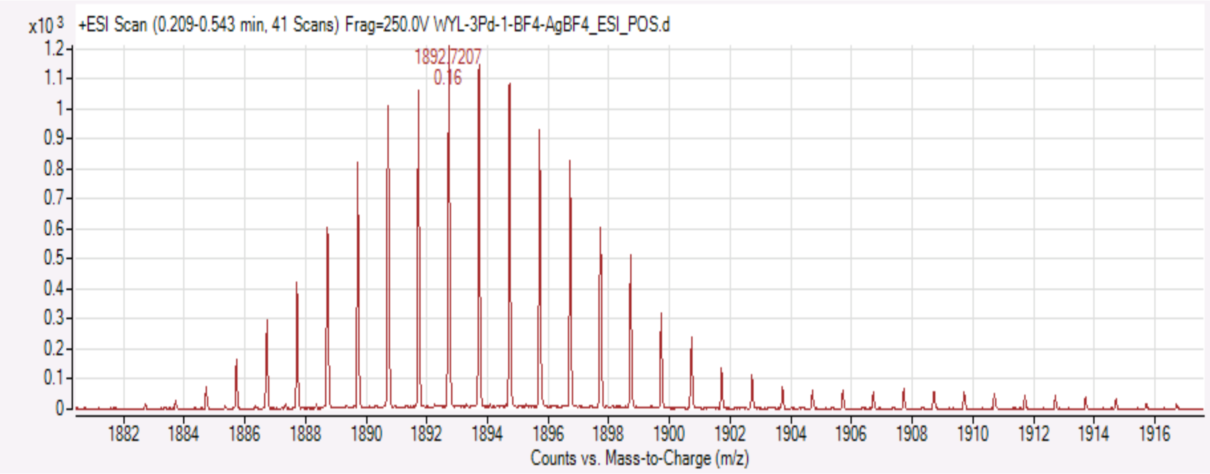

HRMS calculated for $C_{72}H_{48}Cl_3F_9P_3Pd_3S_3AgBF_4^+$ 1891.7261, found 1891.7207.

^{1}H NMR

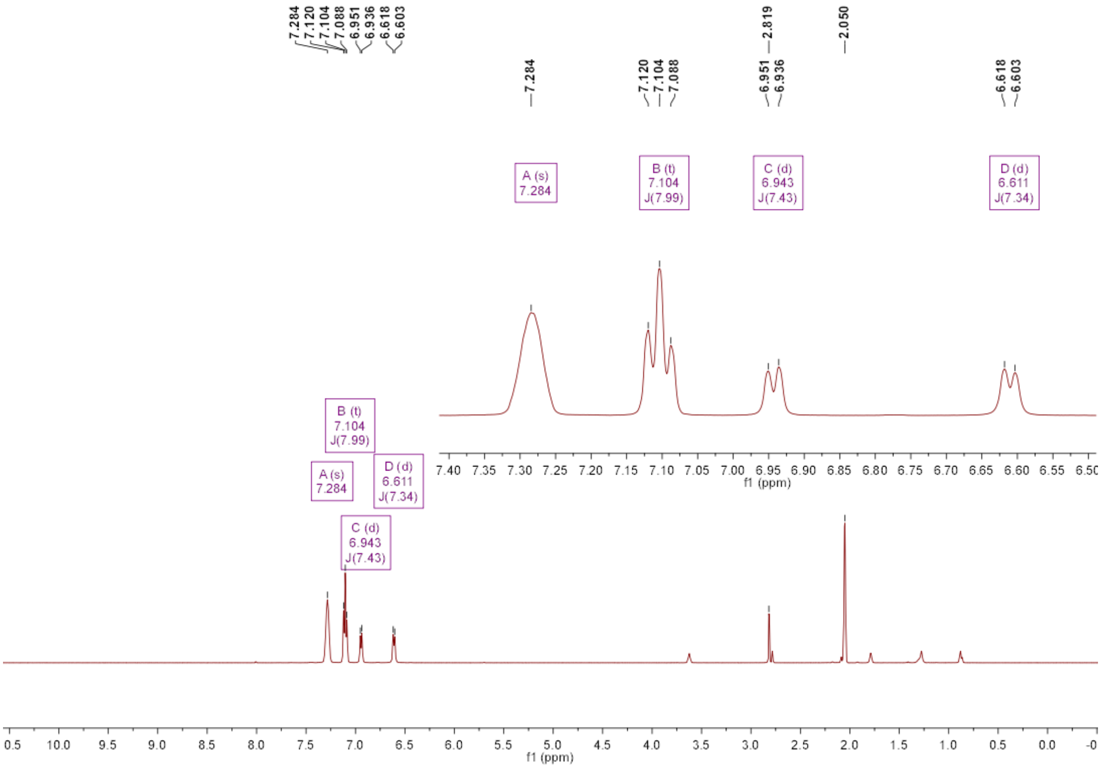

^{1}H NMR (500 MHz, CD_3COCD_3): δ 7.28 (br s, 18H, H2), 7.10 (t, J = 8.0 Hz, 18H, H3), 6.94 (d, J = 7.4 Hz, 6H, H7), 6.61 (d, J = 7.3 Hz, 6H, H6); 2.82 (s, H_2O).

^{13}C NMR

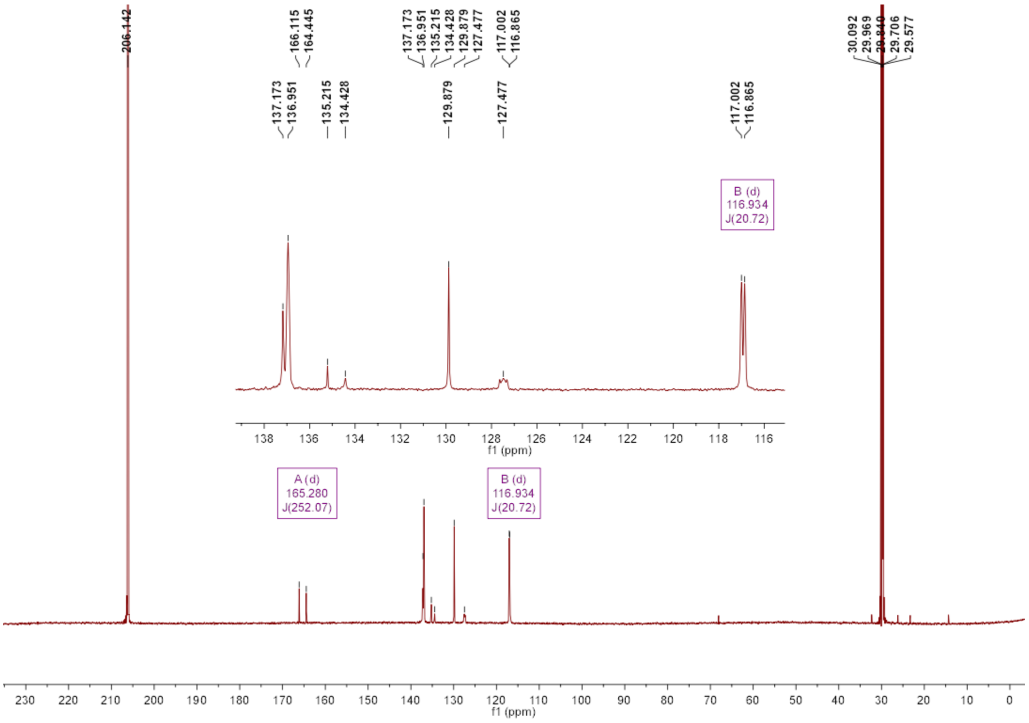

^{13}C NMR (125 MHz, CD_3COCD_3): δ 165.3 (d, J = 252.1 Hz, C4), 137.2 (br s, C2), 137.0 (s, C6), 135.2 (C8), 134.4 (C5), 129.9 (C7), 127.5 (br s, C1), 116.9 (d, J = 20.7 Hz, C3).

^{31}P NMR

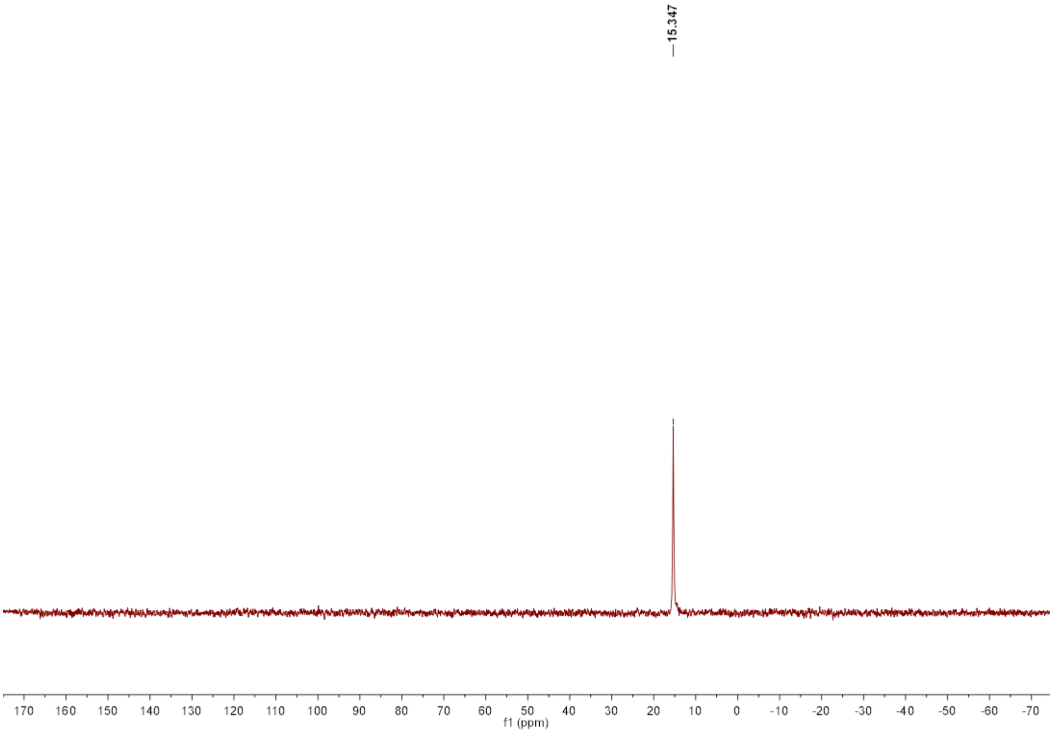

^{31}P NMR (202 MHz, CD_3COCD_3,): δ 15.35 (s, $P(C_6H_4F)_3$).

^{19}F NMR

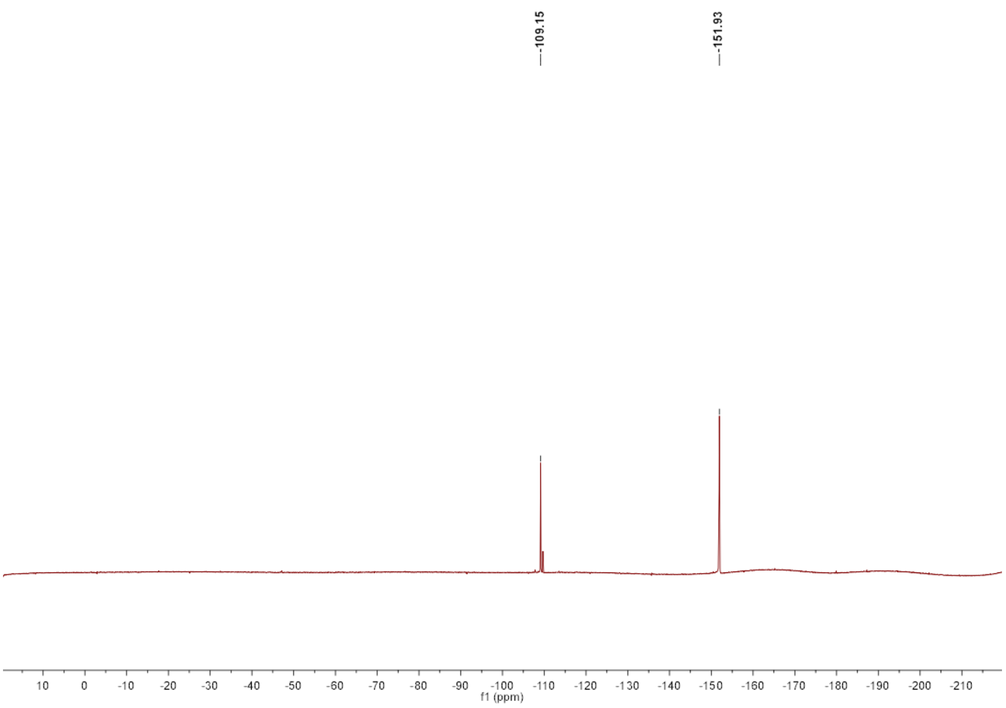

^{19}F NMR (282 MHz, CD_3COCD_3): δ -109.15 (s, $P(C_6H_4F)_3$), -151.93 (s, BF_4^-).

IR

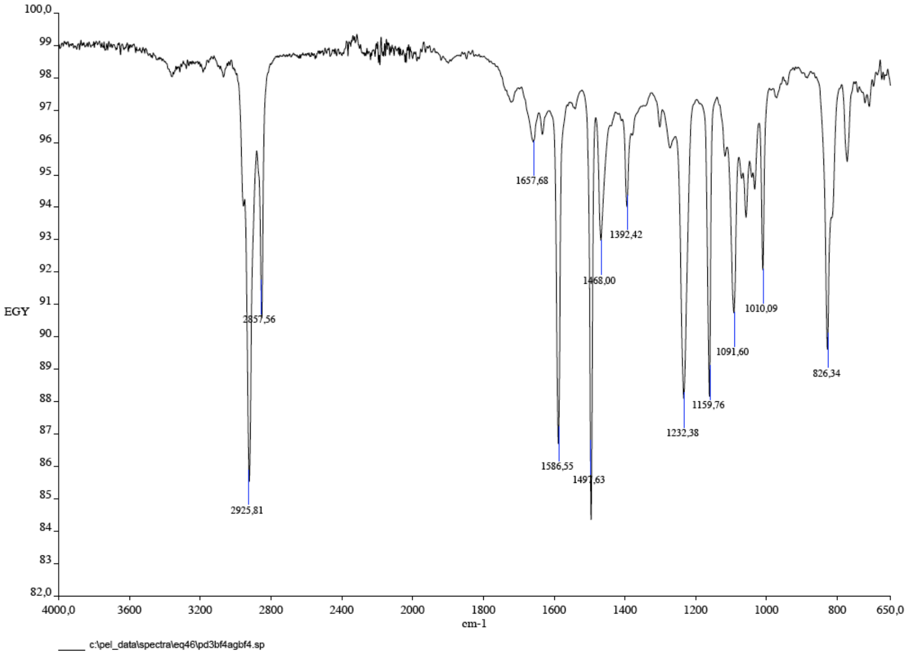

IR (cm^{-1}): ν 2955, 2926, 2857, 1657, 1587, 1498, 1468, 1392, 1232, 1160, 1092, 1011, 826.

UV-vis.

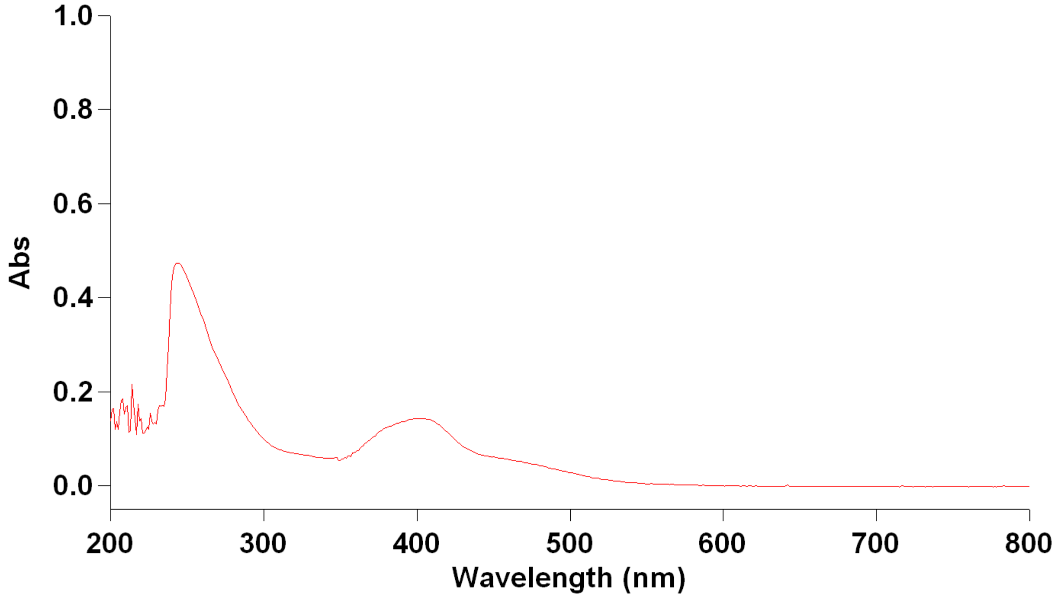

UV-vis.: c = 5 × 10^{-6} mol/L in $CHCl_3$, λ_{max} = 245 nm, ε_{max} = 9.4 × 10^4 M^{-1} cm^{-1}.

4.8. Spectra of **2**-CF_3CO_2

HRMS

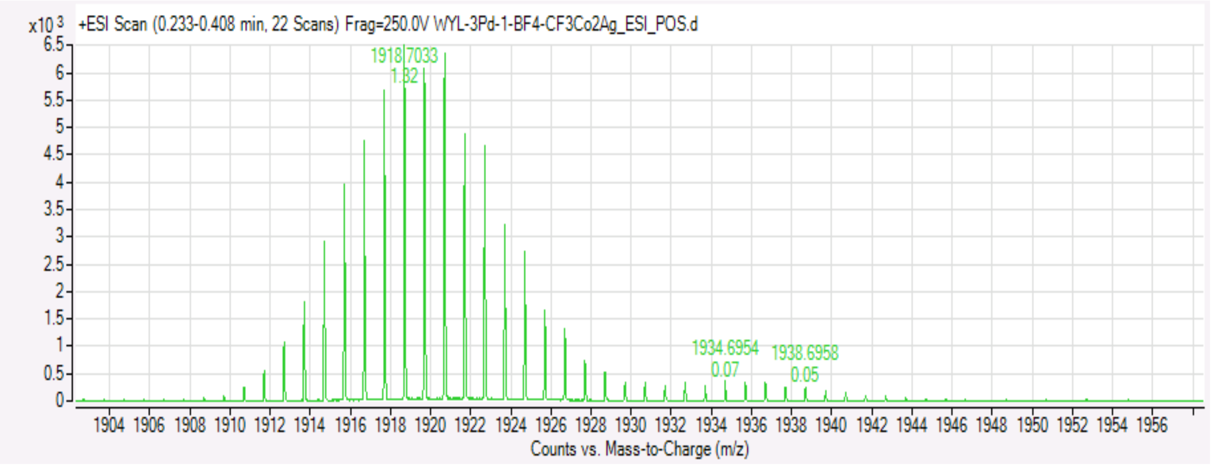

HRMS calculated for $C_{72}H_{48}Cl_3F_9P_3Pd_3S_3CF_3CO_2Ag^+$ 1918.7072, found 1918.7033.

^1H NMR

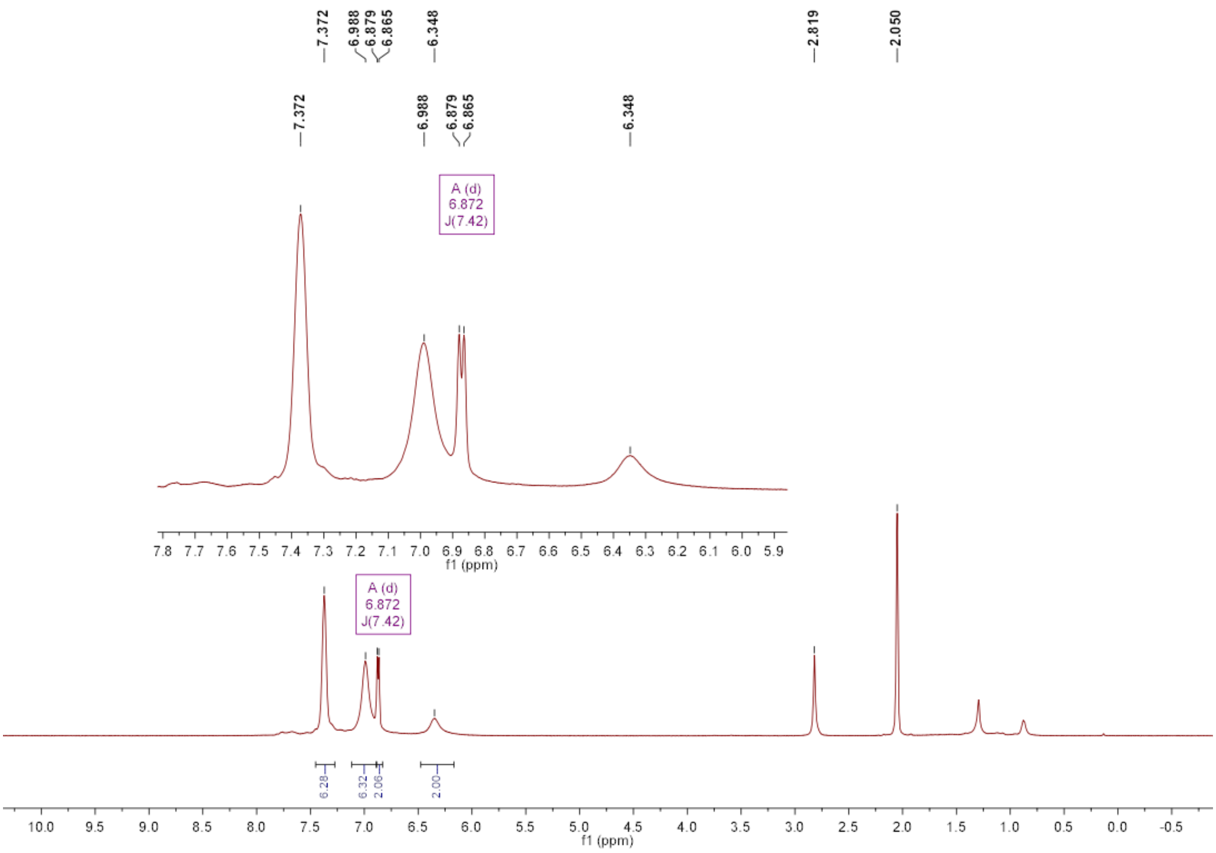

^1H NMR (500 MHz, CD_3COCD_3): δ 7.37 (br s, 18H, H2), 6.99 (br, 18H, H3), 6.87 (d, J = 7.4 Hz, 6H, H7), 6.35 (br, 6H, H6); 2.82 (s, H_2O).

^{13}C NMR

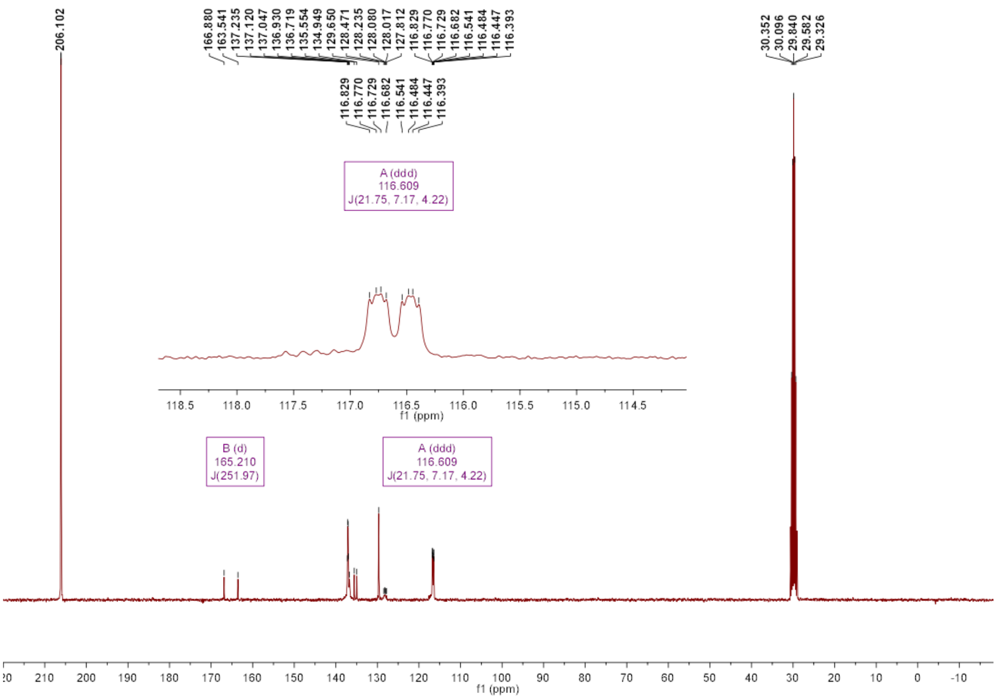

^{13}C NMR (75 MHz, CD_3COCD_3): δ 165.2 (d, J = 252.0 Hz, C4), 136.9-137.2 (br, C2), 137.0 (C6), 135.6 (C8), 134.9 (C5), 129.7 (C7), 127.8-128.5 (br, C1), 116.8 (ddd, J = 22.8, 7.2, 4.0 Hz, C3).

^{31}P NMR

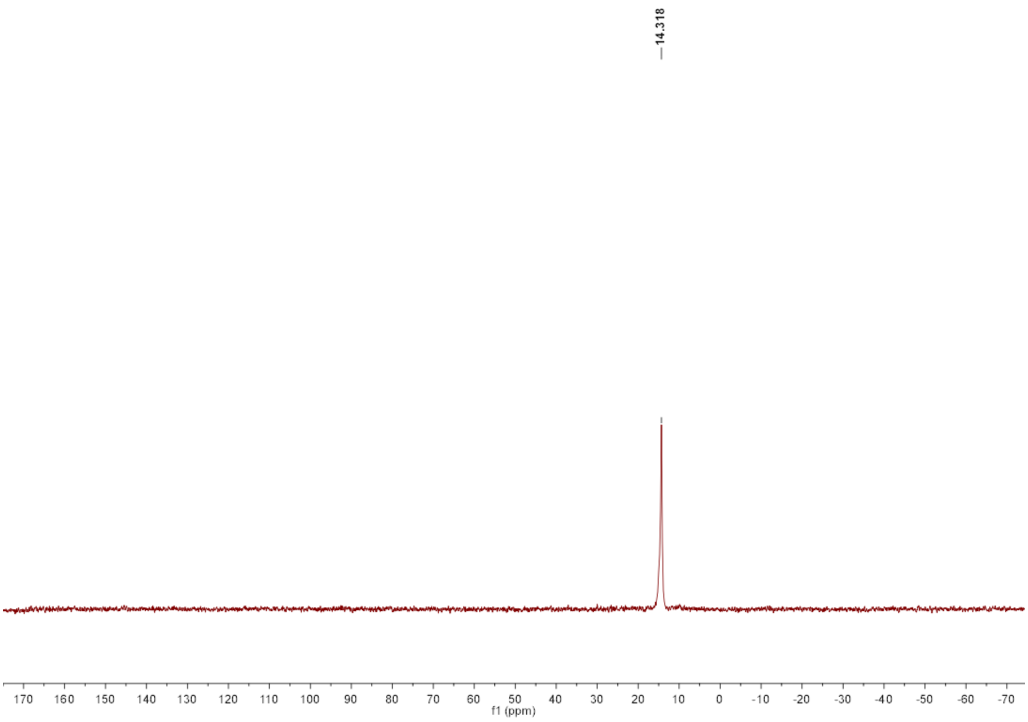

^{31}P NMR (202 MHz, CD_3COCD_3): δ 14.32 (s, $P(C_6H_4F)_3$).

^{19}F NMR

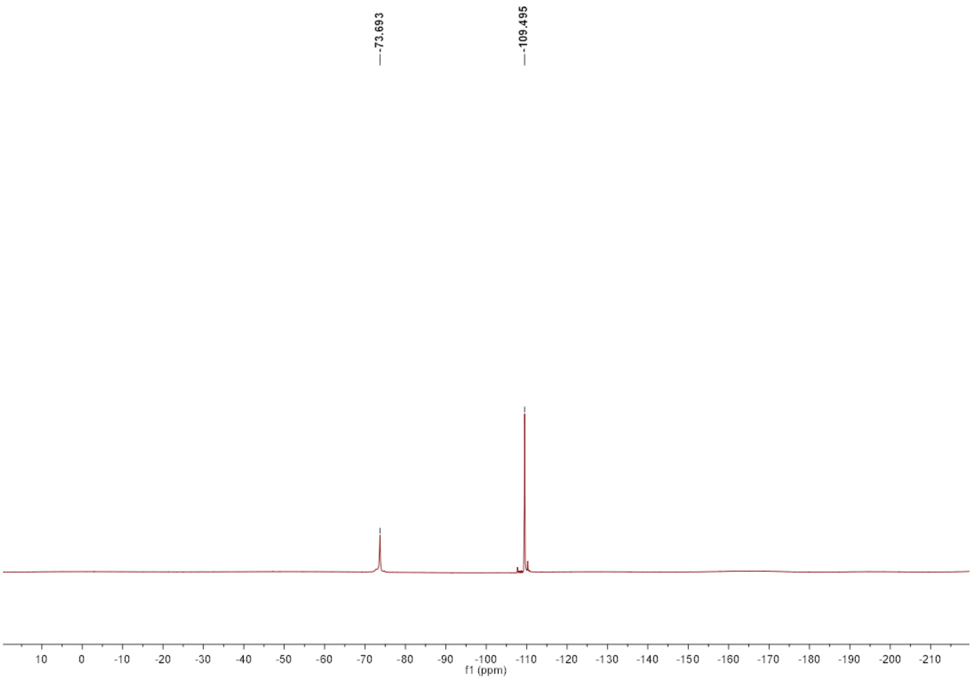

^{19}F NMR (282 MHz, CD_3COCD_3): δ -109.50 (s, $P(C_6H_4F)_3$), -73.69 (s, $CF_3CO_2^-$).

IR

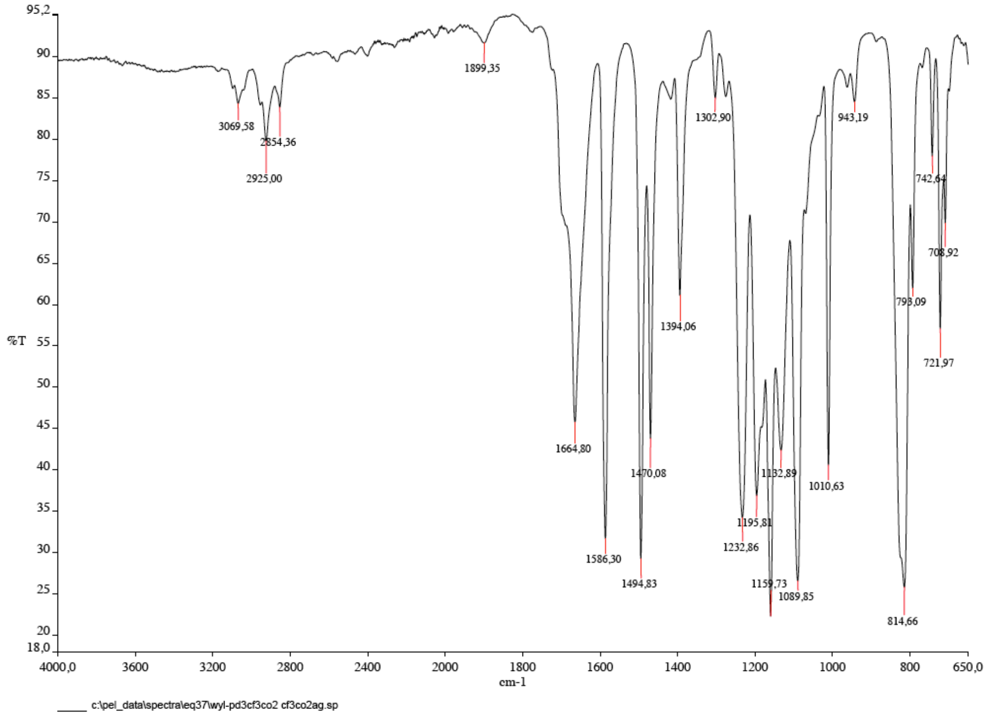

IR (cm^{-1}): ν 3069, 2926, 2854, 1899, 1664, 1586, 1495, 1470, 1394, 1237, 1195, 1160, 1089, 1010, 814, 798, 721.

UV-vis.

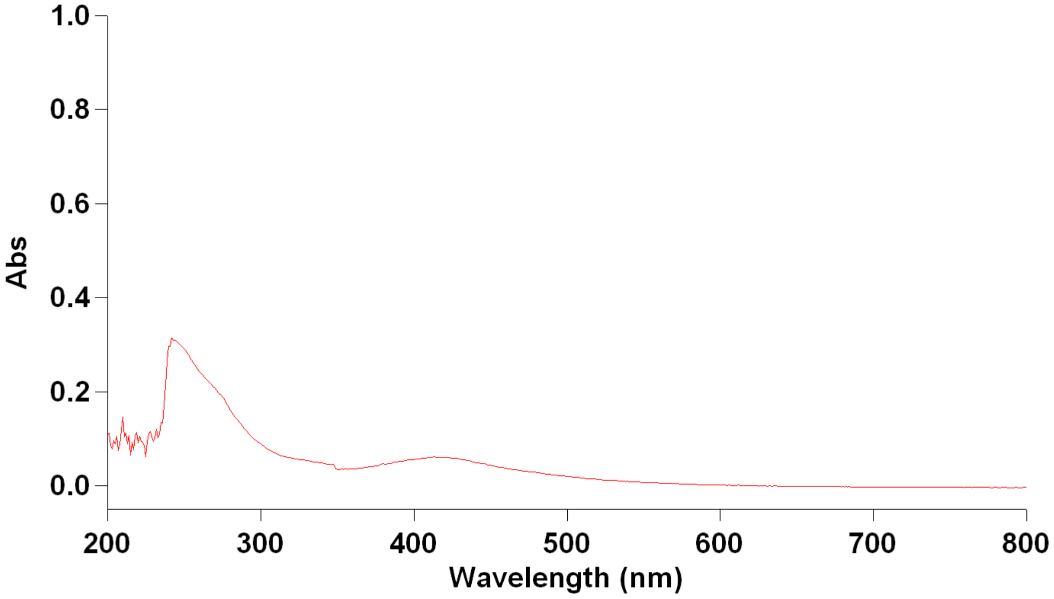

UV-vis.: c = 5 × 10^{-6} mol/L in $CHCl_3$, λ_{max} = 242 nm, ε_{max} = 6.2 × 10^4 M^{-1} cm^{-1}.

4.9. Spectra of of 1-SbF_6-PPh_3 + PPh_3AuCl (1 equiv.)

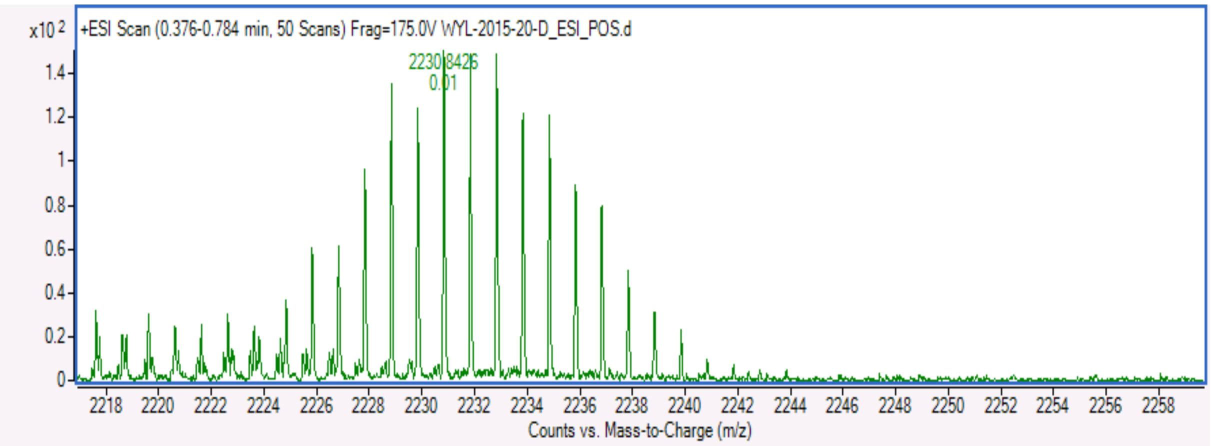

HRMS, calculated for $C_{90}H_{72}Cl_3F_6P_4Pd_3S_3SbAu^+$ 2230.8547, found 2230.8426.

^{1}H NMR

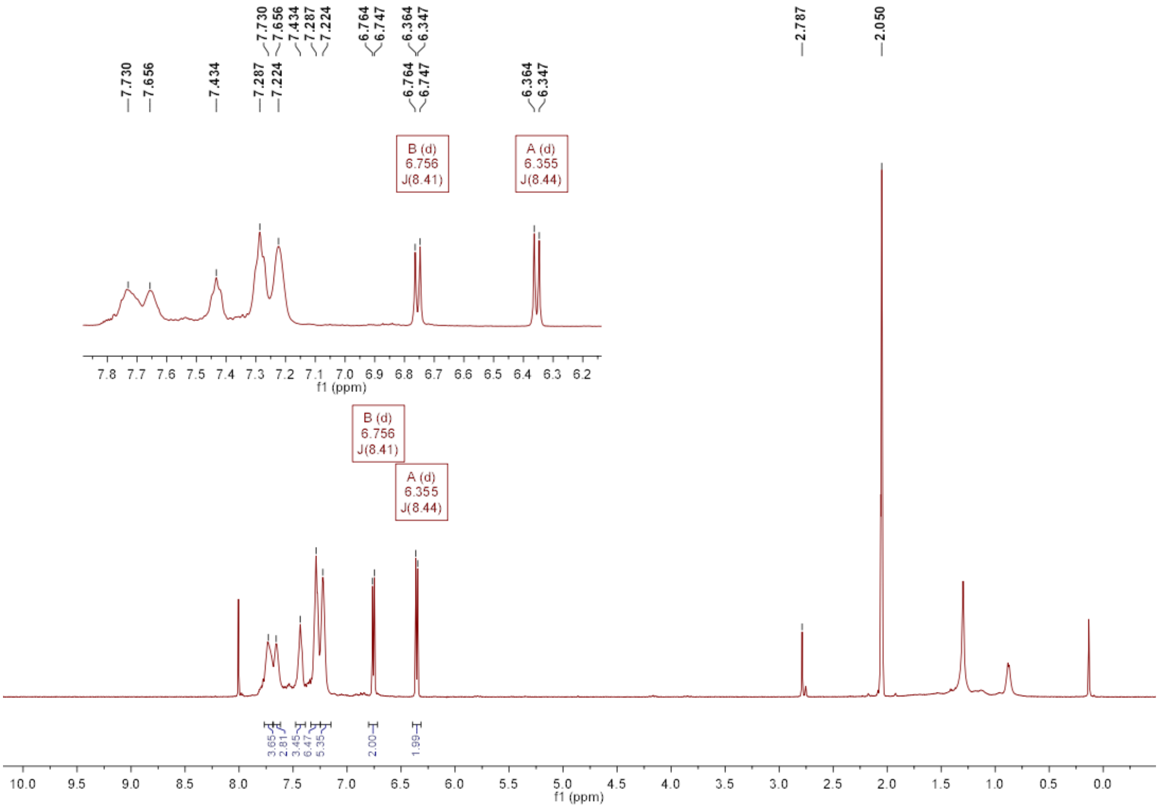

^{1}H NMR (500 MHz, CD_3COCD_3): δ 7.66-7.73 (br, 15H, Ph/AuPPh$_3^+$), 7.43 (br s, 9H, H4), 7.29 (br s, 18H, H3), 7.22 (br s, 18H, H2), 6.76 (d, J = 8.4 Hz, 6H, H7), 6.36 (d, J = 8.4 Hz, 6H, H6); 2.79 (s, H_2O).

^{31}P NMR

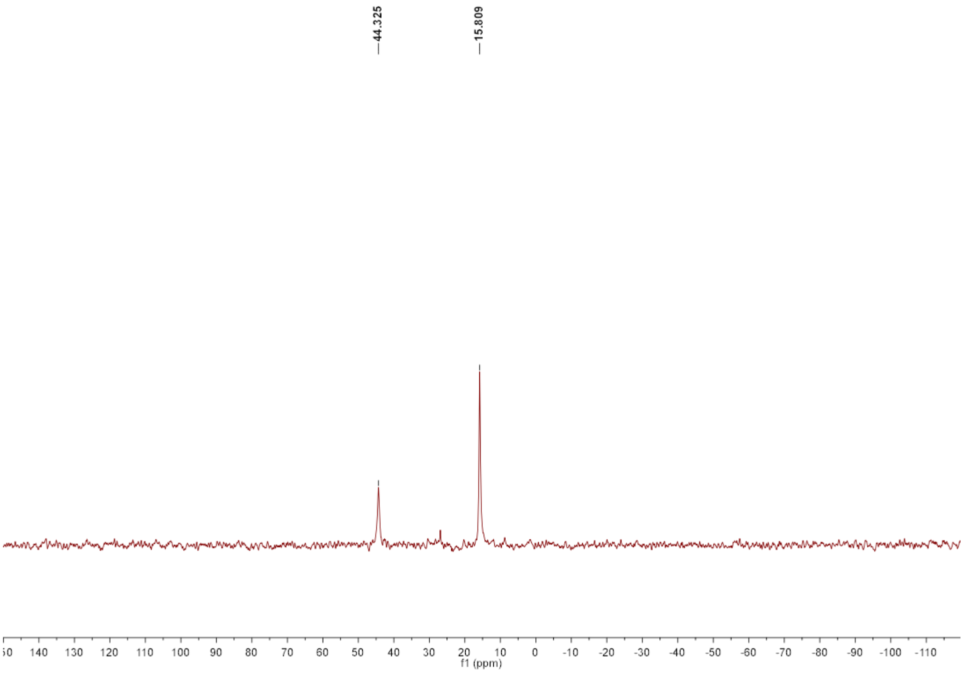

^{31}P NMR (202 MHz, CD_3COCD_3,): δ 15.81 (s, PPh_3), 44.33 (s, $AuPPh_3^+$).

^{13}C NMR

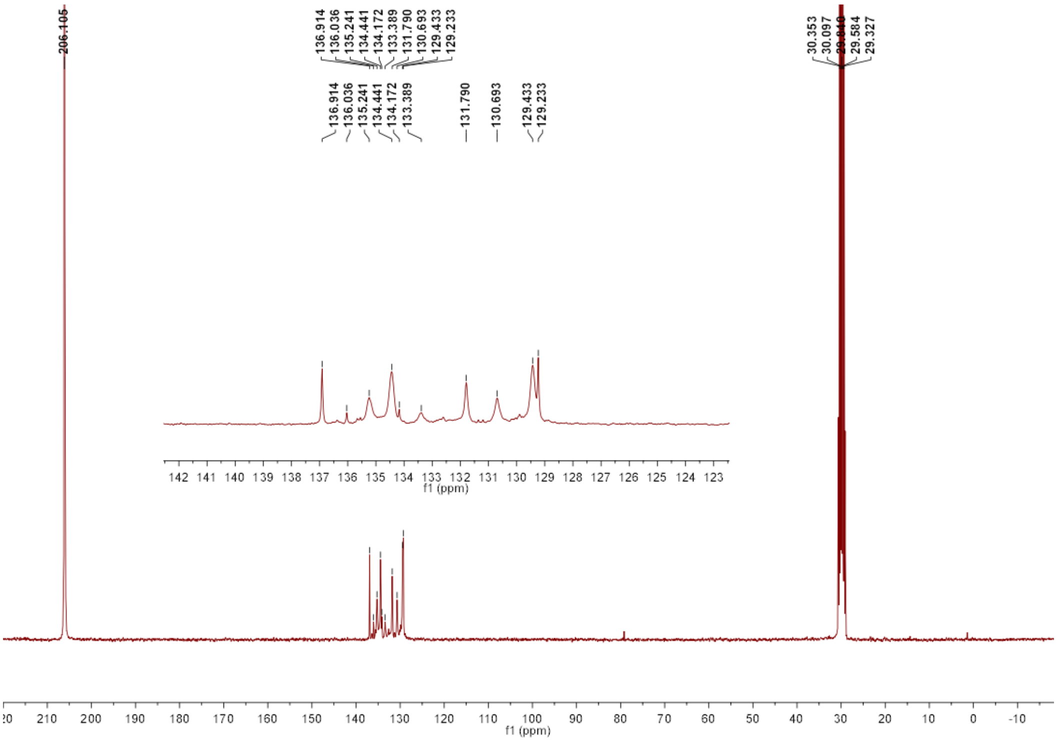

^{13}C NMR (75 MHz, CD_3COCD_3): δ 136.9 (s, C6), 136.0 (C8), 135.2 (Ph/$AuPPh_3^+$), 134.4 (br s, C3), 134.2 (C5), 133.4 (br s, Ph/$AuPPh_3^+$), 131.8 (br s, C4), 130.7 (Ph/$AuPPh_3^+$), 129.4 (br, C1+C2), 129.2 (C7).

IR

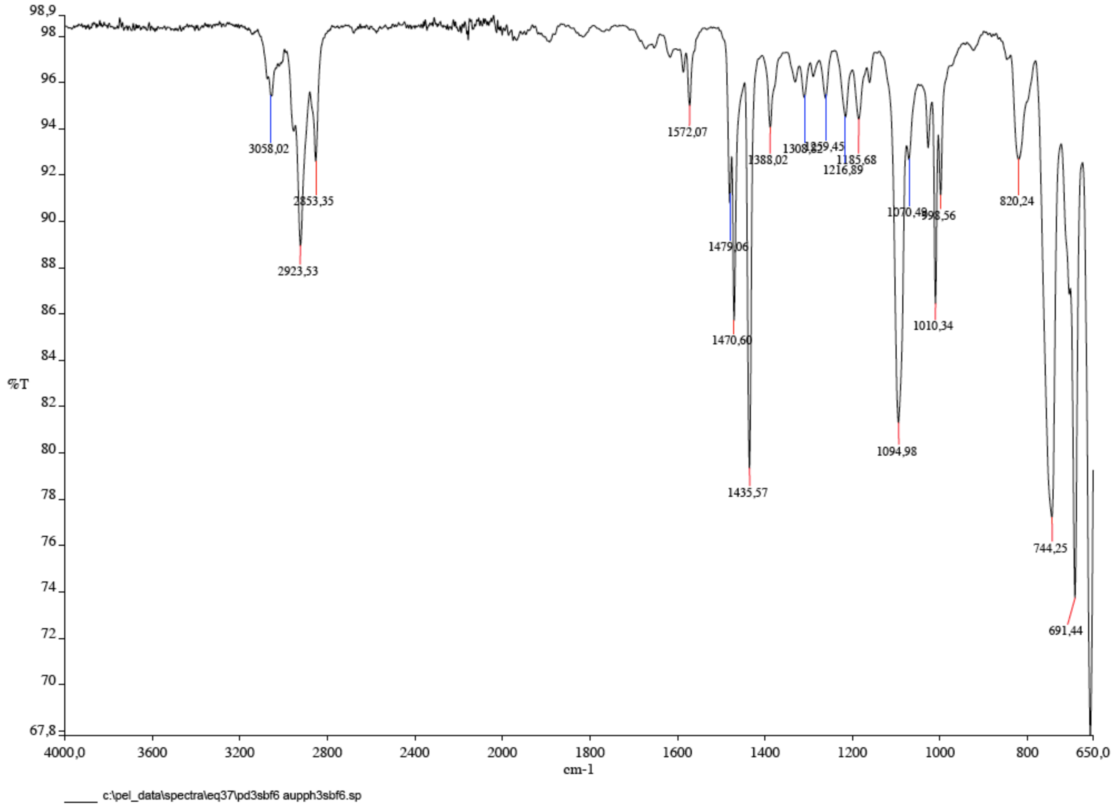

IR (cm^{-1}): ν 3058, 2923, 2853, 1572, 1479, 1470, 1435, 1388, 1308, 1216, 1185, 1095, 1070, 1010, 820, 744, 691.

UV-vis.

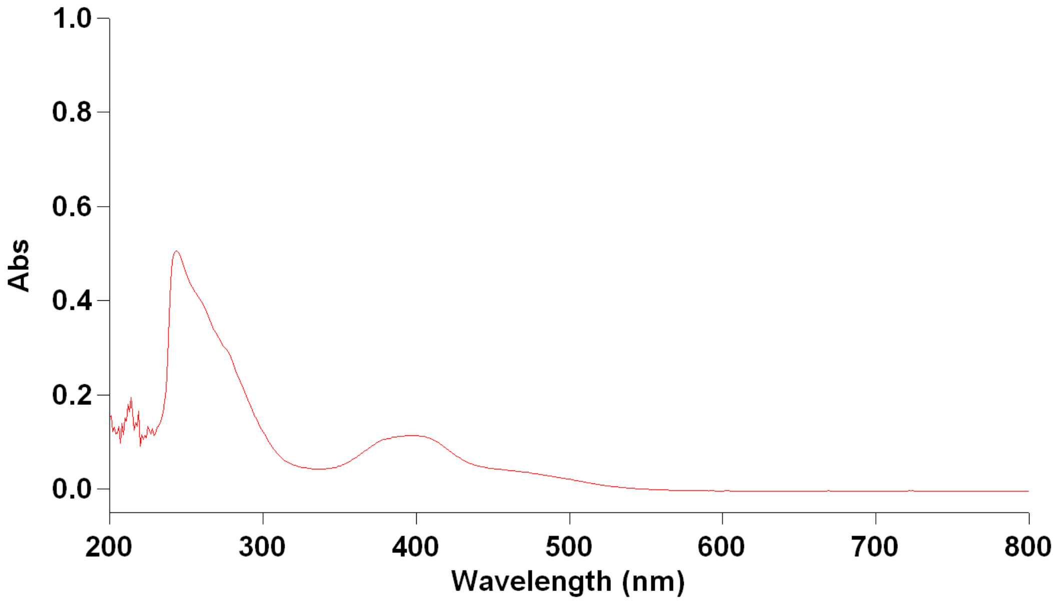

UV-vis.: $c = 1 \times 10^{-5}$ mol/L in $CHCl_3$, $\lambda_{max} = 244$ nm, $\varepsilon_{max} = 5.2 \times 10^{4}$ M^{-1} cm^{-1}.

4.10. Spectra of 1-OTf + CuOTf*Tol (4 equiv.)

HRMS

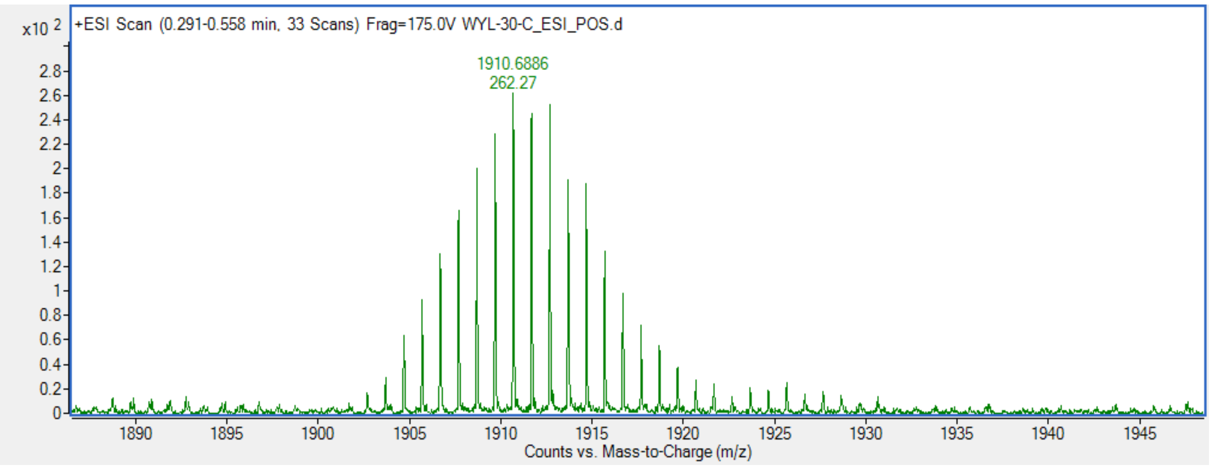

HRMS, calculated for $C_{72}H_{48}Cl_3F_9P_3Pd_3S_3CF_3SO_3Cu^+$ 1910.6981, found 1910.6886.

^{1}H NMR

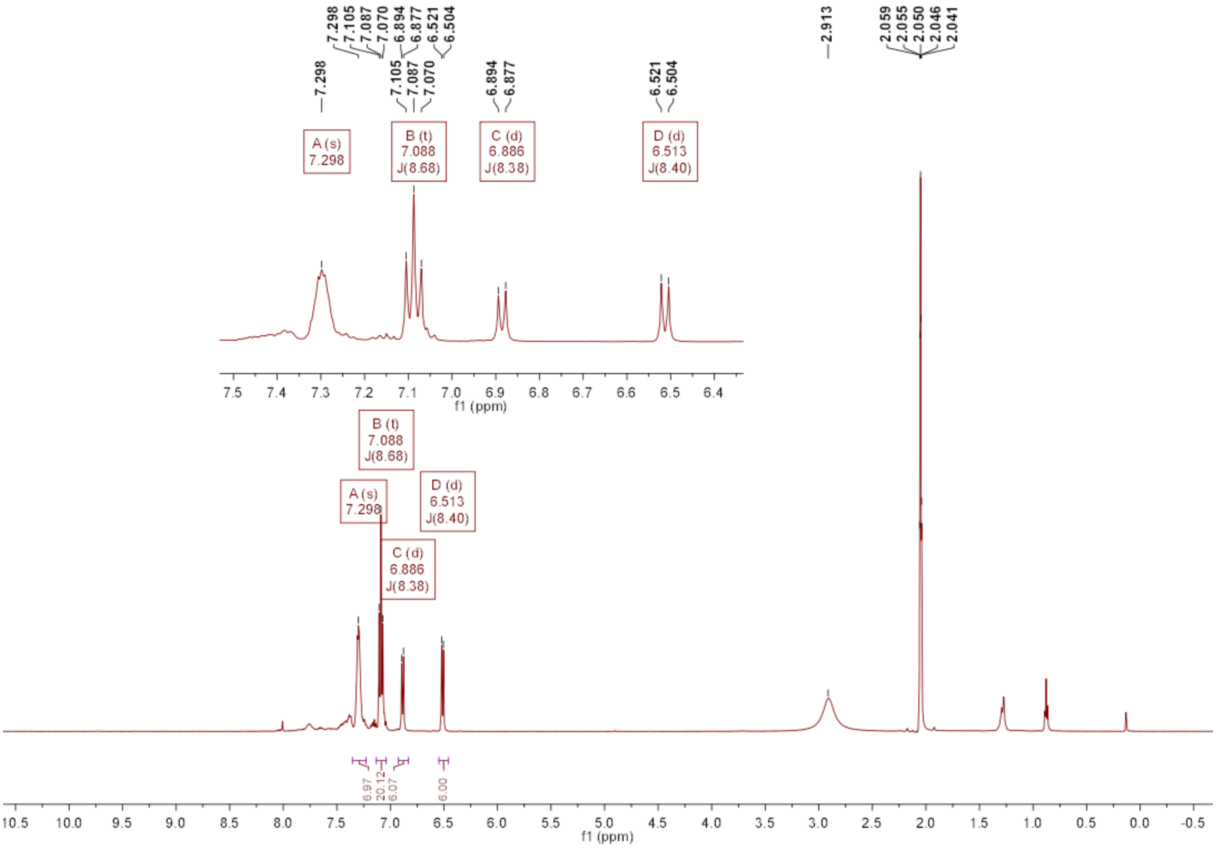

^{1}H NMR (500 MHz, CD_3COCD_3): δ 7.30 (br s, 18H, H2), 7.09 (t, J = 8.7 Hz, 18H, H3), 6.89 (d, J = 8.4 Hz, 6H, H7), 6.51 (d, J = 8.4 Hz, 6H, H6); 2.91 (s, H_2O).

^{13}C NMR

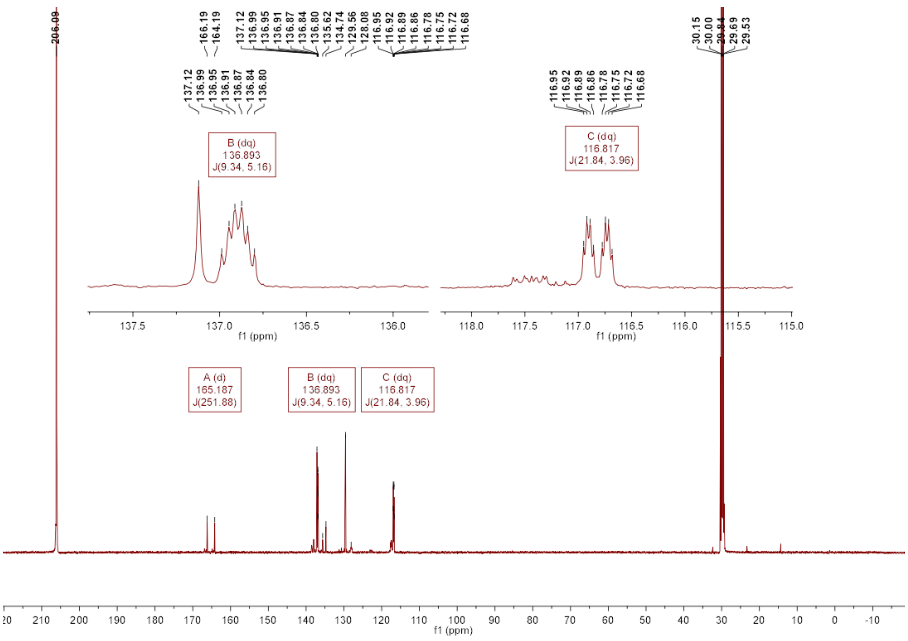

^{13}C NMR (125 MHz, CD_3COCD_3): δ 165.2 (d, J = 251.9 Hz, C4), 137.1 (br s, C2), 137.0 (dq, J = 9.3, 5.2 Hz, C6), 135.6 (C8), 134.7 (C5), 129.6 (C7), 128.1 (br s, C1), 116.8 (dq, J = 22.8, 4.0 Hz, C3).

^{31}P NMR

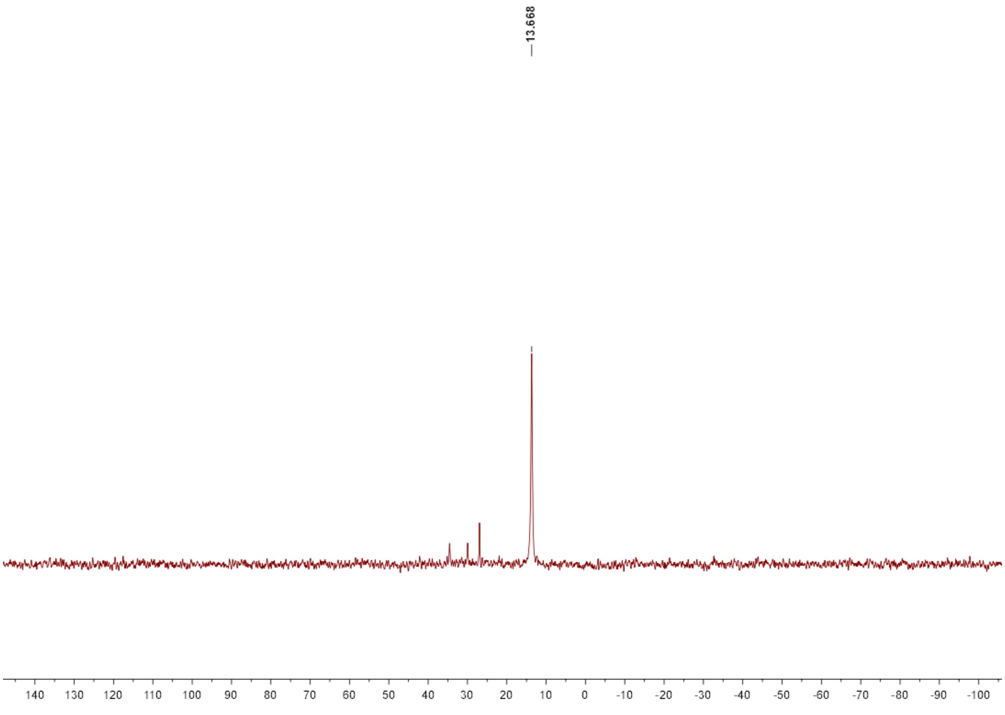

^{31}P NMR (202 MHz, CD_3COCD_3): δ 13.67 (s, $P(C_6H_4F)_3$).

^{19}F NMR

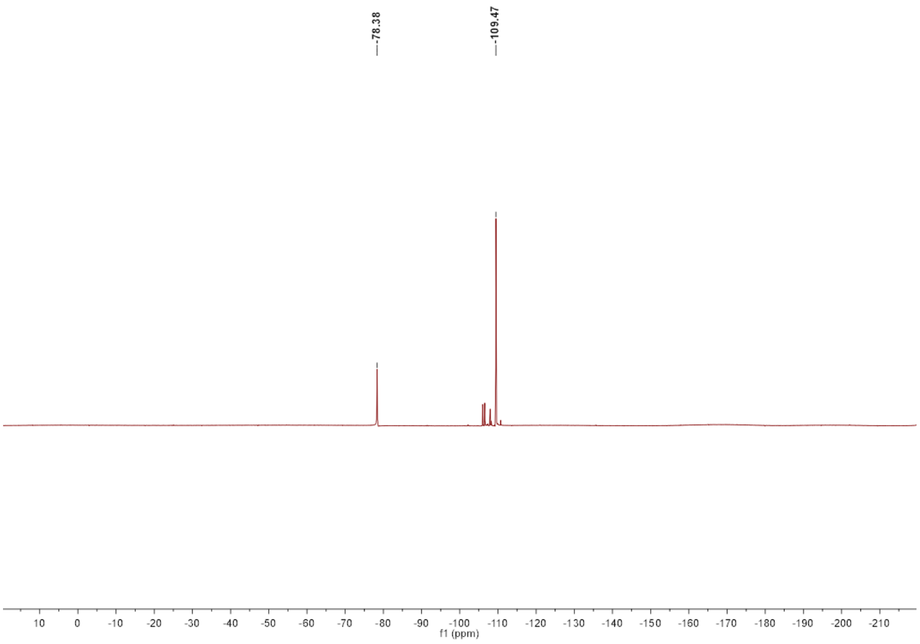

^{19}F NMR (282 MHz, CD_3COCD_3): δ-109.47 (s, $P(C_6H_4F)_3$), -78.38 (s, $(CF_3SO_3^-)$.

IR

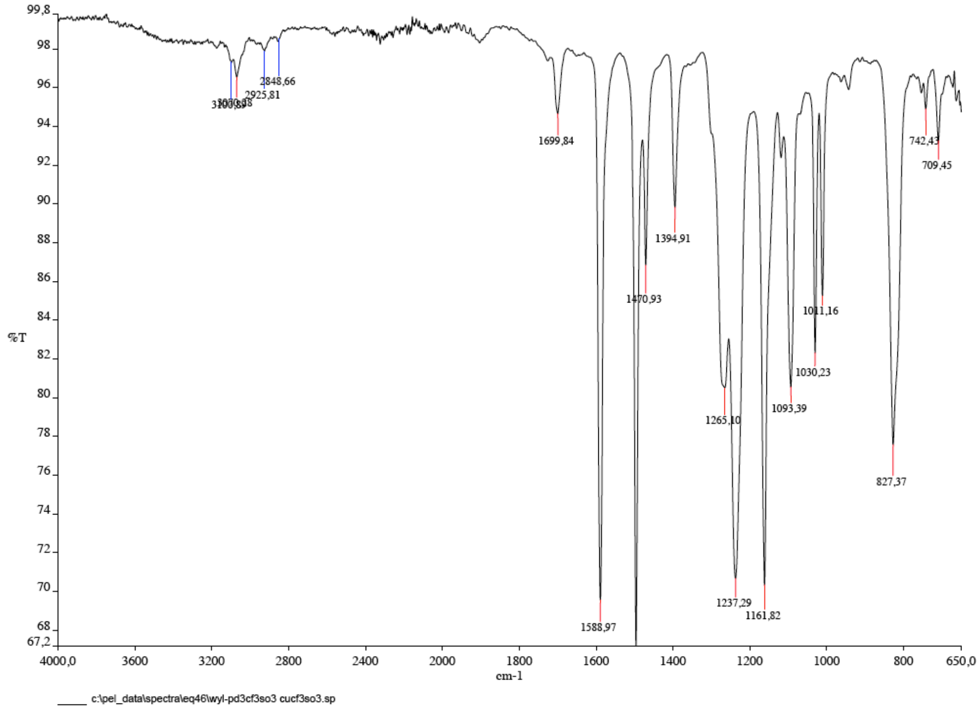

IR (cm^{-1}): ν 3100, 3070, 2925, 2845, 1699, 1589, 1497, 1470, 1395, 1265, 1237, 1161, 1093, 1030, 1011, 827.

UV-vis.

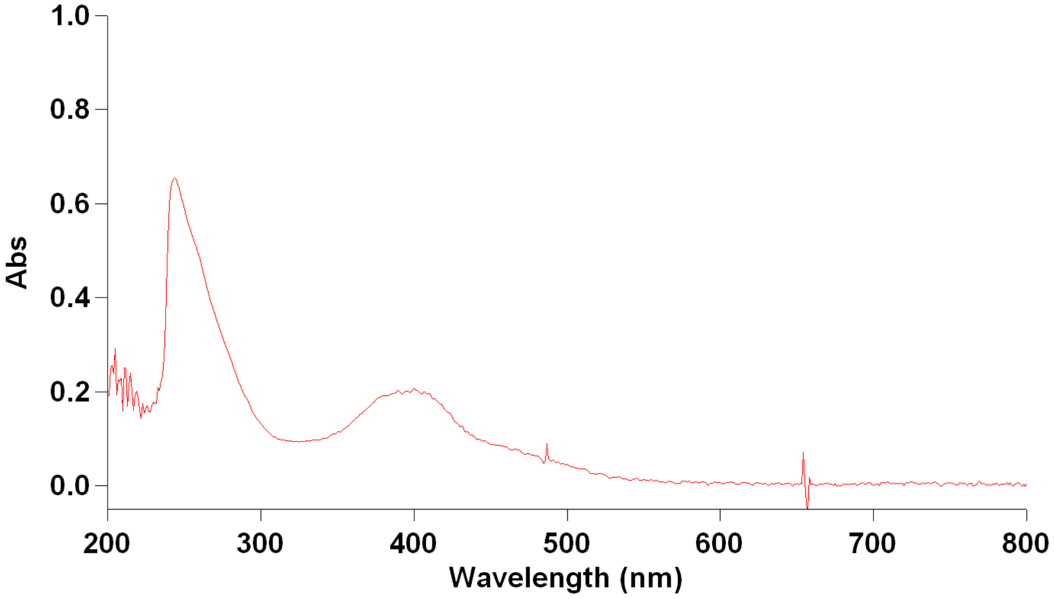

UV-vis.: c = 7.5 × 10^{-6} mol/L in $CHCl_3$, λ_{max} = 244 nm, ε_{max} = 8.7 × 10^4 M^{-1} cm^{-1}.

4.11. Spectra of 1-OTf + CF_3SO_3Li (4 and 10 equiv.)

HRMS

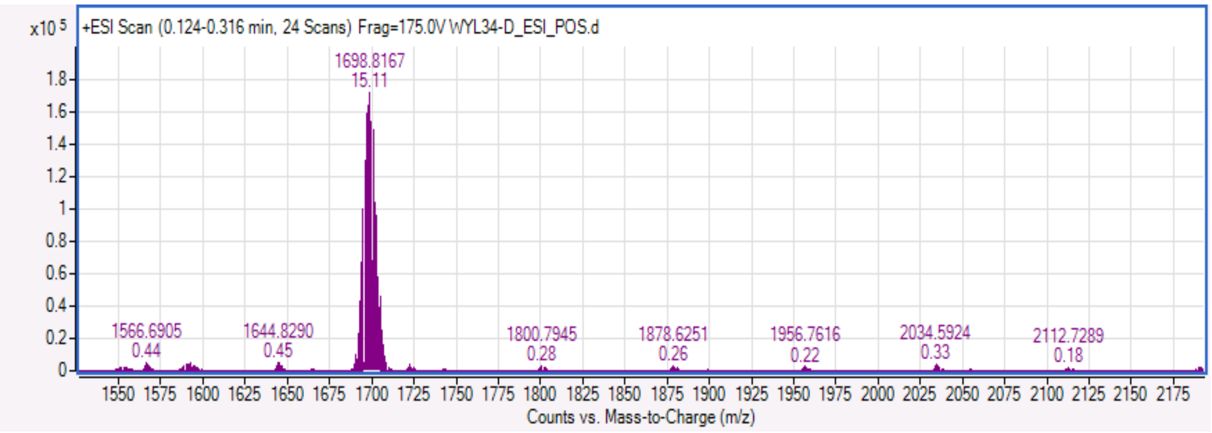

HRMS calculated for $C_{72}H_{48}Cl_3F_9P_3Pd_3S_3CF_3SO_3Li^+$ 1854.7856, found (without CF_3SO_3Li) 1698.8167.

^{1}H NMR comparison in d^6-acetone for **1-OTf** (blue line), **1** + 4 equiv. CF_3SO_3Li (red line) and **1** + 10 equiv. CF_3SO_3Li (green line).

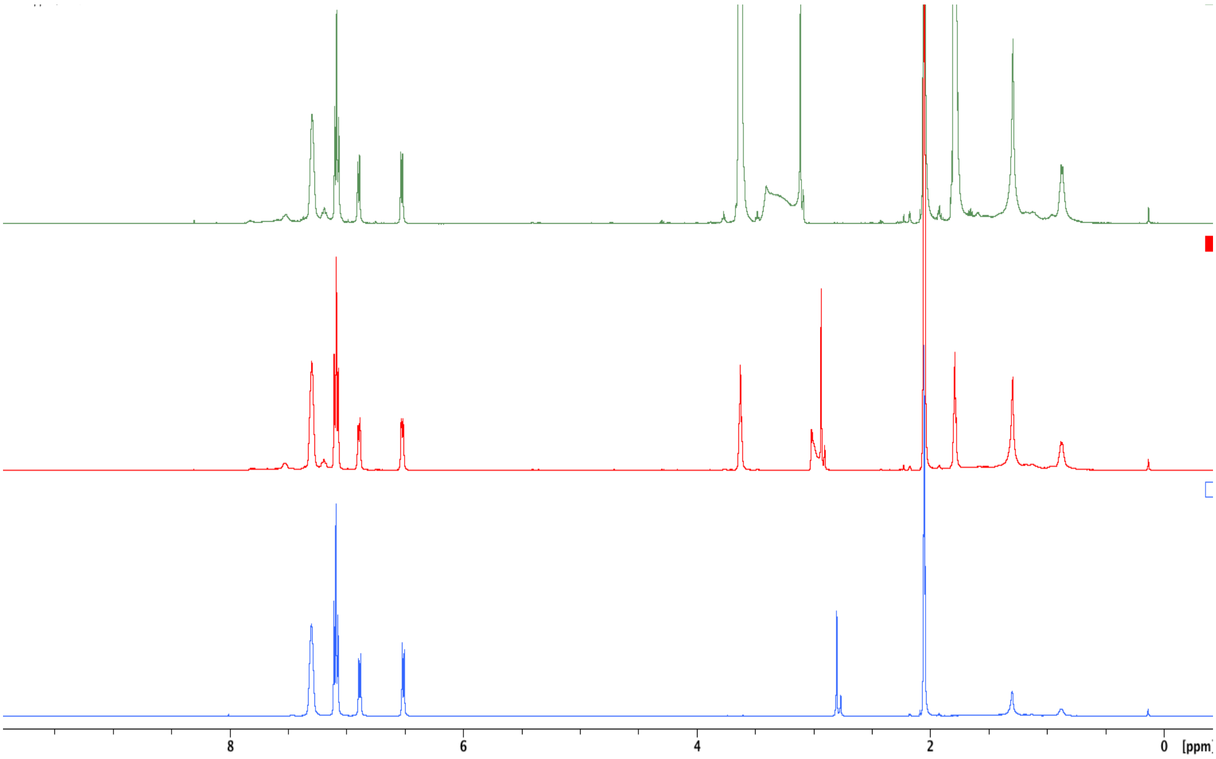

Zoom screen of compared chemical shift of ^{1}H NMR in d^{6}-acetone for the compound **1** (blue line), **1** + 4 equiv. CF_3SO_3Li (red line) and **1** + 10 equiv. CF_3SO_3Li (green line).

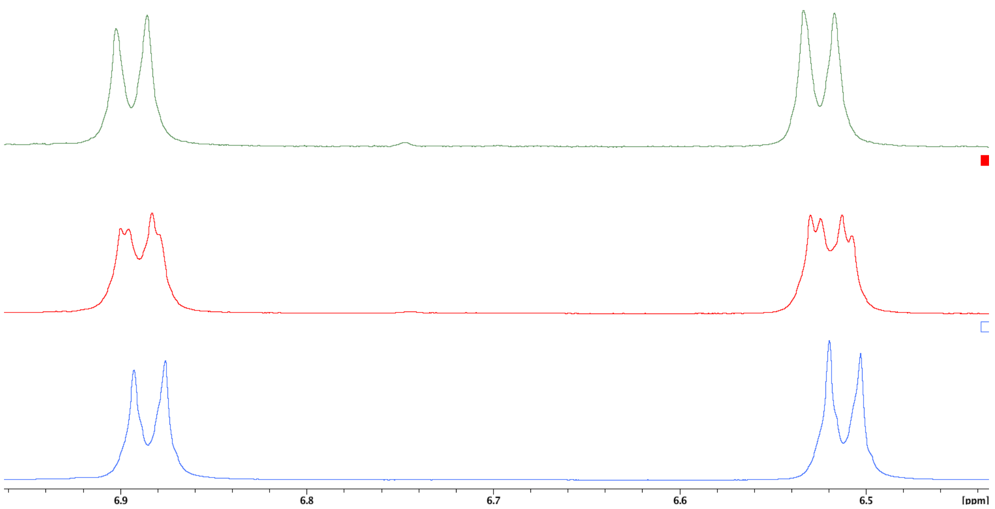

^{1}H NMR of sample (**1** + 10 equiv. CF_3SO_3Li)

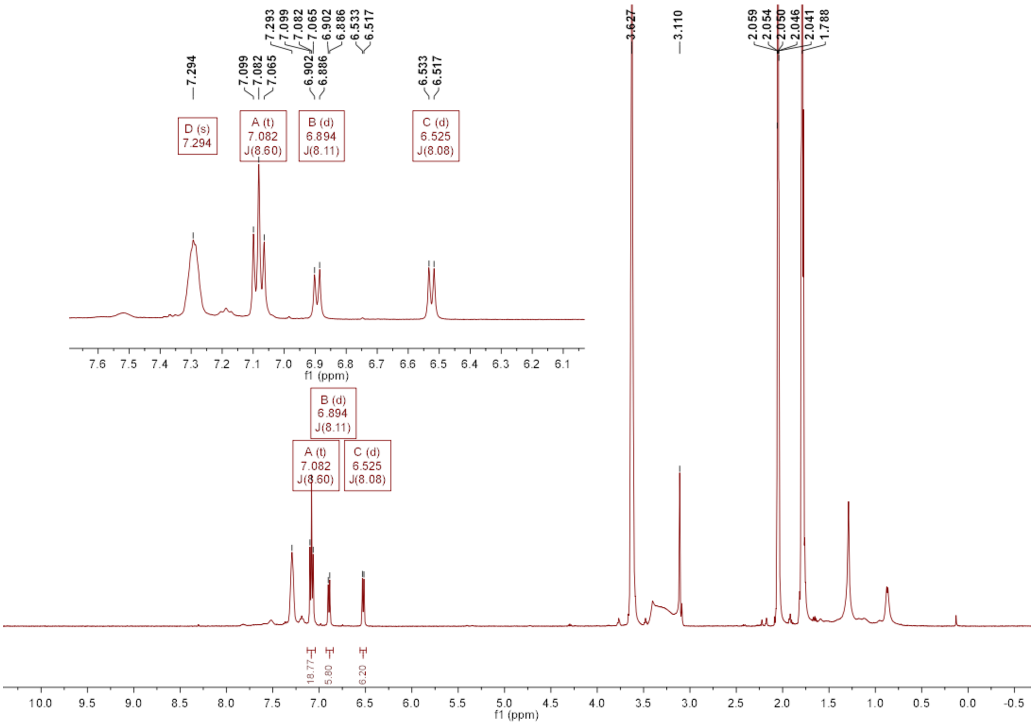

^{1}H NMR of sample (**1** + excess CF_3SO_3Li) (500 MHz, CD_3COCD_3): δ 7.29 (br s, 18H, H2), 7.08 (t, J = 8.6 Hz, 18H, H3), 6.89 (d, J = 8.1 Hz, 6H, H7), 6.53 (d, J = 8.1 Hz, 6H, H6); 3.11 (s, H_2O), 1.79, 3.63 (m, THF).

^{13}C NMR of sample (**1** + 10 equiv. CF_3SO_3Li)

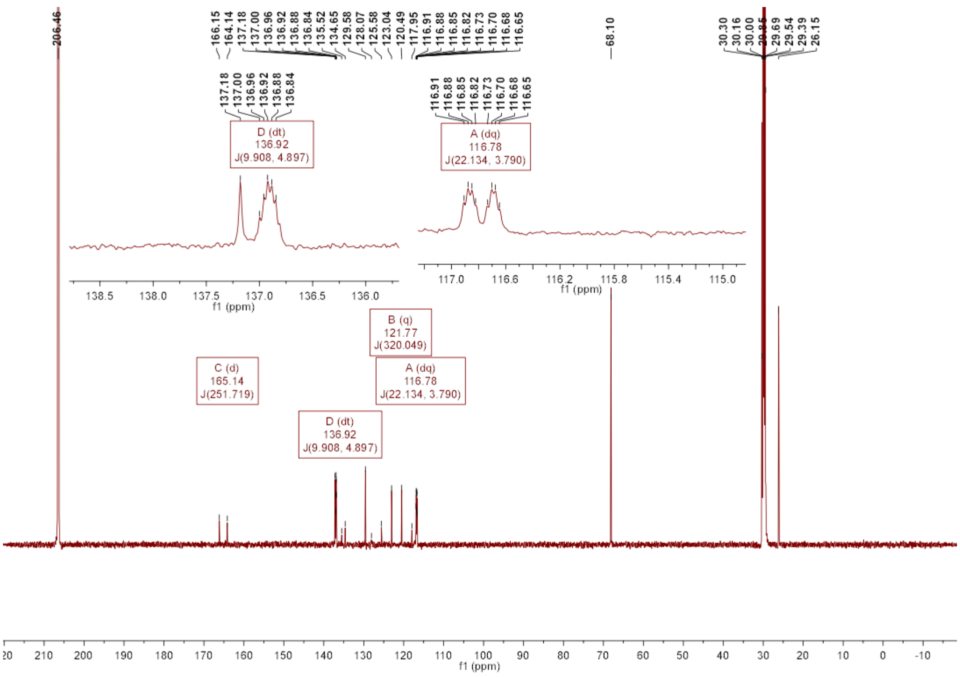

^{13}C NMR of (**1** + excess CF_3SO_3Li) (125 MHz, CD_3COCD_3): δ 165.1 (d, J = 251.9 Hz, C4), 137.2 (br s, C2), 136.9 (dt, J = 9.9, 4.9 Hz, C6), 135.5 (C8), 134.7 (C5), 129.6 (C7), 128.1 (br s, C1), 121.8 (q, J = 320.0 Hz, C/$CF_3SO_3^-$), 116.8 (dq, J = 22.1, 3.8 Hz, C3); 26.15, 68.10 (THF).

^{31}P NMR of sample **(1** + 10 equiv. CF_3SO_3Li)

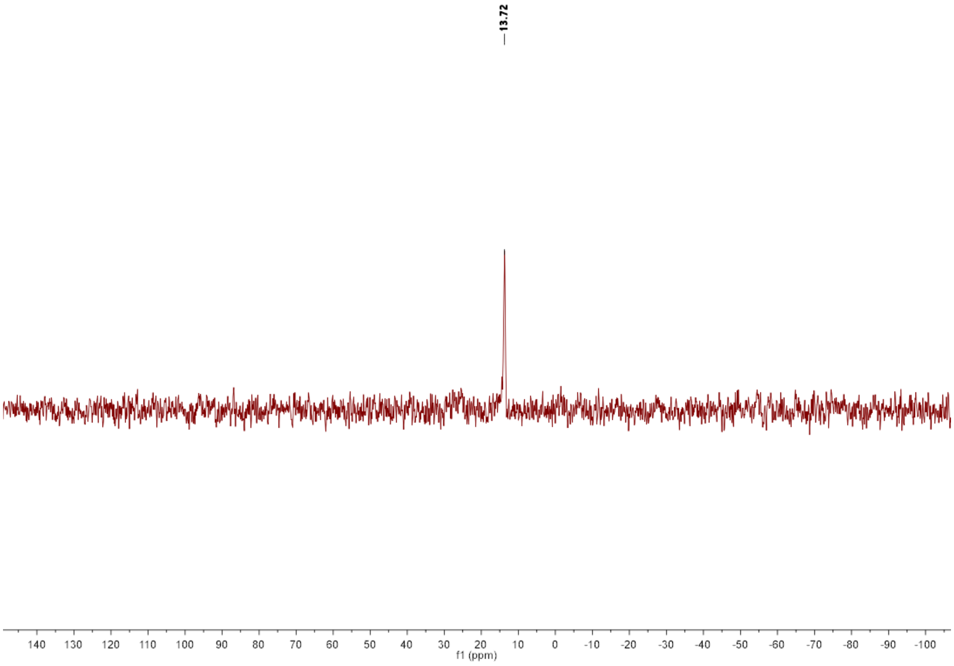

^{31}P NMR of sample (**1** + excess CF_3SO_3Li) (202 MHz, CD_3COCD_3,): δ 13.72 (s, $P(C_6H_4F)_3$).

^{19}F NMR of sample (**1** + 10 equiv. CF_3SO_3Li)

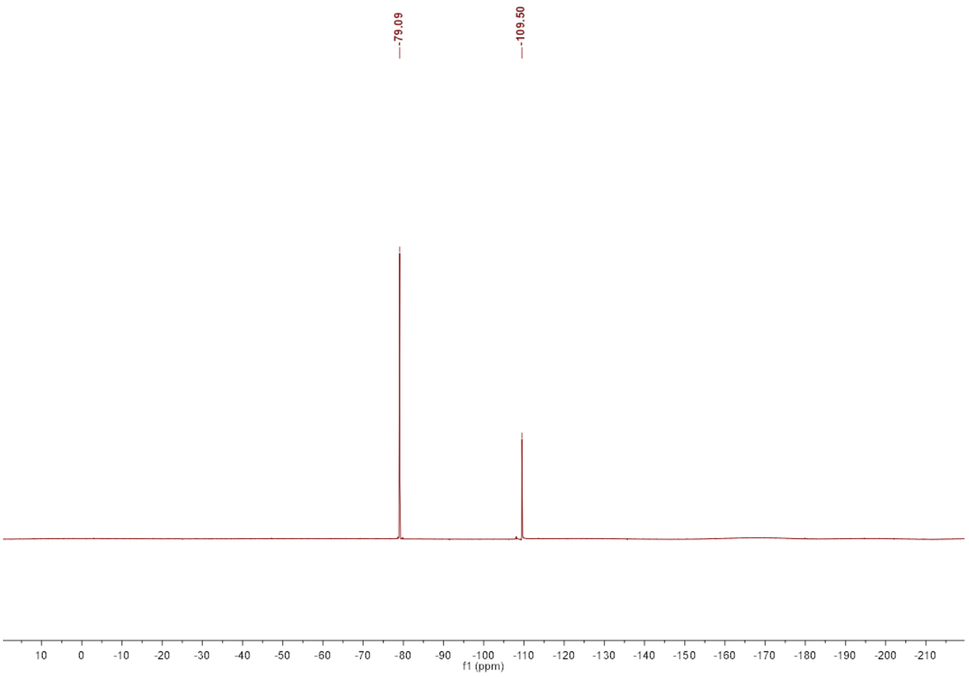

^{19}F NMR of sample (**1** + excess CF_3SO_3Li) (282 MHz, CD_3COCD_3): δ -109.50 (s, $P(C_6H_4F)_3$), -79.09 (s, ($CF_3SO_3^-$) .

IR of sample **(1** + excess CF_3SO_3Li)

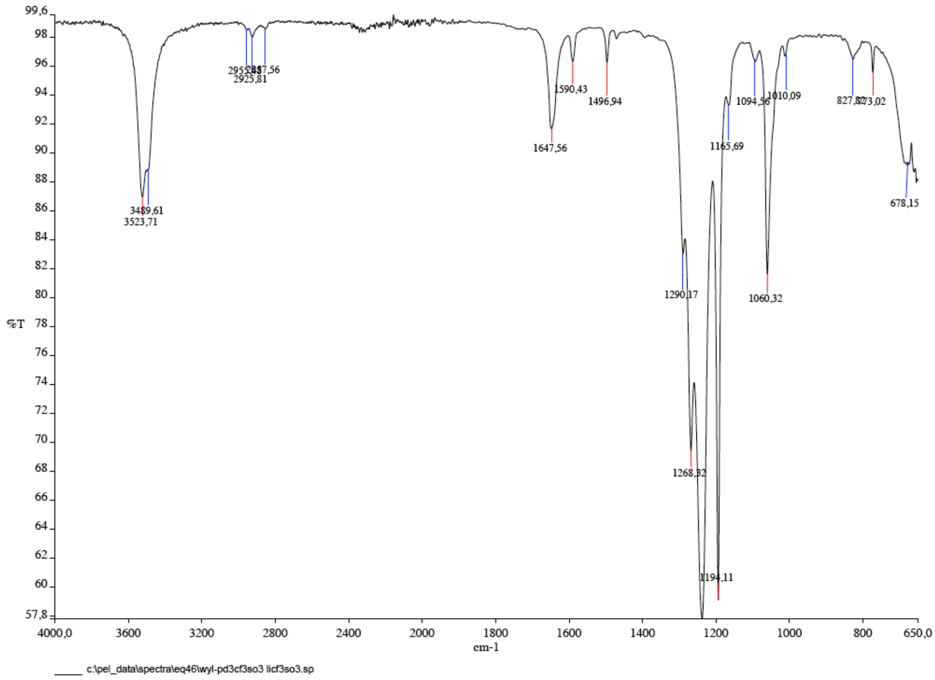

IR (cm^{-1}) of sample (**1** + excess CF_3SO_3Li): ν 3523, 3489, 2955, 2926, 2857, 1647, 1590, 1497, 1290, 1268, 1238, 1194, 1165, 1094, 1060, 1010, 827, 678.

UV-vis. of sample (**1** + excess CF_3SO_3Li)

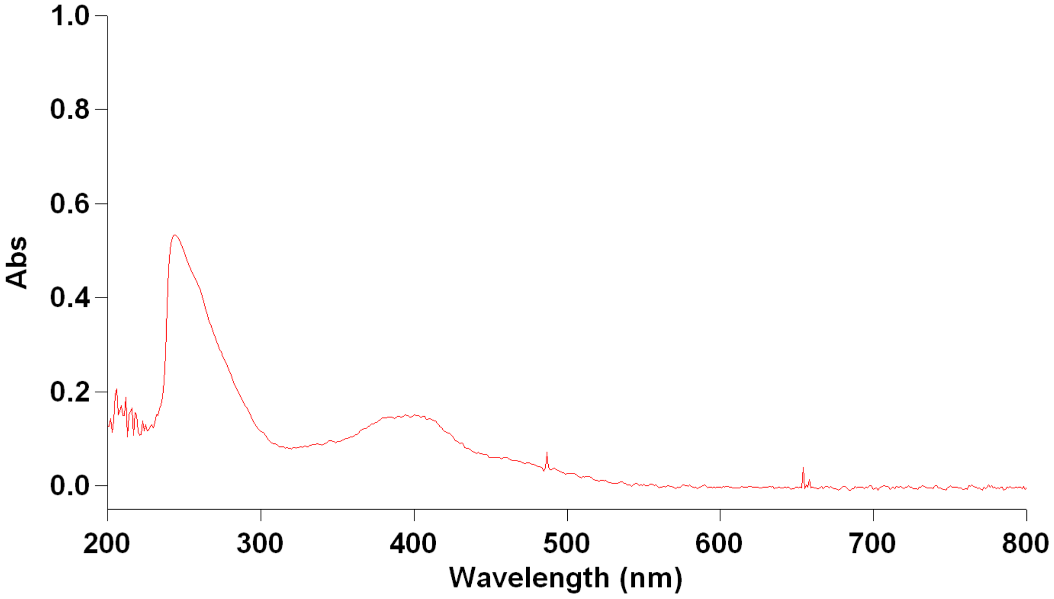

UV-vis. for sample (**1** + excess CF_3SO_3Li): c = 6.5 × 10^{-6} mol/L in $CHCl_3$, λ_{max} = 245 nm, ε_{max} = 8.7 × 10^4 M^{-1} cm^{-1}.

4.12. Spectra 1-BF_4 + 10 equiv. $LiBF_4$

HRMS of **1-BF_4** + 10 equiv. $LiBF_4$

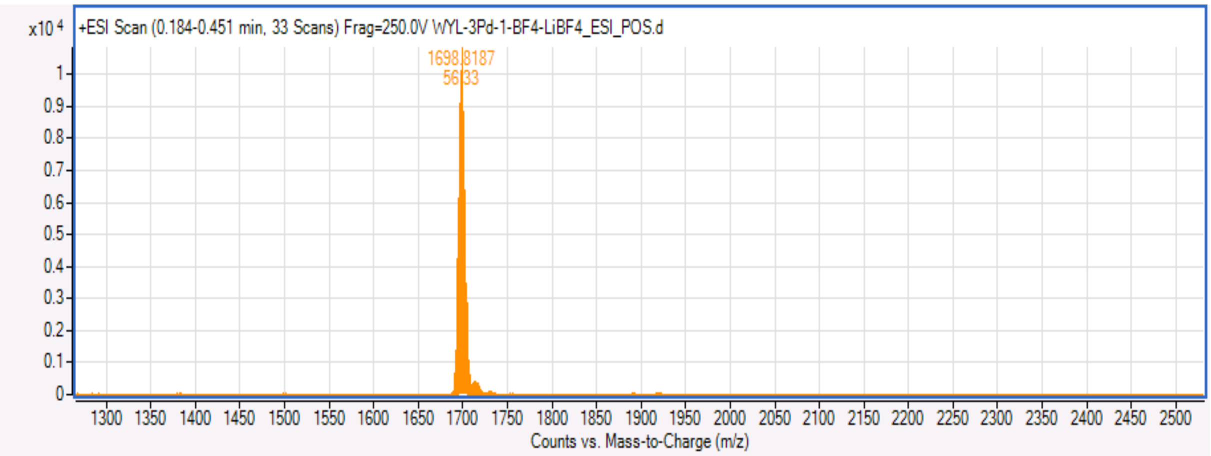

HRMS calculated for $C_{72}H_{48}Cl_3F_9P_3Pd_3S_3LiBF_4^+$ 1792.8366, found (without $LiBF_4$) 1698.8187.

^{1}H NMR of **1-BF_4** + 10 equiv. $LiBF_4$

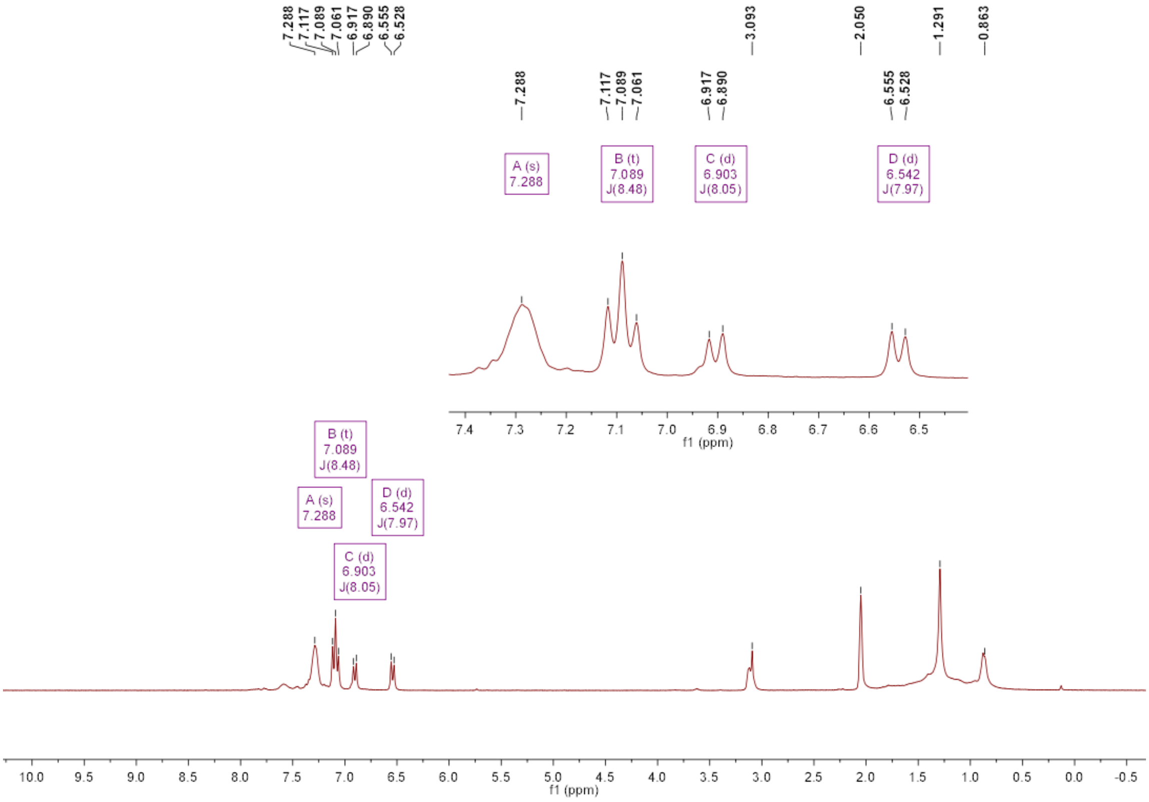

^{1}H NMR of sample (**1-BF_4** + 10 equiv. $LiBF_4$) (500 MHz, CD_3COCD_3): δ 7.29 (br s, 18H, H2), 7.09 (t, J = 8.5 Hz, 18H, H3), 6.90 (d, J = 8.1 Hz, 6H, H7), 6.54 (d, J = 8.0 Hz, 6H, H6); 3.09 (s, H_2O), 0.86, 1.29 (hexane).

^{13}C NMR of sample (**1-BF$_4$** + 10 equiv. LiBF$_4$)

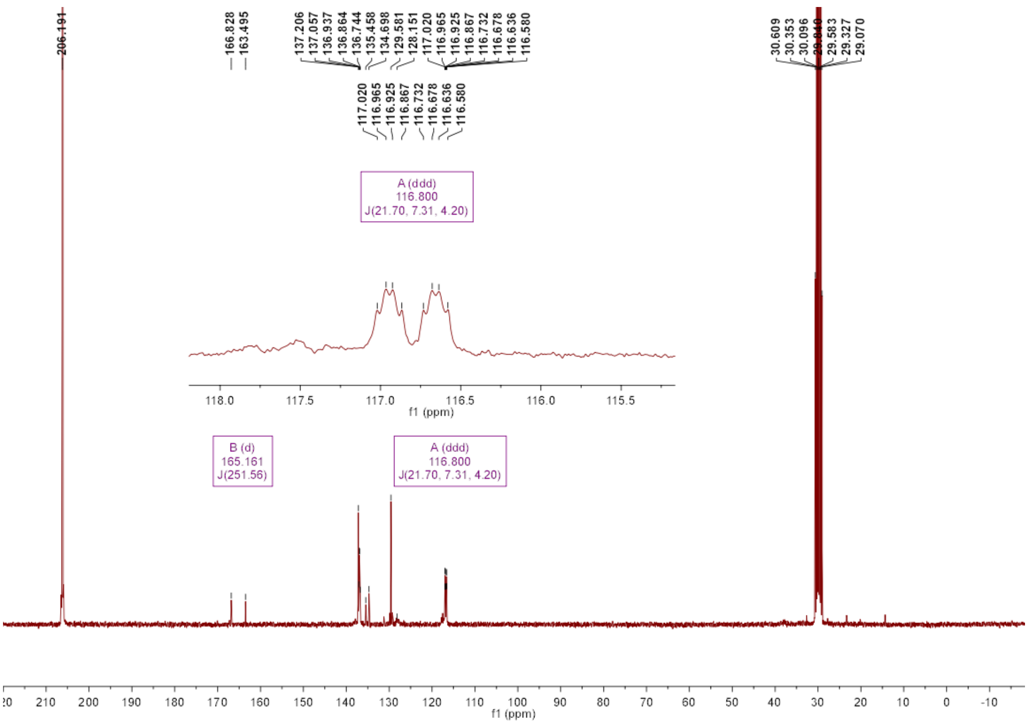

^{13}C NMR of sample (**1-BF$_4$** + 10 equiv. LiBF$_4$) (75 MHz, CD_3COCD_3): δ 165.2 (d, J = 251.6 Hz, C4), 137.2 (br s, C2), 136.9 (m, C6), 135.5 (C8), 134.7 (C5), 129.6 (C7), 128.1 (br s, C1), 116.8 (ddd, J = 21.7, 7.3, 4.2 Hz, C3).

^{31}P NMR of sample (**1-BF$_4$** + 10 equiv. LiBF$_4$)

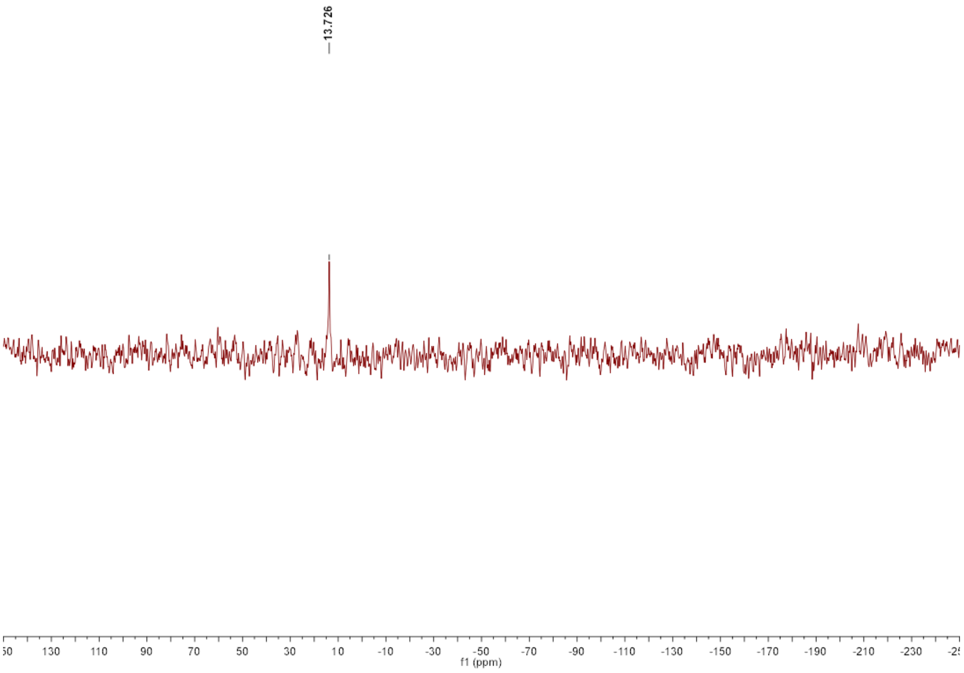

^{31}P NMR of sample (**1-BF$_4$** + 10 equiv. LiBF$_4$) (202 MHz, CD_3COCD_3,): δ 13.73 (s, $P(C_6H_4F)_3$).

^{19}F NMR of sample (**1-BF$_4$** + 10 equiv. $LiBF_4$)

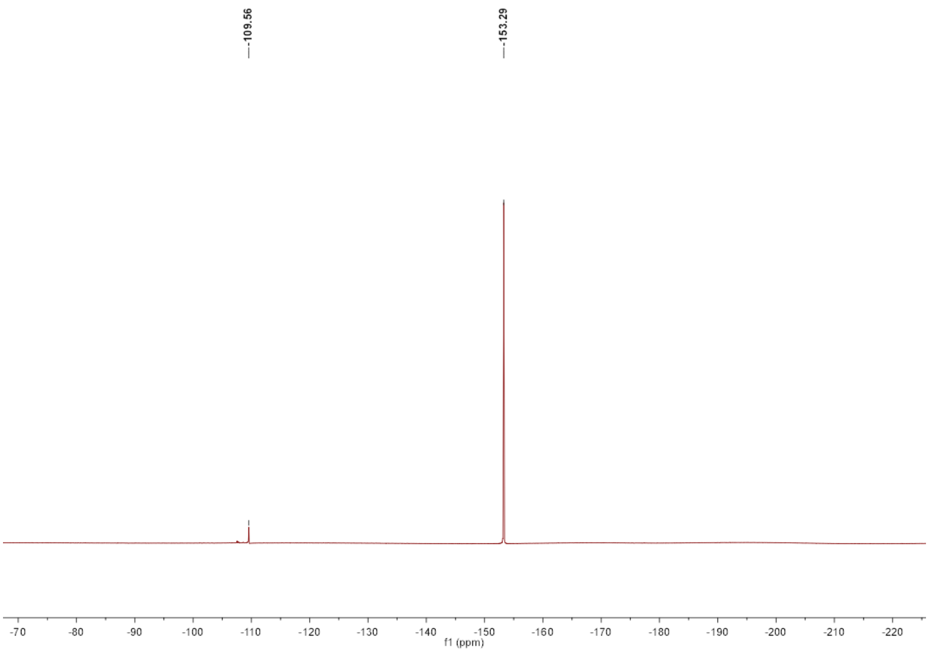

^{19}F NMR of sample (**1-BF$_4$** + 10 equiv. $LiBF_4$) (282 MHz, CD_3COCD_3): δ -109.56 (s, $P(C_6H_4F)_3$), -153.29 (s, BF_4^-) .

IR of sample (**1-BF$_4$** + 10 equiv. $LiBF_4$)

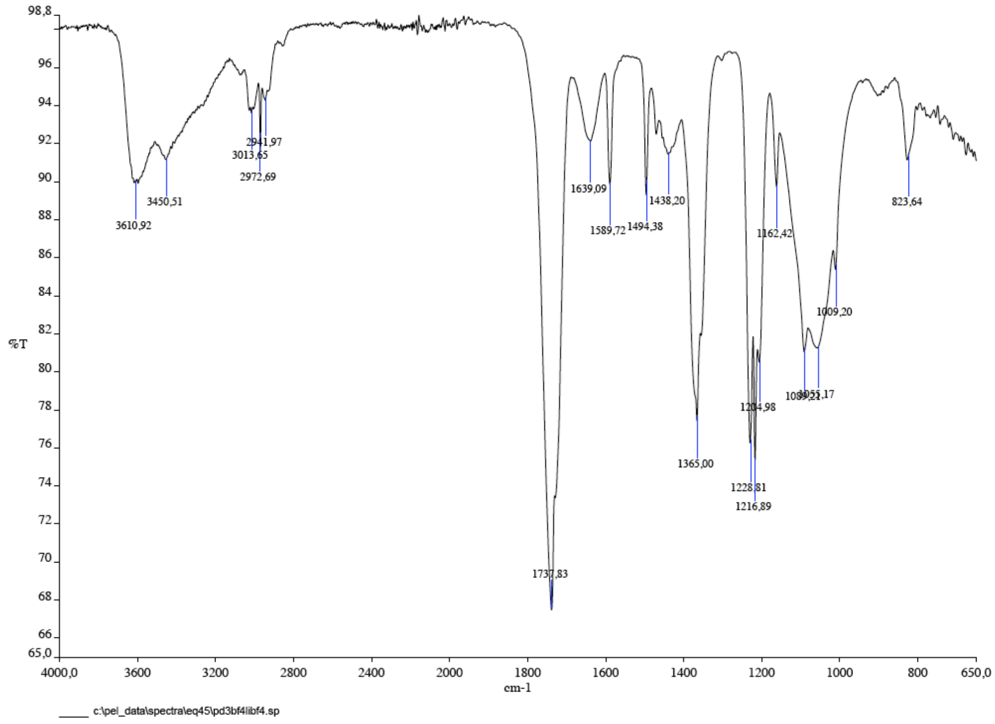

IR (cm^{-1}) of sample (**1-BF$_4$** + 10 equiv. $LiBF_4$): ν 3610, 3450, 3013, 2972, 2941, 1737,1639, 1589, 1494, 1438, 1365, 1228, 1216, 1204, 1162, 1093, 1030, 1009, 824.

UV-vis. of **1-BF$_4$** + 10 equiv. $LiBF_4$

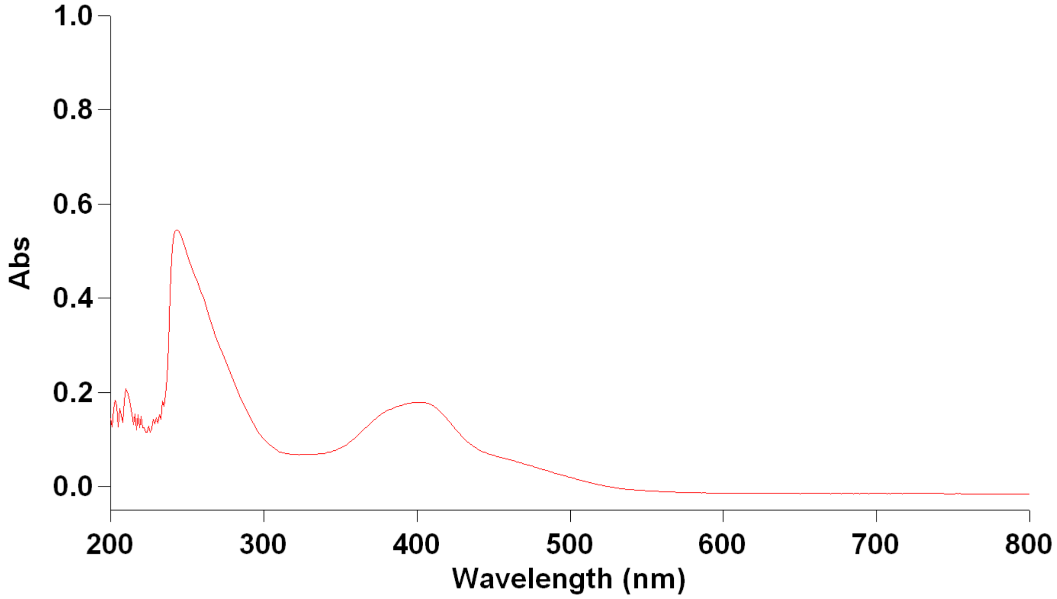

UV-vis. for sample (**1-BF$_4$** + 10 equiv. $LiBF_4$): c = 1 × 10^{-5} mol/L in $CHCl_3$, λ_{max} = 243 nm, ε_{max} = 5.5 × 10^4 M^{-1} cm^{-1}.

5. Computational analyses

Li^+ complexations

Initial structure of the adduct between the all-metal aromatic Pt_3 cation (fragment optimized at the M06/Def2-svp) and Li^+ (below) and optimized structure (next page). Li^+ has been initially put 6 A above the trimetallic core and approaches it center throughout the process. The graph plots the calculated energy during the optimization process, presenting the stabilizing contribution of Li^+ binding by the aromatic Pt_3 cluster (a dummy atom has been put in the center of the triangle to show clearly the distance).

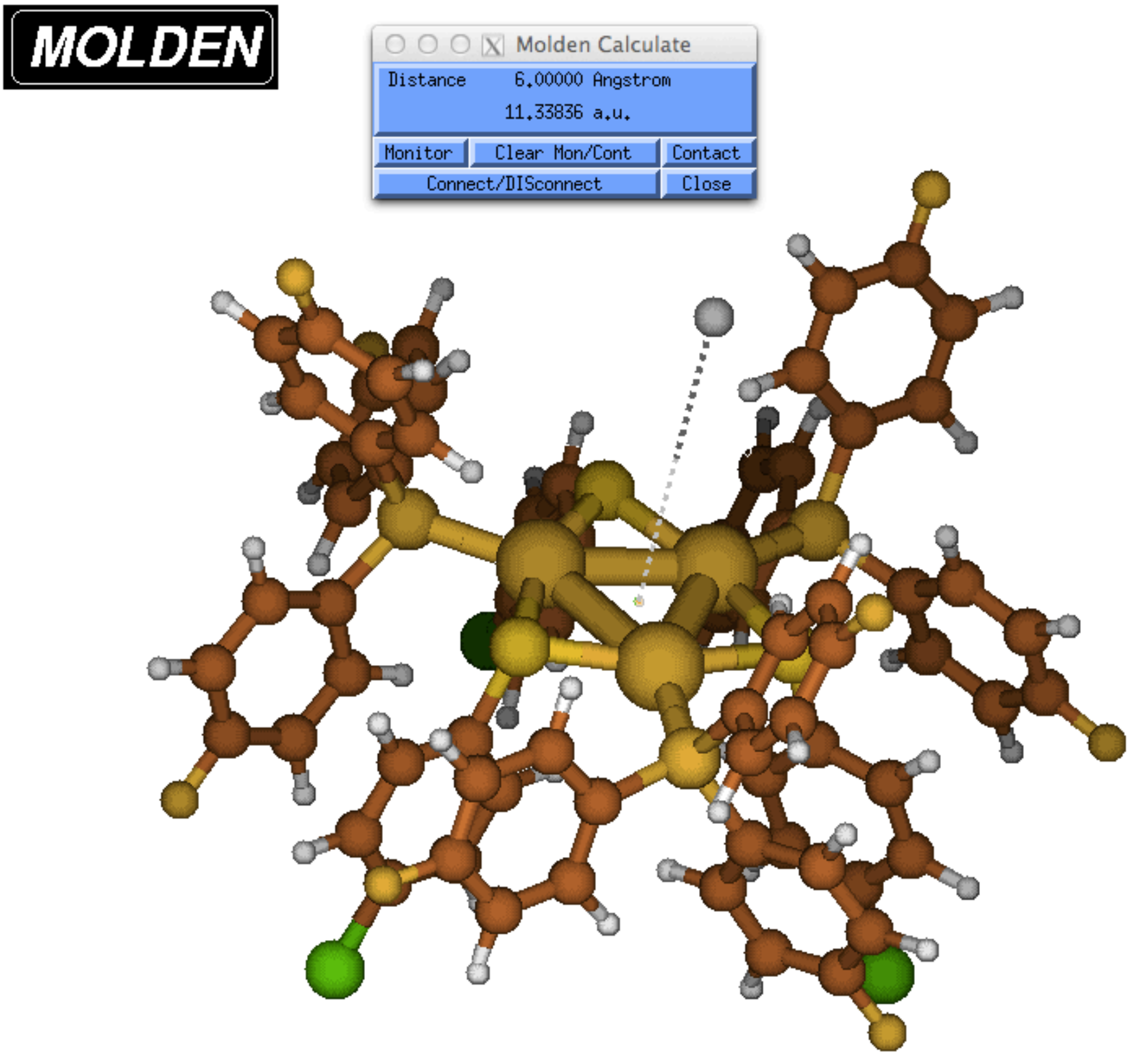

Initial geometry (Li^+ - Pt_3^+ = 6.0 Angstrom).

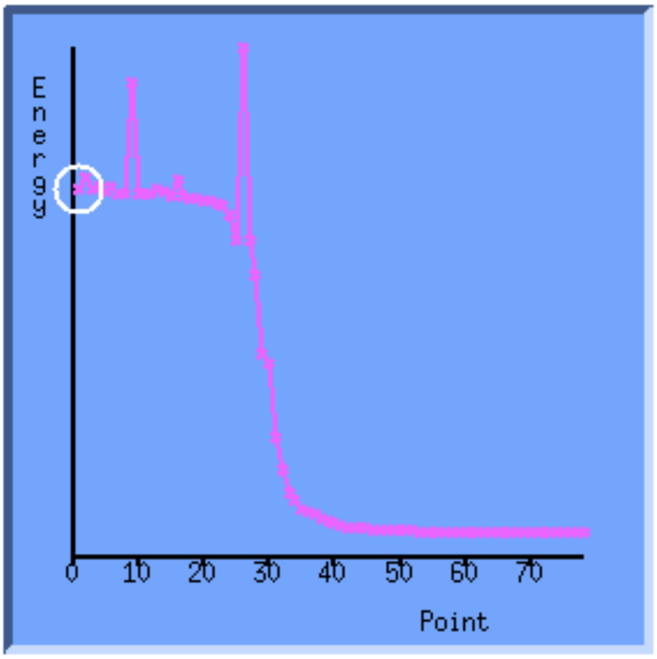

Energy decreases while Li^+ approaches the trimetallic core during optimization (*without any constrain*).

MOLDEN

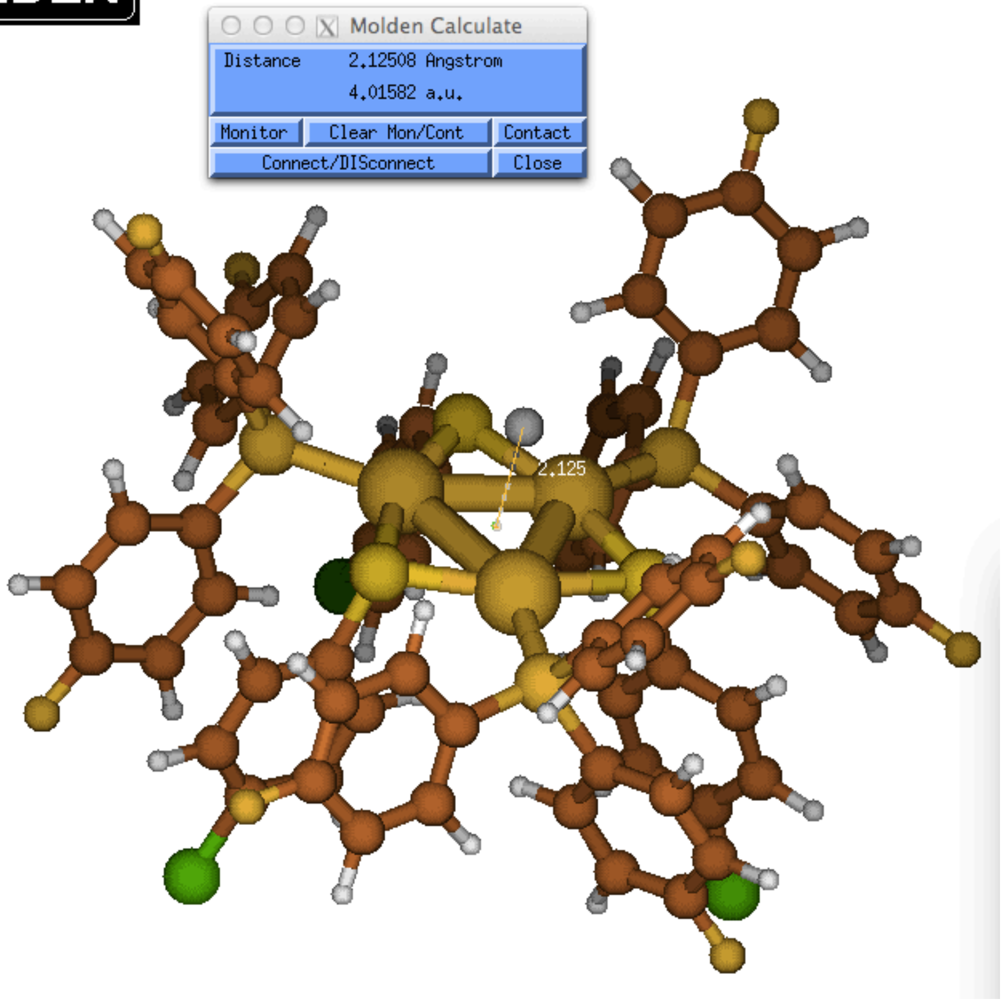

Optimized structure (Li^+ - Pt_3^+ = 2.12508 Angstrom).

HOMO of the optimized $[M_3\text{-Li}]^{2+}$ adducts

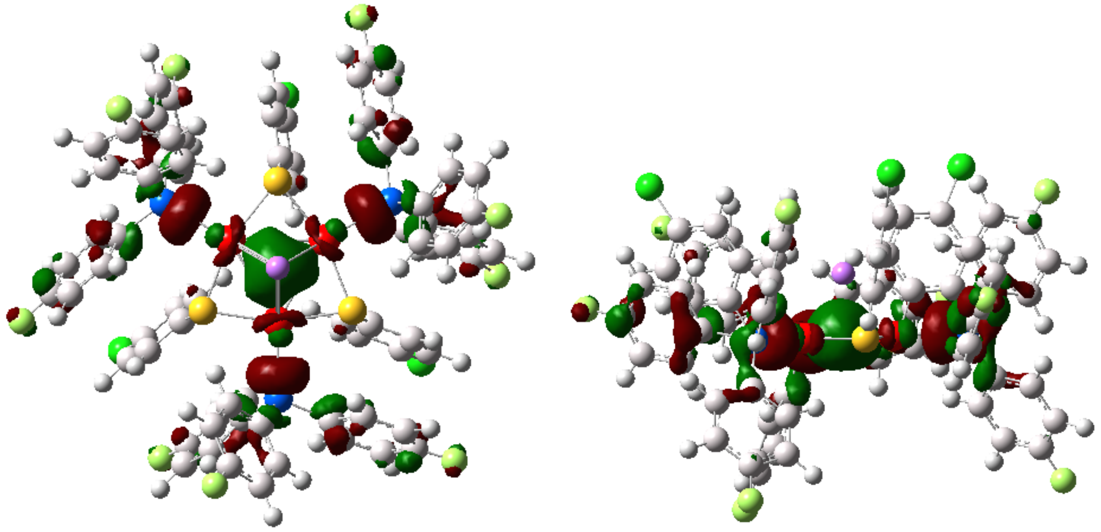

Top and side view of the HOMO of the optimized $[Pd_3\text{-Li}]^{2+}$ adduct.
Compared to the HOMO of cation **1**, this MO is elongated towards the alkali metal.

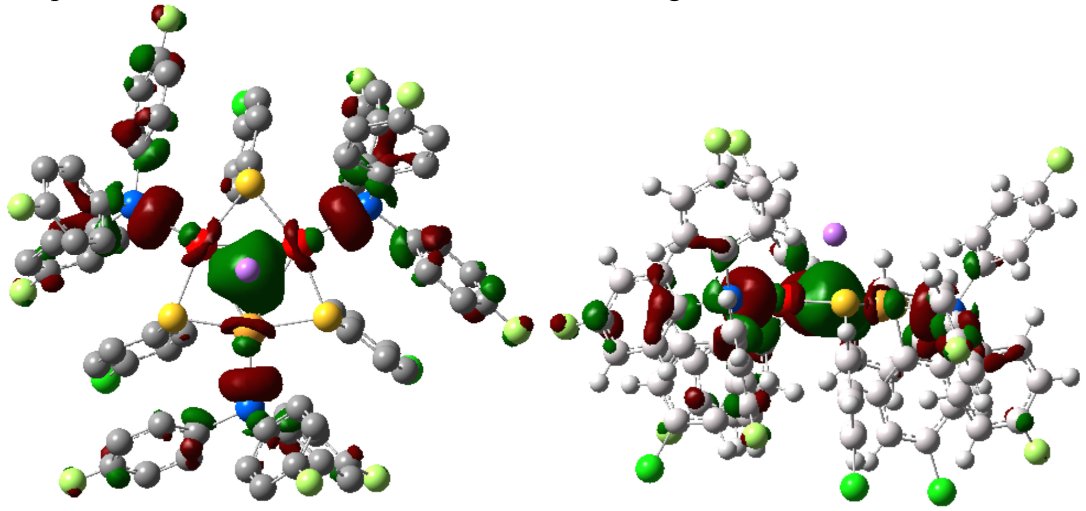

Top and side view of the HOMO of the optimized $[Pd_2Pt\text{-Li}]^{2+}$ adduct.
Compared to the HOMO of cation **1** with 1 Pt and 2 Pd nuclei, this MO is elongated towards the alkali metal.

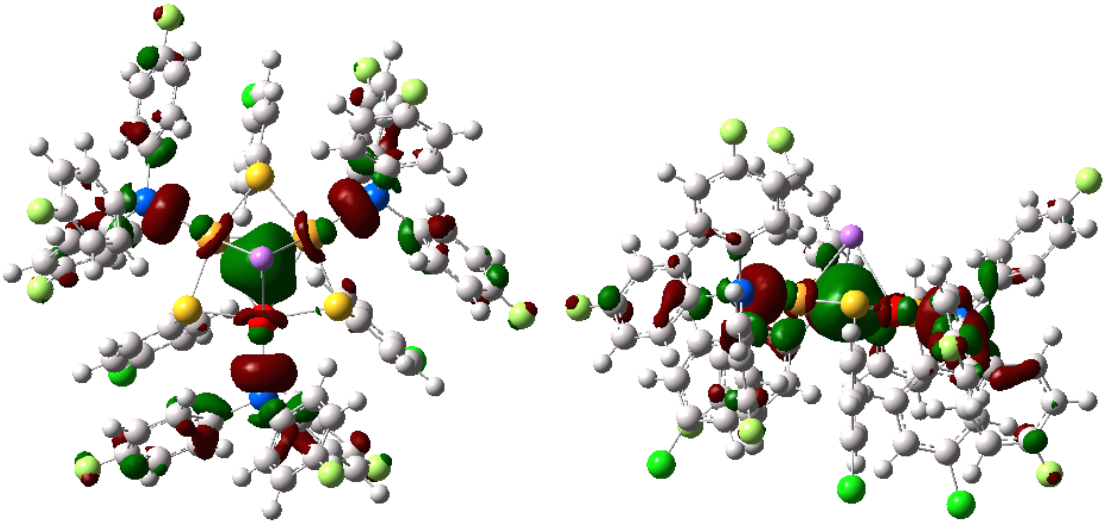

Top and side view of the HOMO of the optimized $[PdPt_2\text{-}Li]^{2+}$ adduct.
Compared to the HOMO of cation **1** with 2 Pt and 1 Pd nuclei, this MO is elongated towards the alkali metal.

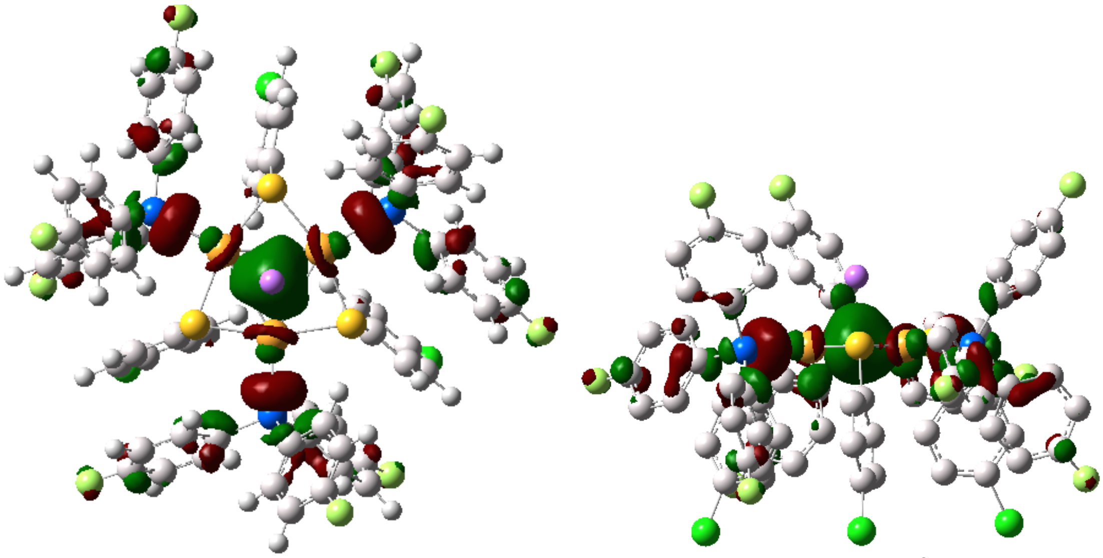

Top and side view of the HOMO of the optimized $[Pt_3\text{-}Li]^{2+}$ adduct.
Compared to the HOMO of cation **1** with 3 Pt nuclei, this MO is elongated towards the alkali metal.

In each case, the delocalized sigmoid MO that makes these clusters d-orbital aromatic elongates towards Li^+ to complex the alkali cation. This looks as the same type of bonding interaction observed modeling a regular aromatics as benzene instead of all-metal aromatic and heteroaromatic frameworks.

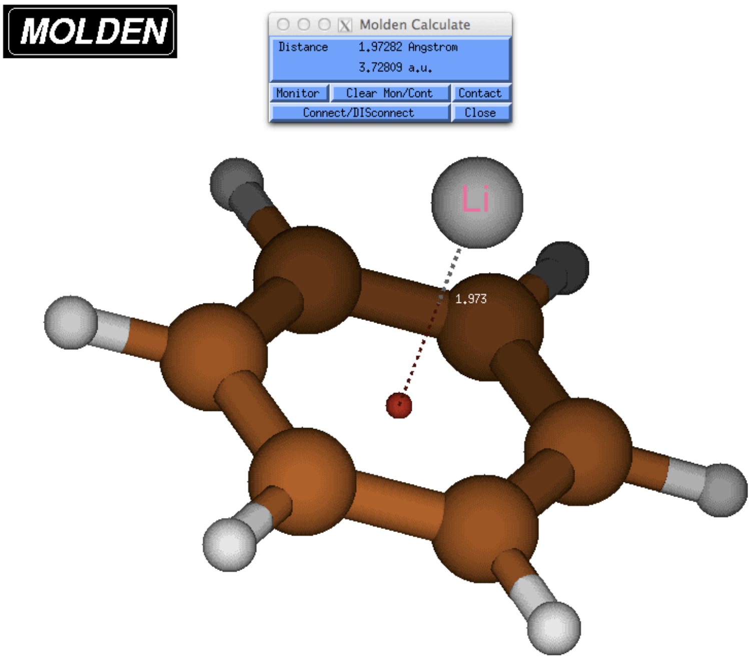

Optimized structure of the Li^+-benzene complex (Li^+ - C_6H_6 = 1.97292 Angstrom, the red dot is a dummy atom put in the center of the aromatic ring).

Comparison of ΔGs for Li^+ binding

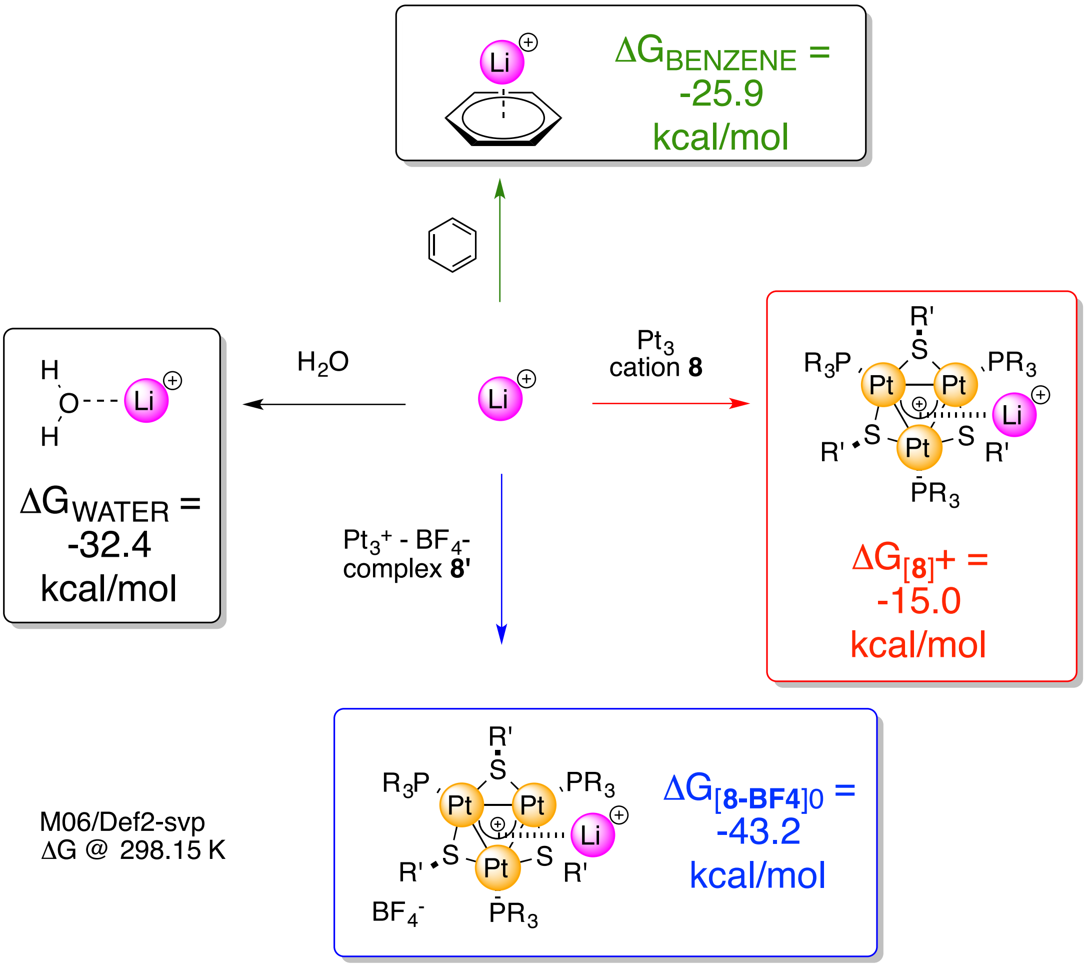

TABLE OF ΔGs

	H (Hartree)	ZPC (Hartree)	S (cal/mol*K)	ΔH (kcal/mol)	ΔS (cal/mol)	ΔG 298.15 K (kcal/mol)
Li^+	-7,286810	0,000000	31,798			
benzene	-231,787059	0,099951	69,078			
[Li^+-benzene]	-239,126921	0,101488	76,176	-33,29	-24,70	**-25,93**
water	-76,302841	0,021579	46,481			
[Li^+-water]	-83,652206	0,024802	55,172	-39,25	-23,11	**-32,36**
Pt_3^+ (cation **1**)	-7621,298337	1,002327	468,503			
[Pt_3^+-Li^+]	-7628,593081	1,003653	534,075	-4,98	33,77	**-15,05**
Pt_3^+ BF_4^- (**1'**)	-8045,498086	1,021337	483,823			
[Pt_3^+-Li^+] BF_4^-	-8052,866216	1,023390	489,489	-51,03	-26,13	**-43,24**
Pt_2Pd^+ BF_4^- (**1''**)	-8054,032724	1,021132	484,004			
[Pt_2Pd^+-Li^+] BF_4^-	-8061,397640	1,023092	489,287	-49,01	-26,52	**-41,11**
$PtPd_2^+$ BF_4^- (**1'''**)	-8062,566638	1,021278	482,248			
[$PtPd_2^+$-Li^+] BF_4^-	-8069,928463	1,023192	488,009	-47,07	-26,04	**-39,31**
Pd_3^+ BF_4^- (**1**^{IV})	-8071,099704	1,021746	480,849			
[Pd_3^+-Li^+] BF_4^-	-8078,458948	1,023222	487,597	-45,45	-25,05	**-37,98**

Optimized structures of $[Pt_3^+ - Li^+]$ adduct with the non-coordinating BF_4^- counteranion (highlighted in red). The anion is far from all metal centers and basically occupies the same position found in solid states structures (fully occupied for Pa-3 crystals, half occupied for R-3c ones, see ref 11 of the main article).

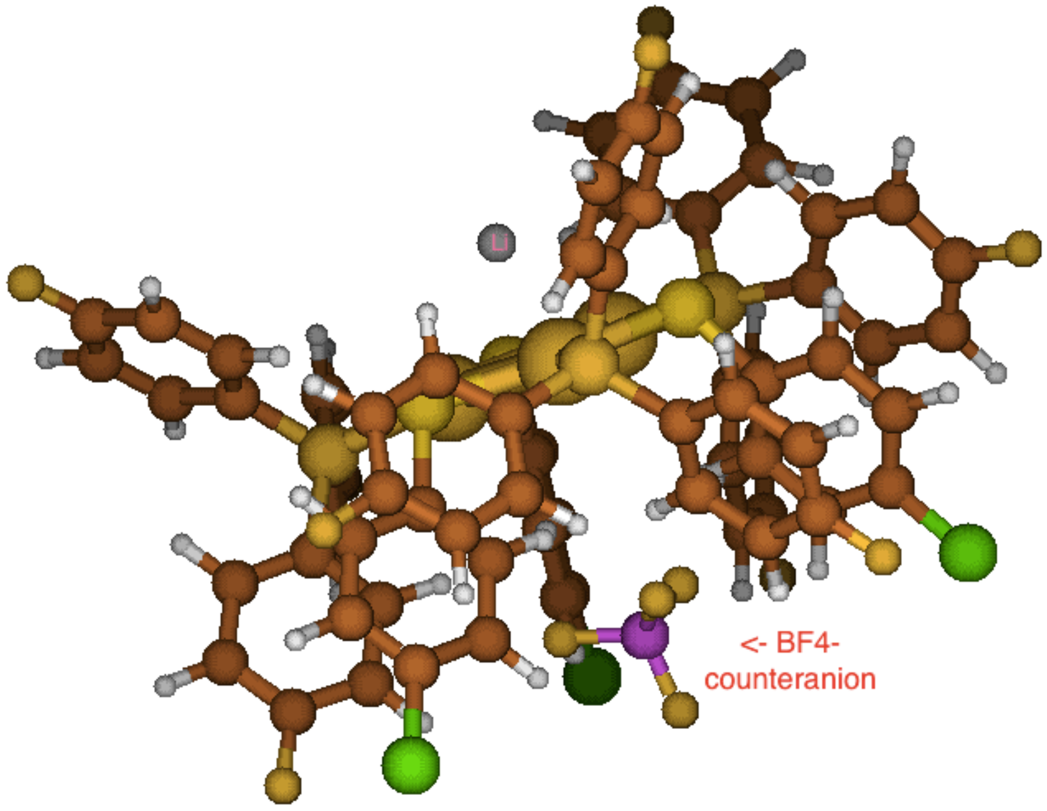

Analogies between σ-aromatic Au_3^+ (ref. 12) and Pd_3^+ (ref. 11) complexes

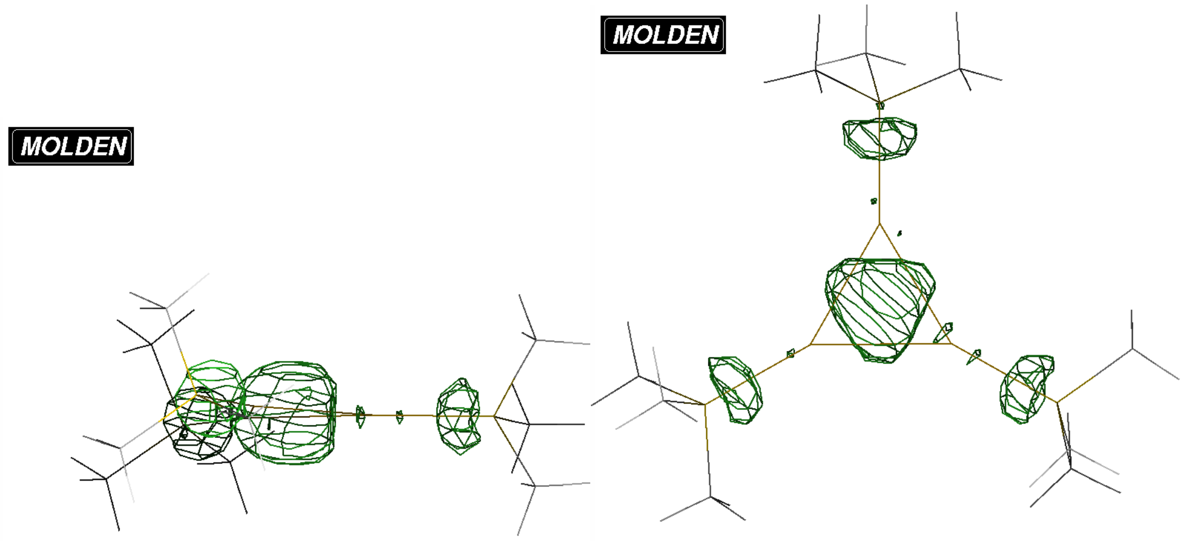

HOMO (3-center-2-electron bond) of $[AuPMe_3]_3^+$ highlighting its sigmoid symmetry, side and top view respectively; details in references 12 of the main article.

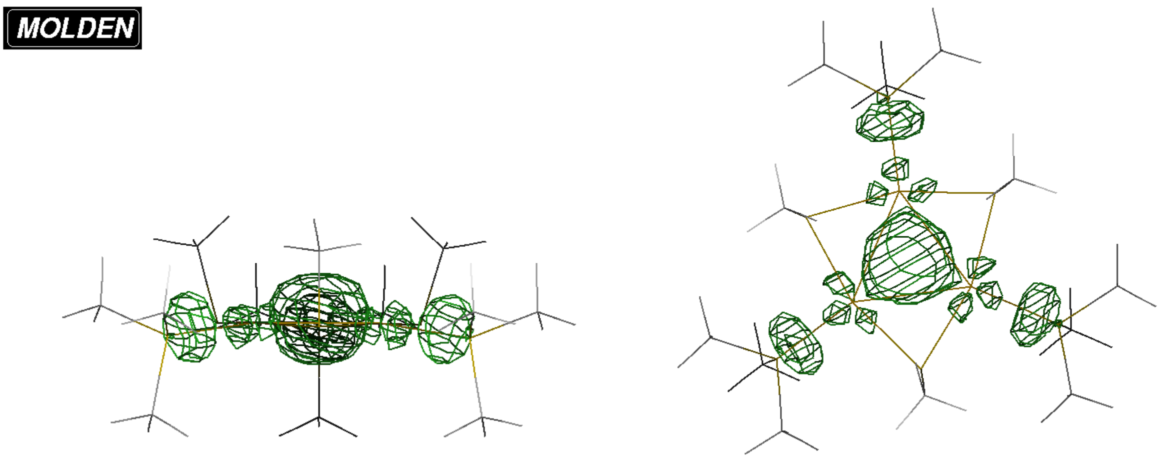

HOMO (3-center-2-electron bond) of $[PdSMePMe_3]_3^+$ highlighting its sigmoid symmetry, side and top view respectively; details in references 11 of the main article.

The comparison shows that both the Au and the Pd cluster present a delocalized metal-metal bond; in both cases, canonical and NBO analyses did not present other metal-metal bonds.

Differences between σ-aromatic Au_4^{++} (ref. 16) and Pd_3^{+}-M’ (M’ = Li^{+}, AgL_n^{+}, $AuPMe_3^{+}$) complexes

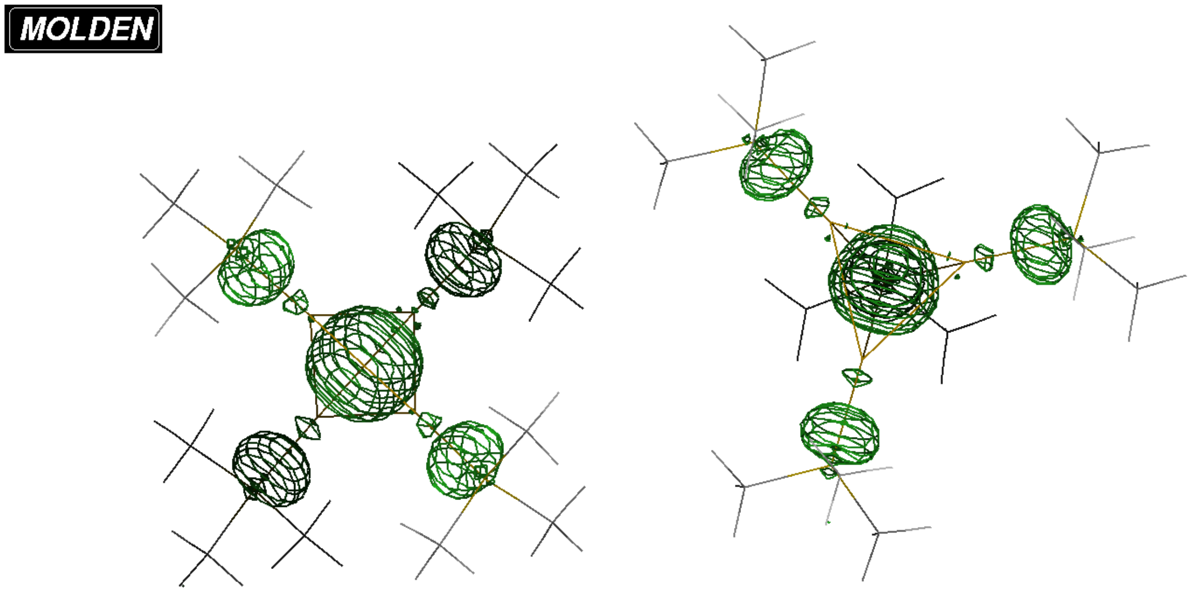

HOMO (4-center-2-electron bond) of $[AuPMe_3]_4^{++}$ highlighting its sigmoid symmetry, side and top view respectively; details in references 16 of the main article.

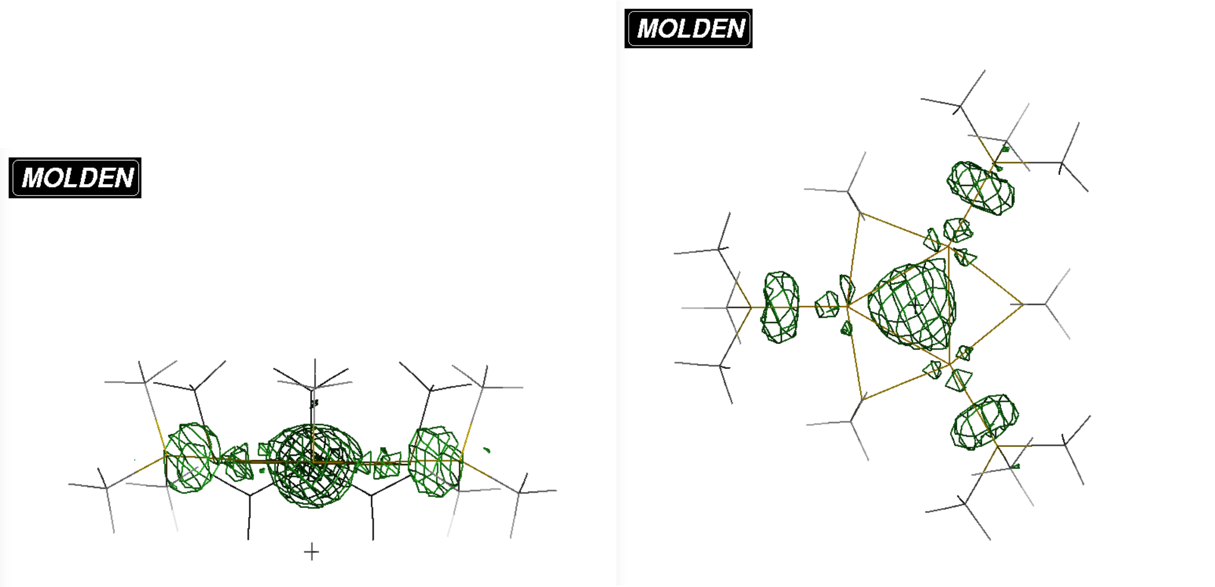

HOMO (3-center-2-electron bond coordinating Li^{+}) of $[PdSMePMe_3]_3Li^{++}$ highlighting the small difference with the bare Pd_3^{+} complex (see previous page), side and top view respectively. Note that phosphines remain coplanar with the Pd triangle, unlike what happened comparing Au_3^{+} with Au_4^{++}. This can be explained with a coordination-like behavior, while it is at odds with the formation of an evenly delocalized 4-center-2-electron bond among the four metal nuclei in heterobimetallic species Pd_3M^{++}.

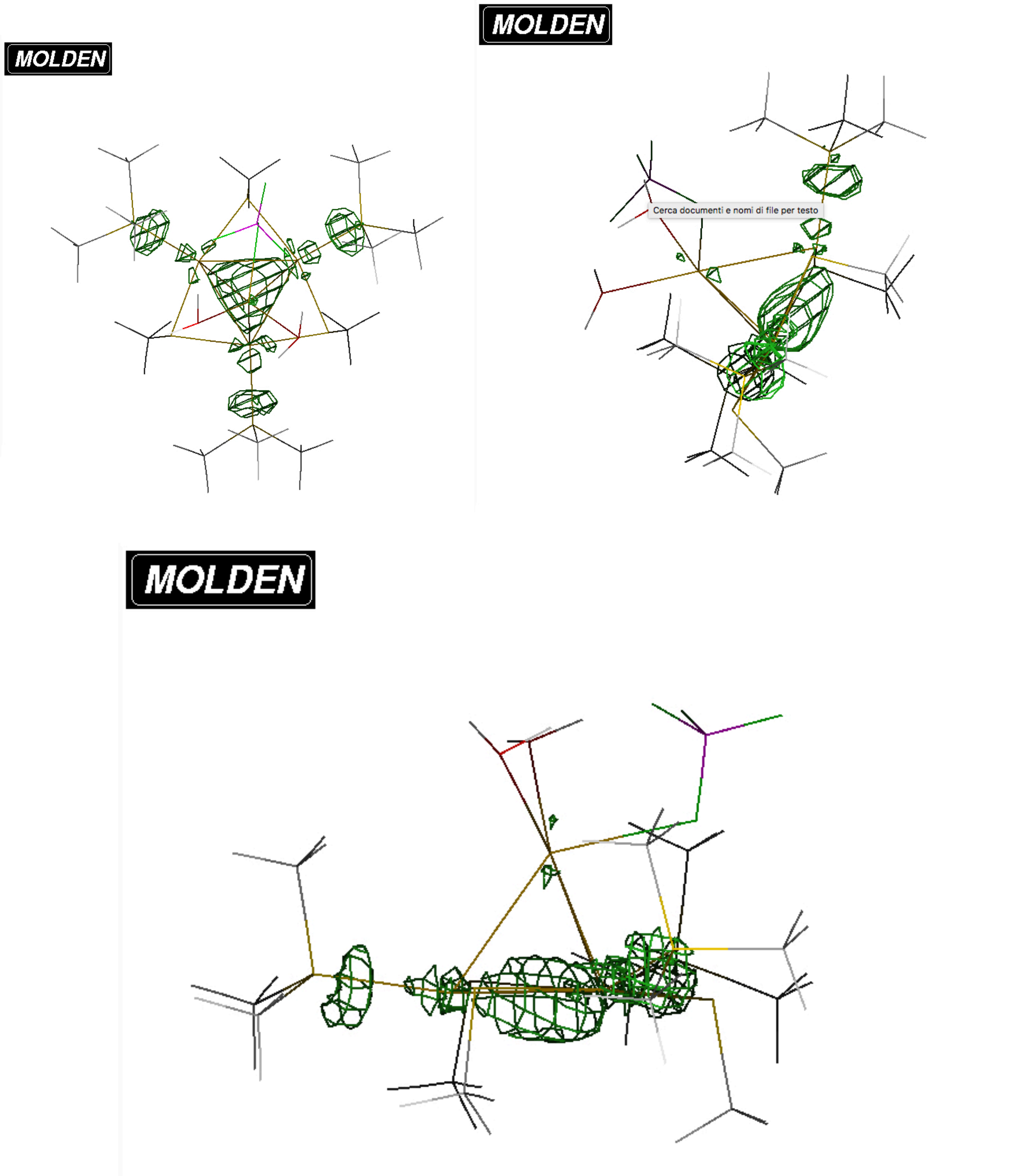

HOMO (3-center-2-electron bond coordinating $Ag(H_2O)_2BF_4$ of $[PdSMePMe_3]_3M'^{++}$ highlighting the small difference with the bare Pd_3^+ complex (see page 69), top, flank and side view respectively. The Ag fragment is the same observed experimentally in crystals of 2-BF_4 used for XRD.

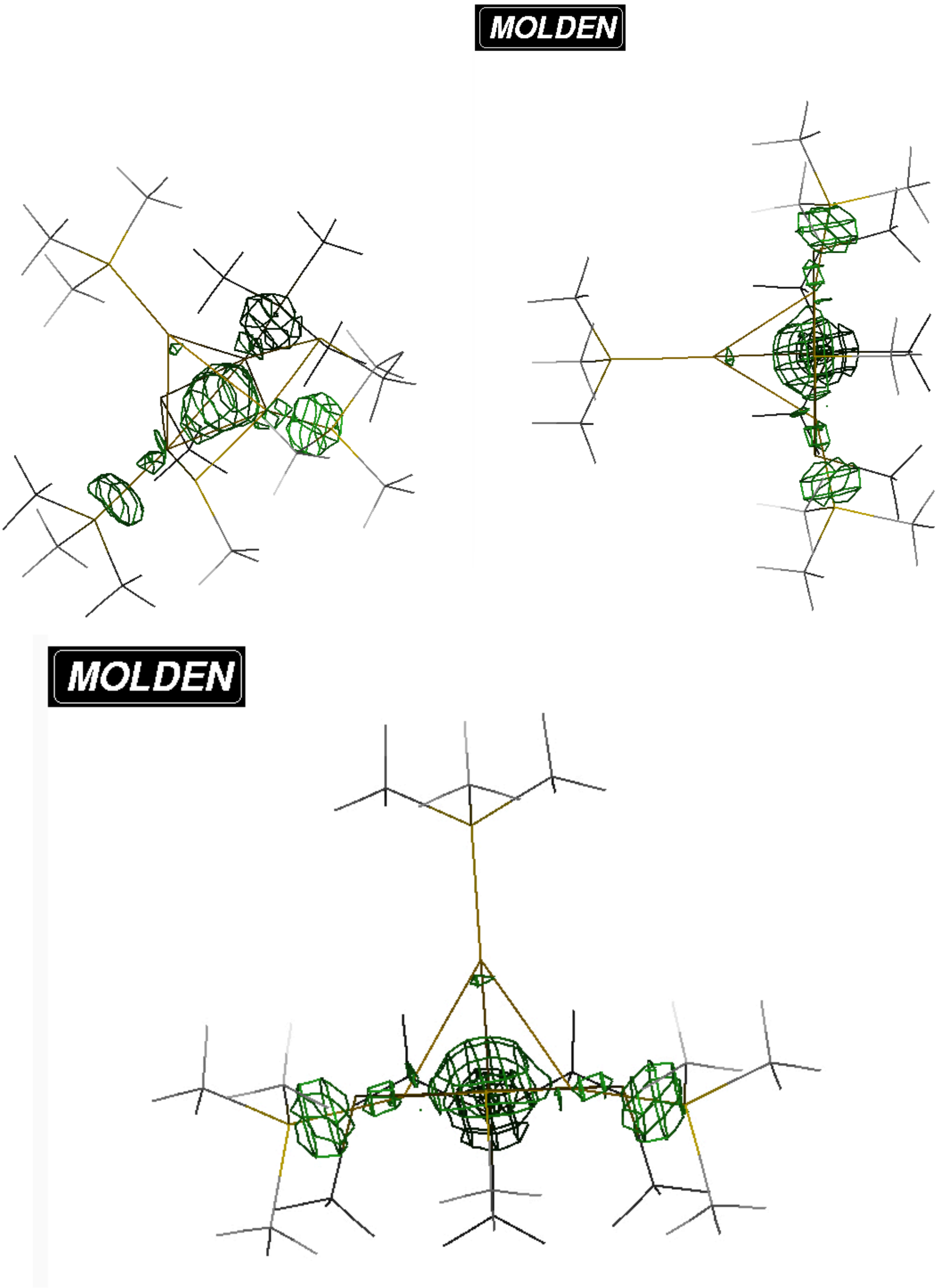

HOMO (3-center-2-electron bond coordinating $AuPMe_3$ of $[(PdSMePMe_3)_3AuPMe_3]^{++}$ highlighting the small difference with the bare Pd_3^+ complex (see page 69), top, flank and side view respectively. Note the localization of the bond on the Pd_3 face of the tetranuclear pyramid, in sharp contrast to the picture of Au_4^{++} complex (see page 71).

Table of NICS values obtained from GIAO magnetic shielding tensors

Negative values confirm the presence of cyclic electron delocalization in all cases.
Those of the Pd_3 face of Pd_3Li^{++} parallel that of bare Pd_3 complex (first two columns). Values calculated from a Pd_2Li face are significantly different. They are still negative and decrease linearly at higher distances from the pyramid (third column).
The trend is opposite regarding Au_4^{++} complex (last two columns). Each face provides identical absolute values (last two columns). Furthermore, the trend presents two flex points in the 0 to 5 Angstrom region, in striking contrast with values of Pd_3M^{++} complexes.
This combines with experimental results (from NMR and X-ray) and other modeling techniques to strengthen the rationale for two distinct bonding mode among these tetranuclear species.
Calculation with double- and triple-z basis sits gave comparable results and identical trends.

Pd3+	Pd3Li++		Au4++		Pd3Ag++		Pd3Au++	
	Pd3 face	Pd2Li face	Au3 face	Au3' face	Pd3 face	Pd2Ag face	Pd3 face	Pd2Au face
-27,6385	-25,4326	-16,9198	-4,4658	-4,3329	-35,338	-4,8574	-36,0748	-13,1278
-27,4106	-23,2558	-13,9055	-0,9986	-0,8624	-33,3502	-3,0598	-30,0379	-9,6935
-25,8264	-20,0197	-11,0485	-0,8378	-0,7034	-30,2464	-3,4349	-22,0613	-7,2747
-21,7777	-16,6049	-8,79985	-6,8338	-6,7197	-24,9645	-6,1039	-14,5943	-7,4227
-14,2479	-12,0465	-5,7944	-10,1872	-10,1349	-15,641	-5,8447	-10,1406	-7,1124
-9,084	-7,9405	-5,9334	-6,5054	-6,4968	-9,7162	-6,2901	-7,1164	-7,2412
-6,3931	-5,7944	-4,7269	-4,1627	-4,1734	-6,9497	-5,6464	-5,473	-5,6082
-4,6684	-4,4434	-3,4194	-3,0987	-3,1115	-5,1246	-4,4836	-4,3627	-4,1087

TZVP	Pd3Li++	Au4++		Pd3Ag++		Pd3Au++	
Dist (A)	Pd2Li face	Au3 face	Au3' face	Pd3 face	Pd2Ag face	Pd3 face	Pd2Au face
0,00	-17,5432	-4,7873	-4,5676	-38,2175	-5,0857	-35,9306	-12,6725
0,20	-14,3653	-1,1944	-0,9617	-36,1774	-3,2498	-29,9353	-9,2047
0,50	-11,0894	-0,7521	-0,5095	-32,4463	-3,4072	-22,0447	-6,6995
1,00	-8,289	-6,3357	-6,1065	-25,7406	-5,7491	-14,6873	-6,7055
2,00	-5,5279	-9,7674	-9,632	-15,4901	-5,6009	-10,2113	-6,5858
3,00	-5,8337	-6,2942	-6,2444	-9,6519	-6,1615	-7,1069	-7,0295
4,00	-4,6271	-4,0481	-4,0388	-6,901	-5,5597	-5,4349	-5,5032
5,00	-3,3546	-3,0319	-3,0335	-5,0778	-4,4216	-4,3278	-4,0499

6. Copy of .cif files for 1-OTf, 2-SbF$_6$ and 2-BF$_4$

Summary of crystallographic information for 1, 2-SbF$_6$ and 2-BF$_4$

	**1-OTf**	**2-SbF$_6$**	**2-BF$_4$**
CCDC number	1410442	1410441	1410440
Empirical formula	$C_{73}H_{48}Cl_3F_{12}O_3P_3Pd_3S_4$	$C_{88}H_{80}AgCl_3$ $F_{21}O_5P_3Pd_3S_3Sb_2$	$C_{72.76}H_{48.76}AgB_2Cl_{5.28}$ $F_{17}O_2P_3Pd_3S_3$
Formula weight	1847.81	2582.53	2103.06
Temperature/K	293(2)	200	200
Crystal system	monoclinic	orthorhombic	orthorhombic
Space group	$P2_1/c$	Pbca	Pccn
a/Å	10.4508(5)	21.8782(4)	19.9665(4)
b/Å	24.6808(11)	22.7145(4)	30.9093(6)
c/Å	29.1391(13)	39.349(3)	32.546(2)
α/°	90	90	90
β/°	94.151(4)	90	90
γ/°	90	90	90
Volume/Å^3	7496.3(6)	19554.8(15)	20085.5(15)
Z	4	8	8
ρ_{calc}g/cm^3	1.637	1.754	1.391
μ/mm^{-1}	1.070	12.846	8.724
F(000)	3664.0	10144.0	8257.0
Crystal size/mm^3	0.123 × 0.114 × 0.106	0.26 × 0.23 × 0.22	0.2 × 0.17 × 0.13
Radiation	MoKα (λ = 0.71073)	CuKα (λ = 1.54187)	CuKα (λ = 1.54187)
2Θ range for data collection/°	4.378 to 54.968	4.492 to 144.734	5.27 to 117.872
Index ranges	$-10 \le h \le 13$, $-26 \le k \le 32$, $-37 \le l \le 37$	$-26 \le h \le 20$, $-18 \le k \le 26$, $-45 \le l \le 34$	$-18 \le h \le 22$, $-23 \le k \le 34$, $-30 \le l \le 36$
Reflections collected	62641	81861	70255
Independent reflections	16722 [R_{int} = 0.0395, R_{sigma} = 0.0485]	18613 [R_{int} = 0.0911, R_{sigma} = 0.0840]	14426 [R_{int} = 0.0685, R_{sigma} = 0.0725]
Data/restraints/parameters	16722/0/947	18613/1140/1162	14426/942/986
Goodness-of-fit on F^2	1.077	1.161	1.196
Final R indexes [I>=2σ (I)]	R_1 = 0.0386, wR_2 = 0.0821	R_1 = 0.0666, wR_2 = 0.1610	R_1 = 0.0806, wR_2 = 0.2144
Final R indexes [all data]	R_1 = 0.0571, wR_2 = 0.0928	R_1 = 0.0877, wR_2 = 0.1925	R_1 = 0.1120, wR_2 = 0.2833
Largest diff. peak/hole / e Å^{-3}	0.57/-0.46	1.30/-2.03	1.71/-1.58

1-OTf (CCDC 1410442)

Ortep of triangular cluster (top) highlighting Pd core and $CF_3SO_3^-$ anion. In the lower caption, $-C_6H_4F$ groups on phosphorous atoms were omitted for clarity (down). Hydrogen atoms omitted for clarity. Thermal ellipsoids are shown at 50% probability.

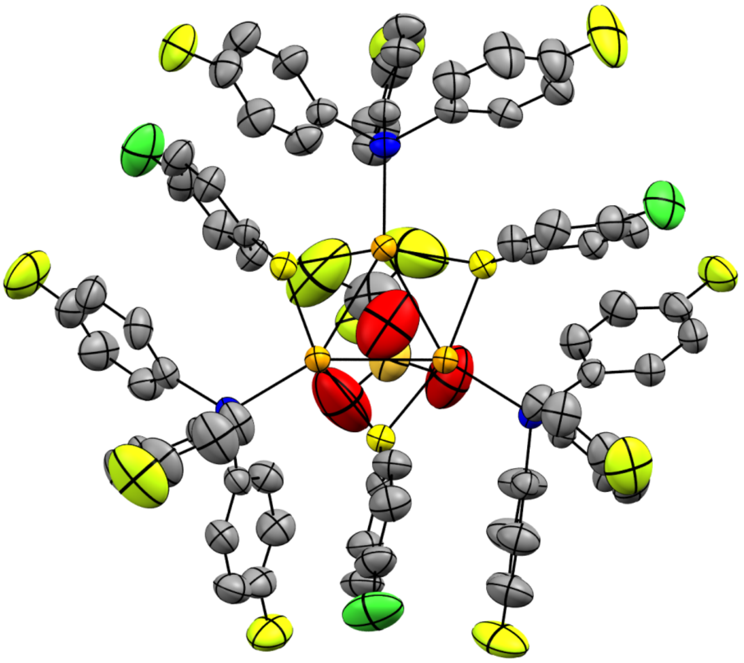

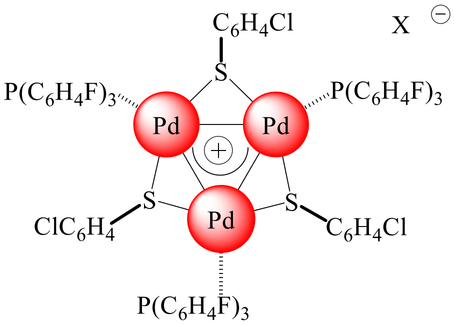

1: X = CF_3SO_3;

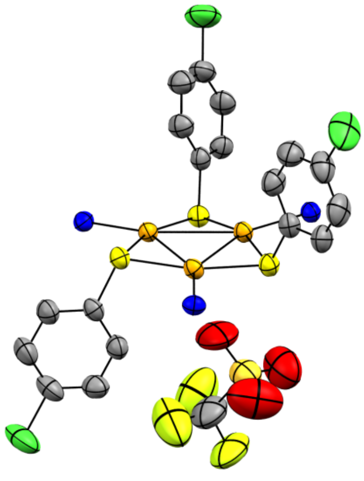

Pd_3 triangle is nearly equilateral, Pd-Pd-Pd angles range between 59.107(8)° and 60.818(8)°.
Pd-Pd distances range between 2.8411(3) Å and 2.8905(3) Å.
Pd-P distances range between 2.2736(8) Å and 2.2926(8) Å.
Pd-S distances range between 2.2700(8) Å and 2.2836(8) Å.

Table 1 Fractional Atomic Coordinates and Equivalent Isotropic Displacement Parameters 1-OTf

Atom	*x*	*y*	*z*	U(eq)
**C1**	0.1198(3)	0.24943(13)	0.19968(11)	0.0458(7)

**C2**	0.1519(4)	0.23367(15)	0.15627(12)	0.0625(10)
**C3**	0.0769(4)	0.19672(17)	0.13022(15)	0.0772(12)
**C4**	-0.0292(4)	0.17626(16)	0.14834(14)	0.0668(10)
**C5**	-0.0653(4)	0.19106(16)	0.19045(14)	0.0695(11)
**C6**	0.0107(3)	0.22765(15)	0.21617(12)	0.0594(9)
**C7**	0.3711(3)	0.30374(12)	0.21196(11)	0.0447(7)
**C8**	0.4797(3)	0.28492(16)	0.23638(13)	0.0612(9)
**C9**	0.6000(4)	0.29328(19)	0.22018(16)	0.0807(13)
**C10**	0.6088(4)	0.32055(18)	0.18047(16)	0.0736(12)
**C11**	0.5050(4)	0.34020(17)	0.15497(14)	0.0713(11)
**C12**	0.1432(3)	0.36272(13)	0.22288(10)	0.0470(8)
**C13**	0.2139(3)	0.40846(14)	0.23598(11)	0.0537(9)
**C14**	0.1651(4)	0.45967(15)	0.22824(13)	0.0623(10)
**C15**	0.0461(5)	0.46425(18)	0.20737(17)	0.0852(13)
**C16**	-0.0263(4)	0.4211(2)	0.19327(18)	0.0944(15)
**C17**	0.0230(3)	0.36946(16)	0.20122(14)	0.0686(11)
**C18**	0.1482(3)	0.06994(13)	0.41250(12)	0.0509(8)
**C19**	0.0421(3)	0.10273(16)	0.41207(15)	0.0711(11)
**C20**	-0.0784(4)	0.0813(2)	0.41857(19)	0.1004(16)
**C21**	-0.0872(4)	0.0274(2)	0.42588(19)	0.0982(16)
**C22**	0.0148(4)	-0.00672(18)	0.42637(17)	0.0903(14)
**C23**	0.1334(4)	0.01473(15)	0.41923(15)	0.0720(11)
**C24**	0.3679(3)	0.05332(12)	0.36089(12)	0.0467(8)
**C25**	0.4918(4)	0.03385(15)	0.36534(13)	0.0621(10)
**C26**	0.5362(4)	0.00016(18)	0.33131(16)	0.0813(13)
**C27**	0.4567(5)	-0.01262(17)	0.29460(16)	0.0790(12)
**C28**	0.3348(4)	0.00643(15)	0.28838(14)	0.0730(11)
**C29**	0.2916(4)	0.03978(14)	0.32252(14)	0.0634(10)
**C30**	0.4064(3)	0.09077(12)	0.45445(11)	0.0471(8)
**C31**	0.3688(4)	0.06382(14)	0.49303(13)	0.0591(9)
**C32**	0.4491(4)	0.06091(16)	0.53306(14)	0.0685(11)
**C33**	0.5643(4)	0.08486(16)	0.53386(13)	0.0638(10)
**C34**	0.6074(4)	0.11280(16)	0.49676(14)	0.0679(10)
**C35**	0.5255(3)	0.11592(14)	0.45740(13)	0.0586(9)
**C36**	0.1826(3)	0.38359(12)	0.48323(11)	0.0457(7)
**C37**	0.0715(3)	0.37012(15)	0.45682(13)	0.0596(9)
**C38**	-0.0478(4)	0.38689(17)	0.46939(16)	0.0768(12)
**C39**	-0.0539(4)	0.41698(18)	0.50761(17)	0.0756(12)
**C40**	0.0515(4)	0.43131(17)	0.53498(15)	0.0777(12)
**C41**	0.1715(4)	0.41474(15)	0.52263(13)	0.0618(10)
**C42**	0.4331(3)	0.33894(12)	0.51402(11)	0.0449(7)
**C43**	0.3828(4)	0.32055(16)	0.55319(14)	0.0689(11)
**C44**	0.4594(5)	0.30129(18)	0.59020(15)	0.0826(13)
**C45**	0.5870(4)	0.30038(17)	0.58746(15)	0.0767(12)
**C46**	0.6416(4)	0.3172(2)	0.54976(16)	0.0896(15)
**C47**	0.5639(4)	0.33712(19)	0.51324(14)	0.0779(12)
**C48**	0.5195(3)	0.41663(14)	0.41884(12)	0.0570(9)
**C49**	0.5827(4)	0.46135(16)	0.40278(14)	0.0684(11)
**C50**	0.5365(4)	0.51141(15)	0.41312(13)	0.0609(10)
**C51**	0.4348(4)	0.51953(14)	0.43765(13)	0.0654(10)
**C52**	0.3738(3)	0.47434(13)	0.45430(12)	0.0555(9)
**C53**	0.4151(3)	0.42255(12)	0.44527(11)	0.0442(7)
**C54**	0.3448(3)	0.15587(12)	0.27366(10)	0.0440(7)
**C55**	0.4710(3)	0.15577(14)	0.29105(12)	0.0544(9)
**C56**	0.5653(4)	0.13419(15)	0.26601(14)	0.0650(10)
**C57**	0.5321(4)	0.11327(15)	0.22306(14)	0.0667(11)
**C58**	0.4078(4)	0.11392(16)	0.20439(14)	0.0702(11)
**C59**	0.3133(4)	0.13507(14)	0.23022(12)	0.0591(9)
**C60**	0.1705(3)	0.39992(12)	0.34297(10)	0.0455(8)
**C61**	0.0436(3)	0.38465(14)	0.33380(12)	0.0589(9)
**C62**	-0.0530(4)	0.42265(16)	0.33138(14)	0.0677(10)
**C63**	-0.0210(4)	0.47656(16)	0.33887(13)	0.0642(10)
**C64**	0.1030(4)	0.49285(14)	0.34871(12)	0.0586(9)
**C65**	0.2001(3)	0.45415(13)	0.35034(11)	0.0508(8)
**C66**	0.2066(3)	0.21291(12)	0.48632(11)	0.0466(8)
**C67**	0.0825(4)	0.22841(15)	0.47335(13)	0.0638(10)
**C68**	-0.0143(4)	0.21770(17)	0.50159(15)	0.0734(11)
**C69**	0.0107(4)	0.19132(18)	0.54214(14)	0.0741(12)
**C70**	0.1339(5)	0.17484(18)	0.55551(13)	0.0773(12)
**C71**	0.2312(4)	0.18598(15)	0.52755(12)	0.0617(10)
**C72**	0.3852(3)	0.33162(15)	0.17089(12)	0.0604(9)
**Cl1**	0.65169(13)	0.08490(6)	0.19160(5)	0.1121(5)
**Cl2**	-0.14313(12)	0.52495(5)	0.33560(5)	0.0991(4)
**Cl3**	-0.11101(14)	0.17766(7)	0.57798(5)	0.1241(5)
**F1**	-0.1014(2)	0.13977(10)	0.12338(9)	0.0975(8)

**F2**	0.7268(2)	0.33011(13)	0.16546(10)	0.1090(9)
**F3**	-0.0016(3)	0.51481(11)	0.19970(13)	0.1415(13)
**F4**	-0.2049(3)	0.00584(15)	0.43170(14)	0.1561(14)
**F5**	0.4995(3)	-0.04445(13)	0.26097(10)	0.1258(11)
**F6**	0.6435(2)	0.08292(11)	0.57318(8)	0.0928(8)
**F7**	-0.1698(2)	0.43304(12)	0.52060(11)	0.1188(10)
**F8**	0.6625(3)	0.28176(13)	0.62373(10)	0.1168(10)
**F9**	0.5983(2)	0.55570(9)	0.39678(8)	0.0868(7)
**P1**	0.21586(8)	0.29658(3)	0.23589(3)	0.04168(19)
**P2**	0.33489(8)	0.36175(3)	0.46354(3)	0.04096(19)
**P3**	0.30295(8)	0.09960(3)	0.40224(3)	0.04291(19)
**Pd1**	0.28503(2)	0.18829(2)	0.38081(2)	0.03902(7)
**Pd2**	0.24410(2)	0.27182(2)	0.31178(2)	0.03808(7)
**Pd3**	0.30669(2)	0.29915(2)	0.40646(2)	0.03834(7)
**S1**	0.21673(8)	0.18035(3)	0.30515(3)	0.04388(19)
**S2**	0.30075(8)	0.35367(3)	0.34333(3)	0.04332(19)
**S3**	0.33760(8)	0.22475(3)	0.45171(3)	0.04542(19)
**S4**	0.7040(2)	0.25176(11)	0.39393(8)	0.0929(10)
**C73**	0.8146(3)	0.25124(19)	0.34707(12)	0.183(9)
**F10**	0.7900(5)	0.2100(3)	0.31793(18)	0.214(5)
**F11**	0.9371(3)	0.2465(3)	0.3633(2)	0.159(4)
**F12**	0.8057(6)	0.2968(3)	0.32214(16)	0.233(5)
**O1**	0.5800(2)	0.2571(2)	0.36900(15)	0.179(5)
**O2**	0.7293(4)	0.19990(16)	0.41580(18)	0.199(4)
**O3**	0.7472(5)	0.29855(18)	0.42059(16)	0.169(5)
**S5**	0.8146(4)	0.2310(3)	0.35308(17)	0.109(2)
**C74**	0.7032(7)	0.2802(3)	0.3769(3)	0.083(5)
**F13**	0.7329(12)	0.3313(2)	0.3668(6)	0.247(9)
**F14**	0.7049(13)	0.2769(6)	0.4228(3)	0.250(11)
**F15**	0.5818(6)	0.2720(5)	0.3605(5)	0.213(11)
**O4**	0.9371(5)	0.2471(5)	0.3751(4)	0.260(19)
**O5**	0.7654(10)	0.1797(2)	0.3680(4)	0.204(9)
**O6**	0.7972(11)	0.2415(5)	0.30424(17)	0.185(12)

Table 2 Anisotropic Displacement Parameters for 1-OTf

Atom	U_{11}	U_{22}	U_{33}	U_{23}	U_{13}	U_{12}
**C1**	0.0428(18)	0.0507(19)	0.0433(18)	0.0004(15)	0.0000(14)	-0.0018(15)
**C2**	0.059(2)	0.076(3)	0.053(2)	-0.0115(19)	0.0106(18)	-0.0102(19)
**C3**	0.083(3)	0.088(3)	0.061(3)	-0.027(2)	0.007(2)	-0.012(2)
**C4**	0.062(3)	0.068(3)	0.068(3)	-0.016(2)	-0.012(2)	-0.005(2)
**C5**	0.053(2)	0.081(3)	0.073(3)	-0.007(2)	0.000(2)	-0.021(2)
**C6**	0.051(2)	0.079(3)	0.049(2)	-0.0063(18)	0.0084(17)	-0.0088(19)
**C7**	0.0410(18)	0.0508(19)	0.0428(19)	0.0029(14)	0.0061(14)	-0.0010(14)
**C8**	0.045(2)	0.077(2)	0.062(2)	0.0136(19)	0.0054(17)	0.0060(18)
**C9**	0.041(2)	0.112(4)	0.090(3)	0.018(3)	0.008(2)	0.006(2)
**C10**	0.050(2)	0.090(3)	0.084(3)	-0.010(2)	0.030(2)	-0.014(2)
**C11**	0.063(3)	0.093(3)	0.060(3)	0.008(2)	0.021(2)	-0.014(2)
**C12**	0.0447(19)	0.055(2)	0.0407(18)	0.0071(15)	0.0033(14)	0.0031(16)
**C13**	0.050(2)	0.058(2)	0.052(2)	0.0120(16)	0.0008(16)	0.0004(17)
**C14**	0.067(3)	0.054(2)	0.066(3)	0.0109(18)	0.000(2)	0.0007(19)
**C15**	0.082(3)	0.062(3)	0.109(4)	0.006(2)	-0.015(3)	0.024(2)
**C16**	0.073(3)	0.082(3)	0.123(4)	0.008(3)	-0.031(3)	0.022(3)
**C17**	0.053(2)	0.069(3)	0.082(3)	0.001(2)	-0.014(2)	0.0084(19)
**C18**	0.048(2)	0.0461(19)	0.060(2)	0.0059(15)	0.0082(16)	-0.0034(16)
**C19**	0.051(2)	0.058(2)	0.107(3)	0.011(2)	0.021(2)	0.0050(19)
**C20**	0.048(3)	0.102(4)	0.154(5)	0.017(3)	0.030(3)	0.003(3)
**C21**	0.057(3)	0.098(4)	0.142(5)	0.026(3)	0.018(3)	-0.024(3)
**C22**	0.075(3)	0.064(3)	0.132(4)	0.028(3)	0.009(3)	-0.023(2)
**C23**	0.058(2)	0.051(2)	0.108(3)	0.014(2)	0.005(2)	-0.0024(18)
**C24**	0.049(2)	0.0336(16)	0.058(2)	0.0050(14)	0.0063(16)	0.0032(14)
**C25**	0.060(2)	0.071(2)	0.056(2)	0.0052(18)	0.0062(18)	0.0194(19)
**C26**	0.076(3)	0.089(3)	0.080(3)	-0.002(2)	0.012(2)	0.042(2)
**C27**	0.094(3)	0.070(3)	0.075(3)	-0.013(2)	0.017(3)	0.031(2)
**C28**	0.087(3)	0.060(2)	0.071(3)	-0.018(2)	-0.002(2)	0.009(2)
**C29**	0.054(2)	0.048(2)	0.088(3)	-0.0128(19)	0.000(2)	0.0081(17)
**C30**	0.053(2)	0.0376(17)	0.051(2)	0.0058(14)	0.0056(16)	0.0044(15)
**C31**	0.060(2)	0.052(2)	0.065(3)	0.0081(18)	0.0039(19)	-0.0010(17)
**C32**	0.078(3)	0.070(3)	0.058(3)	0.0160(19)	0.004(2)	0.009(2)
**C33**	0.068(3)	0.068(2)	0.054(2)	0.0024(19)	-0.007(2)	0.027(2)
**C34**	0.050(2)	0.078(3)	0.074(3)	-0.001(2)	-0.004(2)	0.0059(19)
**C35**	0.053(2)	0.062(2)	0.062(2)	0.0089(18)	0.0037(18)	0.0024(18)
**C36**	0.0463(19)	0.0451(18)	0.0462(19)	-0.0044(14)	0.0059(15)	0.0013(15)

**C37**	0.048(2)	0.068(2)	0.062(2)	-0.0116(18)	0.0049(17)	0.0033(18)
**C38**	0.044(2)	0.086(3)	0.100(3)	-0.010(3)	0.005(2)	0.002(2)
**C39**	0.054(3)	0.077(3)	0.099(3)	-0.001(3)	0.029(2)	0.015(2)
**C40**	0.087(3)	0.079(3)	0.072(3)	-0.015(2)	0.031(2)	0.009(2)
**C41**	0.060(2)	0.065(2)	0.061(2)	-0.0138(18)	0.0085(18)	0.0005(19)
**C42**	0.0468(19)	0.0416(17)	0.0457(19)	-0.0006(14)	-0.0011(15)	-0.0004(14)
**C43**	0.055(2)	0.086(3)	0.066(3)	0.019(2)	0.006(2)	-0.001(2)
**C44**	0.084(3)	0.097(3)	0.067(3)	0.030(2)	0.009(2)	-0.005(3)
**C45**	0.076(3)	0.084(3)	0.066(3)	0.026(2)	-0.025(2)	-0.006(2)
**C46**	0.054(3)	0.130(4)	0.083(3)	0.040(3)	-0.007(2)	0.008(3)
**C47**	0.059(3)	0.115(4)	0.060(3)	0.025(2)	0.006(2)	0.006(2)
**C48**	0.051(2)	0.054(2)	0.067(2)	-0.0011(17)	0.0069(18)	-0.0031(17)
**C49**	0.058(2)	0.071(3)	0.076(3)	0.008(2)	0.009(2)	-0.014(2)
**C50**	0.072(3)	0.052(2)	0.056(2)	0.0119(17)	-0.0100(19)	-0.020(2)
**C51**	0.087(3)	0.044(2)	0.064(3)	-0.0006(17)	-0.002(2)	-0.007(2)
**C52**	0.063(2)	0.048(2)	0.055(2)	-0.0020(16)	0.0022(17)	-0.0008(17)
**C53**	0.0461(18)	0.0416(18)	0.0438(18)	-0.0051(14)	-0.0042(14)	-0.0033(14)
**C54**	0.054(2)	0.0363(16)	0.0425(19)	-0.0024(13)	0.0104(15)	-0.0015(14)
**C55**	0.057(2)	0.057(2)	0.050(2)	-0.0100(16)	0.0045(17)	-0.0033(17)
**C56**	0.051(2)	0.067(2)	0.077(3)	-0.008(2)	0.0080(19)	0.0002(18)
**C57**	0.069(3)	0.060(2)	0.075(3)	-0.015(2)	0.033(2)	-0.003(2)
**C58**	0.081(3)	0.072(3)	0.060(2)	-0.021(2)	0.016(2)	-0.003(2)
**C59**	0.062(2)	0.057(2)	0.058(2)	-0.0141(18)	0.0046(18)	0.0000(18)
**C60**	0.054(2)	0.0443(18)	0.0379(18)	0.0006(14)	0.0014(14)	0.0008(15)
**C61**	0.059(2)	0.048(2)	0.070(3)	-0.0045(17)	0.0041(18)	0.0006(18)
**C62**	0.053(2)	0.071(3)	0.079(3)	-0.007(2)	0.0031(19)	0.004(2)
**C63**	0.064(3)	0.063(3)	0.066(3)	0.0011(19)	0.0086(19)	0.022(2)
**C64**	0.069(3)	0.0419(19)	0.064(2)	0.0002(16)	0.0039(19)	0.0063(18)
**C65**	0.057(2)	0.0431(19)	0.052(2)	0.0019(15)	-0.0018(16)	0.0037(16)
**C66**	0.058(2)	0.0391(17)	0.0430(19)	0.0011(14)	0.0050(15)	-0.0053(15)
**C67**	0.068(3)	0.074(3)	0.050(2)	0.0111(18)	0.0048(19)	-0.002(2)
**C68**	0.060(2)	0.091(3)	0.070(3)	0.002(2)	0.010(2)	-0.002(2)
**C69**	0.080(3)	0.093(3)	0.052(2)	-0.001(2)	0.018(2)	-0.023(2)
**C70**	0.093(3)	0.095(3)	0.044(2)	0.016(2)	0.008(2)	-0.016(3)
**C71**	0.070(3)	0.071(2)	0.043(2)	0.0044(18)	0.0021(18)	0.000(2)
**C72**	0.054(2)	0.076(2)	0.051(2)	0.0104(19)	0.0045(17)	-0.0045(19)
**Cl1**	0.0938(9)	0.1222(10)	0.1271(11)	-0.0417(8)	0.0550(8)	0.0056(8)
**Cl2**	0.0843(8)	0.0858(8)	0.1270(11)	0.0021(7)	0.0060(7)	0.0411(6)
**Cl3**	0.1006(10)	0.1960(16)	0.0798(9)	0.0100(9)	0.0360(7)	-0.0428(10)
**F1**	0.0907(18)	0.0991(18)	0.0995(19)	-0.0374(15)	-0.0145(14)	-0.0240(15)
**F2**	0.0533(14)	0.162(3)	0.116(2)	-0.0034(19)	0.0352(14)	-0.0252(15)
**F3**	0.123(2)	0.0715(18)	0.221(4)	0.0138(19)	-0.052(2)	0.0389(17)
**F4**	0.0717(19)	0.155(3)	0.244(4)	0.055(3)	0.033(2)	-0.0413(19)
**F5**	0.143(3)	0.136(2)	0.100(2)	-0.0442(18)	0.0176(18)	0.061(2)
**F6**	0.0936(17)	0.1120(19)	0.0690(16)	-0.0025(14)	-0.0204(13)	0.0340(15)
**F7**	0.0700(16)	0.124(2)	0.169(3)	-0.013(2)	0.0527(18)	0.0275(16)
**F8**	0.104(2)	0.142(2)	0.098(2)	0.0560(18)	-0.0384(17)	-0.0095(18)
**F9**	0.1073(18)	0.0716(15)	0.0791(16)	0.0194(12)	-0.0103(13)	-0.0395(13)
**P1**	0.0379(4)	0.0485(5)	0.0386(5)	0.0024(4)	0.0028(3)	-0.0001(4)
**P2**	0.0420(4)	0.0399(4)	0.0410(5)	-0.0048(3)	0.0033(3)	-0.0001(4)
**P3**	0.0424(5)	0.0365(4)	0.0503(5)	0.0037(4)	0.0066(4)	0.0018(3)
**Pd1**	0.04547(15)	0.03376(13)	0.03837(14)	0.00023(9)	0.00672(11)	0.00132(10)
**Pd2**	0.04168(14)	0.03696(13)	0.03574(14)	-0.00035(9)	0.00377(10)	0.00153(10)
**Pd3**	0.04349(14)	0.03428(13)	0.03735(14)	-0.00161(9)	0.00363(10)	0.00050(10)
**S1**	0.0486(5)	0.0403(4)	0.0429(5)	-0.0045(3)	0.0040(4)	-0.0021(4)
**S2**	0.0495(5)	0.0374(4)	0.0430(5)	0.0016(3)	0.0024(4)	-0.0001(3)
**S3**	0.0556(5)	0.0409(4)	0.0396(5)	0.0013(3)	0.0025(4)	0.0015(4)
**S4**	0.0733(14)	0.0957(19)	0.111(2)	-0.0141(15)	0.0130(12)	-0.0098(13)
**C73**	0.167(17)	0.137(12)	0.25(2)	0.005(13)	0.030(15)	-0.075(12)
**F10**	0.179(8)	0.308(12)	0.162(8)	-0.124(8)	0.068(7)	-0.105(8)
**F11**	0.064(5)	0.260(10)	0.152(6)	0.008(6)	-0.014(4)	-0.030(6)
**F12**	0.251(10)	0.247(10)	0.192(8)	0.098(8)	-0.052(7)	-0.119(9)
**O1**	0.049(6)	0.269(12)	0.217(12)	-0.098(9)	-0.012(6)	0.023(6)
**O2**	0.192(8)	0.146(7)	0.271(11)	0.080(7)	0.099(8)	0.006(6)
**O3**	0.139(6)	0.155(8)	0.214(11)	-0.085(7)	0.014(6)	-0.043(6)
**S5**	0.080(4)	0.154(5)	0.090(4)	-0.014(3)	-0.014(3)	0.030(3)
**C74**	0.068(10)	0.058(10)	0.125(15)	-0.024(10)	0.020(10)	-0.002(8)
**F13**	0.250(18)	0.161(14)	0.35(3)	-0.032(15)	0.131(18)	-0.009(12)
**F14**	0.30(2)	0.29(2)	0.171(16)	-0.170(16)	0.038(14)	-0.067(18)
**F15**	0.078(14)	0.229(18)	0.33(3)	-0.056(19)	0.008(15)	-0.015(12)
**O4**	0.097(16)	0.55(5)	0.139(15)	0.08(2)	0.034(12)	0.13(2)
**O5**	0.31(2)	0.078(8)	0.227(19)	0.074(10)	0.017(16)	0.056(11)
**O6**	0.125(13)	0.31(3)	0.115(12)	0.125(17)	-0.011(10)	0.046(15)

Table 3 Bond Lengths for 1-OTf					
**Atom**	**Atom**	**Length/Å**	**Atom**	**Atom**	**Length/Å**
C1	C2	1.387(4)	C45	C46	1.340(6)
C1	C6	1.378(4)	C45	F8	1.353(4)
C1	P1	1.823(3)	C46	C47	1.382(5)
C2	C3	1.391(5)	C48	C49	1.385(5)
C3	C4	1.359(6)	C48	C53	1.388(4)
C4	C5	1.360(5)	C49	C50	1.368(5)
C4	F1	1.353(4)	C50	C51	1.338(5)
C5	C6	1.386(5)	C50	F9	1.373(4)
C7	C8	1.376(5)	C51	C52	1.389(5)
C7	C72	1.398(4)	C52	C53	1.380(4)
C7	P1	1.820(3)	C53	P2	1.818(3)
C8	C9	1.390(5)	C54	C55	1.378(5)
C9	C10	1.347(6)	C54	C59	1.383(4)
C10	C11	1.359(6)	C54	S1	1.782(3)
C10	F2	1.358(4)	C55	C56	1.375(5)
C11	C72	1.383(5)	C56	C57	1.375(5)
C12	C13	1.387(5)	C57	C58	1.372(5)
C12	C17	1.375(5)	C57	Cl1	1.749(4)
C12	P1	1.828(3)	C58	C59	1.386(5)
C13	C14	1.376(5)	C60	C61	1.386(5)
C14	C15	1.348(5)	C60	C65	1.387(4)
C15	C16	1.353(6)	C60	S2	1.776(3)
C15	F3	1.356(5)	C61	C62	1.376(5)
C16	C17	1.389(6)	C62	C63	1.385(5)
C18	C19	1.373(5)	C63	C64	1.367(5)
C18	C23	1.387(5)	C63	Cl2	1.745(4)
C18	P3	1.819(3)	C64	C65	1.392(4)
C19	C20	1.390(5)	C66	C67	1.379(5)
C20	C21	1.353(6)	C66	C71	1.381(5)
C21	C22	1.357(6)	C66	S3	1.782(3)
C21	F4	1.362(5)	C67	C68	1.375(5)
C22	C23	1.377(5)	C68	C69	1.358(6)
C24	C25	1.379(5)	C69	C70	1.379(6)
C24	C29	1.366(5)	C69	Cl3	1.736(4)
C24	P3	1.826(3)	C70	C71	1.376(5)
C25	C26	1.399(5)	P1	Pd2	2.2926(8)
C26	C27	1.344(6)	P2	Pd3	2.2736(8)
C27	C28	1.357(6)	P3	Pd1	2.2804(8)
C27	F5	1.357(4)	Pd1	Pd2	2.8905(3)
C28	C29	1.392(5)	Pd1	Pd3	2.8411(3)
C30	C31	1.387(4)	Pd1	S1	2.2761(8)
C30	C35	1.388(5)	Pd1	S3	2.2836(8)
C30	P3	1.814(3)	Pd2	Pd3	2.8693(3)
C31	C32	1.388(5)	Pd2	S1	2.2822(8)
C32	C33	1.340(5)	Pd2	S2	2.2797(8)
C33	C34	1.385(5)	Pd3	S2	2.2764(8)
C33	F6	1.365(4)	Pd3	S3	2.2700(8)
C34	C35	1.383(5)	S4	C73	1.8515
C36	C37	1.387(5)	S4	O1	1.4450
C36	C41	1.394(5)	S4	O2	1.4457
C36	P2	1.812(3)	S4	O3	1.4457
C37	C38	1.387(5)	C73	F10	1.3386
C38	C39	1.344(6)	C73	F11	1.3382
C39	C40	1.359(6)	C73	F12	1.3381
C39	F7	1.354(4)	S5	C74	1.8512
C40	C41	1.390(5)	S5	O4	1.4452
C42	C43	1.368(5)	S5	O5	1.4450
C42	C47	1.369(5)	S5	O6	1.4450
C42	P2	1.821(3)	C74	F13	1.3386
C43	C44	1.380(5)	C74	F14	1.3387
C44	C45	1.342(6)	C74	F15	1.3384

Table 4 Bond Angles for 1-OTf							
**Atom**	**Atom**	**Atom**	**Angle/°**	**Atom**	**Atom**	**Atom**	**Angle/°**
C2	C1	P1	123.2(3)	C65	C60	S2	117.2(3)
C6	C1	C2	118.0(3)	C62	C61	C60	120.8(3)
C6	C1	P1	118.8(2)	C61	C62	C63	118.6(4)
C1	C2	C3	121.2(4)	C62	C63	Cl2	118.8(3)
C4	C3	C2	118.3(4)	C64	C63	C62	122.0(3)
C3	C4	C5	122.6(4)	C64	C63	Cl2	119.2(3)

F1	C4	C3	118.5(4)	C63	C64	C65	118.9(3)
F1	C4	C5	118.9(4)	C60	C65	C64	120.2(3)
C4	C5	C6	118.5(4)	C67	C66	C71	119.0(3)
C1	C6	C5	121.5(3)	C67	C66	S3	123.0(3)
C8	C7	C72	118.4(3)	C71	C66	S3	118.0(3)
C8	C7	P1	119.6(2)	C68	C67	C66	120.1(4)
C72	C7	P1	121.9(3)	C69	C68	C67	120.6(4)
C7	C8	C9	120.5(4)	C68	C69	C70	120.3(4)
C10	C9	C8	119.0(4)	C68	C69	Cl3	120.9(4)
C9	C10	C11	123.1(4)	C70	C69	Cl3	118.8(3)
C9	C10	F2	118.9(4)	C71	C70	C69	119.3(4)
F2	C10	C11	118.0(4)	C70	C71	C66	120.8(4)
C10	C11	C72	117.9(4)	C11	C72	C7	121.1(3)
C13	C12	P1	117.7(2)	C1	P1	C12	104.31(15)
C17	C12	C13	118.6(3)	C1	P1	Pd2	114.54(11)
C17	C12	P1	123.7(3)	C7	P1	C1	108.00(15)
C14	C13	C12	121.3(3)	C7	P1	C12	101.65(14)
C15	C14	C13	118.0(4)	C7	P1	Pd2	109.83(11)
C14	C15	C16	123.3(4)	C12	P1	Pd2	117.42(10)
C14	C15	F3	117.9(4)	C36	P2	C42	107.03(15)
C16	C15	F3	118.8(4)	C36	P2	C53	106.35(15)
C15	C16	C17	118.6(4)	C36	P2	Pd3	111.31(11)
C12	C17	C16	120.2(4)	C42	P2	Pd3	114.62(10)
C19	C18	C23	118.8(3)	C53	P2	C42	104.29(14)
C19	C18	P3	119.3(3)	C53	P2	Pd3	112.64(10)
C23	C18	P3	121.9(3)	C18	P3	C24	103.52(15)
C18	C19	C20	120.9(4)	C18	P3	Pd1	112.02(11)
C21	C20	C19	117.9(4)	C24	P3	Pd1	116.53(10)
C20	C21	C22	123.3(4)	C30	P3	C18	107.25(15)
C20	C21	F4	118.4(5)	C30	P3	C24	104.53(15)
C22	C21	F4	118.2(4)	C30	P3	Pd1	112.15(10)
C21	C22	C23	118.3(4)	P3	Pd1	Pd2	151.69(2)
C22	C23	C18	120.7(4)	P3	Pd1	Pd3	148.11(2)
C25	C24	P3	123.2(3)	P3	Pd1	S3	96.94(3)
C29	C24	C25	118.7(3)	Pd3	Pd1	Pd2	60.075(8)
C29	C24	P3	118.1(2)	S1	Pd1	P3	101.32(3)
C24	C25	C26	119.9(4)	S1	Pd1	Pd2	50.74(2)
C27	C26	C25	119.1(4)	S1	Pd1	Pd3	110.56(2)
C26	C27	C28	123.0(4)	S1	Pd1	S3	161.48(3)
C26	C27	F5	119.6(4)	S3	Pd1	Pd2	111.24(2)
F5	C27	C28	117.4(4)	S3	Pd1	Pd3	51.18(2)
C27	C28	C29	117.3(4)	P1	Pd2	Pd1	149.72(2)
C24	C29	C28	122.0(3)	P1	Pd2	Pd3	150.37(2)
C31	C30	C35	118.0(3)	Pd3	Pd2	Pd1	59.109(8)
C31	C30	P3	123.4(3)	S1	Pd2	P1	100.10(3)
C35	C30	P3	118.4(2)	S1	Pd2	Pd1	50.55(2)
C30	C31	C32	121.1(4)	S1	Pd2	Pd3	109.42(2)
C33	C32	C31	118.6(4)	S2	Pd2	P1	99.46(3)
C32	C33	C34	123.3(4)	S2	Pd2	Pd1	109.29(2)
C32	C33	F6	119.3(4)	S2	Pd2	Pd3	50.91(2)
F6	C33	C34	117.4(4)	S2	Pd2	S1	159.72(3)
C35	C34	C33	117.3(4)	P2	Pd3	Pd1	148.27(2)
C34	C35	C30	121.7(3)	P2	Pd3	Pd2	150.65(2)
C37	C36	C41	118.2(3)	P2	Pd3	S2	100.61(3)
C37	C36	P2	118.2(2)	Pd1	Pd3	Pd2	60.816(8)
C41	C36	P2	123.5(3)	S2	Pd3	Pd1	111.08(2)
C36	C37	C38	121.0(3)	S2	Pd3	Pd2	51.02(2)
C39	C38	C37	118.7(4)	S3	Pd3	P2	96.85(3)
C38	C39	C40	123.0(4)	S3	Pd3	Pd1	51.61(2)
C38	C39	F7	119.4(4)	S3	Pd3	Pd2	112.41(2)
F7	C39	C40	117.6(4)	S3	Pd3	S2	160.37(3)
C39	C40	C41	118.6(4)	C54	S1	Pd1	109.48(11)
C40	C41	C36	120.4(4)	C54	S1	Pd2	106.55(10)
C43	C42	C47	116.6(3)	Pd1	S1	Pd2	78.71(3)
C43	C42	P2	123.2(3)	C60	S2	Pd2	113.00(11)
C47	C42	P2	120.1(3)	C60	S2	Pd3	111.15(10)
C42	C43	C44	122.0(4)	Pd3	S2	Pd2	78.07(3)
C45	C44	C43	118.8(4)	C66	S3	Pd1	107.64(11)
C44	C45	F8	119.0(4)	C66	S3	Pd3	112.25(11)
C46	C45	C44	121.8(4)	Pd3	S3	Pd1	77.21(3)
C46	C45	F8	119.1(4)	O1	S4	C73	102.4
C45	C46	C47	118.7(4)	O1	S4	O2	115.6
C42	C47	C46	122.0(4)	O1	S4	O3	115.5
C49	C48	C53	121.1(3)	O2	S4	C73	102.4
C50	C49	C48	117.5(4)	O3	S4	C73	102.4

C49	C50	F9	117.4(4)	O3	S4	O2	115.5
C51	C50	C49	124.0(3)	F10	C73	S4	111.8
C51	C50	F9	118.6(4)	F11	C73	S4	111.9
C50	C51	C52	117.9(3)	F11	C73	F10	107.0
C53	C52	C51	121.3(3)	F12	C73	S4	111.9
C48	C53	P2	118.3(2)	F12	C73	F10	107.0
C52	C53	C48	118.2(3)	F12	C73	F11	107.0
C52	C53	P2	123.5(3)	O4	S5	C74	102.4
C55	C54	C59	119.6(3)	O5	S5	C74	102.4
C55	C54	S1	123.1(2)	O5	S5	O4	115.5
C59	C54	S1	117.4(3)	O5	S5	O6	115.5
C56	C55	C54	120.6(3)	O6	S5	C74	102.4
C57	C56	C55	119.0(4)	O6	S5	O4	115.5
C56	C57	Cl1	119.0(3)	F13	C74	S5	111.9
C58	C57	C56	121.7(3)	F13	C74	F14	106.9
C58	C57	Cl1	119.3(3)	F14	C74	S5	111.9
C57	C58	C59	118.7(4)	F15	C74	S5	111.9
C54	C59	C58	120.4(3)	F15	C74	F13	107.0
C61	C60	C65	119.5(3)	F15	C74	F14	107.0
C61	C60	S2	123.3(3)				

Table 5 Atomic Occupancy for 1-OTf

Atom	*Occupancy*
S4, C73, O1, O2, O3, F10, F11, F12	0.658(5)
S5, C74, O4, O5, O6, F13, F14, F15	0.342(5)

2-SbF_6 (CCDC 1410441)

Ortep of triangular cluster (top) highlighting $Pd_3[Ag(THF)_4(H_2O)]^{2+}$ core (down). Hydrogen atoms omitted for clarity. Thermal ellipsoids are shown at 50% probability.

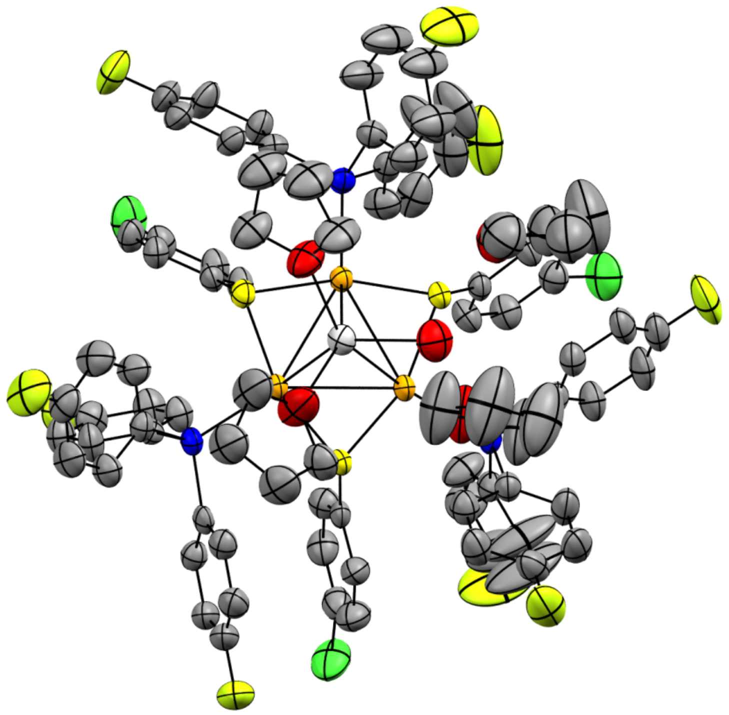

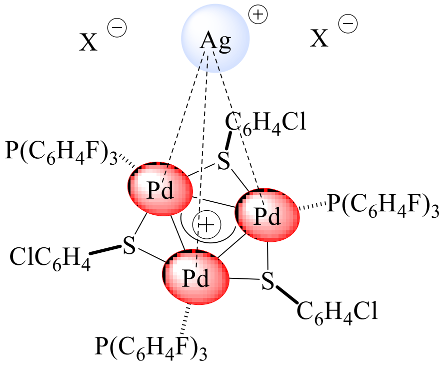

6: X = SbF_6

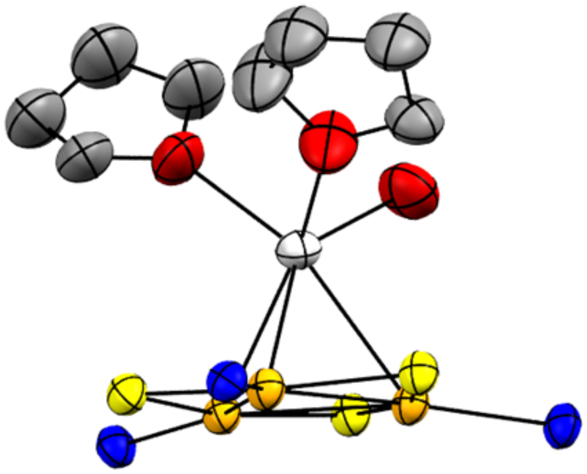

Pd_3 triangle is nearly equilateral, Pd-Pd-Pd angles range between 59.597(18)° and 60.403(18)°. Pd-Pd distances range between 2.8990(8) Å and 2.9166(7) Å. Ag-Pd_3 assembly exhibit a distorted trigonal pyramidal geometry, with Ag-Pd distances between 2.8091(8) Å and 2.8532(9) Å. Pd-Ag-Pd angles are between 61.42(2)° and 62.00(2)°. Ag-O from THF molecule are 2.379(7) Å and 2.384(7) Å. Ag-O from water molecule distance is 2.370(7) Å.

Table 1 Fractional Atomic Coordinates and Equivalent Isotropic Displacement Parameters for 2-SbF$_6$				
**Atom**	***x***	***y***	***z***	**U(eq)**
**C1**	0.8493(4)	0.3519(3)	0.04550(19)	0.0407(17)
**C2**	0.8199(4)	0.3576(4)	0.0768(2)	0.053(2)
**C3**	0.7621(5)	0.3812(4)	0.0798(3)	0.063(3)
**C4**	0.7335(5)	0.3987(5)	0.0507(3)	0.063(2)
**C5**	0.7591(5)	0.3946(5)	0.0197(3)	0.066(3)
**C6**	0.8181(4)	0.3710(5)	0.0169(2)	0.060(3)
**C7**	0.9335(4)	0.2886(4)	0.00228(18)	0.0409(17)
**C8**	0.9180(5)	0.2295(4)	-0.0028(2)	0.061(3)
**C9**	0.9228(6)	0.2033(5)	-0.0337(2)	0.078(3)
**C10**	0.9445(5)	0.2351(5)	-0.0607(2)	0.067(3)
**C11**	0.9596(5)	0.2930(5)	-0.0577(2)	0.069(3)
**C12**	0.9533(5)	0.3197(5)	-0.0264(2)	0.058(2)
**C13**	0.9742(4)	0.3887(4)	0.03994(16)	0.0370(15)
**C14**	1.0344(4)	0.3832(4)	0.0309(2)	0.0471(19)
**C15**	1.0720(5)	0.4315(4)	0.0290(2)	0.058(2)
**C16**	1.0480(4)	0.4853(4)	0.0362(2)	0.053(2)
**C17**	0.9889(4)	0.4939(4)	0.0456(2)	0.0512(19)
**C18**	0.9509(4)	0.4445(4)	0.04702(19)	0.0436(17)
**C19**	1.1712(4)	0.2947(4)	0.1732(2)	0.0465(19)
**C20**	1.1836(5)	0.3005(4)	0.1386(2)	0.058(2)
**C21**	1.2410(4)	0.3130(5)	0.1268(3)	0.066(3)
**C22**	1.2873(5)	0.3194(5)	0.1500(3)	0.065(3)
**C23**	1.2779(5)	0.3116(5)	0.1836(3)	0.072(3)
**C24**	1.2209(5)	0.2999(5)	0.1957(2)	0.070(3)
**C25**	1.1051(4)	0.2432(4)	0.22748(19)	0.0446(18)
**C26**	1.0917(5)	0.2652(5)	0.2594(2)	0.064(3)
**C27**	1.1004(5)	0.2328(5)	0.2884(2)	0.074(3)
**C28**	1.1243(5)	0.1770(5)	0.2850(2)	0.068(3)
**C29**	1.1379(4)	0.1525(5)	0.2536(2)	0.062(2)
**C30**	1.1278(4)	0.1864(4)	0.2250(2)	0.053(2)
**C31**	1.0704(4)	0.3559(4)	0.20352(19)	0.0492(19)
**C32**	1.0108(5)	0.3684(5)	0.2078(3)	0.077(3)
**C33**	0.9904(7)	0.4206(5)	0.2224(3)	0.087(4)
**C34**	1.0295(9)	0.4610(7)	0.2308(5)	0.125(6)
**C35**	1.0908(10)	0.4485(8)	0.2282(7)	0.201(12)
**C36**	1.1111(7)	0.3988(7)	0.2138(5)	0.163(9)
**C37**	0.7951(4)	0.0768(4)	0.1270(2)	0.0428(17)
**C38**	0.7374(4)	0.0872(4)	0.1394(2)	0.052(2)
**C39**	0.6850(4)	0.0769(5)	0.1194(2)	0.065(3)
**C40**	0.6942(5)	0.0570(5)	0.0869(2)	0.062(2)
**C41**	0.7514(5)	0.0456(4)	0.0735(2)	0.057(2)
**C42**	0.8008(4)	0.0565(4)	0.0935(2)	0.050(2)
**C43**	0.8991(4)	0.0185(4)	0.1527(2)	0.0486(18)
**C44**	0.9619(5)	0.0130(5)	0.1546(3)	0.068(3)
**C45**	0.9896(6)	-0.0421(5)	0.1569(4)	0.087(4)
**C46**	0.8922(6)	-0.0865(5)	0.1564(4)	0.111(5)
**C47**	0.8655(5)	-0.0327(5)	0.1532(4)	0.090(4)
**C48**	0.8348(4)	0.1032(4)	0.19471(19)	0.0488(19)
**C49**	0.8163(5)	0.1581(5)	0.2037(2)	0.065(3)
**C50**	0.7934(5)	0.1705(6)	0.2356(3)	0.081(3)
**C51**	0.9537(6)	-0.0897(5)	0.1590(4)	0.101(5)
**C52**	0.7923(6)	0.1276(8)	0.2580(3)	0.095(4)
**C53**	0.8296(7)	0.0584(7)	0.2180(3)	0.107(5)
**C54**	0.8073(8)	0.0702(8)	0.2505(3)	0.127(5)
**C55**	1.0549(6)	0.0284(5)	0.0717(4)	0.094(4)
**C56**	1.0253(7)	-0.0246(6)	0.0575(4)	0.107(5)
**C57**	0.9615(6)	-0.0065(6)	0.0501(4)	0.102(4)
**C58**	0.9656(6)	0.0593(5)	0.0469(3)	0.081(3)
**C59**	1.0831(7)	0.1743(7)	0.0150(3)	0.106(5)
**C60**	1.1131(6)	0.2133(6)	-0.0094(3)	0.092(4)
**C61**	1.1634(6)	0.2417(6)	0.0106(3)	0.091(4)
**C62**	1.1416(5)	0.2386(5)	0.0473(2)	0.069(3)
**C63**	1.1304(9)	0.0249(8)	0.1915(4)	0.130(5)
**C64**	1.1744(8)	-0.0087(7)	0.1412(4)	0.123(5)
**C65**	1.2615(8)	0.1246(10)	0.0765(4)	0.158(8)
**C66**	1.1790(12)	-0.0134(12)	0.1983(5)	0.202(10)
**C67**	1.2181(9)	-0.0213(8)	0.1702(5)	0.148(6)
**C68**	1.2823(7)	0.1595(8)	0.1292(4)	0.139(7)
**C69**	1.3287(9)	0.1194(10)	0.1226(5)	0.173(9)
**C70**	1.3171(9)	0.0982(11)	0.0883(5)	0.192(11)
**C71**	0.8044(4)	0.2125(4)	0.09254(19)	0.0430(18)
**C72**	0.7851(4)	0.2402(4)	0.1221(2)	0.051(2)
**C73**	0.7244(5)	0.2536(4)	0.1282(2)	0.057(2)

**C74**	0.6821(5)	0.2387(5)	0.1044(2)	0.059(2)
**C75**	0.6996(4)	0.2111(4)	0.0743(2)	0.055(2)
**C76**	0.7597(4)	0.1980(4)	0.0684(2)	0.0478(19)
**C77**	1.0192(4)	0.3863(4)	0.11954(17)	0.0378(15)
**C78**	0.9630(4)	0.4060(4)	0.1310(2)	0.054(2)
**C79**	0.9533(5)	0.4649(4)	0.1388(2)	0.059(2)
**C80**	1.0001(5)	0.5042(4)	0.1349(2)	0.061(2)
**C81**	1.0564(5)	0.4866(5)	0.1242(3)	0.068(3)
**C82**	1.0661(4)	0.4270(4)	0.1165(2)	0.052(2)
**C83**	0.9667(4)	0.1823(4)	0.21865(18)	0.0405(17)
**C84**	0.9368(4)	0.2342(4)	0.2254(2)	0.052(2)
**C85**	0.9233(4)	0.2480(4)	0.2591(2)	0.055(2)
**C86**	0.9367(4)	0.2092(4)	0.2844(2)	0.055(2)
**C87**	0.9630(5)	0.1566(5)	0.2776(2)	0.062(3)
**C88**	0.9791(4)	0.1423(4)	0.2442(2)	0.054(2)
**O1**	1.1207(3)	0.1352(3)	0.1256(2)	0.080(2)
**O2**	1.0140(4)	0.0761(3)	0.06887(19)	0.076(2)
**O3**	1.0896(4)	0.2013(4)	0.04727(18)	0.095(3)
**O4**	1.2368(4)	0.1540(5)	0.1045(2)	0.108(3)
**O5**	1.1277(5)	0.0261(4)	0.1559(2)	0.102(3)
**F1**	0.6756(3)	0.4211(3)	0.05313(17)	0.0850(19)
**F2**	0.9519(4)	0.2088(4)	-0.09096(15)	0.111(3)
**F3**	1.0854(3)	0.5334(3)	0.03472(16)	0.0789(18)
**F4**	1.3451(3)	0.3324(3)	0.13904(18)	0.097(2)
**F5**	1.1335(3)	0.1444(4)	0.31317(16)	0.106(2)
**F6**	1.0090(6)	0.5105(4)	0.2452(3)	0.202(6)
**F7**	0.6440(3)	0.0482(3)	0.06753(17)	0.097(2)
**F8**	0.9795(4)	-0.1439(3)	0.1628(3)	0.149(4)
**F9**	0.7705(4)	0.1375(5)	0.29024(15)	0.142(3)
**F10**	0.8559(3)	0.3390(3)	0.18015(18)	0.100(2)
**F11**	0.7348(3)	0.4174(4)	0.22867(19)	0.113(3)
**F12**	0.8403(4)	0.3684(4)	0.24385(18)	0.119(3)
**F13**	0.8342(4)	0.4492(4)	0.1971(2)	0.132(3)
**F14**	0.7520(4)	0.3868(4)	0.16460(18)	0.125(3)
**F15**	0.7590(4)	0.3073(4)	0.2118(2)	0.134(3)
**F16**	0.8354(4)	0.0862(4)	-0.0665(2)	0.131(3)
**F17**	0.7182(4)	0.0690(5)	-0.0692(2)	0.162(4)
**F18**	0.7168(4)	0.0765(8)	-0.0037(2)	0.235(5)
**F19**	0.8315(4)	0.0935(5)	-0.0011(2)	0.135(3)
**F20**	0.7679(6)	0.1612(5)	-0.0407(3)	0.181(4)
**F21**	0.7904(7)	0.0023(6)	-0.0319(4)	0.221(5)
**P1**	0.92612(10)	0.32356(9)	0.04353(4)	0.0376(4)
**P2**	1.09428(10)	0.28460(10)	0.18846(5)	0.0407(5)
**P3**	0.86304(9)	0.09040(10)	0.15178(5)	0.0391(4)
**S1**	0.88000(9)	0.18985(9)	0.08450(4)	0.0402(4)
**S2**	1.03445(9)	0.31399(9)	0.10707(4)	0.0388(4)
**S3**	0.99612(9)	0.16393(8)	0.17776(4)	0.0373(4)
**Cl1**	0.60524(13)	0.25436(16)	0.11146(8)	0.0893(10)
**Cl2**	0.98733(17)	0.57890(13)	0.14237(9)	0.0957(10)
**Cl3**	0.92370(16)	0.22908(15)	0.32647(6)	0.0907(10)
**Pd1**	0.94995(3)	0.26431(3)	0.08939(2)	0.03572(15)
**Pd2**	1.02440(3)	0.24755(2)	0.15011(2)	0.03527(15)
**Pd3**	0.92568(3)	0.16663(2)	0.13484(2)	0.03411(14)
**Ag2**	1.03354(3)	0.16848(3)	0.09545(2)	0.04426(16)
**Sb1**	0.79505(3)	0.37871(3)	0.20449(2)	0.06071(19)
**Sb2**	0.77532(4)	0.07999(5)	-0.03505(2)	0.0861(3)

Table 2 Anisotropic Displacement Parameters for 2-SbF$_6$

Atom	U_{11}	U_{22}	U_{33}	U_{23}	U_{13}	U_{12}
**C1**	0.044(4)	0.035(4)	0.043(3)	0.001(3)	-0.003(3)	0.003(3)
**C2**	0.049(4)	0.063(6)	0.047(4)	0.002(4)	0.001(3)	0.013(4)
**C3**	0.057(5)	0.072(7)	0.059(4)	-0.003(4)	0.005(3)	0.021(4)
**C4**	0.049(5)	0.067(6)	0.072(5)	0.001(4)	0.000(3)	0.010(4)
**C5**	0.055(5)	0.082(7)	0.061(4)	0.004(4)	-0.010(3)	0.016(5)
**C6**	0.054(5)	0.085(7)	0.042(4)	0.009(4)	-0.007(3)	0.014(4)
**C7**	0.038(4)	0.053(4)	0.031(3)	-0.003(3)	-0.002(3)	0.002(3)
**C8**	0.076(7)	0.064(5)	0.044(4)	-0.006(3)	0.007(4)	-0.016(4)
**C9**	0.111(9)	0.078(6)	0.047(4)	-0.020(4)	0.012(4)	-0.021(6)
**C10**	0.074(7)	0.090(6)	0.039(4)	-0.015(4)	0.003(4)	-0.009(5)
**C11**	0.092(8)	0.086(6)	0.030(4)	-0.003(4)	0.010(4)	-0.014(5)
**C12**	0.071(6)	0.066(5)	0.036(3)	0.001(3)	0.004(3)	-0.005(4)
**C13**	0.041(4)	0.051(4)	0.019(3)	0.005(2)	0.000(2)	0.000(3)

**C14**	0.044(4)	0.058(5)	0.040(4)	0.001(3)	0.004(3)	-0.001(3)
**C15**	0.054(5)	0.067(5)	0.053(5)	0.010(4)	-0.002(4)	-0.009(3)
**C16**	0.051(4)	0.053(4)	0.055(5)	0.012(3)	-0.008(3)	-0.012(3)
**C17**	0.050(4)	0.049(4)	0.054(5)	0.009(3)	-0.010(3)	-0.007(3)
**C18**	0.041(4)	0.051(4)	0.039(4)	-0.002(3)	0.000(3)	0.000(3)
**C19**	0.043(4)	0.055(5)	0.041(3)	0.005(3)	-0.004(3)	-0.002(3)
**C20**	0.055(5)	0.073(7)	0.045(4)	0.004(4)	0.001(3)	0.002(4)
**C21**	0.046(4)	0.092(8)	0.061(5)	0.019(5)	0.002(3)	0.004(4)
**C22**	0.051(5)	0.071(7)	0.074(5)	0.024(4)	0.002(3)	-0.001(4)
**C23**	0.045(5)	0.100(9)	0.072(5)	0.021(5)	-0.010(4)	-0.015(5)
**C24**	0.055(5)	0.107(9)	0.048(4)	0.011(4)	-0.009(3)	-0.017(5)
**C25**	0.039(4)	0.061(4)	0.033(3)	0.003(3)	-0.006(3)	-0.001(3)
**C26**	0.072(7)	0.083(6)	0.037(4)	0.003(3)	-0.005(4)	0.010(5)
**C27**	0.088(8)	0.104(6)	0.031(4)	0.012(4)	-0.009(4)	0.009(5)
**C28**	0.056(6)	0.099(6)	0.047(4)	0.022(4)	-0.012(4)	0.000(5)
**C29**	0.048(5)	0.083(6)	0.054(4)	0.024(4)	-0.009(3)	0.006(4)
**C30**	0.046(5)	0.071(5)	0.042(4)	0.006(3)	-0.006(3)	0.004(4)
**C31**	0.066(5)	0.048(4)	0.034(4)	-0.002(3)	-0.007(3)	-0.002(3)
**C32**	0.064(5)	0.059(6)	0.109(9)	-0.032(6)	-0.004(5)	0.002(4)
**C33**	0.103(8)	0.064(6)	0.096(8)	-0.028(6)	-0.002(6)	0.020(5)
**C34**	0.138(9)	0.078(7)	0.158(14)	-0.061(8)	0.012(8)	-0.003(6)
**C35**	0.142(9)	0.114(10)	0.35(3)	-0.151(16)	0.016(9)	-0.013(7)
**C36**	0.087(8)	0.107(8)	0.30(2)	-0.123(12)	0.017(8)	-0.022(6)
**C37**	0.043(4)	0.038(4)	0.048(3)	0.002(3)	0.001(3)	-0.008(3)
**C38**	0.044(4)	0.071(6)	0.041(4)	-0.007(4)	-0.004(3)	-0.005(4)
**C39**	0.037(4)	0.094(8)	0.064(4)	-0.010(4)	-0.009(3)	-0.012(4)
**C40**	0.057(5)	0.072(7)	0.058(4)	-0.005(4)	-0.016(3)	-0.020(4)
**C41**	0.064(5)	0.056(6)	0.049(4)	-0.005(4)	-0.008(3)	-0.018(4)
**C42**	0.046(4)	0.061(6)	0.041(3)	-0.007(3)	0.004(3)	-0.015(4)
**C43**	0.048(4)	0.050(4)	0.048(4)	0.004(3)	0.003(3)	-0.004(3)
**C44**	0.044(5)	0.056(5)	0.104(8)	0.003(5)	-0.002(4)	0.002(4)
**C45**	0.058(6)	0.046(5)	0.157(12)	0.005(5)	-0.006(6)	0.013(4)
**C46**	0.069(6)	0.049(5)	0.215(17)	0.029(7)	-0.016(7)	-0.002(4)
**C47**	0.054(6)	0.053(5)	0.163(12)	0.024(5)	-0.019(6)	-0.007(4)
**C48**	0.037(4)	0.073(5)	0.036(3)	0.004(3)	0.002(3)	-0.007(4)
**C49**	0.079(7)	0.066(5)	0.051(4)	-0.005(4)	0.019(4)	-0.012(4)
**C50**	0.058(6)	0.131(8)	0.054(4)	-0.018(4)	0.015(4)	-0.002(5)
**C51**	0.073(6)	0.043(5)	0.186(15)	0.017(6)	-0.014(6)	0.004(4)
**C52**	0.076(8)	0.162(9)	0.048(4)	0.007(5)	0.016(5)	0.019(6)
**C53**	0.140(12)	0.115(7)	0.066(5)	0.032(5)	0.043(6)	0.033(7)
**C54**	0.152(14)	0.170(9)	0.061(6)	0.033(5)	0.042(7)	0.047(8)
**C55**	0.095(7)	0.048(5)	0.139(11)	-0.021(6)	-0.011(7)	0.013(5)
**C56**	0.111(8)	0.063(6)	0.148(12)	-0.027(6)	-0.003(8)	0.007(5)
**C57**	0.100(8)	0.076(7)	0.131(11)	-0.036(6)	0.004(7)	-0.003(5)
**C58**	0.079(6)	0.073(6)	0.091(7)	-0.033(5)	0.003(5)	0.005(5)
**C59**	0.126(10)	0.146(10)	0.047(5)	-0.008(5)	0.014(5)	-0.048(8)
**C60**	0.110(9)	0.113(9)	0.053(5)	-0.005(5)	0.018(5)	-0.022(7)
**C61**	0.101(8)	0.112(9)	0.061(5)	-0.004(5)	0.025(5)	-0.022(7)
**C62**	0.072(6)	0.083(7)	0.051(5)	0.008(4)	0.012(4)	-0.001(5)
**C63**	0.165(12)	0.142(13)	0.083(7)	-0.002(7)	-0.023(7)	-0.006(10)
**C64**	0.143(11)	0.116(11)	0.109(8)	-0.005(7)	-0.005(7)	0.001(8)
**C65**	0.105(10)	0.27(2)	0.095(8)	-0.063(10)	-0.008(7)	0.060(12)
**C66**	0.218(16)	0.27(2)	0.119(9)	0.032(10)	-0.025(9)	0.073(16)
**C67**	0.167(11)	0.135(14)	0.143(10)	0.000(10)	-0.031(9)	0.007(9)
**C68**	0.115(10)	0.193(16)	0.108(9)	-0.050(10)	-0.044(8)	0.064(10)
**C69**	0.127(11)	0.230(19)	0.162(12)	-0.076(12)	-0.051(10)	0.080(12)
**C70**	0.126(12)	0.29(2)	0.159(12)	-0.100(14)	-0.027(9)	0.083(15)
**C71**	0.044(4)	0.049(5)	0.035(3)	0.008(3)	-0.004(3)	0.001(3)
**C72**	0.055(5)	0.062(6)	0.035(3)	-0.003(3)	-0.003(3)	0.005(4)
**C73**	0.059(5)	0.068(6)	0.044(4)	-0.001(4)	-0.001(3)	0.014(4)
**C74**	0.055(4)	0.071(6)	0.051(4)	0.011(4)	-0.001(3)	0.008(4)
**C75**	0.038(4)	0.075(6)	0.052(4)	0.006(4)	-0.006(3)	-0.001(4)
**C76**	0.049(4)	0.056(5)	0.038(4)	0.002(3)	-0.006(3)	0.003(3)
**C77**	0.040(4)	0.047(4)	0.027(3)	0.004(3)	-0.009(3)	-0.003(3)
**C78**	0.058(5)	0.056(5)	0.049(4)	0.000(4)	0.006(4)	0.000(3)
**C79**	0.068(5)	0.057(5)	0.053(5)	-0.010(4)	-0.004(4)	0.015(4)
**C80**	0.079(5)	0.050(5)	0.055(5)	-0.004(4)	-0.017(4)	0.003(4)
**C81**	0.074(6)	0.051(5)	0.079(6)	-0.005(4)	-0.012(4)	-0.007(4)
**C82**	0.053(5)	0.046(4)	0.056(5)	0.001(3)	-0.009(4)	-0.005(3)
**C83**	0.042(4)	0.050(4)	0.030(3)	0.003(3)	-0.005(3)	-0.001(3)
**C84**	0.061(6)	0.051(5)	0.043(4)	0.008(3)	0.003(3)	0.012(4)
**C85**	0.056(6)	0.062(5)	0.046(4)	-0.004(3)	0.009(3)	0.011(4)
**C86**	0.054(5)	0.065(5)	0.044(4)	-0.002(3)	0.002(3)	0.001(4)
**C87**	0.085(7)	0.069(5)	0.033(4)	0.009(3)	0.003(4)	0.009(5)
**C88**	0.067(6)	0.056(5)	0.040(3)	0.008(3)	-0.002(3)	0.008(4)

**O1**	0.063(4)	0.089(5)	0.089(5)	0.003(4)	-0.005(4)	0.020(4)
**O2**	0.078(5)	0.060(4)	0.089(5)	-0.027(3)	-0.011(4)	0.006(3)
**O3**	0.095(5)	0.138(7)	0.053(3)	-0.007(4)	0.025(3)	-0.046(5)
**O4**	0.074(6)	0.182(9)	0.069(5)	-0.020(5)	0.001(4)	0.032(6)
**O5**	0.126(7)	0.107(7)	0.073(5)	0.000(4)	-0.012(5)	-0.003(5)
**F1**	0.051(3)	0.095(5)	0.109(5)	0.001(4)	0.002(3)	0.020(3)
**F2**	0.145(7)	0.136(6)	0.051(3)	-0.041(4)	0.018(4)	-0.031(5)
**F3**	0.081(4)	0.066(4)	0.090(4)	0.015(3)	-0.013(3)	-0.028(3)
**F4**	0.041(3)	0.140(6)	0.110(5)	0.045(4)	0.006(3)	-0.007(3)
**F5**	0.100(5)	0.149(6)	0.068(4)	0.058(4)	-0.009(3)	0.022(5)
**F6**	0.224(10)	0.087(6)	0.294(14)	-0.117(8)	0.041(10)	0.004(6)
**F7**	0.068(4)	0.131(6)	0.092(4)	-0.022(4)	-0.032(3)	-0.022(4)
**F8**	0.113(6)	0.056(4)	0.278(12)	0.014(6)	-0.030(7)	0.020(4)
**F9**	0.111(6)	0.274(10)	0.041(3)	0.001(4)	0.024(3)	0.050(6)
**F10**	0.096(5)	0.114(5)	0.089(4)	0.001(4)	0.014(4)	0.025(4)
**F11**	0.099(5)	0.135(6)	0.106(5)	-0.021(4)	0.024(4)	0.028(4)
**F12**	0.130(6)	0.144(7)	0.084(4)	0.003(4)	-0.016(4)	0.035(5)
**F13**	0.153(7)	0.101(5)	0.143(7)	0.005(5)	0.019(5)	-0.012(5)
**F14**	0.123(6)	0.169(7)	0.082(4)	-0.004(4)	-0.021(4)	0.051(5)
**F15**	0.153(7)	0.110(5)	0.138(6)	0.008(5)	0.028(6)	-0.021(5)
**F16**	0.121(6)	0.171(8)	0.102(5)	-0.010(5)	0.022(4)	-0.020(5)
**F17**	0.125(6)	0.269(11)	0.091(5)	0.042(6)	-0.025(5)	-0.051(6)
**F18**	0.085(6)	0.518(16)	0.103(6)	0.062(8)	-0.016(4)	-0.015(7)
**F19**	0.089(5)	0.222(9)	0.094(5)	0.023(5)	-0.013(4)	-0.018(5)
**F20**	0.211(10)	0.186(7)	0.147(8)	-0.007(6)	-0.027(7)	0.054(6)
**F21**	0.245(11)	0.174(7)	0.245(11)	0.063(7)	-0.051(9)	-0.048(6)
**P1**	0.0427(11)	0.0444(12)	0.0257(8)	0.0032(7)	-0.0022(7)	0.0010(9)
**P2**	0.0430(12)	0.0489(13)	0.0303(9)	-0.0019(8)	-0.0085(8)	-0.0045(9)
**P3**	0.0344(11)	0.0468(12)	0.0360(9)	0.0028(8)	0.0015(7)	-0.0062(9)
**S1**	0.0432(11)	0.0467(12)	0.0308(8)	-0.0007(7)	-0.0030(7)	-0.0010(9)
**S2**	0.0435(11)	0.0439(11)	0.0290(8)	0.0040(7)	-0.0027(7)	-0.0061(9)
**S3**	0.0418(11)	0.0412(11)	0.0287(8)	0.0040(7)	-0.0036(7)	-0.0013(8)
**Cl1**	0.0544(17)	0.134(3)	0.0797(18)	0.0063(17)	0.0034(13)	0.0252(16)
**Cl2**	0.121(3)	0.0577(18)	0.109(2)	-0.0320(16)	-0.034(2)	0.0142(17)
**Cl3**	0.107(2)	0.123(3)	0.0425(12)	-0.0139(14)	0.0132(13)	0.0113(19)
**Pd1**	0.0398(3)	0.0410(3)	0.0264(2)	0.0026(2)	-0.0042(2)	-0.0020(3)
**Pd2**	0.0404(3)	0.0393(3)	0.0261(2)	0.0018(2)	-0.0045(2)	-0.0023(2)
**Pd3**	0.0355(3)	0.0395(3)	0.0273(3)	0.0011(2)	-0.0009(2)	-0.0019(2)
**Ag2**	0.0441(4)	0.0504(4)	0.0383(3)	-0.0043(2)	0.0059(2)	0.0020(3)
**Sb1**	0.0612(4)	0.0698(5)	0.0512(3)	0.0070(3)	0.0049(3)	0.0120(3)
**Sb2**	0.0666(5)	0.1350(8)	0.0567(4)	0.0177(4)	-0.0014(3)	-0.0039(5)

Table 3 Bond Lengths for 2-SbF_6

Atom	Atom	Length/Å	Atom	Atom	Length/Å
C1	C2	1.396(11)	C55	O2	1.410(12)
C1	C6	1.386(11)	C56	C57	1.485(17)
C1	P1	1.802(8)	C57	C58	1.502(16)
C2	C3	1.379(12)	C58	O2	1.418(12)
C3	C4	1.361(13)	C59	C60	1.464(15)
C4	C5	1.346(13)	C59	O3	1.416(13)
C4	F1	1.369(11)	C60	C61	1.500(16)
C5	C6	1.403(13)	C61	C62	1.522(13)
C7	C8	1.398(12)	C62	O3	1.417(12)
C7	C12	1.399(11)	C63	C66	1.40(2)
C7	P1	1.814(8)	C63	O5	1.401(15)
C8	C9	1.360(12)	C64	C67	1.52(2)
C9	C10	1.368(14)	C64	O5	1.416(17)
C10	C11	1.360(14)	C65	C70	1.43(2)
C10	F2	1.344(10)	C65	O4	1.397(15)
C11	C12	1.382(12)	C66	C67	1.41(2)
C13	C14	1.371(11)	C68	C69	1.387(19)
C13	C18	1.392(11)	C68	O4	1.398(15)
C13	P1	1.821(8)	C69	C70	1.45(2)
C14	C15	1.373(12)	C71	C72	1.386(11)
C15	C16	1.361(13)	C71	C76	1.401(11)
C16	C17	1.359(13)	C71	S1	1.762(9)
C16	F3	1.365(10)	C72	C73	1.384(12)
C17	C18	1.396(12)	C73	C74	1.359(13)
C19	C20	1.394(11)	C74	C75	1.393(13)
C19	C24	1.406(12)	C74	Cl1	1.741(11)
C19	P2	1.801(9)	C75	C76	1.368(12)
C20	C21	1.369(13)	C77	C78	1.383(11)

C21	C22	1.371(14)	C77	C82	1.386(11)
C22	C23	1.350(14)	C77	S2	1.747(8)
C22	F4	1.369(11)	C78	C79	1.389(12)
C23	C24	1.361(13)	C79	C80	1.365(14)
C25	C26	1.385(11)	C80	C81	1.361(15)
C25	C30	1.386(12)	C80	Cl2	1.745(11)
C25	P2	1.817(8)	C81	C82	1.405(13)
C26	C27	1.369(12)	C83	C84	1.374(11)
C27	C28	1.376(15)	C83	C88	1.383(11)
C28	C29	1.390(14)	C83	S3	1.783(8)
C28	F5	1.348(10)	C84	C85	1.393(11)
C29	C30	1.381(11)	C85	C86	1.363(12)
C31	C32	1.345(14)	C86	C87	1.353(13)
C31	C36	1.381(15)	C86	Cl3	1.738(9)
C31	P2	1.803(9)	C87	C88	1.397(12)
C32	C33	1.390(14)	O1	Ag2	2.370(7)
C33	C34	1.297(18)	O2	Ag2	2.384(7)
C34	C35	1.37(2)	O3	Ag2	2.379(7)
C34	F6	1.337(15)	F10	Sb1	1.871(7)
C35	C36	1.340(19)	F11	Sb1	1.847(7)
C37	C38	1.374(11)	F12	Sb1	1.853(7)
C37	C42	1.402(11)	F13	Sb1	1.840(8)
C37	P3	1.805(9)	F14	Sb1	1.840(7)
C38	C39	1.408(11)	F15	Sb1	1.827(8)
C39	C40	1.372(13)	F16	Sb2	1.811(8)
C40	C41	1.383(14)	F17	Sb2	1.852(8)
C40	F7	1.353(10)	F18	Sb2	1.781(10)
C41	C42	1.358(11)	F19	Sb2	1.841(8)
C43	C44	1.382(13)	F20	Sb2	1.864(12)
C43	C47	1.377(13)	F21	Sb2	1.800(14)
C43	P3	1.813(9)	P1	Pd1	2.3107(19)
C44	C45	1.392(14)	P2	Pd2	2.3071(19)
C45	C51	1.339(16)	P3	Pd3	2.307(2)
C46	C47	1.360(15)	S1	Pd1	2.289(2)
C46	C51	1.352(17)	S1	Pd3	2.2806(18)
C48	C49	1.358(13)	S2	Pd1	2.275(2)
C48	C53	1.373(14)	S2	Pd2	2.2788(19)
C48	P3	1.822(8)	S3	Pd2	2.2748(19)
C49	C50	1.382(12)	S3	Pd3	2.2873(18)
C50	C52	1.314(17)	Pd1	Pd2	2.9166(7)
C51	F8	1.362(13)	Pd1	Pd3	2.8990(8)
C52	C54	1.38(2)	Pd1	Ag2	2.8532(9)
C52	F9	1.374(12)	Pd2	Pd3	2.8990(8)
C53	C54	1.394(16)	Pd2	Ag2	2.8091(8)
C55	C56	1.476(16)	Pd3	Ag2	2.8237(8)

Table 5 Bond Angles for 2-SbF_6

Atom	Atom	Atom	Angle/°	Atom	Atom	Atom	Angle/°
C2	C1	P1	120.0(6)	C85	C86	Cl3	119.5(8)
C6	C1	C2	117.5(8)	C87	C86	C85	121.1(8)
C6	C1	P1	122.4(7)	C87	C86	Cl3	119.3(7)
C3	C2	C1	122.2(8)	C86	C87	C88	119.9(9)
C4	C3	C2	117.7(9)	C83	C88	C87	118.9(9)
C3	C4	F1	118.4(9)	C55	O2	C58	108.4(8)
C5	C4	C3	123.4(10)	C55	O2	Ag2	121.9(7)
C5	C4	F1	118.2(9)	C58	O2	Ag2	129.6(6)
C4	C5	C6	118.7(9)	C59	O3	C62	109.9(8)
C1	C6	C5	120.6(9)	C59	O3	Ag2	121.7(7)
C8	C7	C12	116.5(8)	C62	O3	Ag2	126.9(6)
C8	C7	P1	121.7(6)	C65	O4	C68	108.3(11)
C12	C7	P1	121.8(7)	C63	O5	C64	111.6(12)
C9	C8	C7	121.9(9)	C1	P1	C7	106.1(4)
C8	C9	C10	119.3(10)	C1	P1	C13	104.6(4)
C11	C10	C9	122.0(9)	C1	P1	Pd1	112.6(3)
F2	C10	C9	119.6(10)	C7	P1	C13	103.6(3)
F2	C10	C11	118.4(9)	C7	P1	Pd1	115.1(3)
C10	C11	C12	118.3(9)	C13	P1	Pd1	113.8(2)
C11	C12	C7	122.0(9)	C19	P2	C25	103.1(4)
C14	C13	C18	119.1(8)	C19	P2	C31	105.4(4)
C14	C13	P1	120.0(7)	C19	P2	Pd2	116.6(3)
C18	C13	P1	120.9(6)	C25	P2	Pd2	116.8(3)
C13	C14	C15	121.1(9)	C31	P2	C25	103.0(4)
C16	C15	C14	118.4(10)	C31	P2	Pd2	110.5(3)
C15	C16	F3	118.6(9)	C37	P3	C43	102.4(4)

C17	C16	C15	123.5(9)	C37	P3	C48	104.4(4)
C17	C16	F3	117.9(9)	C37	P3	Pd3	117.5(3)
C16	C17	C18	117.5(9)	C43	P3	C48	105.8(4)
C13	C18	C17	120.4(8)	C43	P3	Pd3	115.1(3)
C20	C19	C24	117.0(8)	C48	P3	Pd3	110.5(3)
C20	C19	P2	121.4(7)	C71	S1	Pd1	113.4(3)
C24	C19	P2	121.6(7)	C71	S1	Pd3	108.9(2)
C21	C20	C19	122.1(9)	Pd3	S1	Pd1	78.75(6)
C20	C21	C22	118.2(9)	C77	S2	Pd1	113.4(3)
C23	C22	C21	121.9(10)	C77	S2	Pd2	113.3(2)
C23	C22	F4	118.5(9)	Pd1	S2	Pd2	79.66(7)
F4	C22	C21	119.6(9)	C83	S3	Pd2	109.5(3)
C22	C23	C24	120.3(10)	C83	S3	Pd3	114.6(3)
C23	C24	C19	120.5(9)	Pd2	S3	Pd3	78.91(6)
C26	C25	C30	118.5(8)	P1	Pd1	Pd2	147.33(6)
C26	C25	P2	123.6(7)	P1	Pd1	Pd3	152.41(6)
C30	C25	P2	118.0(6)	P1	Pd1	Ag2	130.87(6)
C27	C26	C25	122.1(10)	S1	Pd1	P1	102.35(7)
C26	C27	C28	117.9(10)	S1	Pd1	Pd2	110.23(5)
C27	C28	C29	122.4(9)	S1	Pd1	Pd3	50.50(5)
F5	C28	C27	119.0(10)	S1	Pd1	Ag2	82.63(6)
F5	C28	C29	118.6(10)	S2	Pd1	P1	97.67(7)
C30	C29	C28	117.9(10)	S2	Pd1	S1	159.31(7)
C29	C30	C25	121.2(9)	S2	Pd1	Pd2	50.23(5)
C32	C31	C36	116.1(11)	S2	Pd1	Pd3	109.86(5)
C32	C31	P2	120.8(8)	S2	Pd1	Ag2	80.32(6)
C36	C31	P2	122.9(9)	Pd3	Pd1	Pd2	59.797(18)
C31	C32	C33	122.9(11)	Ag2	Pd1	Pd2	58.256(19)
C34	C33	C32	119.8(14)	Ag2	Pd1	Pd3	58.79(2)
C33	C34	C35	118.6(15)	P2	Pd2	Pd1	149.12(6)
C33	C34	F6	118.8(16)	P2	Pd2	Pd3	149.37(6)
F6	C34	C35	122.1(15)	P2	Pd2	Ag2	133.38(6)
C36	C35	C34	122.0(16)	S2	Pd2	P2	100.40(7)
C35	C36	C31	120.2(16)	S2	Pd2	Pd1	50.11(5)
C38	C37	C42	118.2(8)	S2	Pd2	Pd3	109.74(5)
C38	C37	P3	122.3(6)	S2	Pd2	Ag2	81.24(5)
C42	C37	P3	119.5(7)	S3	Pd2	P2	99.90(7)
C37	C38	C39	121.4(8)	S3	Pd2	S2	159.18(7)
C40	C39	C38	117.1(9)	S3	Pd2	Pd1	110.43(5)
C39	C40	C41	123.4(9)	S3	Pd2	Pd3	50.74(5)
F7	C40	C39	117.1(9)	S3	Pd2	Ag2	81.48(5)
F7	C40	C41	119.5(9)	Pd3	Pd2	Pd1	59.800(18)
C42	C41	C40	117.7(8)	Ag2	Pd2	Pd1	59.74(2)
C41	C42	C37	122.2(9)	Ag2	Pd2	Pd3	59.27(2)
C44	C43	P3	121.0(7)	P3	Pd3	Pd1	149.50(6)
C47	C43	C44	116.9(9)	P3	Pd3	Pd2	149.18(5)
C47	C43	P3	122.0(8)	P3	Pd3	Ag2	131.78(6)
C43	C44	C45	121.2(10)	S1	Pd3	P3	99.45(7)
C51	C45	C44	118.4(12)	S1	Pd3	S3	160.54(7)
C51	C46	C47	118.9(12)	S1	Pd3	Pd1	50.75(5)
C46	C47	C43	122.1(11)	S1	Pd3	Pd2	111.09(6)
C49	C48	C53	118.8(9)	S1	Pd3	Ag2	83.45(5)
C49	C48	P3	119.3(7)	S3	Pd3	P3	99.60(7)
C53	C48	P3	121.9(9)	S3	Pd3	Pd1	110.65(5)
C48	C49	C50	122.2(10)	S3	Pd3	Pd2	50.36(5)
C52	C50	C49	117.7(13)	S3	Pd3	Ag2	80.94(5)
C45	C51	C46	122.3(12)	Pd1	Pd3	Pd2	60.403(18)
C45	C51	F8	119.6(12)	Ag2	Pd3	Pd1	59.79(2)
C46	C51	F8	118.0(11)	Ag2	Pd3	Pd2	58.78(2)
C50	C52	C54	123.6(12)	O1	Ag2	O2	94.8(3)
C50	C52	F9	120.2(14)	O1	Ag2	O3	94.9(3)
F9	C52	C54	115.9(13)	O1	Ag2	Pd1	143.23(19)
C48	C53	C54	119.9(14)	O1	Ag2	Pd2	82.98(19)
C52	C54	C53	117.6(13)	O1	Ag2	Pd3	113.10(19)
O2	C55	C56	108.6(11)	O2	Ag2	Pd1	121.33(18)
C55	C56	C57	105.1(11)	O2	Ag2	Pd2	152.4(2)
C56	C57	C58	103.6(11)	O2	Ag2	Pd3	94.46(19)
O2	C58	C57	105.2(10)	O3	Ag2	O2	91.1(3)
O3	C59	C60	106.4(10)	O3	Ag2	Pd1	91.4(2)
C59	C60	C61	104.1(10)	O3	Ag2	Pd2	116.5(2)
C60	C61	C62	104.4(10)	O3	Ag2	Pd3	150.9(2)
O3	C62	C61	106.2(9)	Pd2	Ag2	Pd1	62.00(2)
O5	C63	C66	103.7(16)	Pd2	Ag2	Pd3	61.95(2)
O5	C64	C67	104.6(14)	Pd3	Ag2	Pd1	61.42(2)
O4	C65	C70	105.8(13)	F11	Sb1	F10	179.6(4)

C63	C66	C67	112.8(18)	F11	Sb1	F12	90.6(4)
C66	C67	C64	100.6(17)	F12	Sb1	F10	89.3(3)
C69	C68	O4	109.5(13)	F13	Sb1	F10	90.4(4)
C68	C69	C70	105.2(15)	F13	Sb1	F11	90.0(4)
C65	C70	C69	108.1(15)	F13	Sb1	F12	89.6(4)
C72	C71	C76	117.5(8)	F13	Sb1	F14	91.0(4)
C72	C71	S1	124.6(7)	F14	Sb1	F10	88.6(3)
C76	C71	S1	117.7(6)	F14	Sb1	F11	91.6(4)
C73	C72	C71	122.5(8)	F14	Sb1	F12	177.8(4)
C74	C73	C72	118.6(9)	F15	Sb1	F10	87.7(4)
C73	C74	C75	120.7(9)	F15	Sb1	F11	91.9(4)
C73	C74	Cl1	119.8(8)	F15	Sb1	F12	89.3(4)
C75	C74	Cl1	119.5(8)	F15	Sb1	F13	177.8(4)
C76	C75	C74	120.4(9)	F15	Sb1	F14	90.0(4)
C75	C76	C71	120.3(8)	F16	Sb2	F17	90.3(4)
C78	C77	C82	118.1(8)	F16	Sb2	F19	89.9(4)
C78	C77	S2	124.5(7)	F16	Sb2	F20	84.6(5)
C82	C77	S2	117.4(7)	F17	Sb2	F20	89.4(5)
C77	C78	C79	121.3(9)	F18	Sb2	F16	178.0(7)
C80	C79	C78	119.3(10)	F18	Sb2	F17	90.7(4)
C79	C80	Cl2	119.8(9)	F18	Sb2	F19	89.1(4)
C81	C80	C79	121.4(10)	F18	Sb2	F20	93.7(7)
C81	C80	Cl2	118.8(9)	F18	Sb2	F21	92.3(7)
C80	C81	C82	119.1(10)	F19	Sb2	F17	178.2(5)
C77	C82	C81	120.7(9)	F19	Sb2	F20	88.8(5)
C84	C83	C88	121.0(8)	F21	Sb2	F16	89.5(6)
C84	C83	S3	123.2(6)	F21	Sb2	F17	92.4(6)
C88	C83	S3	115.6(7)	F21	Sb2	F19	89.4(5)
C83	C84	C85	118.6(8)	F21	Sb2	F20	173.8(6)
C86	C85	C84	120.3(9)				

2-BF$_4$ (CCDC 1410440)

Ortep of triangular cluster highlighting Pd_3-$Ag(BF_4)$-$(H_2O)_2$ core (down). Hydrogen atoms omitted for clarity. Thermal ellipsoids are shown at 50% probability.

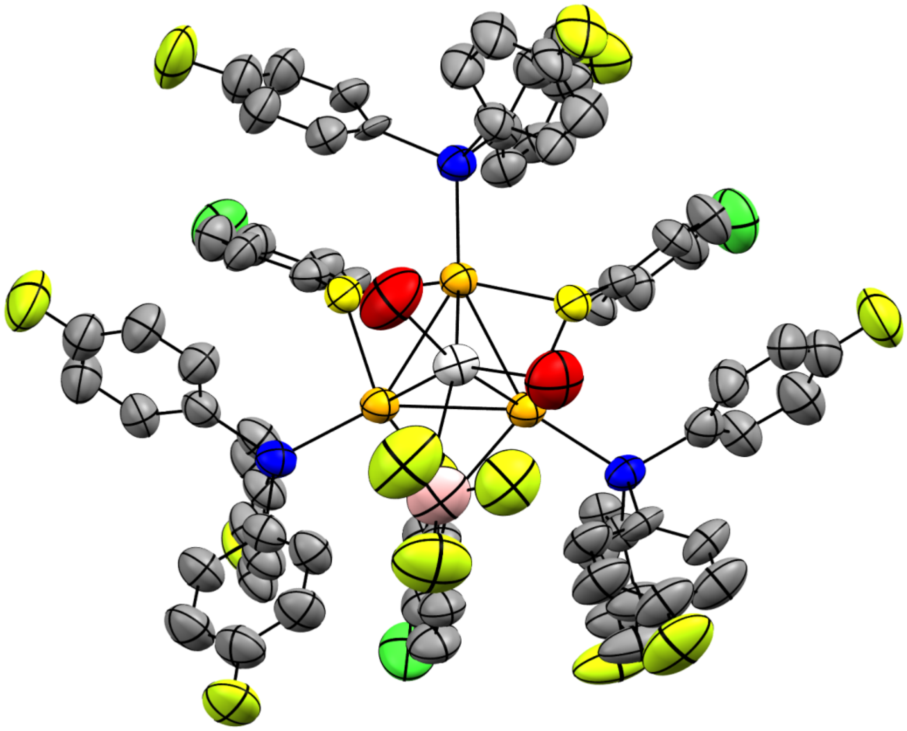

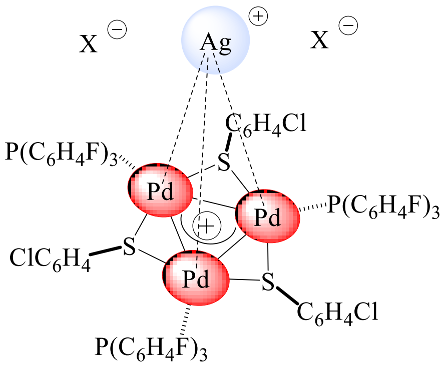

7: X = BF_4

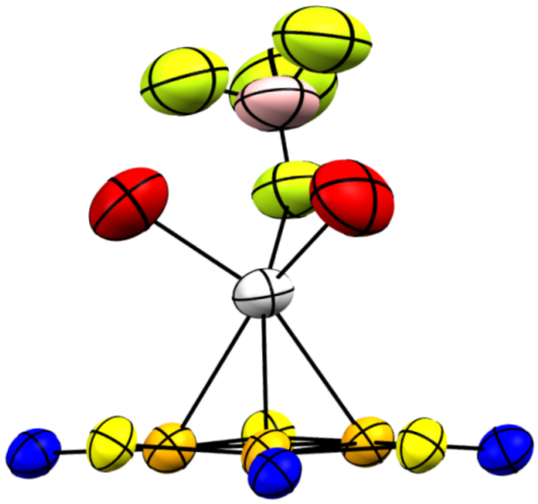

Pd_3 triangle is nearly equilateral, Pd-Pd-Pd angles range between 59.51(3)° and 60.72(3)°. Pd-Pd distances range between 2.8741(11) Å and 2.9093(12) Å. Ag-Pd_3 assembly exhibit a distorted trigonal pyramidal geometry, with Ag-Pd distances between 2.7928(12) Å and 2.8185(12) Å. Pd-Ag-Pd angles are between 61.66(3)° and 62.46(3)°. Ag-water molecule distances are 2.321(11) Å and 2.348(11) Å. Ag-F14 (closest fluorine atom of BF_4 to silver atom) distance is 2.583(10) Å.

Table 1 Fractional Atomic Coordinates and Equivalent Isotropic Displacement Parameters for 2-BF_4				
**Atom**	***x***	***y***	***z***	**U(eq)**
**C1**	0.7646(6)	0.4504(4)	0.4864(4)	0.068(3)
**C2**	0.8056(7)	0.4505(5)	0.4501(4)	0.084(4)
**C3**	0.8016(8)	0.4165(5)	0.4213(4)	0.100(5)
**C4**	0.7558(8)	0.3840(5)	0.4292(4)	0.093(4)
**C5**	0.7131(7)	0.3849(4)	0.4622(4)	0.087(4)
**C6**	0.7166(6)	0.4185(4)	0.4902(4)	0.075(3)
**C7**	0.7651(6)	0.5421(4)	0.4905(4)	0.065(3)
**C8**	0.6997(7)	0.5586(4)	0.4805(4)	0.076(3)
**C9**	0.6909(8)	0.5922(4)	0.4552(4)	0.089(4)
**C10**	0.7494(8)	0.6117(4)	0.4368(4)	0.086(4)
**C11**	0.8108(8)	0.5985(5)	0.4448(4)	0.092(4)
**C12**	0.8200(7)	0.5632(4)	0.4708(4)	0.074(3)
**C13**	0.8621(5)	0.4956(4)	0.5382(4)	0.069(3)
**C14**	0.8829(6)	0.5313(5)	0.5595(4)	0.078(3)
**C15**	0.9492(7)	0.5318(5)	0.5751(5)	0.092(4)
**C16**	0.9904(7)	0.4990(5)	0.5641(5)	0.088(4)
**C17**	0.9699(6)	0.4628(5)	0.5429(4)	0.079(3)
**C18**	0.9049(6)	0.4615(4)	0.5293(4)	0.076(3)
**C19**	0.5998(5)	0.6444(3)	0.6320(3)	0.053(2)
**C20**	0.5757(6)	0.6465(4)	0.5916(4)	0.071(3)
**C21**	0.5908(7)	0.6808(4)	0.5653(5)	0.087(4)
**C22**	0.6312(8)	0.7115(5)	0.5800(5)	0.092(4)
**C23**	0.6574(7)	0.7114(4)	0.6181(5)	0.086(4)
**C24**	0.6422(6)	0.6781(3)	0.6447(4)	0.066(3)
**C25**	0.4884(6)	0.5984(4)	0.6673(4)	0.066(3)
**C26**	0.4563(6)	0.5649(4)	0.6858(4)	0.078(4)
**C27**	0.3882(6)	0.5640(5)	0.6884(5)	0.097(5)
**C28**	0.3525(6)	0.5977(5)	0.6734(4)	0.077(3)
**C29**	0.3818(6)	0.6319(5)	0.6572(4)	0.093(4)
**C30**	0.4502(6)	0.6333(5)	0.6529(4)	0.090(4)
**C31**	0.6087(6)	0.6145(4)	0.7140(4)	0.066(3)
**C32**	0.6724(6)	0.6026(4)	0.7261(4)	0.073(3)
**C33**	0.6985(7)	0.6150(4)	0.7649(4)	0.081(3)
**C34**	0.6577(8)	0.6382(5)	0.7891(5)	0.092(4)
**C35**	0.5947(7)	0.6504(4)	0.7797(4)	0.090(4)
**C36**	0.5707(7)	0.6379(4)	0.7412(4)	0.076(3)
**C37**	0.6182(6)	0.3842(3)	0.7397(4)	0.066(3)
**C38**	0.6559(7)	0.3792(4)	0.7735(4)	0.080(3)
**C39**	0.6301(8)	0.3813(5)	0.8133(5)	0.107(5)
**C40**	0.5627(8)	0.3915(5)	0.8171(5)	0.094(4)
**C41**	0.5217(8)	0.3962(4)	0.7830(5)	0.095(4)
**C42**	0.5502(7)	0.3927(4)	0.7442(5)	0.086(4)
**C43**	0.6076(6)	0.3375(3)	0.6635(4)	0.065(3)
**C44**	0.5995(7)	0.3374(4)	0.6213(5)	0.086(4)
**C45**	0.5656(8)	0.3035(4)	0.6033(6)	0.110(5)
**C46**	0.5430(8)	0.2714(5)	0.6267(6)	0.108(4)
**C47**	0.5471(8)	0.2708(4)	0.6681(6)	0.109(5)
**C48**	0.5793(6)	0.3053(4)	0.6866(5)	0.081(3)
**C49**	0.7371(6)	0.3610(3)	0.6963(3)	0.062(3)
**C50**	0.7877(6)	0.3885(4)	0.7096(4)	0.064(3)
**C51**	0.8515(6)	0.3758(4)	0.7176(4)	0.067(3)
**C52**	0.8658(6)	0.3333(4)	0.7104(5)	0.091(4)
**C53**	0.8199(7)	0.3043(4)	0.6981(6)	0.124(7)
**C54**	0.7556(7)	0.3179(4)	0.6893(5)	0.096(5)
**C55**	0.7367(5)	0.5942(3)	0.5932(4)	0.058(2)
**C56**	0.7751(6)	0.5859(3)	0.6282(4)	0.066(3)
**C57**	0.8307(7)	0.6125(4)	0.6372(4)	0.080(3)
**C58**	0.8458(6)	0.6467(4)	0.6098(4)	0.078(3)
**C59**	0.8090(6)	0.6527(4)	0.5747(4)	0.080(3)
**C60**	0.7537(6)	0.6273(3)	0.5664(4)	0.068(3)
**C61**	0.8073(5)	0.4126(4)	0.6088(4)	0.063(3)
**C62**	0.8442(6)	0.4434(4)	0.6300(3)	0.064(3)
**C63**	0.9103(6)	0.4371(5)	0.6372(4)	0.074(3)
**C64**	0.9406(7)	0.4001(5)	0.6241(4)	0.083(3)
**C65**	0.9034(7)	0.3686(5)	0.6046(5)	0.095(4)
**C66**	0.8354(7)	0.3752(4)	0.5964(4)	0.080(4)
**C67**	0.6238(5)	0.4959(3)	0.7386(4)	0.056(2)
**C68**	0.6934(5)	0.4976(3)	0.7441(3)	0.057(3)
**C69**	0.7190(7)	0.5052(4)	0.7824(4)	0.072(3)
**C70**	0.6785(8)	0.5109(4)	0.8151(4)	0.080(3)
**C71**	0.6093(8)	0.5101(5)	0.8108(4)	0.094(4)
**C72**	0.5818(6)	0.5021(4)	0.7717(4)	0.067(3)

**Ag1**	0.56739(4)	0.47207(3)	0.58925(3)	0.0691(3)
**B1**	0.8789(10)	0.5251(7)	0.7194(6)	0.092(4)
**Cl1**	0.91422(19)	0.67837(12)	0.62037(15)	0.1130(13)
**Cl2**	1.02558(19)	0.39196(16)	0.63073(15)	0.1276(16)
**Cl3**	0.7103(3)	0.52227(18)	0.86262(13)	0.1303(16)
**F1**	0.7530(5)	0.3511(3)	0.4032(2)	0.121(3)
**F2**	0.7386(5)	0.6451(3)	0.4111(3)	0.138(3)
**F3**	1.0541(4)	0.4997(3)	0.5796(3)	0.114(3)
**F4**	0.6499(6)	0.7447(3)	0.5537(3)	0.144(4)
**F5**	0.2839(3)	0.5958(3)	0.6760(3)	0.109(3)
**F6**	0.6818(5)	0.6500(3)	0.8275(3)	0.130(3)
**F7**	0.5356(5)	0.3948(3)	0.8548(3)	0.136(3)
**F8**	0.5113(6)	0.2363(3)	0.6082(3)	0.159(4)
**F9**	0.9294(4)	0.3189(3)	0.7189(4)	0.146(4)
**F10**	0.8360(5)	0.5580(3)	0.7231(3)	0.134(3)
**F11**	0.8462(4)	0.4861(3)	0.7207(3)	0.119(3)
**F12**	0.9170(5)	0.5263(3)	0.7560(3)	0.128(3)
**F13**	0.9209(4)	0.5294(3)	0.6879(3)	0.113(3)
**P1**	0.77291(15)	0.49497(10)	0.52205(10)	0.0623(8)
**P2**	0.57993(14)	0.59912(9)	0.66401(10)	0.0588(8)
**P3**	0.65430(14)	0.38147(9)	0.68838(10)	0.0590(8)
**Pd1**	0.70454(4)	0.49091(3)	0.57923(3)	0.0573(3)
**Pd2**	0.62345(4)	0.53446(3)	0.64091(3)	0.0556(3)
**Pd3**	0.65691(4)	0.44456(3)	0.65029(3)	0.0573(3)
**S1**	0.66708(14)	0.56086(9)	0.58124(9)	0.0612(8)
**S2**	0.72006(15)	0.41974(9)	0.59638(9)	0.0619(8)
**S3**	0.58684(14)	0.48622(9)	0.68956(9)	0.0602(7)
**F14**	0.5660(4)	0.4111(3)	0.5350(3)	0.110(3)
**F15**	0.4764(5)	0.3701(4)	0.5465(4)	0.152(4)
**F16**	0.5466(6)	0.3510(4)	0.4979(4)	0.186(4)
**F17**	0.4788(6)	0.4077(4)	0.4914(4)	0.178(4)
**B2**	0.5212(13)	0.3868(8)	0.5162(8)	0.126(5)
**O1**	0.4851(6)	0.4242(4)	0.6136(4)	0.133(4)
**O2**	0.5016(7)	0.5160(4)	0.5478(4)	0.155(5)
**Cl6**	0.9709(8)	0.2650(4)	0.4946(7)	0.462(14)
**Cl5**	0.9132(7)	0.3471(5)	0.4670(5)	0.370(11)
**Cl4**	1.0465(6)	0.3456(6)	0.5046(5)	0.384(10)
**C73**	0.9880(6)	0.3169(4)	0.4738(3)	0.293(14)

Table 2 Anisotropic Displacement Parameters for 2-BF$_4$

Atom	U_{11}	U_{22}	U_{33}	U_{23}	U_{13}	U_{12}
**C1**	0.063(6)	0.077(5)	0.064(6)	-0.011(5)	-0.004(5)	0.005(4)
**C2**	0.082(8)	0.097(7)	0.074(7)	-0.017(6)	0.009(6)	-0.001(6)
**C3**	0.115(10)	0.108(8)	0.079(8)	-0.030(6)	0.016(7)	0.000(7)
**C4**	0.110(9)	0.096(7)	0.074(7)	-0.025(6)	-0.002(6)	0.006(6)
**C5**	0.106(9)	0.077(7)	0.079(7)	-0.023(6)	-0.009(6)	-0.008(6)
**C6**	0.076(7)	0.086(6)	0.063(6)	-0.014(5)	-0.001(5)	-0.010(5)
**C7**	0.069(5)	0.069(5)	0.057(6)	-0.010(4)	0.010(5)	0.003(4)
**C8**	0.077(6)	0.068(6)	0.082(8)	0.010(6)	-0.001(5)	0.001(5)
**C9**	0.093(7)	0.083(7)	0.092(9)	0.021(7)	-0.012(6)	-0.001(5)
**C10**	0.098(7)	0.077(7)	0.083(8)	0.013(6)	0.000(6)	-0.007(5)
**C11**	0.090(7)	0.098(8)	0.088(9)	0.025(7)	0.005(6)	-0.008(6)
**C12**	0.074(6)	0.086(7)	0.061(7)	-0.001(5)	0.007(5)	-0.007(5)
**C13**	0.043(5)	0.094(6)	0.071(7)	-0.009(5)	0.005(4)	0.002(4)
**C14**	0.051(5)	0.104(7)	0.078(8)	-0.018(6)	0.010(5)	-0.006(5)
**C15**	0.054(5)	0.124(8)	0.098(10)	-0.017(7)	0.003(6)	-0.007(5)
**C16**	0.049(5)	0.125(8)	0.089(9)	0.002(6)	0.012(5)	-0.004(5)
**C17**	0.051(5)	0.118(8)	0.070(7)	0.007(6)	0.009(5)	0.008(5)
**C18**	0.057(5)	0.100(7)	0.073(8)	-0.003(6)	0.005(5)	0.011(5)
**C19**	0.066(6)	0.017(3)	0.076(5)	0.003(4)	0.006(4)	0.008(3)
**C20**	0.074(7)	0.065(6)	0.074(6)	0.005(5)	0.002(5)	0.006(5)
**C21**	0.106(9)	0.068(6)	0.087(7)	0.021(5)	-0.003(6)	-0.003(6)
**C22**	0.108(10)	0.079(6)	0.088(7)	0.018(5)	0.006(6)	-0.009(6)
**C23**	0.103(9)	0.064(6)	0.091(7)	0.011(5)	0.012(6)	-0.016(6)
**C24**	0.080(7)	0.037(4)	0.082(7)	-0.003(4)	0.009(5)	-0.003(4)
**C25**	0.055(5)	0.073(6)	0.069(7)	0.003(5)	0.008(4)	0.005(4)
**C26**	0.045(5)	0.078(6)	0.111(10)	0.026(6)	0.013(5)	0.006(4)
**C27**	0.055(5)	0.101(8)	0.133(12)	0.021(8)	0.008(6)	0.002(5)
**C28**	0.046(5)	0.106(7)	0.079(8)	0.006(6)	0.018(5)	0.012(4)
**C29**	0.054(5)	0.111(8)	0.112(11)	0.018(7)	0.016(6)	0.013(5)
**C30**	0.054(5)	0.095(7)	0.120(11)	0.026(7)	0.017(5)	0.014(5)
**C31**	0.069(6)	0.063(6)	0.066(5)	0.002(4)	0.001(4)	0.001(5)

**C32**	0.074(6)	0.064(7)	0.080(6)	-0.006(5)	-0.008(5)	-0.002(5)
**C33**	0.093(7)	0.058(6)	0.093(7)	-0.006(5)	-0.018(5)	0.001(5)
**C34**	0.106(8)	0.084(8)	0.087(7)	-0.014(6)	-0.018(6)	0.008(6)
**C35**	0.105(7)	0.088(8)	0.078(7)	-0.009(6)	-0.005(5)	0.011(6)
**C36**	0.079(7)	0.081(7)	0.069(6)	-0.003(5)	0.002(5)	0.008(6)
**C37**	0.067(5)	0.051(6)	0.081(5)	0.002(5)	0.004(4)	-0.012(5)
**C38**	0.075(7)	0.095(9)	0.070(6)	-0.011(6)	0.007(5)	-0.004(6)
**C39**	0.109(8)	0.130(12)	0.082(7)	-0.019(7)	0.015(6)	-0.009(7)
**C40**	0.107(8)	0.085(9)	0.090(7)	0.003(6)	0.030(5)	-0.010(7)
**C41**	0.102(8)	0.080(8)	0.105(7)	0.012(6)	0.032(5)	-0.003(7)
**C42**	0.069(6)	0.094(9)	0.095(7)	0.023(6)	0.016(5)	0.003(6)
**C43**	0.067(6)	0.030(4)	0.098(6)	0.009(4)	-0.009(5)	0.005(4)
**C44**	0.115(10)	0.047(5)	0.098(7)	-0.005(5)	-0.017(6)	-0.008(6)
**C45**	0.134(11)	0.057(6)	0.139(9)	-0.007(6)	-0.036(7)	-0.007(7)
**C46**	0.111(10)	0.062(6)	0.152(9)	0.004(6)	-0.043(8)	-0.011(6)
**C47**	0.111(11)	0.061(6)	0.155(9)	0.013(6)	-0.041(8)	-0.022(7)
**C48**	0.075(8)	0.053(5)	0.116(8)	0.024(5)	-0.022(6)	-0.008(5)
**C49**	0.064(5)	0.054(5)	0.067(7)	0.002(5)	-0.006(4)	0.002(4)
**C50**	0.062(5)	0.057(5)	0.073(8)	-0.004(5)	-0.006(5)	0.004(4)
**C51**	0.059(5)	0.069(5)	0.074(8)	0.001(5)	-0.010(5)	0.002(4)
**C52**	0.062(6)	0.073(5)	0.138(12)	-0.016(6)	-0.024(6)	0.007(4)
**C53**	0.073(6)	0.063(6)	0.235(19)	-0.033(8)	-0.050(8)	0.014(5)
**C54**	0.068(6)	0.067(5)	0.153(13)	-0.020(6)	-0.027(7)	0.009(5)
**C55**	0.053(5)	0.049(5)	0.070(6)	-0.003(4)	0.003(4)	0.004(4)
**C56**	0.072(6)	0.039(5)	0.086(7)	0.005(5)	-0.009(5)	0.007(4)
**C57**	0.084(7)	0.064(6)	0.091(8)	0.000(5)	-0.007(6)	-0.005(5)
**C58**	0.072(6)	0.067(6)	0.093(7)	-0.002(5)	0.006(5)	-0.006(5)
**C59**	0.076(7)	0.070(7)	0.093(7)	0.003(6)	0.007(5)	-0.016(5)
**C60**	0.075(6)	0.046(5)	0.084(7)	0.013(5)	0.001(5)	-0.001(4)
**C61**	0.052(5)	0.068(5)	0.070(7)	0.010(5)	-0.002(4)	0.014(4)
**C62**	0.063(5)	0.076(6)	0.053(6)	0.011(5)	0.004(5)	0.007(4)
**C63**	0.057(5)	0.099(7)	0.067(7)	0.008(6)	-0.004(5)	0.005(5)
**C64**	0.073(6)	0.101(7)	0.075(8)	0.012(6)	0.001(5)	0.012(5)
**C65**	0.071(6)	0.091(7)	0.124(11)	0.002(7)	-0.006(6)	0.023(5)
**C66**	0.075(6)	0.070(6)	0.096(9)	-0.002(6)	-0.003(6)	0.015(5)
**C67**	0.062(5)	0.043(5)	0.064(5)	0.001(4)	0.004(4)	0.006(4)
**C68**	0.053(5)	0.069(6)	0.050(5)	-0.003(5)	0.000(4)	0.009(4)
**C69**	0.075(6)	0.085(8)	0.055(5)	0.003(5)	-0.009(4)	0.001(6)
**C70**	0.097(7)	0.088(9)	0.055(5)	0.001(5)	0.005(5)	-0.004(6)
**C71**	0.100(7)	0.122(11)	0.059(6)	-0.016(6)	0.010(5)	-0.001(7)
**C72**	0.068(6)	0.070(7)	0.063(5)	-0.004(5)	0.017(4)	0.009(5)
**Ag1**	0.0571(6)	0.0673(6)	0.0830(7)	-0.0040(5)	-0.0115(5)	-0.0025(4)
**B1**	0.081(8)	0.103(7)	0.091(8)	-0.013(6)	0.003(5)	-0.013(5)
**Cl1**	0.089(3)	0.093(2)	0.157(4)	0.002(3)	-0.018(3)	-0.029(2)
**Cl2**	0.070(2)	0.160(4)	0.154(4)	0.031(3)	-0.005(2)	0.025(2)
**Cl3**	0.150(4)	0.165(4)	0.076(3)	-0.007(3)	-0.016(3)	-0.021(3)
**F1**	0.168(8)	0.104(5)	0.091(5)	-0.040(5)	-0.003(5)	0.005(5)
**F2**	0.154(8)	0.120(6)	0.139(7)	0.062(6)	0.002(6)	0.004(6)
**F3**	0.049(4)	0.175(8)	0.117(7)	-0.001(6)	-0.001(4)	-0.007(4)
**F4**	0.231(11)	0.088(5)	0.111(6)	0.028(5)	0.012(6)	-0.050(6)
**F5**	0.053(4)	0.142(7)	0.132(7)	0.013(6)	0.014(4)	0.011(4)
**F6**	0.153(8)	0.141(8)	0.095(5)	-0.034(5)	-0.035(5)	0.012(6)
**F7**	0.148(8)	0.161(9)	0.098(6)	-0.009(5)	0.053(5)	-0.012(7)
**F8**	0.197(10)	0.082(5)	0.198(9)	-0.010(6)	-0.074(8)	-0.043(6)
**F9**	0.070(5)	0.089(5)	0.280(13)	-0.030(7)	-0.058(6)	0.018(4)
**F10**	0.139(7)	0.137(7)	0.126(7)	0.010(6)	0.031(6)	0.026(5)
**F11**	0.112(6)	0.122(6)	0.124(7)	-0.023(5)	0.020(5)	-0.029(5)
**F12**	0.129(7)	0.135(7)	0.119(6)	-0.006(5)	-0.015(5)	-0.023(5)
**F13**	0.104(6)	0.119(6)	0.116(6)	-0.011(5)	0.017(5)	-0.019(5)
**P1**	0.0517(17)	0.0630(16)	0.072(2)	-0.0051(15)	0.0021(15)	0.0006(13)
**P2**	0.0499(17)	0.0572(16)	0.0691(19)	-0.0001(14)	0.0023(14)	0.0031(13)
**P3**	0.0508(17)	0.0491(14)	0.077(2)	0.0028(14)	-0.0018(15)	-0.0009(12)
**Pd1**	0.0518(5)	0.0544(5)	0.0658(6)	-0.0026(4)	0.0019(4)	0.0016(4)
**Pd2**	0.0482(5)	0.0524(5)	0.0662(6)	-0.0003(4)	0.0034(4)	0.0007(4)
**Pd3**	0.0514(5)	0.0527(5)	0.0678(6)	-0.0007(4)	0.0012(4)	0.0021(4)
**S1**	0.0561(17)	0.0583(15)	0.0690(19)	0.0041(14)	0.0050(14)	0.0010(13)
**S2**	0.0617(18)	0.0535(15)	0.0706(19)	0.0028(14)	-0.0035(14)	0.0072(13)
**S3**	0.0522(16)	0.0576(15)	0.0709(19)	0.0022(14)	0.0100(14)	0.0006(13)
**F14**	0.090(5)	0.122(6)	0.116(6)	-0.039(5)	-0.005(4)	-0.018(4)
**F15**	0.132(7)	0.147(8)	0.178(8)	-0.005(6)	-0.031(6)	-0.031(6)
**F16**	0.193(10)	0.145(8)	0.222(11)	-0.063(8)	-0.015(8)	-0.016(7)
**F17**	0.191(9)	0.150(8)	0.193(9)	0.005(7)	-0.090(8)	-0.031(7)
**B2**	0.126(9)	0.113(9)	0.140(10)	-0.024(7)	-0.043(7)	-0.033(7)
**O1**	0.119(8)	0.108(7)	0.171(10)	-0.002(7)	0.005(7)	-0.031(6)
**O2**	0.172(10)	0.129(9)	0.163(11)	0.034(8)	-0.055(9)	0.012(8)

**Cl6**	0.39(2)	0.387(19)	0.61(3)	0.075(19)	0.07(2)	0.067(15)
**Cl5**	0.329(17)	0.306(16)	0.48(2)	-0.110(16)	-0.037(16)	0.086(13)
**Cl4**	0.320(16)	0.47(2)	0.358(19)	-0.157(17)	0.046(13)	0.052(14)
**C73**	0.30(2)	0.32(2)	0.26(3)	-0.053(19)	0.053(18)	0.077(15)

Table 3 Bond Lengths for 2-BF_4

Atom	Atom	Length/Å	Atom	Atom	Length/Å
C1	C2	1.437(17)	C46	F8	1.393(16)
C1	C6	1.380(16)	C47	C48	1.382(17)
C1	P1	1.809(12)	C49	C50	1.390(15)
C2	C3	1.409(17)	C49	C54	1.401(15)
C3	C4	1.38(2)	C49	P3	1.789(11)
C4	C5	1.372(19)	C50	C51	1.359(14)
C4	F1	1.324(15)	C51	C52	1.363(16)
C5	C6	1.384(16)	C52	C53	1.345(17)
C7	C8	1.440(16)	C52	F9	1.373(13)
C7	C12	1.427(16)	C53	C54	1.381(17)
C7	P1	1.789(12)	C55	C56	1.397(15)
C8	C9	1.339(16)	C55	C60	1.386(14)
C9	C10	1.445(19)	C55	S1	1.774(11)
C10	C11	1.317(18)	C56	C57	1.413(16)
C10	F2	1.346(15)	C57	C58	1.415(17)
C11	C12	1.393(17)	C58	C59	1.371(17)
C13	C14	1.367(17)	C58	Cl1	1.716(13)
C13	C18	1.387(16)	C59	C60	1.381(16)
C13	P1	1.857(12)	C61	C62	1.389(16)
C14	C15	1.419(17)	C61	C66	1.346(16)
C15	C16	1.352(19)	C61	S2	1.802(11)
C16	C17	1.376(18)	C62	C63	1.354(15)
C16	F3	1.369(15)	C63	C64	1.365(18)
C17	C18	1.373(17)	C64	C65	1.379(19)
C19	C20	1.402(15)	C64	Cl2	1.729(14)
C19	C24	1.404(14)	C65	C66	1.399(18)
C19	P2	1.790(10)	C67	C68	1.402(14)
C20	C21	1.395(16)	C67	C72	1.380(15)
C21	C22	1.336(18)	C67	S3	1.783(12)
C22	C23	1.345(18)	C68	C69	1.368(15)
C22	F4	1.388(15)	C69	C70	1.348(17)
C23	C24	1.379(16)	C70	C71	1.39(2)
C25	C26	1.359(15)	C70	Cl3	1.708(14)
C25	C30	1.402(16)	C71	C72	1.408(17)
C25	P2	1.831(12)	Ag1	Pd1	2.8185(12)
C26	C27	1.364(16)	Ag1	Pd2	2.7928(12)
C27	C28	1.356(18)	Ag1	Pd3	2.8045(12)
C28	C29	1.317(18)	Ag1	O1	2.348(11)
C28	F5	1.372(13)	Ag1	O2	2.321(11)
C29	C30	1.373(17)	B1	F10	1.34(2)
C31	C32	1.383(16)	B1	F11	1.37(2)
C31	C36	1.371(15)	B1	F12	1.41(2)
C31	P2	1.790(12)	B1	F13	1.33(2)
C32	C33	1.416(16)	P1	Pd1	2.311(3)
C33	C34	1.339(18)	P2	Pd2	2.305(3)
C34	C35	1.349(18)	P3	Pd3	2.311(3)
C34	F6	1.390(15)	Pd1	Pd2	2.9093(12)
C35	C36	1.396(16)	Pd1	Pd3	2.8818(12)
C37	C38	1.343(16)	Pd1	S1	2.289(3)
C37	C42	1.391(16)	Pd1	S2	2.291(3)
C37	P3	1.820(13)	Pd2	Pd3	2.8741(11)
C38	C39	1.396(18)	Pd2	S1	2.280(3)
C39	C40	1.39(2)	Pd2	S3	2.294(3)
C40	C41	1.39(2)	Pd3	S2	2.293(3)
C40	F7	1.343(15)	Pd3	S3	2.291(3)
C41	C42	1.390(18)	F14	B2	1.32(2)
C43	C44	1.382(17)	F15	B2	1.43(3)
C43	C48	1.366(15)	F16	B2	1.35(3)
C43	P3	1.837(12)	F17	B2	1.34(3)
C44	C45	1.379(17)	Cl6	C73	1.7758
C45	C46	1.33(2)	Cl5	C73	1.7756
C46	C47	1.35(2)	Cl4	C73	1.7767

Table 4 Bond Angles for 2-BF_4

Atom	Atom	Atom	Angle/˚	Atom	Atom	Atom	Angle/˚
C2	C1	P1	118.2(10)	C61	C66	C65	118.2(13)
C6	C1	C2	118.1(12)	C68	C67	S3	122.1(9)
C6	C1	P1	123.4(10)	C72	C67	C68	119.8(11)
C3	C2	C1	120.8(14)	C72	C67	S3	118.1(9)
C4	C3	C2	117.1(14)	C69	C68	C67	119.6(11)
C5	C4	C3	122.8(14)	C70	C69	C68	121.2(13)
F1	C4	C3	117.9(14)	C69	C70	C71	120.9(13)
F1	C4	C5	119.2(15)	C69	C70	Cl3	121.2(12)
C4	C5	C6	119.9(14)	C71	C70	Cl3	117.7(11)
C1	C6	C5	120.8(13)	C70	C71	C72	118.9(13)
C8	C7	P1	119.8(9)	C67	C72	C71	119.6(12)
C12	C7	C8	115.7(11)	Pd2	Ag1	Pd1	62.46(3)
C12	C7	P1	124.3(10)	Pd2	Ag1	Pd3	61.79(3)
C9	C8	C7	122.2(13)	Pd3	Ag1	Pd1	61.66(3)
C8	C9	C10	118.2(14)	O1	Ag1	Pd1	148.1(3)
C11	C10	C9	122.8(14)	O1	Ag1	Pd2	120.8(3)
C11	C10	F2	120.7(14)	O1	Ag1	Pd3	90.9(3)
F2	C10	C9	116.5(13)	O2	Ag1	Pd1	111.2(4)
C10	C11	C12	119.1(14)	O2	Ag1	Pd2	99.9(3)
C11	C12	C7	122.0(13)	O2	Ag1	Pd3	161.7(3)
C14	C13	C18	122.1(12)	O2	Ag1	O1	99.7(5)
C14	C13	P1	116.3(9)	F10	B1	F11	111.3(15)
C18	C13	P1	121.6(10)	F10	B1	F12	104.4(15)
C13	C14	C15	118.2(13)	F11	B1	F12	104.6(16)
C16	C15	C14	117.7(14)	F13	B1	F10	113.3(17)
C15	C16	C17	124.1(14)	F13	B1	F11	114.5(16)
C15	C16	F3	117.1(14)	F13	B1	F12	107.7(14)
F3	C16	C17	118.3(13)	C1	P1	C13	106.1(6)
C18	C17	C16	117.8(13)	C1	P1	Pd1	114.9(4)
C17	C18	C13	119.6(13)	C7	P1	C1	104.2(6)
C20	C19	C24	116.7(10)	C7	P1	C13	103.7(6)
C20	C19	P2	120.4(8)	C7	P1	Pd1	117.0(4)
C24	C19	P2	122.8(9)	C13	P1	Pd1	109.8(4)
C21	C20	C19	122.4(12)	C19	P2	C25	105.4(5)
C22	C21	C20	116.8(14)	C19	P2	C31	104.5(5)
C21	C22	C23	124.3(14)	C19	P2	Pd2	113.9(3)
C21	C22	F4	117.7(14)	C25	P2	Pd2	112.7(4)
C23	C22	F4	117.8(14)	C31	P2	C25	105.7(6)
C22	C23	C24	119.6(14)	C31	P2	Pd2	113.9(4)
C23	C24	C19	120.1(12)	C37	P3	C43	103.7(6)
C26	C25	C30	118.6(11)	C37	P3	Pd3	117.5(4)
C26	C25	P2	120.4(9)	C43	P3	Pd3	113.6(4)
C30	C25	P2	121.0(9)	C49	P3	C37	104.6(6)
C25	C26	C27	120.9(12)	C49	P3	C43	105.7(5)
C28	C27	C26	119.1(14)	C49	P3	Pd3	110.8(4)
C27	C28	F5	117.9(12)	Ag1	Pd1	Pd2	58.34(3)
C29	C28	C27	121.8(13)	Ag1	Pd1	Pd3	58.93(3)
C29	C28	F5	120.2(12)	P1	Pd1	Ag1	132.70(9)
C28	C29	C30	120.6(14)	P1	Pd1	Pd2	149.21(8)
C29	C30	C25	118.9(13)	P1	Pd1	Pd3	150.23(8)
C32	C31	P2	119.0(9)	Pd3	Pd1	Pd2	59.51(3)
C36	C31	C32	117.7(12)	S1	Pd1	Ag1	82.78(8)
C36	C31	P2	123.3(10)	S1	Pd1	P1	99.47(11)
C31	C32	C33	121.3(12)	S1	Pd1	Pd2	50.31(8)
C34	C33	C32	116.4(13)	S1	Pd1	Pd3	109.81(8)
C33	C34	C35	125.7(15)	S1	Pd1	S2	160.82(11)
C33	C34	F6	117.4(14)	S2	Pd1	Ag1	84.54(8)
C35	C34	F6	116.9(14)	S2	Pd1	P1	99.70(11)
C34	C35	C36	116.5(14)	S2	Pd1	Pd2	110.58(8)
C31	C36	C35	122.4(13)	S2	Pd1	Pd3	51.08(8)
C38	C37	C42	118.9(13)	Ag1	Pd2	Pd1	59.20(3)
C38	C37	P3	121.5(10)	Ag1	Pd2	Pd3	59.30(3)
C42	C37	P3	119.6(10)	P2	Pd2	Ag1	130.08(8)
C37	C38	C39	123.1(14)	P2	Pd2	Pd1	146.71(9)
C40	C39	C38	117.0(16)	P2	Pd2	Pd3	153.02(9)
C41	C40	C39	121.6(15)	Pd3	Pd2	Pd1	59.77(3)
F7	C40	C39	119.4(16)	S1	Pd2	Ag1	83.53(8)
F7	C40	C41	119.0(15)	S1	Pd2	P2	96.40(11)
C40	C41	C42	118.5(15)	S1	Pd2	Pd1	50.58(7)
C41	C42	C37	120.8(14)	S1	Pd2	Pd3	110.34(8)
C44	C43	P3	119.8(9)	S1	Pd2	S3	160.43(11)

C48	C43	C44	119.7(12)	S3	Pd2	Ag1	80.74(8)
C48	C43	P3	120.5(11)	S3	Pd2	P2	102.61(11)
C45	C44	C43	118.8(14)	S3	Pd2	Pd1	110.66(8)
C46	C45	C44	119.4(17)	S3	Pd2	Pd3	51.13(7)
C45	C46	C47	124.0(16)	Ag1	Pd3	Pd1	59.41(3)
C45	C46	F8	119.3(17)	Ag1	Pd3	Pd2	58.90(3)
C47	C46	F8	116.7(15)	P3	Pd3	Ag1	128.41(8)
C46	C47	C48	116.8(15)	P3	Pd3	Pd1	149.01(9)
C43	C48	C47	121.0(15)	P3	Pd3	Pd2	150.16(9)
C50	C49	C54	116.2(11)	Pd2	Pd3	Pd1	60.72(3)
C50	C49	P3	120.0(8)	S2	Pd3	Ag1	84.83(8)
C54	C49	P3	123.8(9)	S2	Pd3	P3	98.07(11)
C51	C50	C49	124.3(11)	S2	Pd3	Pd1	51.01(7)
C50	C51	C52	116.3(11)	S2	Pd3	Pd2	111.72(8)
C51	C52	F9	118.1(11)	S3	Pd3	Ag1	80.54(8)
C53	C52	C51	123.4(13)	S3	Pd3	P3	99.28(11)
C53	C52	F9	118.3(12)	S3	Pd3	Pd1	111.70(8)
C52	C53	C54	119.5(13)	S3	Pd3	Pd2	51.23(7)
C53	C54	C49	120.1(12)	S3	Pd3	S2	161.95(11)
C56	C55	S1	120.1(9)	C55	S1	Pd1	107.4(4)
C60	C55	C56	121.0(11)	C55	S1	Pd2	108.7(4)
C60	C55	S1	118.9(9)	Pd2	S1	Pd1	79.11(9)
C55	C56	C57	119.5(11)	C61	S2	Pd1	107.7(4)
C56	C57	C58	118.1(12)	C61	S2	Pd3	113.6(4)
C57	C58	Cl1	118.0(11)	Pd1	S2	Pd3	77.92(9)
C59	C58	C57	120.8(12)	C67	S3	Pd2	112.1(4)
C59	C58	Cl1	121.1(10)	C67	S3	Pd3	109.9(4)
C58	C59	C60	121.0(13)	Pd3	S3	Pd2	77.63(9)
C59	C60	C55	119.4(12)	F14	B2	F15	108.1(19)
C62	C61	S2	122.7(9)	F14	B2	F16	115(2)
C66	C61	C62	121.2(11)	F14	B2	F17	116(2)
C66	C61	S2	116.2(10)	F16	B2	F15	104.0(18)
C63	C62	C61	120.2(12)	F17	B2	F15	101(2)
C62	C63	C64	120.0(13)	F17	B2	F16	112(2)
C63	C64	C65	119.8(13)	Cl6	C73	Cl4	111.2
C63	C64	Cl2	121.2(12)	Cl5	C73	Cl6	111.2
C65	C64	Cl2	119.0(11)	Cl5	C73	Cl4	111.2
C64	C65	C66	120.5(13)				

Table 5 Atomic Occupancy for 2-BF_4

**Atom**	***Occupancy***	**Atom**	***Occupancy***	**Atom**	***Occupancy***
C73	0.761(13)	H73	0.761(13)		
Cl4	0.761(13)	Cl5	0.761(13)	Cl6	0.761(13)

Table 6 Solvent masks information for 2-BF_4

**Number**	X	Y	Z	Volume (Å^3)	Electron count
**1**	0.378	0.250	0.263	1339	459
**2**	0.875	-0.250	0.237	1339	460
**3**	0.120	0.250	0.763	1339	458
**4**	0.620	-0.250	0.737	1339	460

7. XYZ Coordinates

# - Pd_3 cation 1 (M06/Def2-svp)

Optimized structure

141
scf done: -7647.902109

Pd	15.463718	0.250685	31.104253
P	13.135314	0.593521	30.996507
F	10.295581	-4.412294	29.589765
F	11.861105	4.806720	27.028752
F	10.795747	2.372230	36.131741
C	12.694513	2.037194	34.811364
H	13.318306	2.418930	35.622997
C	12.156628	-0.885208	30.541784
C	11.318636	1.968899	34.987916
C	12.413644	1.118262	32.585609
C	11.027029	1.055411	32.794729
H	10.370654	0.669594	32.006770
C	13.238673	1.607932	33.604523
H	14.323180	1.648348	33.456251
C	11.894301	2.993060	30.139932
H	11.514454	3.100819	31.160486
C	12.059848	-1.917701	31.487707
H	12.500474	-1.793636	32.483638
C	13.158114	1.783162	28.474455
H	13.790637	0.935073	28.186607
C	10.952282	-2.261081	28.949156
H	10.513953	-2.427018	27.961715
C	12.670437	1.882745	29.787669
C	10.877305	-3.265509	29.903365
C	11.408841	-3.103923	31.179201
H	11.313818	-3.915783	31.904754
C	12.095125	3.849921	27.911614
C	11.590087	-1.067013	29.276060
H	11.638293	-0.272661	28.525566
C	10.473467	1.482680	33.994013
H	9.397371	1.445864	34.179906
C	12.859174	2.753124	27.527369
H	13.234492	2.701468	26.501269
C	11.604120	3.981714	29.202956
H	11.007560	4.860473	29.460523
S	15.481882	-2.093909	31.197072
Cl	13.598434	-4.161782	25.551577
C	14.110670	-3.570504	27.097133
C	15.199670	-1.880938	28.417437
H	15.713571	-0.915707	28.499177
C	14.996837	-2.648431	29.572717
C	14.348221	-3.883338	29.469069
H	14.199015	-4.493986	30.365843
C	13.905722	-4.348041	28.235187
H	13.409117	-5.318666	28.144035
C	14.754193	-2.334523	27.182334
H	14.907765	-1.743345	26.275438
Pd	17.769307	-1.564271	31.144316
P	18.657333	-3.744364	31.082016
F	24.470087	-3.719704	29.932209
F	15.857873	-6.872742	26.903407

F	18.042713	-6.746489	36.145479
C	17.397858	-4.951292	34.795535
H	16.680578	-4.643122	35.559676
C	20.445892	-3.845333	30.706426
C	18.184070	-6.074602	35.017189
C	18.488987	-4.659587	32.650134
C	19.271005	-5.797599	32.902335
H	20.007407	-6.133586	32.163918
C	17.556150	-4.244351	33.607779
H	16.947686	-3.352766	33.424526
C	17.255485	-6.008829	30.108305
H	17.312387	-6.420026	31.121003
C	21.343602	-3.374761	31.677090
H	20.970522	-3.019701	32.644839
C	17.728389	-4.258881	28.510488
H	18.154747	-3.276759	28.270656
C	22.310278	-4.253526	29.210838
H	22.718086	-4.589159	28.253847
C	17.831978	-4.767845	29.815061
C	23.172829	-3.779184	30.188130
C	22.708794	-3.351003	31.428763
H	23.425248	-2.996670	32.174233
C	16.517574	-6.208441	27.837540
C	20.943941	-4.289835	29.477222
H	20.265226	-4.674824	28.710134
C	19.120643	-6.510591	34.084260
H	19.718285	-7.398614	34.304114
C	17.087929	-4.981444	27.513344
H	16.988357	-4.603552	26.491925
C	16.596064	-6.734587	29.119357
H	16.133487	-7.702647	29.328117
S	19.787381	-0.372959	31.206156
Cl	22.444101	-1.160066	25.547965
C	21.696317	-0.967542	27.098836
C	19.704763	-0.790485	28.435825
H	18.611794	-0.800257	28.528848
C	20.488618	-0.577434	29.578848
C	21.882174	-0.568334	29.461867
H	22.498516	-0.412408	30.353487
C	22.489271	-0.758449	28.225113
H	23.578343	-0.742177	28.124642
C	20.303752	-0.991844	27.198688
H	19.702575	-1.159942	26.301168
Pd	18.188549	1.344150	31.127616
P	19.649375	3.184788	31.020179
F	16.735643	8.210244	29.857605
F	23.639821	2.194671	26.755664
F	22.682421	4.174933	36.003712
C	21.380485	2.728157	34.709715
H	21.468756	1.964828	35.486329
C	18.850906	4.786662	30.639722
C	21.998535	3.958220	34.894696
C	20.562554	3.507173	32.564502
C	21.192511	4.742746	32.781092
H	21.116899	5.536277	32.029168
C	20.661483	2.507601	33.539130
H	20.167049	1.543004	33.382575
C	22.286051	3.093081	29.969933
H	22.644117	3.390547	30.960426
C	18.028350	5.356118	31.623729

H	17.927993	4.874040	32.603186
C	20.489414	2.553999	28.447168
H	19.419489	2.418482	28.245334
C	18.240575	6.577231	29.123186
H	18.298129	7.074142	28.151363
C	20.915693	2.948227	29.725380
C	17.434725	7.116230	30.115468
C	17.328457	6.528032	31.372368
H	16.689236	6.992588	32.127328
C	22.761025	2.464872	27.706474
C	18.953611	5.411594	29.392662
H	19.597050	4.992399	28.613309
C	21.914868	4.972228	33.944638
H	22.414850	5.924823	34.136192
C	21.405317	2.327125	27.428888
H	21.098070	2.014297	26.427017
C	23.214341	2.850466	28.960475
H	24.289164	2.948251	29.132057
S	16.147312	2.493657	31.204285
Cl	15.346771	5.183871	25.565788
C	15.614445	4.449233	27.112178
C	16.484416	2.630616	28.423898
H	17.029315	1.681054	28.499029
C	15.951856	3.211493	29.583559
C	15.262284	4.424167	29.488602
H	14.851203	4.882834	30.393684
C	15.087299	5.044095	28.256116
H	14.530141	5.982012	28.171234
C	16.324472	3.249634	27.190241
H	16.732588	2.804860	26.278319

Energies

Zero-point correction= 1.002487 (Hartree/Particle)
Thermal correction to Energy= 1.089545
Thermal correction to Enthalpy= 1.090489
Thermal correction to Gibbs Free Energy= 0.869456
Sum of electronic and zero-point Energies= -7646.899622
Sum of electronic and thermal Energies= -7646.812564
Sum of electronic and thermal Enthalpies= -7646.811620
Sum of electronic and thermal Free Energies= -7647.032653

	E (Thermal)	CV	S
	KCal/Mol	Cal/Mol-Kelvin	Cal/Mol-Kelvin
Total	683.700	330.902	465.203

Multipole moments

Electronic spatial extent (au): <R**2>= 106309.4107
Charge= 1.0000 electrons
Dipole moment (field-independent basis, Debye):
X= 3.9603 Y= 1.6473 Z= 7.0056 Tot= 8.2144
Quadrupole moment (field-independent basis, Debye-Ang):
XX= -544.6331 YY= -600.7971 ZZ= -537.8865
XY= 0.7440 XZ= 5.5712 YZ= 2.0027
Traceless Quadrupole moment (field-independent basis, Debye-Ang):
XX= 16.4725 YY= -39.6915 ZZ= 23.2191
XY= 0.7440 XZ= 5.5712 YZ= 2.0027
Octapole moment (field-independent basis, Debye-Ang**2):
XXX= -1253.6918 YYY= -1550.7872 ZZZ= -2363.2308 XYY= -506.3033
XXY= -599.3655 XXZ= -851.0904 XZZ= -616.0767 YZZ= -596.5933

YYZ= -889.2996 XYZ= 1.9838
Hexadecapole moment (field-independent basis, Debye-Ang**3):
XXXX= -50306.2217 YYYY= -26686.8502 ZZZZ= -55342.1343 XXXY= -1472.9395
XXXZ= -1864.6963 YYYX= -1192.8222 YYYZ= -2285.5631 ZZZX= -2567.5306
ZZZY= -2639.8503 XXYY= -13526.7792 XXZZ= -18129.0446 YYZZ= -14446.4364
XXYZ= -900.8515 YYXZ= -737.9858 ZZXY= -612.7407
N-N= 2.408688392625D+04 E-N=-6.603148463499D+04 KE= 7.362599744800D+03

# - Pt_3 cation (M06/Def2-svp)

Optimized structure

141
scf done: -7622.300664

Pt	7.486736	3.593933	2.066027
S	9.528549	2.420981	1.989481
P	6.040815	1.772547	2.106177
C	5.228920	1.442176	0.508757
C	7.679980	-0.406693	1.572761
H	7.775192	0.039698	0.576281
C	6.846139	0.186293	2.532788
C	6.744722	-0.397243	3.799886
H	6.088321	0.036367	4.560138
C	3.339716	1.861126	2.963501
H	3.057436	1.592061	1.941283
C	9.767234	1.721960	3.621548
C	4.593192	0.212781	0.274428
H	4.610839	-0.568689	1.042207
C	5.204222	2.426762	-0.485681
H	5.707763	3.384774	-0.317032
C	5.017761	2.351007	4.631237
H	6.068494	2.480701	4.916356
C	8.397171	-1.557854	1.869985
H	9.045983	-2.039971	1.134420
C	4.688365	1.989265	3.315078
C	2.337622	2.088412	3.903604
H	1.278665	2.004913	3.646952
C	4.027721	2.559667	5.581105
H	4.258602	2.847115	6.610489
F	9.009010	-3.174223	3.447315
F	1.750520	2.693948	6.086606
C	10.488756	0.529021	3.707731
H	10.896366	0.075903	2.798150
C	9.243842	2.300545	4.784647
H	8.682018	3.239625	4.712817
C	8.294103	-2.101744	3.146492
C	3.935253	-0.025179	-0.924961
H	3.432065	-0.973153	-1.130436
C	7.473296	-1.541259	4.114577
H	7.416580	-2.005304	5.102563
C	9.440471	1.692050	6.018283
H	9.039222	2.132918	6.935063
C	10.700067	-0.079755	4.940782
H	11.280235	-1.003993	5.020060
C	4.549349	2.197821	-1.691773
H	4.518727	2.949146	-2.484220
C	3.922301	0.974901	-1.893162
C	10.176954	0.507975	6.090022

C	2.696158	2.438838	5.197730
Cl	10.478521	-0.217016	7.634932
F	3.299593	0.751786	-3.036292
Pt	10.234489	4.672623	2.054302
S	10.233284	7.026581	1.996264
P	12.536868	4.340593	2.069070
C	13.211860	3.774685	0.473985
C	13.583419	6.840287	1.462189
H	13.118743	6.681481	0.482218
C	13.514175	5.841498	2.445128
C	14.120795	6.064115	3.685588
H	14.093563	5.295097	4.463269
C	13.834694	1.986595	2.968352
H	14.201889	1.856406	1.945905
C	10.744989	7.553338	3.630066
C	14.592647	3.833834	0.228414
H	15.268504	4.241006	0.988837
C	12.362071	3.259878	-0.511278
H	11.282260	3.224770	-0.333362
C	12.575240	3.224339	4.617859
H	11.935564	4.073905	4.885866
C	14.244156	8.035837	1.710767
H	14.319425	8.822005	0.955221
C	13.045998	3.093420	3.301599
C	14.151386	1.028621	3.928329
H	14.757406	0.151613	3.687444
C	12.898538	2.284248	5.586640
H	12.533691	2.357779	6.615148
F	15.419401	9.389591	3.215226
F	13.935267	0.259987	6.126659
C	11.469274	8.744070	3.720734
H	11.697042	9.310738	2.811743
C	10.479375	6.814525	4.789911
H	9.914742	5.877928	4.713750
C	14.821434	8.236303	2.960933
C	15.116625	3.377844	-0.973680
H	16.187488	3.411871	-1.188196
C	14.774361	7.264907	3.950242
H	15.248155	7.460275	4.915590
C	10.921559	7.269300	6.026131
H	10.716758	6.705906	6.940647
C	11.906005	9.211523	4.956221
H	12.460622	10.150770	5.039679
C	12.876404	2.801790	-1.720592
H	12.232737	2.400365	-2.506888
C	14.247293	2.866364	-1.933377
C	11.622719	8.474619	6.103451
C	13.674006	1.188516	5.221622
Cl	12.113014	9.079205	7.651263
F	14.741783	2.435305	-3.079548
Pt	7.927789	6.521624	2.063874
S	5.895823	5.336395	1.974150
P	7.084340	8.690446	2.111954
C	7.241254	9.589765	0.533889
C	4.394407	8.368472	1.509913
H	4.762631	8.034159	0.532998
C	5.298230	8.801345	2.492061
C	4.807591	9.220664	3.732908
H	5.492091	9.576973	4.508752
C	8.509275	10.956770	3.046981

H	8.441379	11.358958	2.031677
C	5.144331	5.513532	3.589788
C	6.490986	10.754018	0.305128
H	5.790502	11.114965	1.066570
C	8.125977	9.141078	-0.453155
H	8.706526	8.227765	-0.288497
C	8.049587	9.225181	4.667912
H	7.622310	8.248254	4.925074
C	3.028926	8.357409	1.758843
H	2.307085	8.033014	1.004997
C	7.928984	9.722284	3.360242
C	9.192847	11.685349	4.017302
H	9.661198	12.646587	3.791106
C	8.714511	9.950534	5.646748
H	8.830573	9.580506	6.669163
F	1.273666	8.713405	3.265245
F	9.972338	11.834500	6.218803
C	3.748803	5.524452	3.645392
H	3.168945	5.403108	2.724547
C	5.885792	5.666214	4.768393
H	6.981125	5.653080	4.720483
C	2.571631	8.760447	3.010101
C	6.626395	11.460931	-0.881982
H	6.052521	12.368701	-1.083601
C	3.441192	9.196221	3.999383
H	3.038022	9.512416	4.964893
C	5.236929	5.824909	5.987040
H	5.801396	5.939755	6.916160
C	3.092323	5.677469	4.862450
H	2.000094	5.676414	4.918119
C	8.268410	9.841161	-1.647103
H	8.948376	9.507435	-2.434307
C	7.516188	10.991889	-1.844310
C	3.841158	5.821612	6.027666
C	9.290453	11.168932	5.301821
Cl	3.032031	5.954841	7.554204
F	7.643769	11.657923	-2.977715

Energies

Zero-point correction= 1.002327 (Hartree/Particle)
Thermal correction to Energy= 1.089571
Thermal correction to Enthalpy= 1.090515
Thermal correction to Gibbs Free Energy= 0.867914
Sum of electronic and zero-point Energies= -7621.298337
Sum of electronic and thermal Energies= -7621.211093
Sum of electronic and thermal Enthalpies= -7621.210149
Sum of electronic and thermal Free Energies= -7621.432750

	E (Thermal)	CV	S
	KCal/Mol	Cal/Mol-Kelvin	Cal/Mol-Kelvin
Total	683.716	330.903	468.503

Multipole moments

Electronic spatial extent (au): <R**2>= 106288.3777
Charge= 1.0000 electrons
Dipole moment (field-independent basis, Debye):
X= 4.1258 Y= -1.2888 Z= 7.0194 Tot= 8.2435
Quadrupole moment (field-independent basis, Debye-Ang):

XX= -554.5888 YY= -610.5271 ZZ= -547.4334
XY= -1.7011 XZ= 6.2047 YZ= -1.8564
Traceless Quadrupole moment (field-independent basis, Debye-Ang):
XX= 16.2610 YY= -39.6774 ZZ= 23.4164
XY= -1.7011 XZ= 6.2047 YZ= -1.8564
Octapole moment (field-independent basis, Debye-Ang**2):
XXX= -1291.8150 YYY= 1437.2702 ZZZ= -2404.3708 XYY= -525.9457
XXY= 569.7322 XXZ= -860.0061 XZZ= -622.8276 YZZ= 571.6795
YYZ= -895.0673 XYZ= -1.8058
Hexadecapole moment (field-independent basis, Debye-Ang**3):
XXXX= -50503.9279 YYYY= -26599.1413 ZZZZ= -55630.4920 XXXY= 1341.7433
XXXZ= -2100.7851 YYYX= 1236.4671 YYYZ= 2074.3349 ZZZX= -2834.9798
ZZZY= 2575.4271 XXYY= -13571.1674 XXZZ= -18203.7061 YYZZ= -14426.2845
XXYZ= 827.5657 YYXZ= -803.0244 ZZXY= 548.7815
N-N= 2.405355617968D+04 E-N=-6.588900722448D+04 KE= 7.340257576653D+03

# - Pd_2Pt cation (M06/Def2-svp)

Optimized structure

141
scf done: -7639.368469

C	5.282176	8.805120	2.421644
C	4.791533	9.316258	3.627257
H	5.475921	9.725988	4.376120
C	3.424403	9.315059	3.893212
H	3.020696	9.704567	4.831141
C	2.555814	8.800504	2.941659
C	3.013911	8.306275	1.723161
H	2.291981	7.927654	0.995105
C	4.378274	8.304790	1.471799
H	4.746684	7.907217	0.518997
C	7.236611	9.581025	0.462753
C	6.454239	10.718321	0.207884
H	5.723399	11.061200	0.948663
C	6.596449	11.422467	-0.980248
H	5.997967	12.309383	-1.202118
C	7.524810	10.977639	-1.917054
C	8.310991	9.854733	-1.692528
H	9.021491	9.539367	-2.460074
C	8.161795	9.157049	-0.498448
H	8.769916	8.265882	-0.314039
C	8.496341	10.945808	2.984020
H	8.446260	11.340751	1.964593
C	9.164178	11.678917	3.961842
H	9.639386	12.637381	3.737985
C	9.235847	11.171601	5.251692
C	8.650091	9.957084	5.595129
H	8.745491	9.593788	6.622241
C	8.000260	9.227160	4.609740
H	7.561721	8.254465	4.865711
C	7.904941	9.716983	3.297084
C	10.715720	7.605830	3.565971
C	11.393861	8.824183	3.661738
H	11.560525	9.424638	2.761043
C	11.841590	9.287709	4.894398
H	12.360870	10.246732	4.980838
C	11.610515	8.524641	6.036691

C	10.942638	7.301152	5.956963
H	10.771605	6.720418	6.867505
C	10.493244	6.847852	4.722851
H	9.957162	5.894550	4.645763
F	1.257931	8.777100	3.195456
F	7.658215	11.639906	-3.051663
F	9.902984	11.842015	6.175661
Cl	12.119766	9.121302	7.581326
P	7.067371	8.689215	2.043453
S	10.216189	7.061729	1.937574
C	6.867982	0.148332	2.440209
C	6.761408	-0.512680	3.668061
H	6.103144	-0.124684	4.451188
C	7.489178	-1.674703	3.913561
H	7.427452	-2.199939	4.870043
C	8.314311	-2.173874	2.916642
C	8.423317	-1.550763	1.676972
H	9.076713	-1.985454	0.916325
C	7.707454	-0.383661	1.449559
H	7.807892	0.123555	0.482886
C	5.136974	1.441937	0.545793
C	4.507613	0.207996	0.318936
H	4.583627	-0.591331	1.064725
C	3.786090	-0.012608	-0.846838
H	3.286610	-0.963834	-1.046367
C	3.702983	1.008752	-1.788996
C	4.320991	2.237261	-1.594251
H	4.233715	3.006155	-2.365465
C	5.039127	2.448795	-0.421260
H	5.535045	3.411563	-0.257495
C	3.418081	1.802978	3.153568
H	3.055934	1.526176	2.158585
C	2.493609	2.022354	4.171999
H	1.418091	1.928140	4.002426
C	2.951705	2.383243	5.431467
C	4.308832	2.512672	5.706675
H	4.619577	2.802078	6.714242
C	5.220451	2.311897	4.679587
H	6.291138	2.448060	4.878263
C	4.789125	1.947815	3.394044
C	5.169646	5.498003	3.562430
C	3.776256	5.422005	3.648608
H	3.185161	5.264950	2.739873
C	3.138219	5.534151	4.879692
H	2.050690	5.453712	4.959656
C	3.899508	5.733680	6.029169
C	5.289542	5.841924	5.956354
H	5.864850	6.006496	6.871294
C	5.921478	5.716752	4.724964
H	7.014007	5.784333	4.652893
F	9.028441	-3.263426	3.152748
F	3.019809	0.800671	-2.900356
F	2.077381	2.646607	6.388487
Cl	3.117520	5.803406	7.573172
P	6.053906	1.750931	2.091216
S	5.900460	5.340117	1.940210
Pd	7.501065	3.612111	2.008369
C	13.544515	5.846723	2.553968
C	14.128011	6.039943	3.810381
H	14.088574	5.252094	4.568409

C	14.770508	7.236837	4.117140
H	15.223829	7.411177	5.096324
C	14.833051	8.232389	3.152716
C	14.282438	8.060170	1.886518
H	14.368357	8.865134	1.152160
C	13.627899	6.870665	1.597700
H	13.176661	6.736250	0.607786
C	13.278114	3.822942	0.534791
C	14.663909	3.881632	0.319345
H	15.324288	4.272085	1.101691
C	15.211825	3.444676	-0.879082
H	16.287191	3.478250	-1.069807
C	14.361691	2.952567	-1.865795
C	12.986472	2.888126	-1.683072
H	12.358610	2.501498	-2.489261
C	12.448120	3.327418	-0.477135
H	11.364258	3.290431	-0.323909
C	13.806711	1.971234	2.996227
H	14.186703	1.856122	1.976535
C	14.093493	0.987590	3.939583
H	14.688000	0.105708	3.687996
C	13.601054	1.128230	5.229415
C	12.839949	2.229602	5.606438
H	12.463313	2.288289	6.631688
C	12.545259	3.195023	4.653253
H	11.915418	4.047310	4.935096
C	13.033000	3.085517	3.340898
C	9.740585	1.760982	3.555505
C	10.427915	0.547499	3.654770
H	10.838079	0.085168	2.751099
C	10.602225	-0.068281	4.889509
H	11.156999	-1.007358	4.977179
C	10.078382	0.533002	6.031658
C	9.370687	1.733851	5.949354
H	8.964915	2.183414	6.859959
C	9.209224	2.347332	4.713044
H	8.665789	3.297522	4.634227
F	15.420546	9.381481	3.447535
F	14.879151	2.539922	-3.008866
F	13.831729	0.176043	6.118239
Cl	10.347489	-0.194261	7.581352
P	12.562583	4.363324	2.121911
S	9.546069	2.472353	1.931899
Pd	10.231308	4.710820	2.029412
Pt	7.922000	6.532577	2.004142

Energies

Zero-point correction= 1.002067 (Hartree/Particle)
Thermal correction to Energy= 1.089369
Thermal correction to Enthalpy= 1.090314
Thermal correction to Gibbs Free Energy= 0.867822
Sum of electronic and zero-point Energies= -7638.366402
Sum of electronic and thermal Energies= -7638.279100
Sum of electronic and thermal Enthalpies= -7638.278156
Sum of electronic and thermal Free Energies= -7638.500647

	E (Thermal)	CV	S
	KCal/Mol	Cal/Mol-Kelvin	Cal/Mol-Kelvin
Total	683.590	331.089	468.273

Multipole moments

Electronic spatial extent (au): <R**2>= 106197.0909
Charge= 1.0000 electrons
Dipole moment (field-independent basis, Debye):
X= 4.6729 Y= -1.5663 Z= 7.0633 Tot= 8.6127
Quadrupole moment (field-independent basis, Debye-Ang):
XX= -545.6196 YY= -604.4337 ZZ= -541.2409
XY= -1.3893 XZ= 6.6156 YZ= -3.0130
Traceless Quadrupole moment (field-independent basis, Debye-Ang):
XX= 18.1452 YY= -40.6690 ZZ= 22.5238
XY= -1.3893 XZ= 6.6156 YZ= -3.0130
Octapole moment (field-independent basis, Debye-Ang**2):
XXX= -1211.8984 YYY= 1510.6591 ZZZ= -2344.6759 XYY= -509.1626
XXY= 583.5652 XXZ= -843.9114 XZZ= -603.2231 YZZ= 579.2473
YYZ= -889.9500 XYZ= -1.2364
Hexadecapole moment (field-independent basis, Debye-Ang**3):
XXXX= -50239.1485 YYYY= -26656.6398 ZZZZ= -55284.5535 XXXY= 1275.8492
XXXZ= -1813.3535 YYYX= 1202.5696 YYYZ= 2359.7185 ZZZX= -2569.7933
ZZZY= 2559.2772 XXYY= -13504.5522 XXZZ= -18111.0078 YYZZ= -14473.3823
XXYZ= 859.3320 YYXZ= -722.9802 ZZXY= 554.8376
N-N= 2.406595070565D+04 E-N=-6.596440967628D+04 KE= 7.355158557193D+03

# - $PdPt_2$ cation (M06/Def2-svp)

Optimized structure

141
scf done: -7630.834799

C	7.219801	9.610616	0.571085
C	6.475865	10.780769	0.352383
H	5.776972	11.138081	1.116992
C	6.616095	11.498001	-0.828065
H	6.047566	12.410925	-1.021512
C	7.504177	11.033635	-1.794213
C	8.249965	9.877069	-1.607697
H	8.928706	9.547016	-2.397531
C	8.102367	9.166720	-0.420197
H	8.678837	8.249019	-0.264209
C	5.277247	8.816516	2.525339
C	4.366216	8.376780	1.552636
H	4.728907	8.020988	0.581057
C	3.000873	8.388507	1.802424
H	2.274124	8.060610	1.054768
C	2.550108	8.819181	3.046776
C	3.426736	9.258983	4.027923
H	3.028861	9.597962	4.987916
C	4.793357	9.260692	3.760171
H	5.483420	9.618760	4.530348
C	7.913220	9.695124	3.396721
C	8.029854	9.179463	4.697662
H	7.599687	8.199612	4.940573
C	8.689107	9.891979	5.690091
H	8.797801	9.507984	6.708380
C	9.264690	11.116325	5.364978
C	9.175656	11.648085	4.086341
H	9.643920	12.613048	3.875813
C	8.497403	10.932367	3.102888

H	8.432279	11.348736	2.092903
C	10.739322	7.536944	3.590202
C	10.548773	6.768650	4.746827
H	10.024817	5.807702	4.672781
C	11.017443	7.219331	5.974609
H	10.873020	6.629434	6.883904
C	11.669033	8.451937	6.051177
C	11.859909	9.229286	4.910947
H	12.363871	10.196894	4.993852
C	11.395956	8.767363	3.683550
H	11.532545	9.379317	2.785603
F	9.939093	11.772878	6.294318
F	1.251426	8.795575	3.303050
F	7.636133	11.710131	-2.921256
Cl	12.204107	9.040979	7.590177
P	7.061592	8.684033	2.134857
S	10.218445	6.999284	1.967310
C	5.253831	1.405920	0.495303
C	4.612501	0.177806	0.269689
H	4.616026	-0.594117	1.047213
C	3.964988	-0.069930	-0.933295
H	3.457238	-1.016803	-1.132533
C	3.968521	0.918697	-1.913427
C	4.601662	2.139894	-1.720451
H	4.584053	2.881830	-2.522075
C	5.245969	2.378837	-0.510678
H	5.754775	3.334986	-0.347391
C	6.842792	0.169320	2.553630
C	7.686765	-0.434890	1.609249
H	7.793406	0.000655	0.609201
C	8.399441	-1.583224	1.927073
H	9.056038	-2.073727	1.204062
C	8.281968	-2.112964	3.208304
C	7.449146	-1.542652	4.160279
H	7.380087	-1.996610	5.152165
C	6.724924	-0.401812	3.825018
H	6.058559	0.037957	4.572786
C	4.677898	1.993726	3.285703
C	4.987440	2.342624	4.610202
H	6.034661	2.442304	4.919696
C	3.981562	2.581273	5.536271
H	4.195839	2.859775	6.571653
C	2.656696	2.509810	5.119016
C	2.318164	2.176857	3.814987
H	1.263763	2.133791	3.531097
C	3.334777	1.916251	2.899567
H	3.069311	1.663154	1.868849
C	5.179244	5.498782	3.580071
C	5.951127	5.610102	4.744001
H	7.044290	5.559002	4.670587
C	5.337035	5.775588	5.979497
H	5.926715	5.853660	6.896709
C	3.943042	5.824121	6.051799
C	3.164249	5.737535	4.900564
H	2.074295	5.784628	4.979719
C	3.786823	5.573816	3.667381
H	3.182832	5.499049	2.757087
F	1.699445	2.798450	5.985340
F	8.992978	-3.181941	3.528925
F	3.355833	0.685975	-3.059877

Cl	3.174787	5.947027	7.600144
P	6.044742	1.750659	2.099317
Pt	7.485936	3.570372	2.051528
S	5.895358	5.300871	1.953751
C	13.210161	3.746281	0.472122
C	14.591691	3.807611	0.230733
H	15.262986	4.231055	0.986240
C	15.122128	3.333832	-0.961559
H	16.193621	3.369123	-1.172636
C	14.258537	2.803066	-1.915935
C	12.886976	2.736642	-1.707385
H	12.247996	2.320450	-2.489801
C	12.366076	3.212159	-0.507855
H	11.285607	3.177028	-0.334158
C	13.495475	5.850769	2.409511
C	13.569584	6.833324	1.410818
H	13.114082	6.656870	0.429594
C	14.219733	8.036921	1.646743
H	14.296997	8.811598	0.879782
C	14.779996	8.262648	2.900543
C	14.727923	7.307798	3.905299
H	15.186156	7.523521	4.873888
C	14.086448	6.097839	3.652847
H	14.054594	5.342204	4.443379
C	13.029754	3.120234	3.315993
C	12.532681	3.269114	4.620646
H	11.873870	4.113624	4.859291
C	12.856803	2.354921	5.613751
H	12.474293	2.443397	6.634598
C	13.659608	1.267406	5.284171
C	14.161351	1.089891	4.002283
H	14.787341	0.219630	3.789873
C	13.843569	2.021926	3.017612
H	14.230049	1.878826	2.004050
C	9.772403	1.703339	3.618834
C	9.231048	2.274655	4.777089
H	8.657168	3.205589	4.699907
C	9.422017	1.668101	6.012498
H	9.004713	2.103076	6.924946
C	10.171584	0.492860	6.091362
C	10.714764	-0.086894	4.947145
H	11.302521	-1.005795	5.032034
C	10.508137	0.519426	3.712006
H	10.925604	0.068863	2.805541
F	13.925422	0.363788	6.212467
F	15.365127	9.424855	3.142736
F	14.758769	2.355909	-3.053236
Cl	10.458630	-0.233658	7.638127
P	12.528237	4.339089	2.053660
Pt	10.229002	4.654328	2.032072
S	9.531751	2.394991	1.983795
Pd	7.921522	6.485998	2.048029

Energies

Zero-point correction= 1.002923 (Hartree/Particle)
Thermal correction to Energy= 1.089927
Thermal correction to Enthalpy= 1.090871
Thermal correction to Gibbs Free Energy= 0.870197
Sum of electronic and zero-point Energies= -7629.831876

Sum of electronic and thermal Energies= -7629.744872
Sum of electronic and thermal Enthalpies= -7629.743928
Sum of electronic and thermal Free Energies= -7629.964602

	E (Thermal)	CV	S
	KCal/Mol	Cal/Mol-Kelvin	Cal/Mol-Kelvin
Total	683.939	330.621	464.447

Multipole moments

Electronic spatial extent (au): <R**2>= 106211.4878
Charge= 1.0000 electrons
Dipole moment (field-independent basis, Debye):
X= 3.6120 Y= -1.3514 Z= 7.0382 Tot= 8.0255
Quadrupole moment (field-independent basis, Debye-Ang):
XX= -552.1265 YY= -607.2346 ZZ= -544.5557
XY= -2.0113 XZ= 5.7329 YZ= -1.9204
Traceless Quadrupole moment (field-independent basis, Debye-Ang):
XX= 15.8458 YY= -39.2624 ZZ= 23.4166
XY= -2.0113 XZ= 5.7329 YZ= -1.9204
Octapole moment (field-independent basis, Debye-Ang**2):
XXX= -1304.9079 YYY= 1475.4201 ZZZ= -2373.3481 XYY= -530.0392
XXY= 573.6292 XXZ= -852.0025 XZZ= -628.5225 YZZ= 584.6784
YYZ= -880.0184 XYZ= -1.8501
Hexadecapole moment (field-independent basis, Debye-Ang**3):
XXXX= -50587.3569 YYYY= -26652.9985 ZZZZ= -55272.7185 XXXY= 1386.6414
XXXZ= -2094.5753 YYYX= 1326.0696 YYYZ= 2023.3342 ZZZX= -2806.3274
ZZZY= 2561.8151 XXYY= -13623.7795 XXZZ= -18157.4701 YYZZ= -14336.7095
XXYZ= 823.2565 YYXZ= -798.0264 ZZXY= 580.1475
N-N= 2.406998908896D+04 E-N=-6.594713410836D+04 KE= 7.347706901635D+03

# - Pd_3 cation 1 – Li+ adduct (M06/Def2-svp)

143
scf done: -7655.183552

Pd	-0.036589	0.128345	0.032910
Pd	0.043618	0.149788	3.024618
Pd	2.546455	0.195874	1.473022
S	-1.808828	0.055825	1.585567
S	2.259282	0.060792	3.798020
S	2.141212	0.035038	-0.834968
P	-1.249181	0.159752	-2.018211
P	-1.126798	0.159226	5.094716
P	4.920612	0.193540	1.418184
C	-2.654945	1.623980	1.580642
C	2.705593	1.600993	4.571759
C	2.560127	1.542255	-1.685912
C	3.556415	1.466179	-2.666441
C	3.939640	2.598684	-3.375750
C	3.308606	3.817498	-3.125391
C	2.293945	3.908338	-2.168793
C	1.931776	2.773200	-1.456533
C	3.026729	1.563091	5.933481
C	3.379398	2.725031	6.609508
C	3.432363	3.936364	5.919134
C	3.147942	3.988400	4.552242
C	2.787256	2.822027	3.888712
C	-3.944978	1.655126	1.039016
C	-4.658783	2.846344	0.980047

C	-4.082925	4.024130	1.459256
C	-2.817278	4.004801	2.048319
C	-2.117221	2.804305	2.114185
C	5.686423	0.609689	3.019807
C	5.619136	-0.337118	4.054762
C	6.109720	-0.038610	5.317534
C	6.651112	1.224415	5.547908
C	6.741789	2.175130	4.540273
C	6.258282	1.861678	3.273091
C	5.598743	-1.432714	0.970616
C	6.919518	-1.766903	1.314356
C	7.464648	-2.981195	0.920690
C	6.679745	-3.865237	0.184754
C	5.368310	-3.562725	-0.164264
C	4.831358	-2.342895	0.231244
C	5.586131	1.388292	0.213881
C	6.605613	1.041537	-0.681932
C	7.110319	1.980195	-1.576511
C	6.587762	3.267187	-1.569191
C	5.576407	3.642632	-0.688853
C	5.073302	2.694506	0.191451
F	7.192315	-5.018154	-0.187717
F	7.082100	1.523017	6.757862
F	7.040874	4.151531	-2.434642
Cl	3.825346	5.385540	6.767271
C	-2.898001	0.552497	4.896768
C	-3.715135	-0.394937	4.257890
C	-5.042720	-0.108785	3.975332
C	-5.550606	1.141561	4.320715
C	-4.772940	2.089539	4.971780
C	-3.444320	1.788644	5.260634
C	-1.079652	-1.462959	5.911516
C	-2.085187	-1.828891	6.821638
C	-2.019394	-3.042825	7.491608
C	-0.943572	-3.891614	7.244455
C	0.066140	-3.553338	6.350061
C	-0.006814	-2.335420	5.684060
C	-0.466768	1.359981	6.296018
C	-0.155197	0.987266	7.610159
C	0.342087	1.923064	8.512436
C	0.526142	3.234478	8.092881
C	0.218492	3.636751	6.796044
C	-0.264810	2.692179	5.902169
F	-0.880927	-5.044832	7.874461
F	-6.801090	1.429821	4.017686
F	1.029903	4.117354	8.931082
Cl	-4.927199	5.518379	1.295010
C	-0.198395	0.424648	-3.485455
C	0.709292	-0.587392	-3.838231
C	1.581401	-0.414468	-4.902715
C	1.554793	0.788520	-5.605619
C	0.655525	1.798327	-5.291088
C	-0.222167	1.611401	-4.226600
C	-2.136271	-1.394129	-2.346416
C	-2.483876	-1.744995	-3.661757
C	-3.215508	-2.897277	-3.914044
C	-3.595444	-3.701994	-2.842980
C	-3.262425	-3.379861	-1.531831
C	-2.530293	-2.223462	-1.288021
C	-2.502839	1.485503	-2.064298

C	-3.822652	1.239453	-2.464691
C	-4.754403	2.272230	-2.507163
C	-4.359271	3.552963	-2.142428
C	-3.052419	3.830853	-1.748963
C	-2.133990	2.791293	-1.705614
F	-4.286043	-4.796706	-3.077972
F	2.411108	0.970771	-6.591681
F	-5.246963	4.526958	-2.138446
Cl	3.793704	5.230052	-3.986052
H	4.788819	-4.291203	-0.736139
H	7.535154	-1.070007	1.893179
H	3.798845	-2.099931	-0.041070
H	7.016186	0.027175	-0.687742
H	5.183675	-1.326354	3.872856
H	4.265066	2.977009	0.877726
H	7.195864	3.144759	4.759903
H	6.081351	-0.763460	6.134955
H	6.342515	2.606826	2.476027
H	8.490012	-3.262135	1.173345
H	5.194987	4.666911	-0.727402
H	7.906580	1.731793	-2.282591
H	2.612157	2.849057	2.803600
H	2.984909	0.610614	6.472552
H	3.614483	2.704743	7.677496
H	3.218613	4.943087	4.024149
H	0.888132	-4.254151	6.187102
H	-2.927504	-1.156284	7.015264
H	0.785988	-2.061896	4.979744
H	-0.300904	-0.045909	7.941236
H	-3.312306	-1.375237	3.978599
H	-0.489998	3.002492	4.874934
H	-5.219661	3.047232	5.250632
H	-5.699898	-0.835202	3.490735
H	-2.838420	2.533545	5.785371
H	-2.787670	-3.350683	8.204902
H	0.382609	4.680211	6.513290
H	0.590372	1.652688	9.541864
H	-1.170429	2.766690	2.675672
H	-4.386682	0.732156	0.646703
H	-5.665833	2.877479	0.554262
H	-2.402247	4.927716	2.462395
H	-3.576756	-4.046116	-0.725120
H	-2.187294	-1.107962	-4.502014
H	-2.262989	-1.967337	-0.257743
H	-4.134338	0.229525	-2.749603
H	0.730918	-1.528619	-3.276568
H	-1.103404	3.001894	-1.394272
H	0.653426	2.715722	-5.885186
H	2.287700	-1.192157	-5.204067
H	-0.936904	2.404890	-3.987332
H	-3.499248	-3.189406	-4.928072
H	-2.791061	4.855343	-1.469818
H	-5.788340	2.100671	-2.817339
H	1.104632	2.837468	-0.735649
H	4.044715	0.505970	-2.864889
H	4.729632	2.547118	-4.130522
H	1.797904	4.868002	-1.999299
Bq	0.842271	0.211238	1.503263
Li	0.610676	2.229246	1.466058

Energies

Zero-point correction= 1.004135 (Hartree/Particle)
Thermal correction to Energy= 1.094425
Thermal correction to Enthalpy= 1.095369
Thermal correction to Gibbs Free Energy= 0.847782
Sum of electronic and zero-point Energies= -7654.179417
Sum of electronic and thermal Energies= -7654.089127
Sum of electronic and thermal Enthalpies= -7654.088183
Sum of electronic and thermal Free Energies= -7654.335770

	E (Thermal)	CV	S
	KCal/Mol	Cal/Mol-Kelvin	Cal/Mol-Kelvin
Total	686.762	340.000	521.092

# - Pd_3 cation 1 – Li+ adduct with $BF4^-$ (M06/Def2-svp)

147
scf done: -8079.482170

C	4.719596	-2.617480	0.521489
C	5.474616	-1.438128	0.447969
C	6.640039	-1.411608	-0.330363
C	7.048650	-2.550344	-1.014985
C	6.285616	-3.710189	-0.916778
C	5.124602	-3.761791	-0.153509
P	4.932650	0.000096	1.416456
C	5.629156	-0.239466	3.085310
C	5.589393	0.824663	3.998890
C	6.053673	0.661283	5.296170
C	6.532689	-0.587137	5.684813
C	6.580788	-1.658178	4.804152
C	6.129283	-1.478617	3.499829
F	6.934508	-0.757777	6.933188
F	6.674661	-4.787126	-1.574324
Pd	2.557165	0.146015	1.422830
S	2.169834	0.272959	-0.897811
C	2.581906	-1.291500	-1.638848
C	1.962058	-2.481747	-1.251047
C	2.277922	-3.672639	-1.891818
C	3.230007	-3.666406	-2.910937
C	3.875213	-2.486828	-3.289433
C	3.547590	-1.296516	-2.652266
Cl	3.636331	-5.146687	-3.711087
Pd	-0.007272	0.104648	-0.006454
P	-1.216779	-0.048592	-2.047582
C	-2.369814	-1.453172	-2.004290
C	-3.637228	-1.382993	-2.598198
C	-4.473567	-2.493722	-2.592943
C	-4.033245	-3.668566	-1.991520
C	-2.782726	-3.761915	-1.390119
C	-1.956913	-2.645147	-1.390779
F	-4.833394	-4.718931	-1.980575
Pd	0.027320	0.107063	2.933091
P	-1.125074	-0.056607	5.015003
C	-0.504257	-1.464964	5.982541
C	-0.375419	-1.414217	7.377272
C	0.063558	-2.531799	8.079283
C	0.370253	-3.693722	7.377587
C	0.243895	-3.771112	5.995135

C	-0.184640	-2.647728	5.300304
F	0.809640	-4.747029	8.042269
S	-1.832092	0.209685	1.485139
C	-2.653092	-1.368141	1.534748
C	-1.974095	-2.542743	1.865724
C	-2.663518	-3.745143	1.953400
C	-4.030011	-3.767710	1.676604
C	-4.714582	-2.603663	1.319468
C	-4.024076	-1.399690	1.255330
Cl	-4.889263	-5.269253	1.734179
S	2.233543	0.272684	3.747067
C	2.706806	-1.286856	4.457642
C	2.673957	-2.478950	3.729876
C	3.072337	-3.669159	4.324101
C	3.472822	-3.661151	5.659935
C	3.511494	-2.475582	6.397233
C	3.130338	-1.285146	5.791727
Cl	3.875490	-5.152457	6.441165
C	-2.232772	1.403446	-2.486290
C	-2.444544	1.766698	-3.824353
C	-3.298644	2.814985	-4.147382
C	-3.946400	3.496415	-3.123226
C	-3.762756	3.153024	-1.788592
C	-2.903154	2.106577	-1.475711
F	-4.755613	4.494887	-3.423998
C	-0.138080	-0.330046	-3.491139
C	0.680083	0.715259	-3.947074
C	1.571034	0.512538	-4.991274
C	1.662837	-0.756122	-5.558274
C	0.869807	-1.808909	-5.125862
C	-0.035155	-1.589757	-4.091209
F	2.542637	-0.963013	-6.523162
C	-0.998297	1.393809	6.118646
C	-2.057290	1.773377	6.956250
C	-1.910955	2.829317	7.849193
C	-0.694974	3.500361	7.906039
C	0.376179	3.138993	7.096382
C	0.217389	2.086914	6.202209
F	-0.552093	4.506191	8.749100
C	-2.914730	-0.351662	4.813543
C	-3.729579	0.679782	4.321282
C	-5.079433	0.462660	4.082694
C	-5.605334	-0.806727	4.308113
C	-4.823615	-1.846215	4.790059
C	-3.475684	-1.612206	5.046289
F	-6.880585	-1.028795	4.038031
C	5.837926	1.429931	0.732614
C	7.101510	1.791734	1.222869
C	7.818777	2.827888	0.635515
C	7.267102	3.498763	-0.449554
C	6.020397	3.157211	-0.961764
C	5.309237	2.122579	-0.364812
F	7.942353	4.485332	-1.009410
Li	0.806951	2.203947	1.447108
H	-3.305927	1.672129	4.126677
H	-2.857004	-2.432646	5.423242
H	-0.617167	-0.499594	7.928320
H	-3.009415	1.234587	6.921416
H	1.059496	1.794170	5.564830
H	-0.258440	-2.713334	4.209138

H	-5.736351	1.252749	3.710425
H	0.176634	-2.520595	9.166089
H	0.503339	-4.695386	5.470826
H	-4.552600	-0.478659	0.985646
H	-0.891552	-2.549915	2.025913
H	-2.724006	3.140927	8.509527
H	-5.275129	-2.829915	4.943947
H	-2.127273	-4.663868	2.206319
H	-5.785048	-2.649864	1.101011
H	1.315811	3.690654	7.179322
H	0.614990	1.707683	-3.485744
H	-0.663710	-2.418647	-3.750783
H	-3.980418	-0.457763	-3.072410
H	-1.944712	1.219451	-4.629972
H	-2.764072	1.821804	-0.426601
H	-0.981287	-2.729235	-0.898453
H	2.210478	1.313384	-5.370894
H	-5.466213	-2.468891	-3.049211
H	-2.470784	-4.693066	-0.908567
H	4.040534	-0.363658	-2.947463
H	1.245551	-2.509143	-0.423816
H	-3.477265	3.112892	-5.183472
H	0.978012	-2.789875	-5.596069
H	1.798661	-4.604440	-1.580590
H	4.623354	-2.510577	-4.086266
H	-4.298369	3.711930	-1.017316
H	5.195388	1.800183	3.690095
H	6.163734	-2.322083	2.802795
H	7.240143	-0.499155	-0.407304
H	7.539279	1.253991	2.070200
H	4.329406	1.841395	-0.766794
H	3.798665	-2.673678	1.112279
H	6.043279	1.477927	6.022268
H	7.953039	-2.559159	-1.628441
H	4.529271	-4.678056	-0.103912
H	3.158476	-0.347711	6.358384
H	2.298557	-2.508278	2.702263
H	8.804669	3.125271	1.001249
H	6.960324	-2.622158	5.153050
H	3.029329	-4.601202	3.753756
H	3.834154	-2.497130	7.441797
H	5.628593	3.709257	-1.819441
B	0.889923	-4.664106	1.452668
F	0.808968	-6.021932	1.476217
F	2.214966	-4.230739	1.242431
F	0.086386	-4.128575	0.420235
F	0.445042	-4.113826	2.676823

Energies

Zero-point correction= 1.023222 (Hartree/Particle)
Thermal correction to Energy= 1.116925
Thermal correction to Enthalpy= 1.117870
Thermal correction to Gibbs Free Energy= 0.886197
Sum of electronic and zero-point Energies= -8078.458948
Sum of electronic and thermal Energies= -8078.365244
Sum of electronic and thermal Enthalpies= -8078.364300
Sum of electronic and thermal Free Energies= -8078.595973

E (Thermal) CV S

	KCal/Mol	Cal/Mol-Kelvin	Cal/Mol-Kelvin
Total	700.881	354.662	487.597

# - Pt_3 cation – Li+ adduct (M06/Def2-svp)

143
scf done: -7629.596733

Pt	-0.029688	0.083135	-0.020321
Pt	-0.023204	0.076087	2.939320
Pt	2.544626	0.103048	1.450442
S	-1.877097	0.124157	1.469433
S	2.190670	0.111308	3.788377
S	2.172326	0.140261	-0.889012
P	-1.194404	0.029033	-2.071194
P	-1.171383	0.023243	4.997028
P	4.899930	0.069040	1.477159
C	-2.592788	-1.513521	1.516439
C	2.607880	-1.540429	4.333668
C	2.507952	-1.505142	-1.503279
C	1.870943	-2.649478	-1.008302
C	2.182641	-3.898582	-1.528862
C	3.145444	-4.005163	-2.536462
C	3.772456	-2.868919	-3.047102
C	3.447122	-1.618486	-2.531798
C	-1.855206	-2.662316	1.826041
C	-2.480347	-3.902147	1.863063
C	-3.844264	-3.995539	1.572712
C	-4.587720	-2.855191	1.269890
C	-3.960240	-1.613971	1.248169
C	2.464141	-2.670616	3.519345
C	2.843132	-3.920883	3.990407
C	3.359313	-4.043023	5.283876
C	3.511858	-2.921303	6.098463
C	3.137100	-1.670645	5.619304
C	-1.066834	1.577375	5.938792
C	-2.102702	1.958461	6.805342
C	-1.986242	3.102588	7.585242
C	-0.824190	3.862602	7.499233
C	0.222634	3.503098	6.655823
C	0.095210	2.359971	5.876508
C	-2.951979	-0.326811	4.830115
C	-3.758293	0.623841	4.184107
C	-5.110166	0.385931	3.984111
C	-5.651844	-0.821637	4.419444
C	-4.881379	-1.773576	5.073842
C	-3.528641	-1.519831	5.281339
C	-0.482345	-1.272316	6.073625
C	-0.069944	-1.000608	7.383826
C	0.497269	-2.003426	8.165306
C	0.643207	-3.274936	7.625835
C	0.218335	-3.579515	6.335427
C	-0.330551	-2.569800	5.558157
F	-6.929138	-1.063322	4.201556
F	-0.709759	4.948176	8.233794
Cl	-4.602304	-5.545276	1.542401
F	1.224418	-4.217618	8.341665
C	-2.040935	1.588862	-2.474412
C	-2.210696	1.988896	-3.808160

C	-2.936583	3.133486	-4.115368
C	-3.499105	3.873344	-3.080208
C	-3.356339	3.493080	-1.749074
C	-2.624866	2.349864	-1.451434
C	-0.129524	-0.343459	-3.502631
C	0.830916	0.604827	-3.890124
C	1.706219	0.341230	-4.933494
C	1.631630	-0.891434	-5.578391
C	0.681836	-1.841509	-5.227918
C	-0.202122	-1.560958	-4.190166
C	-2.485465	-1.251601	-2.050007
C	-3.801862	-0.976310	-2.440535
C	-4.770521	-1.975101	-2.405415
C	-4.409072	-3.247617	-1.981713
C	-3.103426	-3.554011	-1.606203
C	-2.148068	-2.548179	-1.629927
F	2.486342	-1.158209	-6.546107
F	-4.186159	4.958408	-3.365068
Cl	3.595794	-5.567130	-3.116355
F	-5.329650	-4.188331	-1.905269
C	5.679404	1.613763	0.913809
C	6.933052	2.000169	1.412766
C	7.573504	3.128776	0.917095
C	6.955503	3.869057	-0.085560
C	5.717203	3.504042	-0.605182
C	5.082717	2.375193	-0.101034
C	5.585180	-0.239175	3.138462
C	5.440974	0.759241	4.114799
C	5.918122	0.564647	5.402929
C	6.526327	-0.648673	5.717679
C	6.681589	-1.653874	4.772725
C	6.212241	-1.442810	3.479349
C	5.554896	-1.243072	0.398918
C	6.583065	-0.992823	-0.518660
C	7.064039	-2.012960	-1.334131
C	6.508104	-3.281127	-1.223672
C	5.493516	-3.561172	-0.312242
C	5.015010	-2.536041	0.491541
F	6.955832	-0.847670	6.948574
F	7.555470	4.941366	-0.555607
Cl	3.776588	-5.603901	5.889273
F	6.931670	-4.242265	-2.019715
H	-3.328011	1.571617	3.840051
H	-2.925571	-2.264204	5.809586
H	-0.184470	0.002709	7.806313
H	-3.013076	1.355131	6.879449
H	0.923905	2.060847	5.225558
H	-0.648124	-2.796361	4.532348
H	-5.761341	1.116113	3.497033
H	0.833322	-1.815510	9.188132
H	0.352399	-4.597859	5.959469
H	-4.538041	-0.716463	1.003991
H	-0.781281	-2.579919	2.034097
H	-2.780635	3.418917	8.265521
H	-5.351639	-2.698450	5.416672
H	-1.918789	-4.808996	2.102929
H	-5.653295	-2.950149	1.042962
H	1.119284	4.127084	6.629213
H	0.889828	1.571877	-3.377616
H	-0.958156	-2.305865	-3.925445

H	-4.083535	0.027077	-2.775854
H	-1.777172	1.398562	-4.621746
H	-2.527431	2.033068	-0.407176
H	-1.119917	-2.774906	-1.319544
H	2.450821	1.069970	-5.263518
H	-5.805354	-1.783790	-2.699940
H	-2.872006	-4.572270	-1.281974
H	3.936223	-0.721461	-2.925647
H	1.129869	-2.556349	-0.204967
H	-3.080536	3.464184	-5.146819
H	0.644599	-2.786654	-5.775017
H	1.699506	-4.803403	-1.150540
H	4.522247	-2.974927	-3.836557
H	-3.826422	4.100371	-0.971769
H	4.955378	1.709481	3.863929
H	6.349243	-2.229815	2.731584
H	7.019085	0.007246	-0.608198
H	7.423799	1.411307	2.194566
H	4.115906	2.071954	-0.517762
H	4.211227	-2.747246	1.207457
H	5.832228	1.333625	6.174785
H	7.863300	-1.842159	-2.059459
H	5.086767	-4.575540	-0.271481
H	3.251923	-0.786967	6.255748
H	2.049596	-2.564128	2.509340
H	8.549273	3.448707	1.290867
H	7.177075	-2.585023	5.059181
H	2.736523	-4.813530	3.368118
H	3.916683	-3.037463	7.108035
H	5.275342	4.112770	-1.397685
Bq	0.816095	0.118298	1.442276
Li	0.801142	2.243215	1.463779

Energies

Zero-point correction= 1.003653 (Hartree/Particle)
Thermal correction to Energy= 1.094425
Thermal correction to Enthalpy= 1.095369
Thermal correction to Gibbs Free Energy= 0.841613
Sum of electronic and zero-point Energies= -7628.593081
Sum of electronic and thermal Energies= -7628.502309
Sum of electronic and thermal Enthalpies= -7628.501364
Sum of electronic and thermal Free Energies= -7628.755121

	E (Thermal)	CV	S
	KCal/Mol	Cal/Mol-Kelvin	Cal/Mol-Kelvin
Total	686.762	340.375	534.075

# - Pt_3 cation – Li+ adduct with $BF4^-$ (M06/Def2-svp)

147
scf done: -8053.889607

C	4.717604	-2.625640	0.515022
C	5.469246	-1.443849	0.443454
C	6.643382	-1.417391	-0.321281
C	7.063073	-2.557713	-0.996651
C	6.302859	-3.719547	-0.901397
C	5.133445	-3.771330	-0.150934

P	4.917481	-0.002161	1.400441
C	5.608717	-0.225752	3.073347
C	5.562578	0.843097	3.980747
C	6.016021	0.685038	5.282774
C	6.495471	-0.560486	5.679604
C	6.556291	-1.634046	4.802572
C	6.113113	-1.460838	3.494807
F	6.886320	-0.726065	6.932059
F	6.703189	-4.798524	-1.548576
Pt	2.569880	0.156546	1.415365
S	2.160879	0.256859	-0.916635
C	2.578809	-1.318095	-1.647062
C	1.959694	-2.506822	-1.259724
C	2.286762	-3.698423	-1.894479
C	3.247454	-3.691406	-2.905170
C	3.890133	-2.510596	-3.282736
C	3.551817	-1.319438	-2.651396
Cl	3.671378	-5.174311	-3.692524
Pt	-0.022613	0.117850	-0.010453
P	-1.219756	-0.046347	-2.026922
C	-2.364419	-1.457401	-1.998345
C	-3.627476	-1.388472	-2.601104
C	-4.461698	-2.500870	-2.604526
C	-4.022436	-3.676626	-2.004357
C	-2.774895	-3.769570	-1.396495
C	-1.951277	-2.651329	-1.388439
F	-4.820730	-4.728285	-2.000584
Pt	0.029214	0.120074	2.947215
P	-1.108170	-0.054482	5.003438
C	-0.503787	-1.467308	5.973542
C	-0.389773	-1.417605	7.369436
C	0.039691	-2.536370	8.075736
C	0.349433	-3.699053	7.376791
C	0.233839	-3.776466	5.993378
C	-0.184519	-2.652001	5.294419
F	0.782866	-4.752913	8.044278
S	-1.847670	0.192775	1.502968
C	-2.658606	-1.397684	1.544758
C	-1.976769	-2.569548	1.872450
C	-2.664225	-3.774219	1.950939
C	-4.029468	-3.798923	1.669165
C	-4.715618	-2.634903	1.316448
C	-4.027027	-1.428717	1.260186
Cl	-4.884361	-5.304051	1.711224
S	2.253236	0.253730	3.752852
C	2.714471	-1.317614	4.462089
C	2.683524	-2.507631	3.734009
C	3.074629	-3.698376	4.333040
C	3.466246	-3.689928	5.671276
C	3.502240	-2.504106	6.407647
C	3.127615	-1.313045	5.797886
Cl	3.857711	-5.181862	6.458188
C	-2.239364	1.405361	-2.454421
C	-2.458703	1.764492	-3.792221
C	-3.315159	2.811262	-4.113866
C	-3.957730	3.494871	-3.087957
C	-3.766345	3.155298	-1.753408
C	-2.905071	2.109908	-1.441984
F	-4.769152	4.492038	-3.386848
C	-0.130384	-0.306970	-3.465721

C	0.682946	0.746788	-3.909802
C	1.582119	0.555744	-4.949595
C	1.683376	-0.707982	-5.525698
C	0.892431	-1.767895	-5.106751
C	-0.019340	-1.561005	-4.075815
F	2.570083	-0.903554	-6.486504
C	-0.970637	1.397722	6.102073
C	-2.028531	1.779365	6.939822
C	-1.880408	2.835749	7.831986
C	-0.663313	3.504687	7.887663
C	0.406830	3.140865	7.077691
C	0.246601	2.087963	6.184945
F	-0.518304	4.511161	8.729498
C	-2.899067	-0.330112	4.792578
C	-3.700977	0.707169	4.292111
C	-5.052507	0.502683	4.050619
C	-5.593316	-0.758293	4.287009
C	-4.825086	-1.801679	4.782600
C	-3.475012	-1.581226	5.038518
F	-6.870238	-0.968655	4.016041
C	5.810308	1.431048	0.709621
C	7.078777	1.788007	1.190361
C	7.792630	2.826299	0.602704
C	7.231886	3.504111	-0.473344
C	5.979892	3.167422	-0.975777
C	5.272589	2.130041	-0.379123
F	7.903329	4.492834	-1.033801
Li	0.809808	2.261230	1.451214
H	-3.264937	1.692538	4.089496
H	-2.866395	-2.404748	5.424805
H	-0.632166	-0.501902	7.918382
H	-2.982052	1.243002	6.904390
H	1.087834	1.794402	5.547502
H	-0.247618	-2.716925	4.202681
H	-5.699677	1.296663	3.669651
H	0.144300	-2.524779	9.163364
H	0.494016	-4.702103	5.471892
H	-4.555990	-0.507939	0.990101
H	-0.894608	-2.575100	2.032680
H	-2.693017	3.149820	8.491658
H	-5.289468	-2.777194	4.948899
H	-2.125742	-4.692783	2.199839
H	-5.785159	-2.682042	1.093850
H	1.347184	3.691443	7.159592
H	0.609411	1.735279	-3.441336
H	-0.644950	-2.395725	-3.744535
H	-3.970726	-0.461923	-3.072577
H	-1.961775	1.216116	-4.598919
H	-2.760005	1.829041	-0.393070
H	-0.978247	-2.734539	-0.890721
H	2.219586	1.362651	-5.319652
H	-5.452029	-2.476286	-3.065759
H	-2.464197	-4.701647	-0.915872
H	4.044961	-0.385549	-2.943560
H	1.240318	-2.535339	-0.435424
H	-3.498882	3.106689	-5.149754
H	1.007752	-2.744573	-5.584050
H	1.809527	-4.631254	-1.583062
H	4.645521	-2.533223	-4.072700
H	-4.297913	3.716368	-0.980908

H	5.170118	1.816724	3.664264
H	6.154894	-2.306625	2.801087
H	7.241041	-0.503263	-0.397165
H	7.522484	1.245604	2.031700
H	4.288773	1.853637	-0.773532
H	3.789805	-2.681627	1.095022
H	5.998348	1.504356	6.005743
H	7.973681	-2.566082	-1.600812
H	4.540726	-4.689481	-0.103813
H	3.152720	-0.375271	6.364400
H	2.313687	-2.538994	2.704833
H	8.781900	3.120562	0.961703
H	6.938067	-2.594914	5.157473
H	3.031376	-4.630158	3.762075
H	3.817038	-2.525169	7.454588
H	5.581022	3.725399	-1.826321
B	0.893668	-4.680324	1.451813
F	0.811691	-6.038318	1.473423
F	2.219146	-4.248288	1.240582
F	0.089301	-4.143287	0.420566
F	0.450259	-4.131733	2.677053

Energies

Zero-point correction= 1.023390 (Hartree/Particle)
Thermal correction to Energy= 1.117162
Thermal correction to Enthalpy= 1.118106
Thermal correction to Gibbs Free Energy= 0.885534
Sum of electronic and zero-point Energies= -8052.866216
Sum of electronic and thermal Energies= -8052.772445
Sum of electronic and thermal Enthalpies= -8052.771501
Sum of electronic and thermal Free Energies= -8053.004073

	E (Thermal)	CV	S
	KCal/Mol	Cal/Mol-Kelvin	Cal/Mol-Kelvin
Total	701.030	354.602	489.489

# - Pd_2Pt cation – Li+ adduct (M06/Def2-svp)

143
scf done: -7646.658408

Pd	-0.016161	0.080597	0.016977
Pd	-0.000142	0.041221	2.952030
Pt	2.554933	0.040593	1.468819
S	-1.846376	0.178299	1.497070
S	2.208421	0.035141	3.805291
S	2.170888	0.108967	-0.867645
P	-1.193920	0.015990	-2.052230
P	-1.195184	-0.041164	5.015446
P	4.909298	-0.016961	1.449812
C	-2.585419	-1.442122	1.554991
C	2.592043	-1.621441	4.348658
C	2.429535	-1.539973	-1.505546
C	1.806473	-2.673220	-0.966857
C	2.048769	-3.927105	-1.512708
C	2.923813	-4.049341	-2.596253
C	3.526731	-2.923703	-3.156472
C	3.275036	-1.670163	-2.610277

C	2.459688	-2.743113	3.519492
C	2.799488	-4.004059	3.991080
C	3.260718	-4.147879	5.302581
C	3.405224	-3.036145	6.132519
C	3.076758	-1.773431	5.651003
C	-1.863725	-2.597265	1.884441
C	-2.505839	-3.826452	1.957881
C	-3.873820	-3.906040	1.682038
C	-4.600406	-2.763511	1.347421
C	-3.954954	-1.533335	1.288999
Cl	3.298292	-5.616675	-3.214107
C	5.637846	-0.415172	3.071097
C	6.299985	-1.624293	3.313564
C	6.795446	-1.917177	4.581098
C	6.627926	-0.988147	5.598755
C	5.993155	0.233334	5.380162
C	5.491438	0.509847	4.116962
F	7.077639	-1.264245	6.806562
C	5.684331	1.543734	0.930116
C	6.923091	1.935590	1.460291
C	7.558137	3.081580	0.997883
C	6.950351	3.832501	-0.002688
C	5.727574	3.460710	-0.554668
C	5.098164	2.315332	-0.084052
F	7.544593	4.921151	-0.440616
C	5.497099	-1.291049	0.294109
C	6.465409	-1.014428	-0.679095
C	6.886192	-2.010025	-1.556281
C	6.331451	-3.279178	-1.449478
C	5.380522	-3.586104	-0.479904
C	4.957289	-2.584572	0.381570
F	6.692544	-4.216367	-2.303053
Cl	3.611141	-5.723375	5.912583
C	-2.974378	-0.393323	4.819196
C	-3.526779	-1.642546	5.125691
C	-4.866975	-1.909449	4.862326
C	-5.653477	-0.912364	4.302035
C	-5.138664	0.348506	4.010838
C	-3.795721	0.597133	4.256595
F	-6.917338	-1.167799	4.024270
C	-1.101524	1.499678	5.980536
C	-2.132573	1.869445	6.857792
C	-2.011972	3.004445	7.650236
C	-0.850709	3.766390	7.566434
C	0.191617	3.417851	6.713179
C	0.059146	2.284090	5.920671
F	-0.732889	4.843182	8.313591
C	-0.521492	-1.346207	6.094518
C	-0.141138	-1.083385	7.416730
C	0.404089	-2.091086	8.207371
C	0.560389	-3.360338	7.666053
C	0.171798	-3.655920	6.362451
C	-0.353189	-2.640581	5.575621
F	1.120253	-4.308328	8.392396
Cl	-4.661520	-5.440860	1.721690
C	-0.139053	-0.366291	-3.490429
C	-0.191205	-1.597282	-4.154933
C	0.678821	-1.871692	-5.205951
C	1.593189	-0.901825	-5.593835
C	1.653449	0.339995	-4.965525

C	0.794158	0.596811	-3.906923
F	2.435689	-1.163723	-6.574319
C	-2.045293	1.567498	-2.477987
C	-2.307727	1.894816	-3.817494
C	-3.028493	3.038456	-4.136621
C	-3.488809	3.854791	-3.107670
C	-3.249545	3.551084	-1.771200
C	-2.525794	2.405212	-1.462174
F	-4.169541	4.940365	-3.405458
C	-2.481019	-1.269146	-2.004769
C	-3.808726	-0.995978	-2.357447
C	-4.774510	-1.995699	-2.294831
C	-4.401487	-3.266847	-1.876954
C	-3.087044	-3.569909	-1.530929
C	-2.133371	-2.562970	-1.584142
F	-5.319670	-4.207958	-1.779069
H	6.441059	-2.352751	2.509922
H	7.314539	-2.855157	4.793170
H	5.905601	0.943269	6.206416
H	4.985338	1.466104	3.940685
H	7.405516	1.338403	2.240639
H	8.522440	3.406422	1.396283
H	5.294880	4.077002	-1.346408
H	4.147132	2.003499	-0.530080
H	6.902235	-0.014038	-0.762426
H	7.638124	-1.819392	-2.325939
H	4.975987	-4.601125	-0.440408
H	4.200309	-2.815247	1.141561
H	3.743300	-0.781228	-3.045448
H	4.200990	-3.038883	-4.010335
H	1.577250	-4.823034	-1.099687
H	1.126814	-2.568517	-0.111876
H	-2.914043	-2.426706	5.579920
H	-5.313872	-2.881918	5.084198
H	-5.800309	1.109027	3.588636
H	-3.384569	1.582944	4.009994
H	-3.040968	1.262851	6.931066
H	-2.802095	3.311905	8.339533
H	1.087953	4.042402	6.689253
H	0.883148	1.991638	5.260023
H	-0.265707	-0.082893	7.842753
H	0.712719	-1.908823	9.239819
H	0.315715	-4.671994	5.984406
H	-0.639185	-2.861750	4.539672
H	3.188667	-0.897346	6.298475
H	3.766653	-3.170223	7.156062
H	2.700511	-4.888746	3.356344
H	2.084009	-2.622976	2.496029
H	-0.920913	-2.357623	-3.861105
H	0.654829	-2.826250	-5.737516
H	2.374729	1.080963	-5.319445
H	0.842442	1.570654	-3.405862
H	-1.953142	1.246215	-4.625255
H	-3.245883	3.311772	-5.172085
H	-3.638820	4.218555	-0.998558
H	-2.350077	2.147798	-0.411568
H	-4.100210	0.006627	-2.686419
H	-5.816736	-1.806474	-2.563703
H	-2.845271	-4.585543	-1.204815
H	-1.097547	-2.787869	-1.298392

H	-4.521584	-0.633479	1.027586
H	-5.669785	-2.846877	1.132444
H	-1.955809	-4.734885	2.218086
H	-0.787396	-2.523685	2.085367
Bq	0.843364	0.064091	1.473772
Li	0.921776	2.189116	1.516608

Energies

Zero-point correction= 1.003526 (Hartree/Particle)
Thermal correction to Energy= 1.093570
Thermal correction to Enthalpy= 1.094515
Thermal correction to Gibbs Free Energy= 0.855392
Sum of electronic and zero-point Energies= -7645.654882
Sum of electronic and thermal Energies= -7645.564838
Sum of electronic and thermal Enthalpies= -7645.563894
Sum of electronic and thermal Free Energies= -7645.803016

	E (Thermal)	CV	S
	KCal/Mol	Cal/Mol-Kelvin	Cal/Mol-Kelvin
Total	686.226	338.550	503.275

# - Pd_2Pt cation – Li+ adduct with $BF4^-$ (M06/Def2-svp)

147
scf done: -8063.587916

C	4.711704	-2.629562	0.540827
C	5.467879	-1.452337	0.447422
C	6.615595	-1.439000	-0.355310
C	6.999080	-2.580800	-1.052232
C	6.229452	-3.733724	-0.939326
C	5.092846	-3.778181	-0.141557
P	4.908748	-0.002782	1.404741
C	5.635243	-0.244501	3.068773
C	5.626711	0.830720	3.969138
C	6.099520	0.673775	5.264999
C	6.556772	-0.577840	5.665197
C	6.570906	-1.661113	4.799803
C	6.110372	-1.487804	3.497013
F	6.974750	-0.740165	6.914534
F	6.585108	-4.810752	-1.623926
Pd	2.562207	0.228040	1.409254
S	2.154569	0.316912	-0.908318
C	2.555708	-1.271777	-1.613206
C	1.939897	-2.454893	-1.197943
C	2.254207	-3.663012	-1.807453
C	3.215218	-3.682993	-2.815902
C	3.853061	-2.513844	-3.230966
C	3.517858	-1.306428	-2.628404
Cl	3.669659	-5.197059	-3.536839
Pt	-0.017214	0.194323	-0.022797
P	-1.195703	-0.044167	-2.001889
C	-2.306261	-1.489793	-1.971626
C	-3.581930	-1.475706	-2.549668
C	-4.371585	-2.621411	-2.532641
C	-3.875996	-3.776867	-1.938039
C	-2.615148	-3.818010	-1.355414
C	-1.840142	-2.665752	-1.366096
F	-4.634861	-4.862105	-1.919793

Pd	0.025717	0.212851	2.928225
P	-1.119927	-0.042607	4.978657
C	-0.541573	-1.486167	5.935483
C	-0.416328	-1.472947	7.330326
C	0.001474	-2.613495	8.009627
C	0.291438	-3.764166	7.284506
C	0.173357	-3.806127	5.901071
C	-0.234134	-2.659088	5.231478
F	0.704995	-4.843363	7.932148
S	-1.824522	0.283484	1.480157
C	-2.611281	-1.317613	1.543875
C	-1.915851	-2.483151	1.874384
C	-2.587880	-3.695517	1.978352
C	-3.955127	-3.737525	1.715219
C	-4.657822	-2.588917	1.351790
C	-3.982192	-1.375965	1.275272
Cl	-4.799981	-5.253523	1.810227
S	2.227026	0.370262	3.718491
C	2.670069	-1.206265	4.416791
C	2.591444	-2.395780	3.686542
C	2.969612	-3.602322	4.261787
C	3.399212	-3.614917	5.587080
C	3.475980	-2.439522	6.334149
C	3.112929	-1.234147	5.743629
Cl	3.791706	-5.128020	6.347008
C	-2.248029	1.372512	-2.481798
C	-2.578261	1.597794	-3.826526
C	-3.421896	2.642444	-4.184915
C	-3.934029	3.463828	-3.187018
C	-3.626922	3.264352	-1.847734
C	-2.780728	2.216346	-1.499934
F	-4.733131	4.465188	-3.524551
C	-0.122802	-0.304391	-3.460116
C	0.664414	0.762722	-3.917351
C	1.548427	0.588218	-4.973239
C	1.658970	-0.671511	-5.554458
C	0.897463	-1.745727	-5.120694
C	0.000572	-1.555061	-4.072374
F	2.527226	-0.848915	-6.541353
C	-1.012598	1.368673	6.140978
C	-2.002341	1.598532	7.107708
C	-1.872300	2.635981	8.023858
C	-0.742514	3.444679	7.966713
C	0.253642	3.241651	7.021016
C	0.111462	2.202135	6.107104
F	-0.617450	4.437571	8.835513
C	-2.922396	-0.314470	4.795079
C	-3.717882	0.741188	4.325075
C	-5.074182	0.557636	4.089414
C	-5.626755	-0.701673	4.299532
C	-4.865861	-1.764905	4.760595
C	-3.510879	-1.564365	5.011735
F	-6.913416	-0.892403	4.035378
C	5.851306	1.412425	0.726591
C	7.181837	1.651642	1.101277
C	7.899571	2.700960	0.539336
C	7.276841	3.512974	-0.401737
C	5.961735	3.301136	-0.793084
C	5.251756	2.249003	-0.222907
F	7.956958	4.517054	-0.936677

H	-3.270111	1.724468	4.138668
H	-2.907651	-2.404843	5.368865
H	-0.639732	-0.566299	7.901384
H	-2.887672	0.954890	7.149813
H	0.889587	2.034815	5.354463
H	-0.298140	-2.695509	4.138736
H	-5.713019	1.367470	3.728048
H	0.113479	-2.626879	9.096643
H	0.428254	-4.714370	5.347820
H	-4.523592	-0.462620	1.004149
H	-0.831252	-2.468195	2.028908
H	-2.631819	2.831617	8.784970
H	-5.336012	-2.742558	4.897316
H	-2.032149	-4.598577	2.243207
H	-5.729859	-2.649934	1.144548
H	1.122142	3.904734	7.008651
H	0.589225	1.745218	-3.437148
H	-0.602637	-2.400887	-3.727887
H	-3.974463	-0.565644	-3.014301
H	-2.172765	0.946248	-4.607878
H	-2.530691	2.051404	-0.446852
H	-0.857783	-2.707547	-0.885226
H	2.168967	1.405861	-5.348583
H	-5.372473	-2.636757	-2.971424
H	-2.246108	-4.729497	-0.877001
H	4.008768	-0.379683	-2.946895
H	1.232411	-2.461915	-0.362275
H	-3.689861	2.836169	-5.226542
H	1.023992	-2.721306	-5.597262
H	1.767481	-4.581066	-1.466691
H	4.610828	-2.559813	-4.017989
H	-4.048056	3.937933	-1.097391
H	5.242675	1.807564	3.652294
H	6.115042	-2.342300	2.812594
H	7.217758	-0.530067	-0.451426
H	7.668697	1.007015	1.840961
H	4.211919	2.075545	-0.520668
H	3.804018	-2.675423	1.152071
H	6.107723	1.499398	5.981033
H	7.884655	-2.592837	-1.692776
H	4.492137	-4.689844	-0.076081
H	3.171837	-0.302204	6.317242
H	2.202579	-2.406969	2.662810
H	8.937034	2.904511	0.816110
H	6.926914	-2.630457	5.158661
H	2.886180	-4.522776	3.678110
H	3.815523	-2.477161	7.373001
H	5.510319	3.968004	-1.531605
B	0.916755	-4.807474	1.434388
F	0.998052	-6.170498	1.384704
F	2.200011	-4.231256	1.320069
F	0.118817	-4.317065	0.377189
F	0.351080	-4.380153	2.653276

Energies

Zero-point correction= 1.023192 (Hartree/Particle)
Thermal correction to Energy= 1.116917
Thermal correction to Enthalpy= 1.117861
Thermal correction to Gibbs Free Energy= 0.885992

Sum of electronic and zero-point Energies= -8069.928463
Sum of electronic and thermal Energies= -8069.834739
Sum of electronic and thermal Enthalpies= -8069.833795
Sum of electronic and thermal Free Energies= -8070.065664

	E (Thermal)	CV	S
	KCal/Mol	Cal/Mol-Kelvin	Cal/Mol-Kelvin
Total	700.876	354.681	488.009

# - $PdPt_2$ cation – Li+ adduct (M06/Def2-svp)

143
scf done: -7638.127820

Pt	-0.023515	0.026057	-0.030938
Pt	-0.010343	0.042062	2.928172
Pd	2.542687	0.059175	1.435263
S	-1.872353	0.064489	1.463238
S	2.198088	0.106464	3.766021
S	2.171570	0.091053	-0.895629
P	-1.188926	-0.033674	-2.080172
P	-1.148867	-0.004966	4.990459
P	4.928008	0.016018	1.462003
C	-2.587304	-1.572815	1.518804
C	2.625229	-1.542638	4.303956
C	2.516411	-1.550769	-1.504021
C	3.464496	-1.665263	-2.525483
C	3.794237	-2.915394	-3.038146
C	3.163779	-4.052387	-2.533031
C	2.194816	-3.946368	-1.531257
C	1.879004	-2.697588	-1.013014
C	3.165078	-1.679869	5.584927
C	3.545689	-2.932252	6.054275
C	3.388554	-4.050152	5.235222
C	2.862300	-3.922144	3.946355
C	2.477875	-2.669847	3.485394
C	-3.955536	-1.672821	1.253996
C	-4.584481	-2.913086	1.280906
C	-3.841443	-4.053392	1.585168
C	-2.476727	-3.960448	1.871986
C	-1.850053	-2.721473	1.829711
Cl	3.616137	-5.613821	-3.112208
C	5.717658	1.554359	0.889706
C	6.972893	1.943858	1.382088
C	7.611788	3.069419	0.877389
C	6.990717	3.803924	-0.127656
C	5.750433	3.436616	-0.640493
C	5.117209	2.311272	-0.126296
F	7.589931	4.873116	-0.606621
C	5.626039	-0.288023	3.120451
C	5.480623	0.712263	4.094929
C	5.963321	0.524404	5.382081
C	6.575062	-0.686249	5.699622
C	6.729096	-1.694607	4.757854
C	6.255535	-1.489434	3.464957
F	7.009845	-0.879534	6.929969
C	5.582812	-1.301703	0.387984
C	5.038300	-2.591984	0.487376
C	5.514601	-3.624690	-0.308360
C	6.531718	-3.354340	-1.219810

C	7.090563	-2.088211	-1.339143
C	6.611499	-1.060828	-0.531712
F	6.955670	-4.322930	-2.007147
Cl	3.814216	-5.613201	5.829106
C	-1.045241	1.550249	5.929262
C	-2.083272	1.931503	6.793417
C	-1.968064	3.075359	7.573765
C	-0.805449	3.834930	7.490566
C	0.243289	3.475080	6.649626
C	0.117389	2.332258	5.869773
F	-0.692442	4.920242	8.225443
C	-2.927916	-0.361143	4.826687
C	-3.738298	0.586265	4.180905
C	-5.089901	0.344401	3.984688
C	-5.627131	-0.864047	4.423349
C	-4.852647	-1.812620	5.077966
C	-3.500163	-1.554891	5.281908
F	-6.903972	-1.109824	4.208211
C	-0.450127	-1.299315	6.061070
C	-0.297852	-2.596032	5.543794
C	0.259702	-3.604253	6.316693
C	0.692368	-3.298980	7.604345
C	0.546079	-2.028132	8.145497
C	-0.028809	-1.026476	7.368256
F	1.281489	-4.240143	8.315388
Cl	-4.601157	-5.602275	1.560852
C	-2.037050	1.525599	-2.481475
C	-2.179971	1.946214	-3.811861
C	-2.909208	3.089561	-4.116651
C	-3.502623	3.806674	-3.082965
C	-3.387345	3.405000	-1.755208
C	-2.651867	2.264103	-1.459670
F	-4.193256	4.889974	-3.365256
C	-0.127591	-0.412755	-3.511857
C	0.833114	0.532352	-3.906552
C	1.704444	0.262794	-4.951364
C	1.626933	-0.973273	-5.589831
C	0.677819	-1.920820	-5.231250
C	-0.203031	-1.633901	-4.192486
F	2.478117	-1.245275	-6.558835
C	-2.480067	-1.313274	-2.047999
C	-2.142409	-2.608443	-1.623710
C	-3.097939	-3.613958	-1.595617
C	-4.403881	-3.308509	-1.970857
C	-4.765406	-2.037441	-2.399146
C	-3.796672	-1.039009	-2.438840
F	-5.324520	-4.248583	-1.890284
H	7.466434	1.358982	2.165174
H	8.589229	3.391029	1.245415
H	5.305518	4.040833	-1.434735
H	4.147350	2.006912	-0.535983
H	4.992304	1.660827	3.842493
H	5.878004	1.296247	6.151146
H	7.227655	-2.623602	5.046066
H	6.392538	-2.278876	2.719671
H	4.233318	-2.794359	1.204680
H	5.104624	-4.637471	-0.260788
H	7.891559	-1.924949	-2.064337
H	7.051061	-0.062804	-0.627057
H	1.133013	-2.606321	-0.213905

H	1.710228	-4.851489	-1.155502
H	4.549819	-3.020930	-3.822127
H	3.957137	-0.768356	-2.915394
H	-2.993943	1.328482	6.865709
H	-2.763804	3.391707	8.252454
H	1.140352	4.098581	6.625235
H	0.947664	2.032662	5.221172
H	-3.311212	1.534303	3.833619
H	-5.744258	1.071827	3.497763
H	-5.319550	-2.738253	5.423351
H	-2.893692	-2.296323	5.810372
H	-0.621656	-2.823244	4.520168
H	0.394931	-4.621845	5.939172
H	0.888529	-1.839656	9.166091
H	-0.143130	-0.023556	7.791780
H	2.057327	-2.558431	2.478118
H	2.753534	-4.811324	3.319421
H	3.958232	-3.053134	7.060216
H	3.281633	-0.799939	6.226004
H	-1.722625	1.373841	-4.625111
H	-3.032378	3.436166	-5.145576
H	-3.881745	3.994071	-0.978966
H	-2.576480	1.930264	-0.418851
H	0.896802	1.500977	-3.397779
H	2.449234	0.988876	-5.286714
H	0.638348	-2.869061	-5.772768
H	-0.958642	-2.376937	-3.921638
H	-1.113855	-2.834646	-1.314533
H	-2.866160	-4.631255	-1.268654
H	-5.800341	-1.847219	-2.694013
H	-4.078106	-0.037093	-2.778769
H	-0.775150	-2.641634	2.033557
H	-1.915423	-4.867222	2.112766
H	-5.650699	-3.007505	1.056803
H	-4.532578	-0.775290	1.008166
Bq	0.830051	0.026415	1.433258
Li	0.747101	2.189040	1.425948

Energies

Zero-point correction= 1.003587 (Hartree/Particle)
Thermal correction to Energy= 1.093414
Thermal correction to Enthalpy= 1.094359
Thermal correction to Gibbs Free Energy= 0.857010
Sum of electronic and zero-point Energies= -7637.124232
Sum of electronic and thermal Energies= -7637.034405
Sum of electronic and thermal Enthalpies= -7637.033461
Sum of electronic and thermal Free Energies= -7637.270809

	E (Thermal)	CV	S
	KCal/Mol	Cal/Mol-Kelvin	Cal/Mol-Kelvin
Total	686.128	338.438	499.541

# - $PdPt_2$ cation – Li+ adduct with $BF4^-$ (M06/Def2-svp)

147
scf done: -8062.420732

C	4.716000	-2.623203	0.512280
C	5.468268	-1.441759	0.441542

C	6.643297	-1.415467	-0.322037
C	7.062539	-2.555556	-0.998016
C	6.301290	-3.716904	-0.904225
C	5.131801	-3.768679	-0.153943
P	4.920361	0.000142	1.399726
C	5.612159	-0.223845	3.071732
C	5.570361	0.845131	3.979306
C	6.029345	0.686887	5.279072
C	6.508677	-0.559434	5.674449
C	6.562155	-1.633844	4.798244
C	6.114449	-1.460096	3.491899
F	6.906127	-0.724288	6.924660
F	6.700260	-4.795294	-1.553037
Pt	2.573628	0.154409	1.410321
S	2.170615	0.242825	-0.926393
C	2.584910	-1.333201	-1.655220
C	1.962220	-2.519201	-1.265616
C	2.285957	-3.713082	-1.897790
C	3.247059	-3.710940	-2.908149
C	3.892398	-2.532478	-3.288819
C	3.557423	-1.339080	-2.660065
Cl	3.668403	-5.196762	-3.691134
Pt	-0.016148	0.117196	-0.017195
P	-1.213838	-0.040037	-2.032883
C	-2.361608	-1.447487	-1.997398
C	-3.627427	-1.374073	-2.593979
C	-4.465474	-2.483501	-2.592630
C	-4.027780	-3.660193	-1.992996
C	-2.777664	-3.757275	-1.391072
C	-1.949689	-2.642295	-1.388239
F	-4.829866	-4.708748	-1.984134
Pd	0.034444	0.113376	2.930440
P	-1.119711	-0.063588	5.014107
C	-0.511296	-1.476140	5.985114
C	-0.393150	-1.430552	7.380799
C	0.039971	-2.550647	8.082742
C	0.350515	-3.710616	7.379700
C	0.233720	-3.783431	5.996216
C	-0.188543	-2.657302	5.301806
F	0.784593	-4.766610	8.044071
S	-1.834727	0.213861	1.488640
C	-2.651634	-1.371136	1.539784
C	-1.970821	-2.543909	1.869299
C	-2.659575	-3.747407	1.954450
C	-4.025831	-3.770943	1.677738
C	-4.711714	-2.607057	1.323999
C	-4.021933	-1.402088	1.261972
Cl	-4.883296	-5.274273	1.729867
S	2.248659	0.266789	3.738412
C	2.708403	-1.298019	4.454588
C	2.680368	-2.491296	3.730121
C	3.072970	-3.680079	4.331200
C	3.462350	-3.668607	5.670161
C	3.492739	-2.481335	6.404849
C	3.116882	-1.292153	5.792737
Cl	3.859542	-5.157752	6.458928
C	-2.227681	1.414443	-2.461961
C	-2.437872	1.776926	-3.800442
C	-3.292453	2.824280	-4.124979
C	-3.942172	3.504964	-3.101606

C	-3.760081	3.161619	-1.766658
C	-2.900631	2.115806	-1.452048
F	-4.751567	4.502719	-3.403334
C	-0.123969	-0.308963	-3.469099
C	0.694192	0.739955	-3.916050
C	1.592102	0.542050	-4.955533
C	1.687480	-0.723707	-5.528296
C	0.891656	-1.778880	-5.106503
C	-0.018884	-1.565209	-4.075995
F	2.573159	-0.925877	-6.488473
C	-0.990167	1.384436	6.122215
C	-2.044809	1.762809	6.965629
C	-1.896017	2.820584	7.856113
C	-0.681540	3.494931	7.904678
C	0.385543	3.135105	7.089224
C	0.223892	2.081137	6.197606
F	-0.536301	4.502788	8.745377
C	-2.912140	-0.347446	4.812747
C	-3.719284	0.688931	4.318396
C	-5.070390	0.480850	4.076939
C	-5.605309	-0.784425	4.302665
C	-4.831542	-1.828478	4.787900
C	-3.482500	-1.603625	5.046018
F	-6.881828	-0.998493	4.030532
C	5.811458	1.431622	0.703803
C	7.068023	1.808746	1.199729
C	7.782661	2.843014	0.605715
C	7.235253	3.495443	-0.492454
C	5.995655	3.137199	-1.011114
C	5.287316	2.104487	-0.408071
F	7.907191	4.479993	-1.059263
Li	0.838232	2.246914	1.400537
H	-3.288888	1.678751	4.125262
H	-2.870068	-2.427914	5.424823
H	-0.637734	-0.517390	7.933049
H	-2.996120	1.222116	6.936304
H	1.062601	1.790843	5.555015
H	-0.254759	-2.719104	4.209984
H	-5.721213	1.275248	3.703086
H	0.145493	-2.542802	9.170344
H	0.495188	-4.706633	5.471124
H	-4.550966	-0.481415	0.992025
H	-0.888277	-2.550510	2.029021
H	-2.706071	3.131523	8.520444
H	-5.290409	-2.808548	4.943132
H	-2.122756	-4.666087	2.206285
H	-5.782217	-2.653524	1.105788
H	1.324059	3.689660	7.165317
H	0.625190	1.729999	-3.450233
H	-0.648703	-2.395906	-3.742634
H	-3.969440	-0.446645	-3.064656
H	-1.936041	1.230582	-4.605410
H	-2.763234	1.831134	-0.403005
H	-0.974427	-2.728987	-0.895405
H	2.233149	1.345020	-5.327944
H	-5.457852	-2.455752	-3.049238
H	-2.468248	-4.690042	-0.910975
H	4.052919	-0.407166	-2.954516
H	1.242833	-2.543080	-0.441208
H	-3.469350	3.122318	-5.161294

H	1.002377	-2.757250	-5.581397
H	1.806389	-4.643870	-1.583868
H	4.647609	-2.558937	-4.078845
H	-4.297243	3.720212	-0.996241
H	5.176384	1.818773	3.665013
H	6.151617	-2.306424	2.798587
H	7.242125	-0.501905	-0.395803
H	7.502368	1.285197	2.057588
H	4.314194	1.809966	-0.816007
H	3.787807	-2.679440	1.091550
H	6.015698	1.506398	6.001880
H	7.973611	-2.564182	-1.601476
H	4.539133	-4.686857	-0.106958
H	3.140701	-0.353335	6.357569
H	2.312970	-2.525302	2.700063
H	8.762664	3.153062	0.976699
H	6.942272	-2.595721	5.152150
H	3.031971	-4.612836	3.761614
H	3.804948	-2.500128	7.452596
H	5.607526	3.674960	-1.879491
B	0.893455	-4.671634	1.452678
F	0.813320	-6.029569	1.478568
F	2.218990	-4.239144	1.240023
F	0.089087	-4.138704	0.419297
F	0.450141	-4.118859	2.675528

Energies

Zero-point correction= 1.023092 (Hartree/Particle)
Thermal correction to Energy= 1.116896
Thermal correction to Enthalpy= 1.117840
Thermal correction to Gibbs Free Energy= 0.885364
Sum of electronic and zero-point Energies= -8061.397640
Sum of electronic and thermal Energies= -8061.303836
Sum of electronic and thermal Enthalpies= -8061.302892
Sum of electronic and thermal Free Energies= -8061.535368

	E (Thermal)	CV	S
	KCal/Mol	Cal/Mol-Kelvin	Cal/Mol-Kelvin
Total	700.863	354.727	489.287

# - Pt_3 cation – BF_4^- (M06/Def2-svp)

146
scf done: -8046.519423

C	4.702496	-2.628783	0.532665
C	5.454495	-1.448524	0.441793
C	6.605575	-1.430839	-0.355966
C	6.995207	-2.570534	-1.052917
C	6.228994	-3.726095	-0.943775
C	5.090349	-3.775360	-0.149144
P	4.891967	0.000271	1.396923
C	5.614246	-0.232420	3.062974
C	5.604483	0.845965	3.959305
C	6.074917	0.692422	5.256560
C	6.535324	-0.557025	5.659783
C	6.554515	-1.642192	4.796525
C	6.094279	-1.473226	3.493334

F	6.952694	-0.715370	6.909527
F	6.589186	-4.800816	-1.629236
Pt	2.579006	0.220161	1.418141
S	2.157931	0.310866	-0.901843
C	2.550228	-1.282923	-1.610569
C	1.934472	-2.464002	-1.192329
C	2.246895	-3.672302	-1.803037
C	3.203927	-3.692320	-2.815204
C	3.840047	-2.523569	-3.233398
C	3.507387	-1.316003	-2.629129
Cl	3.657292	-5.207022	-3.536039
Pt	-0.016935	0.189468	-0.009246
P	-1.201938	-0.052344	-1.993840
C	-2.304502	-1.506191	-1.979540
C	-3.570090	-1.503407	-2.579377
C	-4.351070	-2.655183	-2.575652
C	-3.856372	-3.806693	-1.972904
C	-2.606186	-3.836743	-1.367185
C	-1.839721	-2.678367	-1.365644
F	-4.605773	-4.898897	-1.970188
Pt	0.036581	0.204270	2.953349
P	-1.091388	-0.039856	4.974704
C	-0.528767	-1.487041	5.934524
C	-0.423353	-1.474018	7.331088
C	-0.019627	-2.615881	8.016751
C	0.274355	-3.768395	7.296176
C	0.172647	-3.810857	5.911537
C	-0.220042	-2.662609	5.235432
F	0.677731	-4.848259	7.948739
S	-1.819962	0.276733	1.509027
C	-2.610055	-1.326648	1.549702
C	-1.922182	-2.495813	1.880302
C	-2.599576	-3.706199	1.968487
C	-3.963419	-3.741903	1.687198
C	-4.657430	-2.588692	1.321771
C	-3.976876	-1.377301	1.262023
Cl	-4.814086	-5.256052	1.757401
S	2.254839	0.344596	3.742862
C	2.687470	-1.242720	4.441711
C	2.611333	-2.430667	3.711691
C	2.980072	-3.637193	4.293412
C	3.398548	-3.647802	5.622353
C	3.471957	-2.471373	6.367696
C	3.116862	-1.265806	5.771310
Cl	3.776321	-5.160719	6.390582
C	-2.263665	1.359227	-2.472028
C	-2.602475	1.584129	-3.814587
C	-3.447560	2.629240	-4.168469
C	-3.952584	3.451758	-3.167887
C	-3.636097	3.253779	-1.830654
C	-2.788063	2.205440	-1.487761
F	-4.753605	4.453414	-3.501055
C	-0.127355	-0.296249	-3.454822
C	0.657359	0.777631	-3.899954
C	1.545152	0.616397	-4.955284
C	1.661503	-0.637044	-5.548197
C	0.902655	-1.718084	-5.126405
C	0.002495	-1.540599	-4.078639
F	2.532549	-0.802136	-6.535261
C	-0.974840	1.373565	6.132698

C	-1.965677	1.603225	7.098332
C	-1.836307	2.639183	8.016230
C	-0.705805	3.446895	7.961566
C	0.291319	3.244033	7.016765
C	0.150240	2.205684	6.101549
F	-0.580915	4.438613	8.831437
C	-2.894363	-0.294203	4.780092
C	-3.680827	0.771220	4.317746
C	-5.037579	0.598861	4.076667
C	-5.600109	-0.657578	4.278395
C	-4.848723	-1.729001	4.736066
C	-3.492624	-1.540438	4.990543
F	-6.887293	-0.837023	4.010269
C	5.824434	1.421090	0.718278
C	7.158375	1.651396	1.086974
C	7.877507	2.702521	0.530602
C	7.252291	3.525983	-0.398826
C	5.933771	3.323354	-0.783452
C	5.222499	2.268912	-0.219475
F	7.933137	4.532172	-0.928408
H	-3.224886	1.751755	4.137233
H	-2.896637	-2.387237	5.344538
H	-0.648793	-0.565986	7.899027
H	-2.852354	0.961225	7.137564
H	0.929310	2.040258	5.350090
H	-0.267001	-2.699467	4.142003
H	-5.669541	1.415649	3.718898
H	0.079264	-2.628352	9.105032
H	0.427951	-4.721299	5.362138
H	-4.512721	-0.460195	0.991844
H	-0.839625	-2.485426	2.046879
H	-2.597251	2.834825	8.775874
H	-5.326802	-2.703511	4.867127
H	-2.049896	-4.612682	2.234610
H	-5.726825	-2.644490	1.100193
H	1.160094	3.906760	7.006094
H	0.576159	1.755808	-3.411723
H	-0.598612	-2.391830	-3.743695
H	-3.961901	-0.596842	-3.051325
H	-2.201120	0.932348	-4.598034
H	-2.528298	2.043395	-0.436582
H	-0.865101	-2.712216	-0.868418
H	2.163302	1.440028	-5.321464
H	-5.343642	-2.678888	-3.032641
H	-2.239459	-4.745505	-0.881905
H	3.999212	-0.389513	-2.946882
H	1.231470	-2.471049	-0.352972
H	-3.721833	2.822763	-5.208504
H	1.033507	-2.688569	-5.612164
H	1.761664	-4.590336	-1.460111
H	4.595049	-2.569679	-4.023069
H	-4.050886	3.929085	-1.078324
H	5.219948	1.821330	3.638733
H	6.101934	-2.328885	2.810457
H	7.204549	-0.519710	-0.450396
H	7.646864	0.998698	1.818485
H	4.180285	2.104201	-0.512334
H	3.791213	-2.676424	1.138071
H	6.081035	1.519714	5.970693
H	7.882408	-2.578548	-1.691244

H	4.493202	-4.689601	-0.086061
H	3.170477	-0.333089	6.344435
H	2.229312	-2.442569	2.686082
H	8.917255	2.899308	0.803513
H	6.914610	-2.609143	5.157609
H	2.896802	-4.558865	3.711149
H	3.800373	-2.508169	7.410083
H	5.480490	3.999310	-1.512496
B	0.914661	-4.814483	1.446812
F	0.990762	-6.178199	1.401559
F	2.200295	-4.243898	1.331455
F	0.118903	-4.323976	0.388651
F	0.350117	-4.382444	2.665038

Energies

Zero-point correction= 1.021337 (Hartree/Particle)
Thermal correction to Energy= 1.113481
Thermal correction to Enthalpy= 1.114426
Thermal correction to Gibbs Free Energy= 0.884546
Sum of electronic and zero-point Energies= -8045.498086
Sum of electronic and thermal Energies= -8045.405941
Sum of electronic and thermal Enthalpies= -8045.404997
Sum of electronic and thermal Free Energies= -8045.634877

	E (Thermal)	CV	S
	KCal/Mol	Cal/Mol-Kelvin	Cal/Mol-Kelvin
Total	698.720	349.204	483.823

# - Pd_3 cation 1 – BF_4^- (M06/Def2-svp)

146
scf done: -8072.121450

C	4.709941	-2.618483	0.535425
C	5.463823	-1.439413	0.446538
C	6.603783	-1.415880	-0.367067
C	6.981641	-2.549758	-1.079711
C	6.213528	-3.704216	-0.972429
C	5.085071	-3.758879	-0.163698
P	4.910508	0.001961	1.418163
C	5.635811	-0.252856	3.079978
C	5.629240	0.816459	3.987459
C	6.102638	0.650283	5.281875
C	6.558340	-0.604666	5.673334
C	6.570964	-1.682089	4.800612
C	6.109941	-1.499622	3.499256
F	6.976142	-0.776137	6.921369
F	6.560839	-4.772371	-1.674727
Pd	2.568745	0.229646	1.427144
S	2.173534	0.329365	-0.882865
C	2.555440	-1.253847	-1.602879
C	1.936105	-2.434735	-1.183726
C	2.230963	-3.642899	-1.802881
C	3.174996	-3.666123	-2.827204
C	3.815636	-2.500446	-3.247776
C	3.500971	-1.293521	-2.634181
Cl	3.598916	-5.179391	-3.567726
Pd	0.002407	0.199261	-0.009290

P	-1.194867	-0.050765	-2.019538
C	-2.307381	-1.496421	-1.981106
C	-3.585919	-1.489289	-2.552899
C	-4.371943	-2.637433	-2.528052
C	-3.870218	-3.788842	-1.930986
C	-2.607478	-3.822882	-1.352221
C	-1.836293	-2.667921	-1.370784
F	-4.624799	-4.877377	-1.907088
Pd	0.034121	0.215373	2.936481
P	-1.117000	-0.040044	4.978601
C	-0.534204	-1.481130	5.935794
C	-0.408507	-1.465238	7.330613
C	0.009957	-2.604461	8.011742
C	0.300305	-3.756159	7.288431
C	0.181609	-3.800667	5.905122
C	-0.226580	-2.655123	5.233516
F	0.715829	-4.833727	7.937391
S	-1.803508	0.289584	1.485280
C	-2.597121	-1.303532	1.542685
C	-1.906618	-2.473036	1.873785
C	-2.582464	-3.682629	1.980688
C	-3.949919	-3.720545	1.717657
C	-4.648110	-2.570417	1.349701
C	-3.968865	-1.359925	1.272019
Cl	-4.800730	-5.232362	1.821044
S	2.233883	0.363068	3.739118
C	2.676633	-1.215456	4.432675
C	2.601071	-2.402097	3.697488
C	2.979093	-3.610609	4.268610
C	3.406630	-3.627992	5.594533
C	3.481721	-2.455306	6.346077
C	3.117947	-1.248067	5.759915
Cl	3.799270	-5.143412	6.349403
C	-2.248747	1.363299	-2.507786
C	-2.592992	1.582965	-3.849889
C	-3.430495	2.633687	-4.205081
C	-3.922539	3.466849	-3.206691
C	-3.601006	3.273996	-1.869957
C	-2.760337	2.220014	-1.525709
F	-4.716165	4.473797	-3.541719
C	-0.134606	-0.323817	-3.486628
C	0.657158	0.737253	-3.950451
C	1.540380	0.554098	-5.005744
C	1.648300	-0.709587	-5.578283
C	0.881897	-1.778085	-5.139131
C	-0.014561	-1.578311	-4.092132
F	2.519539	-0.897237	-6.561278
C	-1.013436	1.372997	6.138751
C	-2.005278	1.600184	7.103969
C	-1.878837	2.637279	8.020971
C	-0.750727	3.448469	7.965979
C	0.247290	3.248204	7.021609
C	0.108951	2.208833	6.106987
F	-0.628996	4.440947	8.835548
C	-2.918238	-0.318209	4.794678
C	-3.718877	0.734994	4.327884
C	-5.074969	0.546526	4.096383
C	-5.622292	-0.715220	4.306781
C	-4.856166	-1.776121	4.764056
C	-3.501200	-1.570417	5.011949

F	-6.908862	-0.910136	4.046130
C	5.851781	1.422781	0.751350
C	7.181719	1.658256	1.130936
C	7.901733	2.710367	0.577438
C	7.281961	3.529028	-0.359893
C	5.967823	3.320511	-0.756214
C	5.255462	2.265354	-0.194805
F	7.964145	4.536027	-0.886389
H	-3.275066	1.719652	4.139782
H	-2.894146	-2.408700	5.367628
H	-0.632165	-0.557679	7.900102
H	-2.889223	0.954563	7.144290
H	0.888750	2.043333	5.355758
H	-0.290969	-2.694236	4.140871
H	-5.717839	1.354160	3.737299
H	0.122465	-2.615856	9.098726
H	0.436631	-4.709855	5.353466
H	-4.508242	-0.445047	1.001983
H	-0.822154	-2.462248	2.030067
H	-2.639895	2.830730	8.781100
H	-5.322015	-2.755855	4.900659
H	-2.030162	-4.586999	2.248104
H	-5.720264	-2.628324	1.142108
H	1.114348	3.913183	7.010967
H	0.583497	1.724178	-3.478896
H	-0.621323	-2.420035	-3.743575
H	-3.982890	-0.582809	-3.020912
H	-2.202077	0.922042	-4.630955
H	-2.497184	2.060519	-0.474623
H	-0.851117	-2.706235	-0.894837
H	2.163546	1.367652	-5.385627
H	-5.374190	-2.658051	-2.963596
H	-2.234227	-4.731363	-0.871489
H	3.994367	-0.369584	-2.956286
H	1.238737	-2.439691	-0.338920
H	-3.709462	2.823263	-5.244615
H	1.005255	-2.756957	-5.609978
H	1.741435	-4.558652	-1.460474
H	4.556449	-2.547807	-4.050817
H	-4.006219	3.957005	-1.119344
H	5.246069	1.795816	3.677535
H	6.113293	-2.349345	2.808906
H	7.203538	-0.504987	-0.459622
H	7.666356	1.008281	1.867348
H	4.216848	2.093530	-0.497768
H	3.808348	-2.671640	1.154934
H	6.112215	1.470984	6.003497
H	7.860701	-2.553958	-1.729230
H	4.485593	-4.671761	-0.103552
H	3.174752	-0.318482	6.337523
H	2.214538	-2.409391	2.672991
H	8.938748	2.911010	0.857937
H	6.925978	-2.654204	5.152881
H	2.897706	-4.528705	3.680985
H	3.820137	-2.496678	7.385169
H	5.518986	3.992071	-1.492039
B	0.921784	-4.804494	1.441315
F	1.001639	-6.167599	1.394933
F	2.205776	-4.229730	1.326060
F	0.124706	-4.314629	0.383916

F 0.356757 -4.374219 2.659882

Energies

Zero-point correction= 1.021746 (Hartree/Particle)
Thermal correction to Energy= 1.113658
Thermal correction to Enthalpy= 1.114603
Thermal correction to Gibbs Free Energy= 0.886136
Sum of electronic and zero-point Energies= -8071.099704
Sum of electronic and thermal Energies= -8071.007791
Sum of electronic and thermal Enthalpies= -8071.006847
Sum of electronic and thermal Free Energies= -8071.235314

	E (Thermal)	CV	S
	KCal/Mol	Cal/Mol-Kelvin	Cal/Mol-Kelvin
Total	698.831	349.016	480.849

- Pd_2Pt cation – BF_4^- (M06/Def2-svp)

146
scf done: -8063.587916

C	4.711704	-2.629562	0.540827
C	5.467879	-1.452337	0.447422
C	6.615595	-1.439000	-0.355310
C	6.999080	-2.580800	-1.052232
C	6.229452	-3.733724	-0.939326
C	5.092846	-3.778181	-0.141557
P	4.908748	-0.002782	1.404741
C	5.635243	-0.244501	3.068773
C	5.626711	0.830720	3.969138
C	6.099520	0.673775	5.264999
C	6.556772	-0.577840	5.665197
C	6.570906	-1.661113	4.799803
C	6.110372	-1.487804	3.497013
F	6.974750	-0.740165	6.914534
F	6.585108	-4.810752	-1.623926
Pd	2.562207	0.228040	1.409254
S	2.154569	0.316912	-0.908318
C	2.555708	-1.271777	-1.613206
C	1.939897	-2.454893	-1.197943
C	2.254207	-3.663012	-1.807453
C	3.215218	-3.682993	-2.815902
C	3.853061	-2.513844	-3.230966
C	3.517858	-1.306428	-2.628404
Cl	3.669659	-5.197059	-3.536839
Pt	-0.017214	0.194323	-0.022797
P	-1.195703	-0.044167	-2.001889
C	-2.306261	-1.489793	-1.971626
C	-3.581930	-1.475706	-2.549668
C	-4.371585	-2.621411	-2.532641
C	-3.875996	-3.776867	-1.938039
C	-2.615148	-3.818010	-1.355414
C	-1.840142	-2.665752	-1.366096
F	-4.634861	-4.862105	-1.919793
Pd	0.025717	0.212851	2.928225
P	-1.119927	-0.042607	4.978657
C	-0.541573	-1.486167	5.935483

C	-0.416328	-1.472947	7.330326
C	0.001474	-2.613495	8.009627
C	0.291438	-3.764166	7.284506
C	0.173357	-3.806127	5.901071
C	-0.234134	-2.659088	5.231478
F	0.704995	-4.843363	7.932148
S	-1.824522	0.283484	1.480157
C	-2.611281	-1.317613	1.543875
C	-1.915851	-2.483151	1.874384
C	-2.587880	-3.695517	1.978352
C	-3.955127	-3.737525	1.715219
C	-4.657822	-2.588917	1.351790
C	-3.982192	-1.375965	1.275272
Cl	-4.799981	-5.253523	1.810227
S	2.227026	0.370262	3.718491
C	2.670069	-1.206265	4.416791
C	2.591444	-2.395780	3.686542
C	2.969612	-3.602322	4.261787
C	3.399212	-3.614917	5.587080
C	3.475980	-2.439522	6.334149
C	3.112929	-1.234147	5.743629
Cl	3.791706	-5.128020	6.347008
C	-2.248029	1.372512	-2.481798
C	-2.578261	1.597794	-3.826526
C	-3.421896	2.642444	-4.184915
C	-3.934029	3.463828	-3.187018
C	-3.626922	3.264352	-1.847734
C	-2.780728	2.216346	-1.499934
F	-4.733131	4.465188	-3.524551
C	-0.122802	-0.304391	-3.460116
C	0.664414	0.762722	-3.917351
C	1.548427	0.588218	-4.973239
C	1.658970	-0.671511	-5.554458
C	0.897463	-1.745727	-5.120694
C	0.000572	-1.555061	-4.072374
F	2.527226	-0.848915	-6.541353
C	-1.012598	1.368673	6.140978
C	-2.002341	1.598532	7.107708
C	-1.872300	2.635981	8.023858
C	-0.742514	3.444679	7.966713
C	0.253642	3.241651	7.021016
C	0.111462	2.202135	6.107104
F	-0.617450	4.437571	8.835513
C	-2.922396	-0.314470	4.795079
C	-3.717882	0.741188	4.325075
C	-5.074182	0.557636	4.089414
C	-5.626755	-0.701673	4.299532
C	-4.865861	-1.764905	4.760595
C	-3.510879	-1.564365	5.011735
F	-6.913416	-0.892403	4.035378
C	5.851306	1.412425	0.726591
C	7.181837	1.651642	1.101277
C	7.899571	2.700960	0.539336
C	7.276841	3.512974	-0.401737
C	5.961735	3.301136	-0.793084
C	5.251756	2.249003	-0.222907
F	7.956958	4.517054	-0.936677
H	-3.270111	1.724468	4.138668
H	-2.907651	-2.404843	5.368865
H	-0.639732	-0.566299	7.901384

H	-2.887672	0.954890	7.149813
H	0.889587	2.034815	5.354463
H	-0.298140	-2.695509	4.138736
H	-5.713019	1.367470	3.728048
H	0.113479	-2.626879	9.096643
H	0.428254	-4.714370	5.347820
H	-4.523592	-0.462620	1.004149
H	-0.831252	-2.468195	2.028908
H	-2.631819	2.831617	8.784970
H	-5.336012	-2.742558	4.897316
H	-2.032149	-4.598577	2.243207
H	-5.729859	-2.649934	1.144548
H	1.122142	3.904734	7.008651
H	0.589225	1.745218	-3.437148
H	-0.602637	-2.400887	-3.727887
H	-3.974463	-0.565644	-3.014301
H	-2.172765	0.946248	-4.607878
H	-2.530691	2.051404	-0.446852
H	-0.857783	-2.707547	-0.885226
H	2.168967	1.405861	-5.348583
H	-5.372473	-2.636757	-2.971424
H	-2.246108	-4.729497	-0.877001
H	4.008768	-0.379683	-2.946895
H	1.232411	-2.461915	-0.362275
H	-3.689861	2.836169	-5.226542
H	1.023992	-2.721306	-5.597262
H	1.767481	-4.581066	-1.466691
H	4.610828	-2.559813	-4.017989
H	-4.048056	3.937933	-1.097391
H	5.242675	1.807564	3.652294
H	6.115042	-2.342300	2.812594
H	7.217758	-0.530067	-0.451426
H	7.668697	1.007015	1.840961
H	4.211919	2.075545	-0.520668
H	3.804018	-2.675423	1.152071
H	6.107723	1.499398	5.981033
H	7.884655	-2.592837	-1.692776
H	4.492137	-4.689844	-0.076081
H	3.171837	-0.302204	6.317242
H	2.202579	-2.406969	2.662810
H	8.937034	2.904511	0.816110
H	6.926914	-2.630457	5.158661
H	2.886180	-4.522776	3.678110
H	3.815523	-2.477161	7.373001
H	5.510319	3.968004	-1.531605
B	0.916755	-4.807474	1.434388
F	0.998052	-6.170498	1.384704
F	2.200011	-4.231256	1.320069
F	0.118817	-4.317065	0.377189
F	0.351080	-4.380153	2.653276

Energies

Zero-point correction= 1.021278 (Hartree/Particle)
Thermal correction to Energy= 1.113309
Thermal correction to Enthalpy= 1.114253
Thermal correction to Gibbs Free Energy= 0.885121
Sum of electronic and zero-point Energies= -8062.566638
Sum of electronic and thermal Energies= -8062.474607
Sum of electronic and thermal Enthalpies= -8062.473663

Sum of electronic and thermal Free Energies= -8062.702795

	E (Thermal)	CV	S
	KCal/Mol	Cal/Mol-Kelvin	Cal/Mol-Kelvin
Total	698.612	349.241	482.248

# - $PdPt_2$ cation – BF_4^- (M06/Def2-svp)

146
scf done: -8055.053856

C	4.717665	-2.616188	0.507182
C	5.460808	-1.429325	0.427030
C	6.611100	-1.393066	-0.370938
C	7.011678	-2.523067	-1.076652
C	6.256524	-3.686315	-0.976240
C	5.115957	-3.753137	-0.185055
P	4.893933	0.007343	1.395946
C	5.613358	-0.239240	3.060807
C	5.609927	0.833179	3.964371
C	6.078669	0.668041	5.260520
C	6.530114	-0.587352	5.655929
C	6.542693	-1.666739	4.785701
C	6.084975	-1.485907	3.482983
F	6.944561	-0.756723	6.905000
F	6.631095	-4.752324	-1.667219
Pt	2.586212	0.217376	1.410162
S	2.176708	0.290050	-0.916509
C	2.557331	-1.308982	-1.620162
C	1.933957	-2.483681	-1.195521
C	2.227045	-3.695331	-1.809401
C	3.170972	-3.725481	-2.833489
C	3.818426	-2.563936	-3.255067
C	3.506468	-1.353165	-2.645863
Cl	3.583808	-5.243490	-3.572294
Pt	-0.003357	0.185234	-0.022710
P	-1.185344	-0.051218	-2.004107
C	-2.297325	-1.496353	-1.975255
C	-3.570361	-1.484680	-2.558956
C	-4.360155	-2.630350	-2.541487
C	-3.867163	-3.783765	-1.940873
C	-2.609129	-3.822567	-1.351990
C	-1.834023	-2.670290	-1.363474
F	-4.625715	-4.869331	-1.923226
Pd	0.043230	0.205238	2.931375
P	-1.109906	-0.048248	4.984991
C	-0.544240	-1.494860	5.946362
C	-0.437175	-1.486719	7.342734
C	-0.029369	-2.629807	8.024028
C	0.267922	-3.778622	7.299038
C	0.167080	-3.815897	5.914183
C	-0.229954	-2.666164	5.242766
F	0.672469	-4.860553	7.948105
S	-1.803471	0.287961	1.482486
C	-2.596493	-1.310379	1.546296
C	-1.906392	-2.477827	1.881179
C	-2.582888	-3.687719	1.984657
C	-3.949042	-3.725681	1.715499
C	-4.646575	-2.575174	1.348232
C	-3.966763	-1.364479	1.273311

Cl	-4.799109	-5.239067	1.807096
S	2.253386	0.353823	3.723723
C	2.678417	-1.227615	4.431074
C	2.608866	-2.418745	3.703998
C	2.976966	-3.623361	4.289547
C	3.387596	-3.631021	5.620938
C	3.452203	-2.453170	6.365114
C	3.098205	-1.249562	5.764806
Cl	3.769665	-5.141296	6.392048
C	-2.234712	1.368188	-2.484124
C	-2.570287	1.591466	-3.827904
C	-3.406413	2.642291	-4.185899
C	-3.905700	3.472108	-3.188439
C	-3.592914	3.275214	-1.850166
C	-2.754008	2.221074	-1.502915
F	-4.697467	4.479531	-3.525666
C	-0.115389	-0.311187	-3.464737
C	0.679561	0.752824	-3.915635
C	1.561872	0.578402	-4.973362
C	1.662720	-0.678268	-5.562419
C	0.892738	-1.749417	-5.135469
C	-0.002014	-1.558732	-4.085598
F	2.530108	-0.856636	-6.550207
C	-0.997052	1.363054	6.147717
C	-1.982022	1.597667	7.118173
C	-1.846409	2.637861	8.030485
C	-0.715794	3.444782	7.965853
C	0.275564	3.237686	7.016094
C	0.127602	2.195426	6.106155
F	-0.585403	4.440167	8.831230
C	-2.914434	-0.309683	4.800436
C	-3.703052	0.751141	4.330662
C	-5.059484	0.575350	4.089596
C	-5.619257	-0.681629	4.294437
C	-4.865331	-1.749898	4.755219
C	-3.510069	-1.557120	5.011367
F	-6.906139	-0.865105	4.025461
C	5.820229	1.437301	0.729383
C	7.148412	1.678117	1.111723
C	7.865043	2.734306	0.561712
C	7.243273	3.551801	-0.375153
C	5.930667	3.337977	-0.774018
C	5.221836	2.278562	-0.216597
F	7.921580	4.562771	-0.898527
H	-3.249984	1.732844	4.148673
H	-2.912299	-2.401879	5.367552
H	-0.666699	-0.581698	7.913981
H	-2.868511	0.955949	7.165748
H	0.901482	2.026057	5.349766
H	-0.280170	-2.699417	4.149143
H	-5.692798	1.389549	3.728295
H	0.068865	-2.646575	9.112338
H	0.425212	-4.723826	5.362152
H	-4.504648	-0.449551	1.000394
H	-0.822262	-2.466645	2.039519
H	-2.602401	2.837217	8.794133
H	-5.340882	-2.725538	4.887641
H	-2.031747	-4.592243	2.253941
H	-5.718062	-2.632752	1.137178
H	1.144661	3.899855	6.997469

H	0.610714	1.733138	-3.429891
H	-0.612269	-2.401761	-3.746495
H	-3.961041	-0.576337	-3.028495
H	-2.173734	0.933886	-4.608890
H	-2.498527	2.059010	-0.450707
H	-0.853799	-2.710164	-0.877938
H	2.188243	1.393776	-5.343993
H	-5.358995	-2.647286	-2.984877
H	-2.242647	-4.732406	-0.868480
H	4.005695	-0.431982	-2.967364
H	1.236289	-2.481332	-0.351774
H	-3.678015	2.834704	-5.226833
H	1.011567	-2.722811	-5.618542
H	1.734426	-4.608098	-1.463353
H	4.562285	-2.616926	-4.054916
H	-4.003623	3.955638	-1.100201
H	5.230116	1.812679	3.651194
H	6.087847	-2.337217	2.794668
H	7.201124	-0.475297	-0.457156
H	7.634710	1.029572	1.848291
H	4.184706	2.104333	-0.521709
H	3.807608	-2.675864	1.113838
H	6.089121	1.490299	5.980346
H	7.899616	-2.518663	-1.713975
H	4.527968	-4.674277	-0.130935
H	3.149036	-0.315612	6.336236
H	2.235549	-2.433124	2.675058
H	8.900347	2.939318	0.845229
H	6.895005	-2.638744	5.140803
H	2.899661	-4.546069	3.708257
H	3.774727	-2.487431	7.409434
H	5.480114	4.009206	-1.509073
B	0.923007	-4.814023	1.448270
F	1.001590	-6.177598	1.405288
F	2.207665	-4.241711	1.326204
F	0.121516	-4.327536	0.392035
F	0.362138	-4.379755	2.666793

Energies

Zero-point correction= 1.021132 (Hartree/Particle)
Thermal correction to Energy= 1.113276
Thermal correction to Enthalpy= 1.114220
Thermal correction to Gibbs Free Energy= 0.884254
Sum of electronic and zero-point Energies= -8054.032724
Sum of electronic and thermal Energies= -8053.940580
Sum of electronic and thermal Enthalpies= -8053.939636
Sum of electronic and thermal Free Energies= -8054.169602

	E (Thermal)	CV	S
	KCal/Mol	Cal/Mol-Kelvin	Cal/Mol-Kelvin
Total	698.591	349.298	484.004

# - Li+ (M06/Def2-svp)

Energies

Zero-point correction= 0.000000 (Hartree/Particle)

Thermal correction to Energy= 0.001416
Thermal correction to Enthalpy= 0.002360
Thermal correction to Gibbs Free Energy= -0.012748
Sum of electronic and zero-point Energies= -7.286810
Sum of electronic and thermal Energies= -7.285394
Sum of electronic and thermal Enthalpies= -7.284449
Sum of electronic and thermal Free Energies= -7.299558

	E (Thermal)	CV	S
	KCal/Mol	Cal/Mol-Kelvin	Cal/Mol-Kelvin
Total	0.889	2.981	31.798

# - BF_4^- (M06/Def2-svp)

5
scf done: -424.069598

B	1.126655	-6.691637	1.428312
F	0.457699	-6.279003	2.585427
F	1.203900	-8.088108	1.406370
F	2.417906	-6.152996	1.419812
F	0.428388	-6.245089	0.301309

Energies

Zero-point correction= 0.014847 (Hartree/Particle)
Thermal correction to Energy= 0.019141
Thermal correction to Enthalpy= 0.020086
Thermal correction to Gibbs Free Energy= -0.012703
Sum of electronic and zero-point Energies= -424.054751
Sum of electronic and thermal Energies= -424.050457
Sum of electronic and thermal Enthalpies= -424.049513
Sum of electronic and thermal Free Energies= -424.082301

	E (Thermal)	CV	S
	KCal/Mol	Cal/Mol-Kelvin	Cal/Mol-Kelvin
Total	12.011	14.051	69.009

# - H_2O (M06/Def2-svp)

3
scf done: -76.324421

O	1.552756	7.531096	2.266828
H	0.872008	8.174863	2.486985
H	2.363193	7.956919	2.563967

Energies

Zero-point correction= 0.021579 (Hartree/Particle)
Thermal correction to Energy= 0.024415
Thermal correction to Enthalpy= 0.025359
Thermal correction to Gibbs Free Energy= 0.003274
Sum of electronic and zero-point Energies= -76.302841
Sum of electronic and thermal Energies= -76.300006
Sum of electronic and thermal Enthalpies= -76.299062
Sum of electronic and thermal Free Energies= -76.321146

	E (Thermal)	CV	S
	KCal/Mol	Cal/Mol-Kelvin	Cal/Mol-Kelvin
Total	15.321	6.011	46.481

# - Benzene (M06/Def2-svp)

12
scf done: -231.887011

C	4.928116	2.513115	0.320265
C	5.675595	1.539545	0.980183
C	7.024646	1.761084	1.252239
C	7.626666	2.956252	0.863682
C	6.878997	3.930701	0.203553
C	5.529950	3.708790	-0.068400
H	5.203325	0.601043	1.285076
H	7.351931	4.868899	-0.101259
H	7.609756	0.995501	1.770547
H	3.869003	2.338656	0.107840
H	8.685614	3.131371	1.075856
H	4.944642	4.474173	-0.586644

Energies

Zero-point correction= 0.099951 (Hartree/Particle)
Thermal correction to Energy= 0.104361
Thermal correction to Enthalpy= 0.105306
Thermal correction to Gibbs Free Energy= 0.072484
Sum of electronic and zero-point Energies= -231.787059
Sum of electronic and thermal Energies= -231.782649
Sum of electronic and thermal Enthalpies= -231.781705
Sum of electronic and thermal Free Energies= -231.814526

	E (Thermal)	CV	S
	KCal/Mol	Cal/Mol-Kelvin	Cal/Mol-Kelvin
Total	65.488	17.292	69.078

# - Li^+-H_2O (M06/Def2-svp)

4
scf done: -83.677008

O	1.452905	7.555368	2.265266
Li	0.952005	5.982491	1.468476
H	0.879763	8.287030	2.540802
H	2.348284	7.837990	2.506819

Energies

Zero-point correction= 0.024802 (Hartree/Particle)
Thermal correction to Energy= 0.028288
Thermal correction to Enthalpy= 0.029233
Thermal correction to Gibbs Free Energy= 0.003019
Sum of electronic and zero-point Energies= -83.652206
Sum of electronic and thermal Energies= -83.648720
Sum of electronic and thermal Enthalpies= -83.647776
Sum of electronic and thermal Free Energies= -83.673990

	E (Thermal)	CV	S
	KCal/Mol	Cal/Mol-Kelvin	Cal/Mol-Kelvin
Total	17.751	9.665	55.172

# - Li^+-benzene (M06/Def2-svp)

13
scf done: -239.228410

C	4.755601	2.483800	-0.057717
C	5.331471	1.716447	0.963119
C	6.513237	2.146524	1.580659
C	7.119332	3.343456	1.177402
C	6.543449	4.110758	0.156195
C	5.361618	3.680850	-0.461227
H	4.865600	0.775333	1.269172
H	7.024074	5.038250	-0.167935
Li	4.880833	3.895720	1.905236
H	6.969976	1.541065	2.368739
H	3.840742	2.141756	-0.550154
H	8.048855	3.672923	1.650497
H	4.918453	4.272249	-1.267464

Energies

Zero-point correction= 0.101488 (Hartree/Particle)
Thermal correction to Energy= 0.107546
Thermal correction to Enthalpy= 0.108490
Thermal correction to Gibbs Free Energy= 0.072296
Sum of electronic and zero-point Energies= -239.126921
Sum of electronic and thermal Energies= -239.120864
Sum of electronic and thermal Enthalpies= -239.119920
Sum of electronic and thermal Free Energies= -239.156113

	E (Thermal)	CV	S
	KCal/Mol	Cal/Mol-Kelvin	Cal/Mol-Kelvin
Total	67.486	22.680	76.176

Pd_3^+ Complex 1 + $AgBF_4$ (M06/Def2-svp)

158
scf done: -8795.906361

C	11.749867	15.601863	25.179554
C	12.510933	15.381514	24.024927
C	13.905168	15.347149	24.106962
C	14.542970	15.513459	25.330670
C	13.772444	15.727556	26.471354
C	12.379618	15.779582	26.405674
S	11.642431	15.103298	22.494143
Pd	13.055962	13.752336	21.168853
S	14.372102	12.949681	19.386264
Pd	14.019470	15.197705	18.787071
S	13.198036	17.408237	18.761370
Pd	12.416746	16.591869	20.826725
P	11.598087	18.606368	21.702590
C	12.067402	18.870916	23.441492
C	13.389365	18.566567	23.801867
C	13.833757	18.760035	25.103661

C	12.936391	19.245003	26.047459
C	11.616989	19.540872	25.721686
C	11.182213	19.350282	24.413793
F	13.338771	19.417033	27.296846
Cl	14.554157	15.945977	28.007310
C	12.181934	20.092345	20.808414
C	11.651913	20.365310	19.537777
C	12.137038	21.423957	18.781487
C	13.175947	22.194592	19.293820
C	13.724030	21.943777	20.542920
C	13.221069	20.888763	21.299237
F	13.666149	23.183670	18.559012
C	9.778514	18.707850	21.601430
C	9.031072	17.547808	21.365604
C	7.642870	17.600330	21.283675
C	7.009713	18.825801	21.439914
C	7.724302	19.996798	21.672851
C	9.110026	19.933208	21.748510
F	5.689771	18.886022	21.359899
Ag	11.285663	14.640749	19.131185
O	9.820272	15.376147	17.444477
F	11.489558	12.616511	17.727824
B	10.432359	11.983657	17.010644
F	9.811730	12.946067	16.208390
O	9.450099	13.325875	20.022249
P	15.454007	15.239209	16.921787
C	17.184601	15.272613	17.482775
C	17.578202	16.366986	18.271965
C	18.840213	16.421676	18.844376
C	19.711960	15.360794	18.626644
C	19.352079	14.262073	17.855297
C	18.081222	14.214995	17.290634
F	20.912202	15.392622	19.182434
C	15.230410	13.848864	15.761253
C	16.074004	13.704025	14.648418
C	15.853986	12.685885	13.730263
C	14.771359	11.828685	13.919204
C	13.903414	11.965688	14.992476
C	14.144515	12.981109	15.914712
F	14.566944	10.863266	13.035726
C	15.247868	16.699624	15.832891
C	13.948785	16.984833	15.388592
C	13.709784	18.059937	14.543722
C	14.783326	18.851446	14.145675
C	16.079092	18.591223	14.567022
C	16.307418	17.509337	15.415089
F	14.558059	19.882799	13.343095
P	13.119105	11.776667	22.448261
C	14.821911	11.155837	22.671925
C	15.802137	12.078451	23.070947
C	17.119270	11.679745	23.260967
C	17.458268	10.356842	23.006460
C	16.513378	9.425126	22.592513
C	15.192190	9.828864	22.423957
F	18.721445	9.978045	23.131957
C	12.423710	11.902770	24.135768
C	13.232364	12.136462	25.253873
C	12.668307	12.366034	26.504818
C	11.286927	12.361488	26.629456
C	10.455442	12.116084	25.542242

C	11.030674	11.897747	24.296796
F	10.742461	12.607244	27.813591
C	12.158847	10.431098	21.675218
C	11.716689	10.535795	20.349729
C	11.021430	9.490053	19.745850
C	10.774968	8.340776	20.483869
C	11.188122	8.211038	21.807067
C	11.877997	9.259825	22.399714
F	10.119864	7.338219	19.921249
C	14.637149	18.396265	19.126458
C	15.411663	18.193066	20.273734
C	16.536205	18.972705	20.513700
C	16.887776	19.956215	19.588334
C	16.128120	20.171717	18.438335
C	14.997737	19.391296	18.210187
Cl	18.306189	20.920765	19.866446
C	16.077360	12.835979	19.882981
C	16.762649	13.845695	20.570169
C	18.102242	13.679642	20.894466
C	18.746534	12.495537	20.542595
C	18.078676	11.477386	19.863756
C	16.742131	11.654196	19.529650
Cl	20.410096	12.264427	20.982985
F	9.517046	11.532389	17.983910
F	10.933859	10.949524	16.283653
F	16.810589	15.036335	23.316976
B	17.143907	16.399952	23.162885
F	17.926585	16.555710	22.015318
F	15.933146	17.113812	22.965456
F	17.770465	16.857980	24.291277
H	16.908379	14.397343	14.492762
H	16.497266	12.545889	12.858213
H	13.045000	11.296921	15.103753
H	13.452382	13.091986	16.755763
H	13.106800	16.361612	15.716127
H	12.706269	18.298322	14.183023
H	16.892398	19.237375	14.227160
H	17.328477	17.300945	15.749858
H	16.875014	17.182903	18.468465
H	19.135965	17.241556	19.504490
H	20.066615	13.443318	17.732615
H	17.786633	13.331795	16.715375
H	10.853529	19.736308	19.127537
H	11.736837	21.656091	17.791387
H	14.545451	22.568671	20.902632
H	13.657359	20.686036	22.282447
H	9.539905	16.586520	21.234587
H	7.043572	16.706482	21.094438
H	7.182144	20.939171	21.783701
H	9.682283	20.852214	21.916888
H	14.102589	18.164370	23.073738
H	14.861497	18.513112	25.388309
H	10.947239	19.912577	26.501144
H	10.141870	19.576005	24.159052
H	14.322258	12.146838	25.159588
H	13.286838	12.570601	27.383267
H	9.372568	12.109392	25.687960
H	10.380599	11.709887	23.434683
H	11.910756	11.441536	19.762444
H	10.669317	9.565827	18.713975

H	10.958944	7.290197	22.349448
H	12.200198	9.170356	23.443453
H	15.564896	13.138336	23.221819
H	17.887957	12.399149	23.558522
H	16.829235	8.397517	22.395333
H	14.451636	9.099727	22.081703
H	15.167747	17.406512	20.995748
H	17.146806	18.780300	21.399620
H	16.420773	20.952668	17.731139
H	14.382526	19.561787	17.319635
H	16.254110	14.762168	20.893263
H	18.621626	14.465786	21.446640
H	18.606356	10.552318	19.615595
H	16.197121	10.863189	19.003459
H	14.522642	15.209111	23.213099
H	15.635763	15.499908	25.378746
H	11.800169	15.952325	27.316713
H	10.657197	15.631685	25.114701
H	9.779288	14.608867	16.832598
H	8.949907	15.385622	17.863081
H	9.438952	12.560737	19.382002
H	9.536369	12.859558	20.863722

Zero-point correction= 1.040810 (Hartree/Particle)
Thermal correction to Energy= 1.140189
Thermal correction to Enthalpy= 1.141133
Thermal correction to Gibbs Free Energy= 0.898030
Sum of electronic and zero-point Energies= -8642.144654
Sum of electronic and thermal Energies= -8642.045275
Sum of electronic and thermal Enthalpies= -8642.044331
Sum of electronic and thermal Free Energies= -8642.287433

	E (Thermal)	CV	S
	KCal/Mol	Cal/Mol-Kelvin	Cal/Mol-Kelvin
Total	715.479	373.840	511.652

Complex 2-SbF_6 optimized at the M06/Def2-svp $(Pd_3Ag)^{++}$

204
scf done: -9638.568888

O	27.003465	3.348137	3.859829
C	27.261969	2.859908	2.556508
C	27.982429	1.534015	2.774384
C	28.690856	1.731387	4.129206
C	28.164366	3.082122	4.628885
O	24.626271	3.528725	5.210482
Ag	22.747273	4.044030	3.871611
Pd	20.359504	3.795385	5.420239
Pd	22.436543	5.795740	6.077698
Pd	20.814153	6.136025	3.608943
S	22.716475	7.325976	4.310148
C	22.491349	9.037926	4.737532
C	21.249605	9.625174	4.991268
C	21.165805	10.985502	5.270071
C	22.328123	11.752788	5.306420
C	23.577566	11.176869	5.067014
C	23.653760	9.821926	4.774299
Cl	22.233405	13.440850	5.680562

P	20.099075	7.392265	1.738558
C	20.888007	9.029150	1.576855
C	22.278048	9.086765	1.394510
C	22.940495	10.304959	1.370913
C	22.204981	11.472697	1.561007
C	20.827927	11.447812	1.728252
C	20.169772	10.219616	1.731183
F	22.842531	12.630193	1.590749
C	18.308226	7.711814	1.762670
C	17.705991	8.029828	2.989603
C	16.346574	8.306323	3.061004
C	15.587143	8.230104	1.899458
C	16.156702	7.921942	0.668177
C	17.523224	7.664569	0.602870
F	14.282185	8.430658	1.972417
C	20.417320	6.575812	0.128852
C	20.257469	5.187624	0.014030
C	20.408802	4.553101	-1.215058
C	20.733419	5.314947	-2.332049
C	20.898666	6.693065	-2.250010
C	20.734744	7.318989	-1.017770
F	20.890617	4.713634	-3.499079
S	19.307043	4.336442	3.397383
C	17.650567	4.871165	3.764873
C	17.292382	5.418326	5.000802
C	15.972873	5.776319	5.251143
C	15.019715	5.606887	4.246819
C	15.364811	5.068166	3.006344
C	16.682443	4.696621	2.769307
Cl	13.386037	6.114579	4.519191
P	24.031861	6.646181	7.581259
C	23.755461	8.398159	7.987575
C	22.434711	8.866719	8.003429
C	22.149488	10.182040	8.351898
C	23.205441	11.032541	8.655558
C	24.528230	10.597018	8.641490
C	24.801099	9.275216	8.307563
F	22.951558	12.295308	8.952760
C	25.756828	6.511798	6.985395
C	26.007485	6.411339	5.611413
C	27.309250	6.335150	5.128552
C	28.362453	6.347115	6.032698
C	28.150271	6.443555	7.402866
C	26.843071	6.527084	7.873845
F	29.602674	6.237285	5.573029
C	24.044730	5.776741	9.186824
C	23.305815	6.258583	10.273947
C	23.212835	5.518522	11.450109
C	23.864277	4.296407	11.528427
C	24.612396	3.795809	10.466289
C	24.685060	4.533437	9.291336
F	23.756935	3.572379	12.632231
S	21.939145	3.762480	7.171511
C	21.096944	4.044957	8.711420
C	20.413773	5.233596	8.985171
C	19.822002	5.426895	10.227567
C	19.895844	4.417633	11.184653
C	20.559181	3.218355	10.921220
C	21.165939	3.038524	9.683277
Cl	19.130723	4.647157	12.724292

P	18.991934	1.959759	6.028894
C	18.327496	2.049320	7.722329
C	17.893029	3.294840	8.199035
C	17.313450	3.410172	9.456901
C	17.184113	2.268765	10.240904
C	17.614314	1.022218	9.797256
C	18.187229	0.915394	8.534280
F	16.647190	2.368723	11.443811
C	17.520834	1.716703	4.967173
C	16.225543	2.000944	5.412684
C	15.136513	1.882124	4.551705
C	15.354323	1.470667	3.244901
C	16.628959	1.161601	2.778436
C	17.708063	1.299177	3.641022
F	14.328453	1.380579	2.413404
C	19.866469	0.356196	5.906822
C	21.261393	0.320092	6.021357
C	21.948131	-0.888472	5.943756
C	21.232301	-2.059373	5.733914
C	19.846150	-2.057285	5.618815
C	19.168122	-0.845843	5.709018
F	21.890337	-3.203648	5.626554
O	22.430553	1.981929	2.686101
C	23.339040	0.954987	3.083584
C	22.800349	-0.336502	2.478660
C	21.317977	-0.020785	2.300961
C	21.373201	1.440834	1.903913
O	24.226678	4.393012	1.993309
C	23.710387	4.080916	0.696355
C	24.091026	5.255532	-0.187575
C	25.381785	5.734667	0.464432
C	25.047728	5.561084	1.933076
O	24.450392	1.048517	6.446492
C	24.424008	0.842563	7.861525
C	25.454673	-0.240590	8.123642
C	26.492131	0.058770	7.048614
C	25.604226	0.435088	5.875974
F	19.555894	8.206810	9.058752
Sb	18.305888	8.307041	7.597482
F	17.664316	6.531371	8.016599
F	19.658723	7.426182	6.489578
F	16.977027	9.100727	8.692411
F	19.053943	10.001098	7.097606
F	17.166481	8.357702	6.050640
H	18.288242	8.053743	3.920821
H	15.870228	8.546968	4.016647
H	15.518741	7.880932	-0.220856
H	17.973529	7.417066	-0.366577
H	20.000973	4.589652	0.899796
H	20.280634	3.470909	-1.326400
H	21.145686	7.258316	-3.154720
H	20.848320	8.408048	-0.953477
H	22.857228	8.158967	1.281767
H	24.025074	10.374804	1.237352
H	20.289566	12.390659	1.869884
H	19.082623	10.195444	1.873167
H	25.165684	6.392182	4.909071
H	27.523700	6.248710	4.057313
H	29.010259	6.451611	8.080652
H	26.670332	6.599249	8.955264

H	22.777489	7.218558	10.206764
H	22.619410	5.865739	12.303215
H	25.114311	2.828152	10.580348
H	25.248337	4.138836	8.433820
H	21.604197	8.201717	7.730243
H	21.117620	10.549347	8.363290
H	25.325538	11.306036	8.887419
H	25.843995	8.936510	8.286601
H	16.052125	2.325126	6.445974
H	14.117461	2.116148	4.877884
H	16.752190	0.829036	1.742434
H	18.717496	1.069129	3.274494
H	21.830222	1.249634	6.164226
H	23.039605	-0.925220	6.030456
H	19.319403	-3.003845	5.458921
H	18.074564	-0.838808	5.617940
H	18.002560	4.203669	7.592490
H	16.979902	4.380693	9.840969
H	18.529188	-0.066795	8.186177
H	17.494877	0.152951	10.452176
H	23.360717	0.926100	4.192852
H	24.357180	1.215632	2.725338
H	22.985853	-1.216340	3.120729
H	23.268262	-0.534219	1.495365
H	20.784410	-0.130266	3.266082
H	20.817957	-0.659376	1.552647
H	21.611253	1.554106	0.822668
H	20.456068	2.022380	2.119384
H	22.617276	3.928916	0.769941
H	24.168086	3.128317	0.351646
H	23.311638	6.042824	-0.123550
H	24.201435	4.978428	-1.250051
H	25.653365	6.772659	0.204578
H	26.228396	5.081301	0.174711
H	25.921340	5.395686	2.592118
H	24.474502	6.429996	2.323628
H	18.058031	5.598059	5.766803
H	15.698932	6.225452	6.210995
H	14.596972	4.950090	2.233268
H	16.967634	4.276654	1.795889
H	20.341759	9.013702	5.010804
H	20.195599	11.438422	5.497484
H	24.478676	11.797832	5.109393
H	24.630586	9.360755	4.576487
H	20.323802	6.013005	8.218183
H	19.293506	6.361249	10.437975
H	20.604350	2.440115	11.690682
H	21.700463	2.103696	9.464470
H	23.395246	0.569671	8.161786
H	24.678510	1.793418	8.374163
H	26.064286	1.153177	5.166747
H	25.297245	-0.461374	5.293021
H	25.010740	-1.242585	7.960401
H	25.856117	-0.216479	9.151019
H	27.164036	-0.788175	6.827364
H	27.120288	0.919752	7.350886
H	27.908682	3.586228	2.014926
H	26.294319	2.794044	2.025948
H	28.907336	3.893180	4.472222
H	27.869077	3.100595	5.695254

H	28.444767	0.915285	4.832615
H	29.789712	1.743165	4.031880
H	28.680008	1.297405	1.953346
H	27.252004	0.705402	2.827958
H	25.477414	3.451503	4.719064
H	24.488635	2.720670	5.754171

Zero-point correction= 1.526513 (Hartree/Particle)
Thermal correction to Energy= 1.649266
Thermal correction to Enthalpy= 1.650210
Thermal correction to Gibbs Free Energy= 1.362127
Sum of electronic and zero-point Energies= -9637.042375
Sum of electronic and thermal Energies= -9636.919622
Sum of electronic and thermal Enthalpies= -9636.918677
Sum of electronic and thermal Free Energies= -9637.206761

	E (Thermal)	CV	S
	KCal/Mol	Cal/Mol-Kelvin	Cal/Mol-Kelvin
Total	1034.930	462.330	606.323

Complex 2-SbF_6 optimized at the M11/Def2-svp $(Pd_3Ag)^{++}$

204
scf done: -9638.069597

O	27.045771	3.327131	4.096895
C	27.357086	2.679733	2.872073
C	28.086571	1.396635	3.291924
C	28.783906	1.802483	4.613288
C	28.188350	3.190841	4.939276
O	24.545946	3.573321	5.200760
Ag	22.726658	4.055357	3.802776
Pd	20.298988	3.856627	5.374554
Pd	22.361437	5.794143	6.014446
Pd	20.724442	6.131305	3.618351
S	22.603574	7.298129	4.275856
C	22.371645	9.009378	4.709832
C	21.118408	9.583538	4.907338
C	21.015072	10.934209	5.237081
C	22.172304	11.693865	5.373259
C	23.435907	11.131267	5.178314
C	23.531299	9.787348	4.839432
Cl	22.053781	13.368296	5.825284
P	20.116430	7.306927	1.752568
C	21.048247	8.860117	1.572117
C	22.429954	8.745375	1.346473
C	23.244517	9.866307	1.388528
C	22.662228	11.103261	1.671106
C	21.294899	11.250120	1.850066
C	20.483002	10.114472	1.799885
F	23.452385	12.170148	1.781314
C	18.346454	7.712577	1.807747
C	17.782114	8.004918	3.059924
C	16.421409	8.262767	3.179549
C	15.629162	8.183971	2.039787
C	16.159078	7.900133	0.785027
C	17.528041	7.667724	0.671515
F	14.312678	8.347455	2.159955
C	20.387178	6.474862	0.143930

C	20.292395	5.082428	0.033558
C	20.435226	4.457262	-1.204125
C	20.675274	5.238911	-2.328772
C	20.773068	6.625029	-2.251414
C	20.626236	7.238197	-1.010424
F	20.815873	4.642932	-3.511489
S	19.233627	4.379510	3.397137
C	17.575953	4.895985	3.803241
C	17.260826	5.399669	5.066918
C	15.942967	5.716789	5.381329
C	14.956613	5.554343	4.409424
C	15.264218	5.078319	3.134228
C	16.580859	4.740307	2.834137
Cl	13.310180	5.967010	4.782820
P	23.987117	6.576006	7.411719
C	23.701581	8.308329	7.882555
C	22.377034	8.766759	7.882475
C	22.068498	10.053996	8.312094
C	23.109525	10.882959	8.713504
C	24.438374	10.463020	8.701529
C	24.731711	9.167314	8.288621
F	22.836393	12.121839	9.116712
C	25.694268	6.476819	6.766411
C	25.916483	6.396467	5.386611
C	27.212315	6.384304	4.878316
C	28.279036	6.435038	5.765933
C	28.094997	6.493006	7.142697
C	26.792987	6.513237	7.638648
F	29.523713	6.384543	5.276160
C	24.073397	5.658336	8.986755
C	23.428716	6.130847	10.134146
C	23.364749	5.343208	11.283395
C	23.964911	4.092889	11.267784
C	24.640309	3.610522	10.148452
C	24.674230	4.391285	8.998377
F	23.874988	3.313262	12.346958
S	21.889082	3.783622	7.067395
C	21.138881	4.050469	8.661658
C	20.558585	5.274614	8.998204
C	20.016817	5.465120	10.266153
C	20.059401	4.419834	11.186393
C	20.629794	3.188501	10.862303
C	21.170956	3.006992	9.592818
Cl	19.362063	4.643198	12.762926
P	19.020629	2.030969	5.929562
C	18.446715	2.080039	7.653552
C	18.043978	3.323354	8.162818
C	17.512929	3.426809	9.443505
C	17.413835	2.273560	10.215876
C	17.823882	1.030230	9.742525
C	18.338242	0.935176	8.451413
F	16.925804	2.358844	11.451472
C	17.491106	1.811024	4.949114
C	16.239769	2.177823	5.456250
C	15.099166	2.088550	4.659610
C	15.226829	1.623501	3.358099
C	16.456520	1.239875	2.829002
C	17.588332	1.347128	3.629248
F	14.144069	1.552236	2.582526
C	19.900393	0.444130	5.706895

C	21.300334	0.414233	5.750774
C	21.984162	-0.792010	5.608681
C	21.258773	-1.955881	5.390425
C	19.867738	-1.958719	5.337386
C	19.192779	-0.752339	5.504890
F	21.921986	-3.099427	5.205580
O	22.359085	1.918621	2.838194
C	23.350897	0.892240	2.949485
C	22.725664	-0.378617	2.337389
C	21.235315	-0.023066	2.202244
C	21.286850	1.489129	2.002277
O	23.718456	5.058203	1.868598
C	23.649142	4.289729	0.672463
C	24.059945	5.284050	-0.408972
C	25.171254	6.090855	0.296203
C	24.904568	5.853605	1.803891
O	24.356900	1.138086	6.476540
C	24.037267	0.860341	7.852170
C	25.267831	0.147224	8.426218
C	26.395445	0.662843	7.524404
C	25.684500	0.705253	6.173970
F	19.611428	8.098684	9.002335
Sb	18.359956	8.178608	7.557662
F	17.712474	6.420873	7.995721
F	19.681211	7.276663	6.436516
F	17.042874	8.983193	8.645556
F	19.120753	9.853269	7.052042
F	17.223101	8.243938	6.023027
H	18.389888	7.993588	3.978706
H	15.969964	8.473733	4.155456
H	15.489096	7.850669	-0.081641
H	17.953677	7.427182	-0.312186
H	20.100853	4.474921	0.929140
H	20.358456	3.368898	-1.318996
H	20.957718	7.202428	-3.165230
H	20.700384	8.332413	-0.940136
H	22.867054	7.749544	1.169640
H	24.329776	9.810582	1.237031
H	20.886856	12.245517	2.063352
H	19.402115	10.210883	1.976547
H	25.058230	6.326725	4.705009
H	27.415433	6.314890	3.802707
H	28.969450	6.521944	7.803866
H	26.631006	6.550502	8.725792
H	22.942843	7.117178	10.128652
H	22.835760	5.675851	12.185068
H	25.109937	2.620334	10.200757
H	25.156140	4.008397	8.083418
H	21.565056	8.118758	7.521175
H	21.032916	10.415287	8.317359
H	25.221396	11.161120	9.020760
H	25.777678	8.833311	8.276184
H	16.143542	2.546080	6.486393
H	14.111743	2.390393	5.030392
H	16.504185	0.871604	1.797269
H	18.567319	1.054801	3.222027
H	21.873775	1.342923	5.889705
H	23.080415	-0.832650	5.647496
H	19.336324	-2.902495	5.166419
H	18.094242	-0.740308	5.463316

H	18.147355	4.241782	7.567077
H	17.204411	4.397865	9.851087
H	18.660366	-0.043464	8.070181
H	17.728300	0.153671	10.394475
H	23.600074	0.786641	4.023423
H	24.265900	1.207769	2.405739
H	22.888050	-1.271003	2.968495
H	23.163928	-0.585747	1.343306
H	20.689690	-0.262762	3.134457
H	20.744797	-0.548517	1.363680
H	21.508233	1.746493	0.941775
H	20.376720	2.031061	2.320399
H	22.620600	3.904615	0.571439
H	24.357165	3.430065	0.732261
H	23.193242	5.929373	-0.652116
H	24.400414	4.797891	-1.340184
H	25.134433	7.162758	0.030628
H	26.173230	5.719154	0.014282
H	25.738628	5.299644	2.288898
H	24.714163	6.779507	2.377638
H	18.056851	5.571957	5.804695
H	15.692917	6.113548	6.372327
H	14.465336	4.967510	2.389932
H	16.840055	4.349604	1.840300
H	20.214714	8.967712	4.823788
H	20.034894	11.385408	5.425792
H	24.331806	11.752536	5.296115
H	24.517560	9.326260	4.684238
H	20.527142	6.085678	8.259033
H	19.558622	6.424818	10.529100
H	20.643107	2.382571	11.606322
H	21.615274	2.040377	9.317168
H	23.120123	0.240908	7.891695
H	23.828321	1.823256	8.355750
H	26.118462	1.419598	5.447492
H	25.657718	-0.303382	5.703559
H	25.168218	-0.949789	8.315924
H	25.423543	0.362406	9.498304
H	27.291040	0.016262	7.526050
H	26.700257	1.685682	7.827295
H	28.015302	3.336600	2.261530
H	26.413726	2.511100	2.320508
H	28.911025	4.001784	4.712031
H	27.850737	3.314365	5.986807
H	28.579852	1.072083	5.417221
H	29.880510	1.863655	4.500205
H	28.795641	1.047456	2.521222
H	27.355142	0.584887	3.465370
H	25.452544	3.520912	4.822624
H	24.365334	2.794217	5.773227

Zero-point correction= 1.529637 (Hartree/Particle)
Thermal correction to Energy= 1.654589
Thermal correction to Enthalpy= 1.655533
Thermal correction to Gibbs Free Energy= 1.355885
Sum of electronic and zero-point Energies= -9636.539960
Sum of electronic and thermal Energies= -9636.415009
Sum of electronic and thermal Enthalpies= -9636.414064
Sum of electronic and thermal Free Energies= -9636.713712

	E (Thermal)	CV	S
	KCal/Mol	Cal/Mol-Kelvin	Cal/Mol-Kelvin
Total	1038.270	462.826	630.662

Cation 1 with PMe_3 and SMe fragments optimized at the M06/Def2-svp (Pd_3*small*)$^+$

57
scf done: -3080.825666

C	-2.177976	4.064980	1.299051
P	-1.868824	3.490477	-0.408476
C	-1.016997	4.913050	-1.181151
Pd	-0.766166	1.454814	-0.319879
S	1.430043	2.243615	-0.239125
C	1.893832	2.475628	-2.001090
Pd	1.689040	-0.051085	0.109258
S	1.242774	-2.318240	0.452785
C	1.663106	-3.091192	-1.159648
Pd	-0.887366	-1.423946	0.118891
S	-2.647493	0.080992	-0.182035
C	-3.156336	-0.151758	-1.931164
P	3.943029	-0.055656	0.644075
C	4.099847	0.205953	2.446605
P	-2.134964	-3.308816	0.627636
C	-2.386326	-3.374228	2.437152
C	5.019628	1.247445	-0.057383
C	4.915071	-1.578905	0.356624
C	-1.435192	-4.955244	0.245578
C	-3.835502	-3.425943	-0.037209
C	-3.530974	3.551185	-1.172312
H	1.519599	1.660047	-2.625572
H	1.481069	3.427641	-2.352388
H	2.985669	2.525295	-2.075651
H	-2.305403	-0.418864	-2.563688
H	-3.915393	-0.940588	-1.976895
H	-3.601779	0.781832	-2.293291
H	1.338739	-2.470024	-1.999015
H	2.747767	-3.237670	-1.207922
H	1.178682	-4.071942	-1.218503
H	-3.958730	4.558187	-1.072462
H	-4.193153	2.833645	-0.672678
H	-3.473715	3.297941	-2.237708
H	-2.772627	3.315804	1.835524
H	-2.715357	5.023102	1.298836
H	-1.221986	4.186081	1.822560
H	-0.854856	4.722665	-2.248816
H	-0.043779	5.066238	-0.698842
H	-1.618653	5.825644	-1.072431
H	-2.106021	-5.746987	0.605974
H	-0.460470	-5.064483	0.736605
H	-1.300988	-5.071687	-0.836335
H	-4.333223	-4.329605	0.340416
H	-3.810695	-3.468023	-1.132530
H	-4.413350	-2.545524	0.269458
H	-2.982470	-4.252714	2.719651
H	-2.902087	-2.464741	2.768087
H	-1.412306	-3.421006	2.939159
H	6.026494	1.182498	0.377208

H	4.597611	2.235465	0.164245
H	5.097853	1.132642	-1.144938
H	5.155153	0.214891	2.752095
H	3.575338	-0.595429	2.980873
H	3.636569	1.162232	2.718218
H	5.930945	-1.464335	0.758879
H	4.984287	-1.789466	-0.717164
H	4.428655	-2.427604	0.853055

Zero-point correction= 0.466556 (Hartree/Particle)
Thermal correction to Energy= 0.505669
Thermal correction to Enthalpy= 0.506613
Thermal correction to Gibbs Free Energy= 0.392937
Sum of electronic and zero-point Energies= -3080.359111
Sum of electronic and thermal Energies= -3080.319997
Sum of electronic and thermal Enthalpies= -3080.319053
Sum of electronic and thermal Free Energies= -3080.432729

	E (Thermal)	CV	S
	KCal/Mol	Cal/Mol-Kelvin	Cal/Mol-Kelvin
Total	317.312	134.404	239.251

Cation 1 with PMe_3 and SMe fragments optimized at the M06/Def2-svp with Def2-TZVP set for Pd (Pd_3*smallTZ*)$^+$

58
scf done: -3088.223150

C	-0.337201	-0.480599	6.394116
P	-1.169090	0.313776	4.977802
C	-1.457394	2.032897	5.522684
Pd	-0.004972	0.247970	2.941225
S	2.177874	0.197709	3.801817
C	2.471790	1.940268	4.312456
Pd	-0.006588	0.251768	0.027630
P	-1.173249	0.323272	-2.007299
C	-0.343522	-0.468618	-3.426276
Pd	2.521460	0.240966	1.482946
P	4.868465	0.301364	1.481488
C	5.678369	-0.481984	2.916949
S	-1.847117	0.252621	1.485408
C	-2.370241	2.016264	1.488279
S	2.175211	0.202996	-0.835616
C	2.469212	1.946402	-1.343293
C	-2.834640	-0.430668	-1.997767
C	-1.461119	2.043713	-2.548241
C	-2.830020	-0.441171	4.968653
C	5.676427	-0.479582	0.043631
C	5.502076	2.014911	1.482731
Li	0.813649	-1.888237	1.481811
H	-1.513765	2.696670	1.490114
H	-2.988309	2.192082	2.375997
H	-2.987078	2.195480	0.600381
H	2.050906	2.654626	3.599000
H	3.552743	2.095619	4.401720
H	2.023290	2.091104	5.300893
H	2.049066	2.659619	-0.628261
H	2.020011	2.099093	-2.331127
H	3.550128	2.101637	-1.433176
H	-2.027262	2.040067	6.461876

H	-0.503807	2.548951	5.684968
H	-2.023049	2.584576	4.762649
H	0.631197	-0.005563	6.588524
H	-0.964371	-0.395966	7.291785
H	-0.161509	-1.541899	6.183853
H	-3.477817	0.065393	4.244270
H	-2.767522	-1.499576	4.691494
H	-3.280665	-0.360924	5.967193
H	6.600676	2.007103	1.482175
H	5.153978	2.554998	0.594094
H	5.154849	2.553188	2.372813
H	6.769466	-0.404028	2.817382
H	5.369008	0.007315	3.847537
H	5.402230	-1.540864	2.977929
H	6.767649	-0.401466	0.141658
H	5.400490	-1.538455	-0.018409
H	5.365492	0.010893	-0.885816
H	-2.032163	2.053092	-3.486702
H	-2.025482	2.594262	-1.786432
H	-0.507425	2.559497	-2.710722
H	-3.287050	-0.346685	-2.995201
H	-2.772175	-1.490081	-1.724463
H	-3.480781	0.073641	-1.270328
H	-0.971755	-0.381965	-4.323006
H	0.624836	0.006396	-3.620947
H	-0.168003	-1.530390	-3.218275

Zero-point correction= 0.466631 (Hartree/Particle)
Thermal correction to Energy= 0.505679
Thermal correction to Enthalpy= 0.506623
Thermal correction to Gibbs Free Energy= 0.393400
Sum of electronic and zero-point Energies= -3080.470295
Sum of electronic and thermal Energies= -3080.431247
Sum of electronic and thermal Enthalpies= -3080.430302
Sum of electronic and thermal Free Energies= -3080.543526

	E (Thermal)	CV	S
	KCal/Mol	Cal/Mol-Kelvin	Cal/Mol-Kelvin
Total	317.318	134.366	238.298

Complex $(AuPMe_3)_3^+$ optimized at the M06/Def2-svp (Au_3*small*)$^+$

42
scf done: -1789.212752

C	5.418417	12.865548	3.264155
P	7.043206	13.099918	4.066747
C	7.892141	14.302499	2.984315
Au	8.264631	11.089703	4.490101
Au	9.898902	9.048197	3.627241
P	11.119835	7.985655	1.868021
C	12.042178	9.127994	0.780232
Au	9.044868	9.116238	6.244357
P	8.964985	8.154543	8.430354
C	7.999501	9.119581	9.645600
C	6.647912	14.068489	5.565613
C	10.582540	7.923233	9.247479
C	8.203510	6.494447	8.492501
C	12.386698	6.792071	2.422275
C	10.087116	7.019535	0.710759
H	4.920472	13.833439	3.117747

H	12.898234	6.342439	1.560849
H	11.082515	8.891500	9.363897
H	8.185788	6.114425	9.522824
H	7.986654	8.612668	10.619782
H	8.773865	5.800945	7.864216
H	7.178138	6.541103	8.108389
H	7.572601	14.352292	6.080764
H	8.084212	13.849609	2.005007
H	6.088680	14.976242	5.301922
H	6.043829	13.462040	6.250106
H	9.557807	6.229612	1.255699
H	12.781655	9.684555	1.367104
H	9.341236	7.673324	0.244561
H	10.705694	6.562733	-0.073539
H	12.557215	8.574748	-0.016454
H	11.350757	9.847525	0.327267
H	13.124846	7.299069	3.053925
H	11.915090	5.998119	3.012506
H	7.276442	15.201830	2.849238
H	8.853494	14.588042	3.426067
H	5.548969	12.378159	2.291297
H	4.783483	12.225052	3.886758
H	8.440763	10.115648	9.765658
H	6.969667	9.238900	9.290140
H	11.221660	7.277879	8.634317
H	10.451410	7.464853	10.236758

Zero-point correction= 0.344806 (Hartree/Particle)
Thermal correction to Energy= 0.374677
Thermal correction to Enthalpy= 0.375622
Thermal correction to Gibbs Free Energy= 0.275986
Sum of electronic and zero-point Energies= -1788.867946
Sum of electronic and thermal Energies= -1788.838075
Sum of electronic and thermal Enthalpies= -1788.837131
Sum of electronic and thermal Free Energies= -1788.936767

	E (Thermal)	CV	S
	KCal/Mol	Cal/Mol-Kelvin	Cal/Mol-Kelvin
Total	235.114	97.383	209.701

Complex $(AuPMe_3)_3^+$ optimized at the M06/Def2-svp with Def2-TZVP set for Au (Au_3*smallTZ*)$^+$

42
scf done: -1790.185114

C	5.428806	12.888664	3.255698
P	7.053391	13.102678	4.066355
C	7.914790	14.304033	2.990677
Au	8.252235	11.085348	4.489715
Au	9.873376	9.042176	3.627325
P	11.099727	7.983337	1.877102
C	12.018121	9.125656	0.784360
Au	9.018113	9.109178	6.237095
P	8.956274	8.149523	8.418449
C	7.965639	9.085176	9.637907
C	6.656779	14.076001	5.563058
C	10.580189	7.967662	9.238001
C	8.247587	6.465570	8.493132
C	12.377688	6.800470	2.432359

C	10.080540	7.003425	0.718019
H	4.942471	13.862697	3.111480
H	12.891023	6.354845	1.569913
H	11.055070	8.948950	9.349876
H	8.250270	6.092924	9.526239
H	7.972839	8.576537	10.611254
H	8.834961	5.785690	7.865710
H	7.218162	6.475635	8.117578
H	7.579383	14.353704	6.085121
H	8.102201	13.857103	2.007777
H	6.107292	14.988053	5.293776
H	6.042270	13.476778	6.244549
H	9.554815	6.210547	1.261984
H	12.754111	9.689444	1.368618
H	9.331580	7.647666	0.243716
H	10.708556	6.549172	-0.060195
H	12.537624	8.569598	-0.007418
H	11.325361	9.839520	0.324663
H	13.113608	7.313420	3.061723
H	11.915236	6.002318	3.024024
H	7.307218	15.209864	2.862500
H	8.879052	14.578588	3.433004
H	5.556778	12.403842	2.281314
H	4.783717	12.253170	3.872844
H	8.377469	10.093596	9.758522
H	6.930778	9.174721	9.288716
H	11.237352	7.335438	8.630327
H	10.459160	7.511681	10.229686

Zero-point correction= 0.344666 (Hartree/Particle)
Thermal correction to Energy= 0.373686
Thermal correction to Enthalpy= 0.374630
Thermal correction to Gibbs Free Energy= 0.278697
Sum of electronic and zero-point Energies= -1789.840449
Sum of electronic and thermal Energies= -1789.811428
Sum of electronic and thermal Enthalpies= -1789.810484
Sum of electronic and thermal Free Energies= -1789.906417

	E (Thermal)	CV	S
	KCal/Mol	Cal/Mol-Kelvin	Cal/Mol-Kelvin
Total	234.492	95.492	201.908

Complex $(AuPMe_3)_4^{++}$ optimized at the M06/Def2-svp $(Au_4$***small***$)^{++}$

56
scf done: -2385.426004

Au	8.840835	11.599654	4.663805
Au	9.656756	9.576736	6.501245
Au	10.590168	9.517947	3.803848
P	7.032617	13.006954	4.019709
P	8.986924	8.250164	8.360197
P	11.186060	8.122413	1.970382
Au	11.537375	11.522589	5.598696
P	13.422334	12.835068	6.221977
C	14.775729	11.861939	6.957825
C	13.042654	14.124294	7.452664
H	12.644676	13.671015	8.367348
C	14.186549	13.727260	4.828874

C	5.621647	12.094329	3.314518
C	6.328503	13.968037	5.398631
C	7.470540	14.247088	2.758682
C	12.427382	6.854010	2.383307
C	9.784843	7.195129	1.265205
C	11.896749	9.039526	0.565148
C	10.354687	7.311364	9.114330
C	7.726577	6.997817	7.955418
C	8.270788	9.218353	9.727630
H	4.822477	12.794926	3.038004
H	12.295249	14.819026	7.053279
H	13.953973	14.685309	7.699263
H	13.459459	14.406198	4.369578
H	14.424664	11.345701	7.858404
H	15.044663	14.312411	5.185719
H	14.531333	13.017485	4.068559
H	15.609610	12.523361	7.228114
H	15.133652	11.112673	6.242889
H	12.667480	6.262207	1.489928
H	11.133249	7.996206	9.468701
H	7.462338	6.428860	8.856835
H	7.982420	8.549566	10.549491
H	8.112329	6.305249	7.198941
H	6.824983	7.479414	7.560907
H	7.092375	14.622136	5.833636
H	7.841073	13.753841	1.853219
H	5.494970	14.584635	5.036824
H	5.960102	13.293602	6.179739
H	9.341740	6.541459	2.024758
H	12.799829	9.574527	0.879589
H	9.014652	7.887784	0.907785
H	10.130276	6.580348	0.423352
H	12.157369	8.343563	-0.243364
H	11.172732	9.769554	0.186143
H	13.343701	7.325691	2.755213
H	12.039423	6.184616	3.159182
H	6.586744	14.846510	2.502494
H	8.253221	14.912781	3.139155
H	5.935554	11.542132	2.421666
H	5.230226	11.379533	4.047067
H	9.002688	9.943898	10.099930
H	7.383877	9.762291	9.383790
H	10.796687	6.629981	8.378826
H	9.981146	6.724929	9.964355

Zero-point correction= 0.460136 (Hartree/Particle)
Thermal correction to Energy= 0.500750
Thermal correction to Enthalpy= 0.501694
Thermal correction to Gibbs Free Energy= 0.377877
Sum of electronic and zero-point Energies= -2384.965869
Sum of electronic and thermal Energies= -2384.925254
Sum of electronic and thermal Enthalpies= -2384.924310
Sum of electronic and thermal Free Energies= -2385.048128

	E (Thermal)	CV	S
	KCal/Mol	Cal/Mol-Kelvin	Cal/Mol-Kelvin
Total	314.225	131.835	260.596

Complex $(AuPMe_3)_4^{++}$ optimized at the M06/Def2-svp with Def2-TZVP set for Au $(Au_4 \textit{smallTZ})^{++}$

56
scf done: -2386.721401

Au	8.843787	11.596531	4.664196
Au	9.657588	9.577789	6.497523
Au	10.589638	9.519531	3.806814
P	7.041570	13.004289	4.019445
P	8.987880	8.253318	8.352996
P	11.184523	8.123954	1.978229
Au	11.533569	11.520319	5.597826
P	13.413914	12.832138	6.221405
C	14.779334	11.863611	6.942775
C	13.038762	14.111168	7.465294
H	12.645872	13.651191	8.378779
C	14.170962	13.742350	4.835075
C	5.623577	12.098260	3.317858
C	6.338309	13.974910	5.393165
C	7.477938	14.241155	2.753519
C	12.432455	6.859598	2.387227
C	9.787685	7.186137	1.276333
C	11.888222	9.035129	0.564657
C	10.351069	7.306839	9.107688
C	7.722792	7.003240	7.952389
C	8.275573	9.217138	9.726524
H	4.828428	12.803670	3.042091
H	12.289105	14.809037	7.075914
H	13.950979	14.670793	7.711634
H	13.441072	14.423892	4.384280
H	14.438853	11.336763	7.841150
H	15.027531	14.326773	5.196658
H	14.517303	13.043017	4.065959
H	15.608387	12.530664	7.213960
H	15.141322	11.123376	6.220559
H	12.670627	6.268455	1.492931
H	11.132633	7.986594	9.464974
H	7.459784	6.436240	8.855365
H	7.988133	8.544348	10.545400
H	8.104221	6.308206	7.196082
H	6.820783	7.485503	7.559766
H	7.102303	14.630395	5.825732
H	7.845667	13.746308	1.847837
H	5.506749	14.591142	5.026365
H	5.966718	13.306851	6.178108
H	9.349270	6.530274	2.036632
H	12.791317	9.574432	0.871279
H	9.012116	7.872076	0.917849
H	10.136783	6.572341	0.435327
H	12.147482	8.334381	-0.240113
H	11.161674	9.760999	0.182671
H	13.349253	7.333448	2.755064
H	12.050591	6.188590	3.164640
H	6.593799	14.840421	2.498279
H	8.261831	14.907810	3.129612
H	5.931254	11.542891	2.424910
H	5.228246	11.386808	4.051483
H	9.008514	9.939964	10.101937
H	7.388405	9.764002	9.388304

H	10.791554	6.623828	8.372867
H	9.972953	6.721205	9.956188

Zero-point correction= 0.460145 (Hartree/Particle)
Thermal correction to Energy= 0.500729
Thermal correction to Enthalpy= 0.501673
Thermal correction to Gibbs Free Energy= 0.378521
Sum of electronic and zero-point Energies= -2386.261256
Sum of electronic and thermal Energies= -2386.220672
Sum of electronic and thermal Enthalpies= -2386.219728
Sum of electronic and thermal Free Energies= -2386.342880

	E (Thermal)	CV	S
	KCal/Mol	Cal/Mol-Kelvin	Cal/Mol-Kelvin
Total	314.212	131.885	259.195

Complex Pd_3Li with PMe_3 and SMe fragments optimized at the M06/Def2-svp (Pd_3Li*small*)$^{++}$

58
scf done: -3084.626546

C	-0.347268	-0.484758	6.411005
P	-1.179275	0.308782	4.994638
C	-1.460459	2.031055	5.532325
Pd	-0.010159	0.247243	2.950130
S	2.185147	0.194569	3.814706
C	2.470456	1.940761	4.312658
Pd	-0.011786	0.250739	0.018723
P	-1.183469	0.318070	-2.024159
C	-0.353443	-0.472608	-3.443285
Pd	2.532064	0.239838	1.482951
P	4.888891	0.298688	1.481421
C	5.699389	-0.480757	2.918319
S	-1.862104	0.253163	1.485431
C	-2.367328	2.020672	1.488251
S	2.182455	0.199959	-0.848487
C	2.467825	1.947015	-1.343293
C	-2.847166	-0.430059	-2.012270
C	-1.464366	2.041728	-2.557553
C	-2.842628	-0.440111	4.982857
C	5.697318	-0.478426	0.042104
C	5.511230	2.016154	1.482633
Li	0.813709	-1.900782	1.482479
H	-1.504791	2.693255	1.489929
H	-2.983841	2.202633	2.375983
H	-2.982827	2.205963	0.600500
H	2.045800	2.648972	3.595462
H	3.550773	2.102004	4.400230
H	2.022288	2.095726	5.300741
H	2.043978	2.654009	-0.624415
H	2.018868	2.103966	-2.330698
H	3.548107	2.108116	-1.431521
H	-2.032779	2.046461	6.470003
H	-0.504555	2.542812	5.694455
H	-2.020823	2.582146	4.768009
H	0.623222	-0.012116	6.601195
H	-0.971799	-0.395893	7.310205
H	-0.175039	-1.547016	6.203266
H	-3.486198	0.068716	4.256159

H	-2.783939	-1.498763	4.706221
H	-3.295610	-0.357505	5.980231
H	6.609921	2.017428	1.481787
H	5.158668	2.553793	0.594243
H	5.160019	2.551802	2.372761
H	6.790471	-0.398355	2.821320
H	5.385494	0.007692	3.847877
H	5.427529	-1.540626	2.979551
H	6.788534	-0.396035	0.137557
H	5.425481	-1.538237	-0.020237
H	5.381923	0.011278	-0.886285
H	-2.037676	2.059489	-3.494590
H	-2.023672	2.591404	-1.791451
H	-0.508415	2.553407	-2.719596
H	-3.301795	-0.343583	-3.008565
H	-2.788366	-1.489770	-1.739715
H	-3.489260	0.076221	-1.282488
H	-0.978973	-0.381585	-4.341570
H	0.617002	0.000097	-3.633576
H	-0.181345	-1.535361	-3.237983

Zero-point correction= 0.468031 (Hartree/Particle)
Thermal correction to Energy= 0.508757
Thermal correction to Enthalpy= 0.509702
Thermal correction to Gibbs Free Energy= 0.396126
Sum of electronic and zero-point Energies= -3084.158515
Sum of electronic and thermal Energies= -3084.117788
Sum of electronic and thermal Enthalpies= -3084.116844
Sum of electronic and thermal Free Energies= -3084.230419

	E (Thermal)	CV	S
	KCal/Mol	Cal/Mol-Kelvin	Cal/Mol-Kelvin
Total	319.250	140.233	239.039

Complex Pd_3Li with PMe_3 and SMe fragments optimized at the M06/Def2-svp with Def2-TZVP set for Pd and Li (Pd_3Li*smallTZ*)$^{++}$

58
scf done: -3088.223150

C	-0.337201	-0.480599	6.394116
P	-1.169090	0.313776	4.977802
C	-1.457394	2.032897	5.522684
Pd	-0.004972	0.247970	2.941225
S	2.177874	0.197709	3.801817
C	2.471790	1.940268	4.312456
Pd	-0.006588	0.251768	0.027630
P	-1.173249	0.323272	-2.007299
C	-0.343522	-0.468618	-3.426276
Pd	2.521460	0.240966	1.482946
P	4.868465	0.301364	1.481488
C	5.678369	-0.481984	2.916949
S	-1.847117	0.252621	1.485408
C	-2.370241	2.016264	1.488279
S	2.175211	0.202996	-0.835616
C	2.469212	1.946402	-1.343293
C	-2.834640	-0.430668	-1.997767
C	-1.461119	2.043713	-2.548241
C	-2.830020	-0.441171	4.968653

C	5.676427	-0.479582	0.043631
C	5.502076	2.014911	1.482731
Li	0.813649	-1.888237	1.481811
H	-1.513765	2.696670	1.490114
H	-2.988309	2.192082	2.375997
H	-2.987078	2.195480	0.600381
H	2.050906	2.654626	3.599000
H	3.552743	2.095619	4.401720
H	2.023290	2.091104	5.300893
H	2.049066	2.659619	-0.628261
H	2.020011	2.099093	-2.331127
H	3.550128	2.101637	-1.433176
H	-2.027262	2.040067	6.461876
H	-0.503807	2.548951	5.684968
H	-2.023049	2.584576	4.762649
H	0.631197	-0.005563	6.588524
H	-0.964371	-0.395966	7.291785
H	-0.161509	-1.541899	6.183853
H	-3.477817	0.065393	4.244270
H	-2.767522	-1.499576	4.691494
H	-3.280665	-0.360924	5.967193
H	6.600676	2.007103	1.482175
H	5.153978	2.554998	0.594094
H	5.154849	2.553188	2.372813
H	6.769466	-0.404028	2.817382
H	5.369008	0.007315	3.847537
H	5.402230	-1.540864	2.977929
H	6.767649	-0.401466	0.141658
H	5.400490	-1.538455	-0.018409
H	5.365492	0.010893	-0.885816
H	-2.032163	2.053092	-3.486702
H	-2.025482	2.594262	-1.786432
H	-0.507425	2.559497	-2.710722
H	-3.287050	-0.346685	-2.995201
H	-2.772175	-1.490081	-1.724463
H	-3.480781	0.073641	-1.270328
H	-0.971755	-0.381965	-4.323006
H	0.624836	0.006396	-3.620947
H	-0.168003	-1.530390	-3.218275

Zero-point correction= 0.468108 (Hartree/Particle)
Thermal correction to Energy= 0.508763
Thermal correction to Enthalpy= 0.509707
Thermal correction to Gibbs Free Energy= 0.396225
Sum of electronic and zero-point Energies= -3087.755043
Sum of electronic and thermal Energies= -3087.714387
Sum of electronic and thermal Enthalpies= -3087.713443
Sum of electronic and thermal Free Energies= -3087.826926

	E (Thermal)	CV	S
	KCal/Mol	Cal/Mol-Kelvin	Cal/Mol-Kelvin
Total	319.254	140.155	238.844

Complex Pd_3Ag with PMe_3 and SMe fragments optimized at the M06/Def2-svp (Pd_3Ag*small*)$^{++}$

69
scf done: -3804.586962

C	-1.196322	1.906634	-2.412496

P	-1.134817	0.102542	-2.142999
C	-2.869957	-0.439720	-2.332901
Pd	-0.113664	-0.176953	-0.080640
S	2.069286	-0.273753	-0.921147
C	2.355763	-2.084126	-1.034025
Pd	-0.126575	0.003620	2.827582
S	2.057821	0.251670	3.671050
C	2.504335	-1.448349	4.212133
Pd	2.391611	0.080962	1.361713
P	4.567054	0.858425	1.276809
C	5.750154	0.148080	2.474310
S	-1.960166	-0.011556	1.353669
C	-2.600808	-1.732323	1.415145
P	-1.246108	0.392930	4.831929
C	-2.982098	-0.159531	4.989592
C	-0.306068	-0.546566	-3.635113
C	-1.342843	2.207634	5.028625
C	-0.483309	-0.161703	6.398350
C	4.521277	2.633667	1.684437
C	5.425835	0.786835	-0.330307
Ag	0.560276	2.275072	1.258825
H	1.908899	-2.625582	-0.194422
H	1.929193	-2.450439	-1.974924
H	3.437398	-2.261463	-1.049577
H	-1.788537	-2.462008	1.473975
H	-3.250400	-1.830520	2.291963
H	-3.194179	-1.914089	0.512232
H	2.135899	-2.204953	3.513134
H	3.595590	-1.516754	4.284852
H	2.075152	-1.626172	5.204700
H	-3.268638	-0.125372	-3.307161
H	-3.479776	0.005487	-1.536927
H	-2.937290	-1.531555	-2.259012
H	-1.864726	2.349887	-1.662858
H	-1.575842	2.143607	-3.415888
H	-0.198251	2.345423	-2.282941
H	-0.243209	-1.640204	-3.593439
H	0.709230	-0.135703	-3.693417
H	-0.862928	-0.256381	-4.536316
H	-1.088115	0.164006	7.255676
H	0.521617	0.268786	6.489736
H	-0.402303	-1.254833	6.418255
H	-3.418815	0.203238	5.930081
H	-3.031074	-1.254801	4.977760
H	-3.570224	0.228453	4.148921
H	-1.874717	2.478330	5.950973
H	-1.867195	2.642612	4.167615
H	-0.325754	2.619525	5.060394
H	6.428832	1.226122	-0.243307
H	4.847587	1.353113	-1.070510
H	5.514681	-0.250542	-0.674369
H	5.532720	3.060206	1.637087
H	4.112773	2.765269	2.694325
H	3.873981	3.161559	0.971791
H	6.724942	0.648336	2.395286
H	5.884831	-0.924723	2.293472
H	5.358203	0.288495	3.489548
F	2.021350	2.727991	-0.728102
O	1.368529	4.039474	2.612129
O	-0.941937	4.061705	0.734974

B	2.007919	4.128499	-1.001458
F	2.867163	4.429631	-1.995631
F	2.343656	4.775157	0.207758
F	0.674776	4.462064	-1.323760
H	1.872060	4.485040	1.904794
H	0.571154	4.569546	2.736978
H	-0.499496	4.396567	-0.074511
H	-1.889423	4.017360	0.572121

Zero-point correction= 0.536597 (Hartree/Particle)
Thermal correction to Energy= 0.588066
Thermal correction to Enthalpy= 0.589010
Thermal correction to Gibbs Free Energy= 0.451012
Sum of electronic and zero-point Energies= -3804.050366
Sum of electronic and thermal Energies= -3803.998897
Sum of electronic and thermal Enthalpies= -3803.997952
Sum of electronic and thermal Free Energies= -3804.135951

	E (Thermal)	CV	S
	KCal/Mol	Cal/Mol-Kelvin	Cal/Mol-Kelvin
Total	369.017	177.381	290.442

Complex Pd_3Ag with PMe_3 and SMe fragments optimized at the M06/Def2-svp with Def2-TZVP set for Pd and Ag (Pd_3Ag***smallTZ***)$^{++}$

69
scf done: -3804.741545

C	-1.099060	1.923074	-2.421852
P	-1.139662	0.120805	-2.137039
C	-2.904045	-0.326066	-2.304246
Pd	-0.114405	-0.189164	-0.077289
S	2.069889	-0.280800	-0.923546
C	2.362898	-2.090760	-1.034988
Pd	-0.118311	-0.008971	2.832387
S	2.066725	0.250584	3.674314
C	2.518922	-1.444574	4.225870
Pd	2.395934	0.066998	1.363003
P	4.567684	0.864399	1.280601
C	5.753216	0.161523	2.480023
S	-1.958106	-0.023696	1.363924
C	-2.592909	-1.746975	1.416352
P	-1.243288	0.381814	4.836002
C	-2.970190	-0.196777	4.996387
C	-0.365983	-0.588462	-3.631315
C	-1.366563	2.196478	5.013389
C	-0.470585	-0.148955	6.405415
C	4.505592	2.638363	1.691978
C	5.430094	0.798156	-0.324548
Ag	0.575299	2.255541	1.244313
H	1.905162	-2.633248	-0.202089
H	1.951909	-2.458350	-1.982260
H	3.445337	-2.263899	-1.034285
H	-1.777680	-2.473006	1.478166
H	-3.248407	-1.850903	2.288100
H	-3.178646	-1.927122	0.508143
H	2.150536	-2.206107	3.532479
H	3.610503	-1.509626	4.296664
H	2.092334	-1.616327	5.220640

H	-3.296197	0.007910	-3.274594
H	-3.479078	0.151651	-1.501477
H	-3.028868	-1.412699	-2.227195
H	-1.661965	2.421380	-1.621779
H	-1.541695	2.181138	-3.393666
H	-0.065953	2.290460	-2.376564
H	-0.395277	-1.683537	-3.592511
H	0.679857	-0.262814	-3.687678
H	-0.896145	-0.250599	-4.532002
H	-1.076011	0.179265	7.261320
H	0.530342	0.292129	6.489031
H	-0.378898	-1.241039	6.435387
H	-3.411004	0.158899	5.937656
H	-3.002021	-1.292657	4.983482
H	-3.565168	0.182971	4.156864
H	-1.906830	2.469847	5.930089
H	-1.892678	2.613453	4.144588
H	-0.355559	2.622911	5.045234
H	6.431010	1.241650	-0.234869
H	4.851522	1.361914	-1.066043
H	5.523757	-0.239072	-0.667869
H	5.514056	3.072751	1.652032
H	4.089677	2.764456	2.699671
H	3.858874	3.163926	0.977415
H	6.724454	0.669045	2.403571
H	5.895714	-0.910054	2.297998
H	5.358113	0.298084	3.494483
F	2.045918	2.734683	-0.733868
O	1.325299	4.038971	2.597020
O	-0.971934	3.979500	0.672728
B	2.019394	4.136614	-0.999631
F	2.885212	4.452598	-1.983516
F	2.339347	4.779595	0.217278
F	0.686707	4.458341	-1.332369
H	1.834527	4.479669	1.889226
H	0.511850	4.551526	2.690270
H	-0.530262	4.337141	-0.127171
H	-1.918297	3.918935	0.509309

Zero-point correction= 0.536706 (Hartree/Particle)
Thermal correction to Energy= 0.588010
Thermal correction to Enthalpy= 0.588954
Thermal correction to Gibbs Free Energy= 0.451736
Sum of electronic and zero-point Energies= -3804.204839
Sum of electronic and thermal Energies= -3804.153536
Sum of electronic and thermal Enthalpies= -3804.152591
Sum of electronic and thermal Free Energies= -3804.289809

	E (Thermal)	CV	S
	KCal/Mol	Cal/Mol-Kelvin	Cal/Mol-Kelvin
Total	368.982	177.320	288.799

Complex Pd_3Au with PMe_3 and SMe fragments optimized at the M06/Def2-svp (Pd_3Au*small*)$^{++}$

71
scf done: -3677.037587

C	-0.372300	-0.377664	6.439516
P	-1.154355	0.349885	4.958636

C	-1.344193	2.118311	5.377965
Pd	-0.012667	-0.055205	2.962263
Au	0.829032	-2.369846	1.511832
P	0.881647	-4.723478	1.525406
Pd	-0.014407	-0.068622	0.039851
P	-1.157996	0.320028	-1.958581
C	-0.377090	-0.418311	-3.434732
Pd	2.520596	-0.061590	1.499901
P	4.823001	0.310858	1.494488
C	5.708204	-0.388652	2.929645
S	-1.854864	-0.047848	1.502004
C	-2.388705	1.709227	1.494416
S	2.171912	-0.102774	-0.822531
C	2.471096	1.627724	-1.357562
S	2.174799	-0.080252	3.822729
C	2.472364	1.655781	4.340877
C	-2.859084	-0.340297	-2.008509
C	-1.349050	2.085296	-2.390464
C	-2.855442	-0.309918	5.014666
C	5.702400	-0.411532	0.067029
C	5.312684	2.070667	1.479082
H	-1.536351	2.395641	1.489210
H	-3.007367	1.892909	2.380394
H	-3.011066	1.883857	0.609187
H	2.047368	2.374449	3.633383
H	3.552946	1.816764	4.429194
H	2.031808	1.805770	5.333385
H	2.052784	2.353613	-0.653458
H	2.024494	1.770701	-2.348360
H	3.551536	1.784573	-1.454774
H	-1.879315	2.222582	6.331859
H	-0.363127	2.598247	5.471294
H	-1.909365	2.642047	4.598559
H	0.619544	0.057266	6.607239
H	-0.997546	-0.191575	7.323152
H	-0.252600	-1.458794	6.306397
H	-3.478618	0.161252	4.246379
H	-2.843544	-1.389817	4.828510
H	-3.299357	-0.120841	6.001315
H	6.408006	2.156815	1.476702
H	4.918724	2.571301	0.587231
H	4.921211	2.586543	2.363315
H	6.788260	-0.219694	2.823674
H	5.363899	0.074184	3.861379
H	5.519695	-1.466147	2.995957
H	6.782856	-0.240616	0.165498
H	5.513787	-1.489991	0.019230
H	5.353735	0.035782	-0.870680
H	-1.885009	2.182424	-3.344649
H	-1.914098	2.613967	-1.614283
H	-0.368457	2.565362	-2.487828
H	-3.303995	-0.157341	-2.995865
H	-2.847261	-1.418993	-1.815555
H	-3.481272	0.135841	-1.242499
H	-1.003950	-0.240454	-4.318915
H	0.613834	0.016641	-3.607708
H	-0.255621	-1.498148	-3.292985
C	1.743942	-5.437429	0.087948
C	1.728652	-5.421091	2.979858
C	-0.764146	-5.504049	1.521306

H	1.720033	-6.518097	2.929153
H	2.767116	-5.073196	3.015128
H	1.223248	-5.101653	3.897971
H	1.732242	-6.533867	0.149503
H	1.250283	-5.126014	-0.839249
H	2.783594	-5.092286	0.061578
H	-0.657952	-6.597096	1.528558
H	-1.332412	-5.195293	2.405722
H	-1.322975	-5.206084	0.627249

Zero-point correction= 0.582743 (Hartree/Particle)
Thermal correction to Energy= 0.632029
Thermal correction to Enthalpy= 0.632973
Thermal correction to Gibbs Free Energy= 0.498083
Sum of electronic and zero-point Energies= -3676.454845
Sum of electronic and thermal Energies= -3676.405558
Sum of electronic and thermal Enthalpies= -3676.404614
Sum of electronic and thermal Free Energies= -3676.539505

	E (Thermal)	CV	S
	KCal/Mol	Cal/Mol-Kelvin	Cal/Mol-Kelvin
Total	396.604	168.690	283.901

Complex Pd_3Au with PMe_3 and SMe fragments optimized at the M06/Def2-svp with Def2-TZVP set for Pd and Au (Pd_3Li***smallTZ***)$^{++}$

71
scf done: -3677.472609

C	-0.369601	-0.377737	6.440475
P	-1.152525	0.349079	4.959938
C	-1.339733	2.118510	5.375287
Pd	-0.009800	-0.058715	2.960942
Au	0.832855	-2.378960	1.512099
P	0.877359	-4.727994	1.525727
Pd	-0.011587	-0.072566	0.040894
P	-1.156425	0.319261	-1.960039
C	-0.375287	-0.418099	-3.436309
Pd	2.521759	-0.063674	1.499609
P	4.826168	0.317880	1.494449
C	5.713525	-0.377631	2.929950
S	-1.855760	-0.051511	1.501910
C	-2.383982	1.707542	1.494120
S	2.175766	-0.106173	-0.825371
C	2.470280	1.626591	-1.356359
S	2.178679	-0.082923	3.825002
C	2.471789	1.655224	4.339202
C	-2.857949	-0.339545	-2.009549
C	-1.344468	2.085721	-2.387464
C	-2.853808	-0.309780	5.015891
C	5.708244	-0.400401	0.066822
C	5.303201	2.080892	1.479067
H	-1.528638	2.390086	1.489609
H	-3.002840	1.892822	2.379603
H	-3.005369	1.883967	0.608591
H	2.045128	2.370252	3.629166
H	3.551910	1.818732	4.428246
H	2.030492	1.806155	5.331222
H	2.050164	2.348763	-0.649656

H	2.022972	1.770527	-2.346685
H	3.550247	1.786065	-1.454323
H	-1.874313	2.226161	6.329050
H	-0.357726	2.596814	5.466750
H	-1.904111	2.640957	4.594466
H	0.623686	0.055004	6.605093
H	-0.992372	-0.189151	7.325288
H	-0.252886	-1.459243	6.307809
H	-3.475681	0.159938	4.245699
H	-2.841321	-1.390040	4.831818
H	-3.299005	-0.118723	6.001541
H	6.397737	2.175822	1.475292
H	4.903699	2.578434	0.587984
H	4.908390	2.593056	2.363945
H	6.792668	-0.201492	2.826778
H	5.363792	0.080810	3.861800
H	5.531596	-1.456461	2.993134
H	6.787806	-0.223023	0.163265
H	5.525649	-1.479999	0.021283
H	5.355143	0.043390	-0.870860
H	-1.880848	2.186608	-3.340964
H	-1.907587	2.613343	-1.609198
H	-0.362772	2.563646	-2.483969
H	-3.304246	-0.154577	-2.995865
H	-2.845962	-1.418586	-1.818491
H	-3.478504	0.135558	-1.241597
H	-0.999635	-0.236750	-4.321517
H	0.617397	0.014043	-3.606021
H	-0.257590	-1.498491	-3.295653
C	1.736475	-5.449147	0.089267
C	1.719697	-5.432905	2.980071
C	-0.771378	-5.503436	1.520737
H	1.704234	-6.529680	2.926331
H	2.760227	-5.091728	3.017494
H	1.215836	-5.113092	3.898832
H	1.717106	-6.545328	0.153239
H	1.245921	-5.136585	-0.839117
H	2.778470	-5.111428	0.062482
H	-0.667688	-6.596692	1.528082
H	-1.339649	-5.193386	2.404609
H	-1.329247	-5.204246	0.626580

Zero-point correction= 0.582693 (Hartree/Particle)
Thermal correction to Energy= 0.631992
Thermal correction to Enthalpy= 0.632936
Thermal correction to Gibbs Free Energy= 0.498115
Sum of electronic and zero-point Energies= -3676.889916
Sum of electronic and thermal Energies= -3676.840618
Sum of electronic and thermal Enthalpies= -3676.839673
Sum of electronic and thermal Free Energies= -3676.974494

	E (Thermal)	CV	S
	KCal/Mol	Cal/Mol-Kelvin	Cal/Mol-Kelvin
Total	396.581	168.732	283.754

Complex 2-Au (Pd_3Au) optimized at the M06/Def2-svp with Def2-TZVP set for Pd and Au

160

scf done: -8668.686217

C	5.140742	2.250479	-0.079838
C	5.762045	1.415231	0.858395
C	7.073034	1.706971	1.265436
C	7.749056	2.807011	0.751631
C	7.106313	3.617735	-0.177097
C	5.810414	3.352173	-0.603227
P	4.904287	-0.078073	1.474155
Pd	2.554537	0.147457	1.477235
S	2.219530	0.245181	3.807162
C	2.681081	-1.333017	4.483758
C	2.671958	-2.508173	3.728096
C	3.108789	-3.701361	4.289320
C	3.539262	-3.712472	5.615440
C	3.527000	-2.551255	6.390309
C	3.099070	-1.359064	5.819939
Cl	4.081824	-5.197759	6.322854
F	7.741606	4.668015	-0.666275
C	5.487977	-1.460830	0.449306
C	4.766013	-2.663323	0.486718
C	5.193720	-3.765612	-0.242452
C	6.336642	-3.648308	-1.025724
C	7.067884	-2.466496	-1.084508
C	6.640356	-1.370591	-0.343327
F	6.731738	-4.681231	-1.747930
C	5.622079	-0.354598	3.129199
C	5.608824	0.696313	4.058666
C	6.091089	0.508184	5.346067
C	6.560852	-0.751140	5.708856
C	6.580126	-1.810492	4.813029
C	6.111308	-1.606131	3.518397
F	6.982928	-0.944101	6.947675
S	2.178343	0.269247	-0.858516
Pd	-0.008973	0.122510	0.026741
S	-1.850656	0.202502	1.511847
C	-2.650924	-1.384833	1.564862
C	-1.965517	-2.558575	1.886996
C	-2.649734	-3.764625	1.974269
C	-4.017950	-3.791409	1.707514
C	-4.710518	-2.629124	1.361677
C	-4.024549	-1.422834	1.297250
Cl	-4.872115	-5.297243	1.763728
Pd	0.014103	0.126372	2.962755
P	-1.139906	-0.147614	5.019207
C	-2.930768	-0.455357	4.838575
C	-3.755655	0.581503	4.376203
C	-5.109023	0.364362	4.158105
C	-5.628390	-0.908789	4.375749
C	-4.836388	-1.953520	4.828692
C	-3.484370	-1.720167	5.063958
F	-6.908665	-1.129691	4.125794
C	2.581585	-1.292446	-1.607918
C	1.955044	-2.482825	-1.230679
C	2.275713	-3.672894	-1.870831
C	3.241989	-3.666702	-2.875815
C	3.882884	-2.485613	-3.255980
C	3.549694	-1.296399	-2.619554
Cl	3.681095	-5.154855	-3.646013
P	-1.193232	-0.092105	-1.997934
C	-0.132302	-0.377252	-3.455322

C	0.678081	0.669476	-3.921235
C	1.560412	0.467379	-4.972783
C	1.649146	-0.801236	-5.539808
C	0.861740	-1.854968	-5.099594
C	-0.032970	-1.636898	-4.055772
F	2.519724	-1.006754	-6.513893
C	-2.383360	-1.464336	-1.969687
C	-3.654832	-1.367178	-2.549440
C	-4.510785	-2.463242	-2.543684
C	-4.084971	-3.650686	-1.956878
C	-2.830524	-3.770840	-1.369214
C	-1.985907	-2.668270	-1.368474
F	-4.904474	-4.686428	-1.944634
C	-2.133654	1.419703	-2.417382
C	-2.470676	1.705534	-3.749584
C	-3.221398	2.830745	-4.068240
C	-3.638082	3.672749	-3.042804
C	-3.325619	3.413339	-1.713798
C	-2.569600	2.286053	-1.405910
F	-4.348704	4.746259	-3.338808
C	-0.510990	-1.540102	6.003847
C	-0.392045	-1.486434	7.398779
C	0.034794	-2.604680	8.107039
C	0.339798	-3.770382	7.411333
C	0.237613	-3.846888	6.026874
C	-0.181068	-2.722816	5.326275
F	0.748032	-4.828889	8.088177
C	-1.044054	1.336007	6.082135
C	-2.008224	1.574076	7.074330
C	-1.909212	2.679981	7.909827
C	-0.838411	3.552883	7.746327
C	0.131244	3.343150	6.773865
C	0.022084	2.233147	5.942484
F	-0.743322	4.610064	8.533401
Au	0.826493	2.414727	1.494356
F	0.459655	-4.154527	2.687756
B	0.890524	-4.693168	1.453724
F	0.077094	-4.147837	0.433538
F	0.806000	-6.051747	1.464770
F	2.213410	-4.261409	1.232819
H	-3.335565	1.575824	4.185017
H	-2.857666	-2.544017	5.419834
H	-0.632001	-0.569503	7.946370
H	-2.849633	0.884271	7.200500
H	0.784891	2.058674	5.176740
H	-0.241319	-2.789798	4.234211
H	-5.773721	1.157717	3.807198
H	0.135813	-2.592807	9.195037
H	0.499092	-4.770731	5.502875
H	-4.557273	-0.501600	1.036675
H	-0.881937	-2.561905	2.041354
H	-2.648379	2.880768	8.689141
H	-5.283124	-2.940419	4.976324
H	-2.110011	-4.682905	2.221131
H	-5.782794	-2.678684	1.152754
H	0.957469	4.053022	6.684283
H	0.617061	1.661249	-3.457932
H	-0.655234	-2.467626	-3.708379
H	-3.990256	-0.432620	-3.010013
H	-2.143736	1.037630	-4.553700

H	-2.326297	2.070718	-0.359733
H	-1.008789	-2.770836	-0.882000
H	2.195829	1.268542	-5.358416
H	-5.508548	-2.416410	-2.986882
H	-2.530148	-4.710561	-0.896782
H	4.043308	-0.361788	-2.908977
H	1.231567	-2.509945	-0.409408
H	-3.493969	3.068544	-5.099449
H	0.967207	-2.836057	-5.570034
H	1.793335	-4.605813	-1.568602
H	4.638236	-2.509167	-4.046222
H	-3.690181	4.094940	-0.940662
H	5.217805	1.679796	3.772289
H	6.123926	-2.440487	2.809712
H	7.213820	-0.439562	-0.393890
H	7.580118	1.063354	1.991935
H	4.121182	2.026602	-0.413650
H	3.853500	-2.764856	1.086125
H	6.100121	1.313518	6.084607
H	7.959713	-2.423196	-1.714663
H	4.634425	-4.705003	-0.225171
H	3.090536	-0.438299	6.413923
H	2.297816	-2.520148	2.699359
H	8.770822	3.048811	1.054363
H	6.948968	-2.785430	5.143008
H	3.090114	-4.620612	3.697813
H	3.860244	-2.589786	7.431125
H	5.352004	4.011799	-1.344747
P	0.972704	4.719198	1.025764
C	-0.560428	5.673454	1.249702
C	2.243470	5.624452	1.962479
C	1.419245	4.945692	-0.725928
H	-1.359912	5.227081	0.639206
H	-0.870692	5.642828	2.305015
H	1.511289	6.014731	-0.978784
H	2.375010	4.436354	-0.925502
H	0.645510	4.483700	-1.359602
H	2.296935	6.673495	1.627833
H	2.004733	5.598054	3.036396
H	3.222931	5.143763	1.814860

Zero-point correction= 1.135687 (Hartree/Particle)
Thermal correction to Energy= 1.238104
Thermal correction to Enthalpy= 1.239048
Thermal correction to Gibbs Free Energy= 0.988579
Sum of electronic and zero-point Energies= -8667.550530
Sum of electronic and thermal Energies= -8667.448113
Sum of electronic and thermal Enthalpies= -8667.447169
Sum of electronic and thermal Free Energies= -8667.697638

	E (Thermal)	CV	S
	KCal/Mol	Cal/Mol-Kelvin	Cal/Mol-Kelvin
Total	776.922	384.004	527.156

8. References

1. Rigaku, CrystalClear-SM Expert 2.1 b43. **2015**.

2. a) G. M. Sheldrick. *SHELXT* – Integrated space-group and crystal-structure determination. *Acta Crystallogr. Sect. Found. Adv.* **2015**, *71***,** 3–8; b) G. M. Sheldrick. Crystal structure refinement with *SHELXL*. *Acta Crystallogr. Sect. C Struct. Chem.* **2015**, *71***,** 3–8.

3. I. A. Guzei. An idealized molecular geometry library for refinement of poorly behaved molecular fragments with constraints. *J. Appl. Crystallogr.* **2014**, *47***,** 806–809.

4. Gaussian 09, Revision **A.1**, Frisch, M. J.; Trucks, G. W.; Schlegel, H. B.; Scuseria, G. E.; Robb, M. A.; Cheeseman, J. R.; Scalmani, G.; Barone, V.; Mennucci, B.; Petersson, G. A.; Nakatsuji, H.; Caricato, M.; Li, X.; Hratchian, H. P.; Izmaylov, A. F.; Bloino, J.; Zheng, G.; Sonnenberg, J. L.; Hada, M.; Ehara, M.; Toyota, K.; Fukuda, R.; Hasegawa, J.; Ishida, M.; Nakajima, T.; Honda, Y.; Kitao, O.; Nakai, H.; Vreven, T.; Montgomery, Jr., J. A.; Peralta, J. E.; Ogliaro, F.; Bearpark, M.; Heyd, J. J.; Brothers, E.; Kudin, K. N.; Staroverov, V. N.; Kobayashi, R.; Normand, J.; Raghavachari, K.; Rendell, A.; Burant, J. C.; Iyengar, S. S.; Tomasi, J.; Cossi, M.; Rega, N.; Millam, N. J.; Klene, M.; Knox, J. E.; Cross, J. B.; Bakken, V.; Adamo, C.; Jaramillo, J.; Gomperts, R.; Stratmann, R. E.; Yazyev, O.; Austin, A. J.; Cammi, R.; Pomelli, C.; Ochterski, J. W.; Martin, R. L.; Morokuma, K.; Zakrzewski, V. G.; Voth, G. A.; Salvador, P.; Dannenberg, J. J.; Dapprich, S.; Daniels, A. D.; Farkas, Ö.; Foresman, J. B.; Ortiz, J. V.; Cioslowski, J.; Fox, D. J. Gaussian, Inc., Wallingford CT, 2009.

5. Zhao, Y.; Truhlar, D. G. *Theor. Chem. Account* **2008**, *120*, 215.

6. Weigen, F.; Ahlrichs, R. *Phys. Chem. Chem. Phys.* **2005**, *7*, 3297; Andrae, D.; Haeussermann, U.; Dolg, M.; Stoll, H.; Preuss, H. *Theor. Chim. Acta* **1990**, *77*, 123; Peterson, K. A.; Figgen, D.; Goll, E.; Stoll, H.; Dolg, M. *J. Chem. Phys.* **2003**, *119*, 11113.

7. Hay, J. P.; Wadt, W. R. *J. Chem. Phys.* **1985**, *82*, 299; b) Friesner, R. A.; Murphy, R. B.; Beachy, M. D.; Ringlanda, M. N.; Pollard, W. T.; Dunietz, B. D.; Cao, Y. X. *J. Phys. Chem. A* **1999**, *103*, 1913.

8. A. Bergner, M. Dolg, W. Kuechle, H. Stoll, H. Preuss *Mol. Phys.* **1993**, *80*, 1431; b) M. Kaupp, P. v. R. Schleyer, H. Stoll, H. Preuss *J. Chem. Phys.* **1991**, *94*, 1360; c) M. Dolg, H. Stoll, H. Preuss, R.M. Pitzer, *J. Phys. Chem.* **1993**, *97*, 5852.

9. Head-Gordon, M.; Pople, J. A.; Frisch, M. J. *Chem. Phys. Lett.* **1988**, *153*, 503.

10. T. Schwabe, S. Grimme *Phys. Chem. Chem. Phys.* **2007**, *9*, 3397.

11. a) X. Huang, H.-J. Zhai, B. Kiran, L.-S. Wang, *Angew. Chem. Int. Ed.* **2005**, *44*, 7251; b) H.-J. Zhai, B. B. Averkiev, D. Y. Zubarev, L.-S. Wang, A. I. Boldyrev, *Angew. Chem. Int. Ed.* **2007**, *46*, 4277.

12. a) D. Y. Zubarev, A. I. Boldyrev, *Phys. Chem. Chem. Phys.* **2008**, *10*, 5207; b) A. S. Ivanov, I. A. Popov, A I Boldyrev, V. V. Zhdankin, *Angew. Chem. Int. Ed.* **2014**, *53*, 9617.

13. C. Albrecht; S. Schwieger, C. Bruhn, C. Wagner, R. Kluge, H. Schmidt, D. Steinborn, *J. Am. Chem. Soc.* **2007**, *129*, 4551.
